# Integrated phylogenetic analyses reveal the evolutionary, biogeographic, and diversification history of Asian warty treefrog genus *Theloderma* (Anura, Rhacophoridae)

**DOI:** 10.1002/ece3.10829

**Published:** 2023-12-21

**Authors:** Tao Luo, Xin‐Rui Zhao, Chang‐Ting Lan, Wei Li, Huai‐Qing Deng, Ning Xiao, Jiang Zhou

**Affiliations:** ^1^ School of Life Science Guizhou Normal University Guiyang China; ^2^ School of Karst Sciences Guizhou Normal University Guiyang China; ^3^ Guiyang Healthcare Vocational University Guiyang China

**Keywords:** biogeography, diversification, Indochina Peninsula, phylogeny

## Abstract

Asian warty treefrogs, genus *Theloderma*, are morphologically variable arboreal frogs endemic to Southeast Asia and Southern China. However, integrated systematic studies are lacking, and knowledge of the genus in terms of diversity, origin, and historical diversification remains limited. To address these knowledge gaps, we used three mitochondrial and five nuclear gene fragments to reconstruct the *Theloderma* phylogeny, estimate divergence times, and examine the biogeography of the genus. Phylogenetic and species delimitation analyses suggest that the genus *Theloderma* comprises three major clades corresponding to two subgenera and seven species groups, and mPTP identified at least 12 putative cryptic species, suggesting that species diversity has been underestimated. Biogeographic analyses indicated that most recent common ancestor of *Theloderma* originated in the Indochina Peninsula during the Middle Oligocene (ca. 27.77 Ma) and the splitting of Clade A to C occurred in the Late Oligocene (ca. 23.55–25.57 Ma). Current biogeographic patterns result from two distinct processes: in situ diversification in the Indochina Peninsula and dispersal in multiple areas, namely southward dispersal to the Malay Peninsula and Borneo, northeastward dispersal to Southern China, northward dispersal to the Himalayas, and dispersal from Southern China to the Indochina Peninsula. Ancestral character reconstruction suggests that the ancestor of *Theloderma* may have possessed a small body size, rough dorsal skin, and absence of vomerine teeth and hand webbing, and that these four characters have undergone multiple evolutions. Principal component analysis based on eight bioclimatic variables did not clearly distinguish the three major clades of *Theloderma*, suggesting that species in these clades may occupy similar climatic ecological niches. Our research highlights the importance of orogeny and paleoclimatic changes, in shaping amphibian biodiversity in mountain ecosystems.

## Graphical abstract

The most comprehensive nucleotide data has been applied to the genus *Theloderma*. *Theloderma* originated in the middle Oligocene Indochina Peninsula and later dispersed outward into the Malay Peninsula, Borneo, Himalayas, and Southern China. Elevational uplift drove early clade diversification, and innovations in key characteristics contributed to the outward spread of *Theloderma* to survive the dramatically changed early environment.

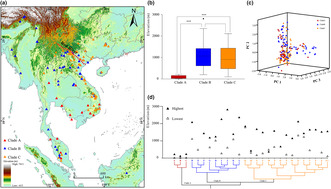

## 1 INTRODUCTION

An important goal of biogeography is to reveal the environmental factors that drive the origins of diversity and the patterns of species distributions. The selection of unique biota for biogeographic studies can therefore help to improve our understanding of how large‐scale geoclimatic environments drive the evolution of species diversity. The family Rhacophoridae is a highly diverse group of arboreal amphibians with two subfamilies, 24 genera, and 455 species, comprising about 5.3% of the order Anura, and are widely distributed in East Asia, Southeast Asia, Sundaland, Himalayas, Indochina Peninsula, and Africa (Frost, 2023). Several representative studies suggest that the Rhacophoridae originated in Africa in the Early Eocene by dispersal from the Indian subcontinent, and that orogeny, paleoclimate, and reproductive transitions have combined to shape the current patterns of species richness (Chen et al., 2020; Ellepola et al., 2022; Li et al., 2013). The focus of these studies on exploring the evolution of species diversification and distribution patterns at a large scale (genus level) and the lack of studies of species within genera may have led to the limited understanding of key evolutionary information.

**FIGURE 1:**
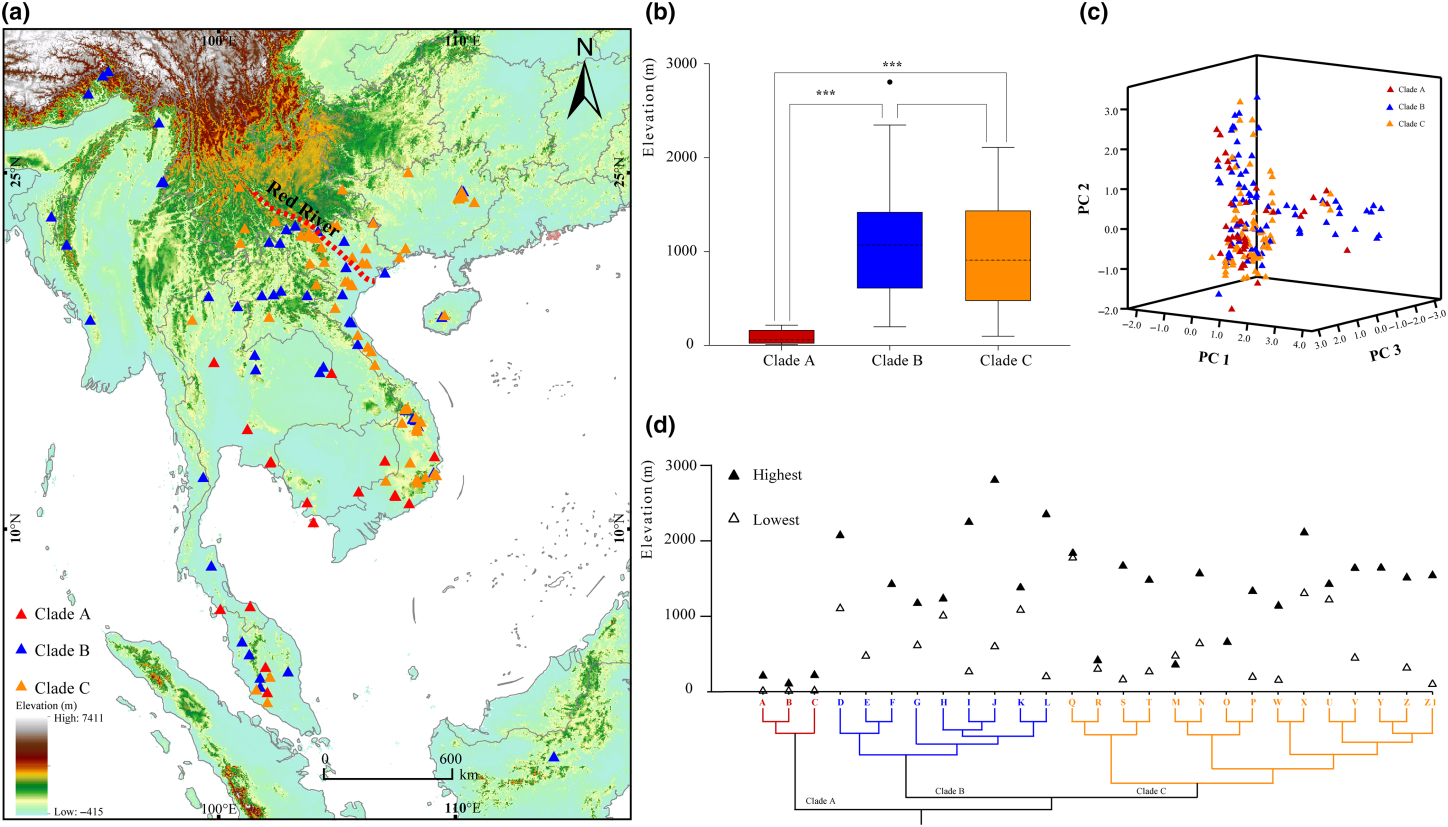
Distribution of *Theloderma* and elevational differentiation among its three clades. (a) Geographic distribution. (b) Elevation differentiation among the three clades. (c) Principal component analyses of the eight bioclimatic variables. (d) Highest (▲) and lowest (△) elevations documented for 27 species were mapped onto a simplified phylogenetic tree; the species names corresponding to the letters in this tree are listed in Table S5.

The Asian warty treefrog, genus *Theloderma* (Anura, Rhacophoridae), is endemic to Southeast Asia, South Asia, and Southern China and currently containing 29 species (Figure 1) (Frost, 2023). Previous phylogenetic studies have supported *Theloderma* as a sister clade to *Nyctixalus* (Chan et al., 2018, 2020; Chen et al., 2020; Poyarkov et al., 2018), and it can be further divided into two subgenera (*Stelladerma* and *Theloderma*) and six species groups, that is, *T. leporosum* group, *T. leave* group, *T. lateriticum* group, *T. corticale* group, *T. moloch* group, and *T. asperum* group (Poyarkov et al., 2015). According to several biogeographic studies, the genus formed in the Middle Oligocene (Li et al., 2013)/Late Eocene (Ellepola et al., 2022)/Early Miocene (Chen et al., 2020) by the dispersal of the ancestor of *Nyctixalus* through the Sundaland. Species within this genus have variable morphological characters, such as body size (less than 20 mm to more than 50 mm), dorsal skin (rough or smooth, i.e., the presence of calcified tubercles/asperities), hand webbing (absent or present) and vomerine teeth (absent or present) (Nguyen et al., 2015; Poyarkov et al., 2015). A study of reconstructions of four characters (body size, vomerine teeth, vocal opening, and hand webbing) for 16 species of *Theloderma* suggests that their ancestors exhibited either large or small body sizes and absence of vomerine teeth, vocal opening, and hand webbing (Nguyen et al., 2015). Trends in the spatial and temporal evolution of these characters are also unknown due to the lack of sufficient representative species and evolutionary time. To date, no detailed study of *Theloderma* has been conducted, based on large‐scale genetic data on species diversity, phylogeny, and biogeography.

To better understand the biodiversity of the Indochina Peninsula and adjacent areas and the process of historical diversification, here we assess the diversity of *Theloderma* and present its historical biogeographic patterns. In this study, we collected all reported sequence data of *Theloderma* from NCBI, as well as the most recent DNA sequences of three species of *Theloderma* collected from western Guangxi, China. We infer phylogenetic relationships, species boundaries, divergence times, and reconstruct ancestral characters for the genus. We also investigate the historical diversification and biogeographic processes of *Theloderma* in relation to orogeny and climatic transitions.

## 2 MATERIALS AND METHODS

### 2.1 Tissue samples and genetic sequences collection

Sampling at the species group level of *Theloderma* to acquire samples to employ in subsequent analyses will help improve phylogenetic resolution. Therefore, a total of six samples of three species (*T. corticale*, *T. lacustrinum*, *T. albopunctatum*) were collected from the Bangliang Gibbon National Nature Reserve, Jingxi City, Guangxi, China, representing the three species groups *T. lateriticum*, *T. corticale*, and *T. asperum*. Muscle samples used for molecular analysis were preserved in 95% alcohol and stored at −20°C. We downloaded a total of 508 nucleotide sequences from the NCBI nucleotide database for the ingroup and outgroup, including three mitochondrial and five nuclear genes (Table S1). We also collected distribution data of the species from the GBIF database (https://www.gbif.org/) and the published literature (Table S2).

### 2.2 Extraction, PCR amplification, and sequencing

Genomic DNA was extracted from the six muscle tissue samples using a DNA extraction kit (Tiangen Biotech Co., Ltd., Beijing). From the genomic DNA, we amplified the mitochondrial genes 12S rRNA plus 16S rRNA plus the complete tRNA of valine (12–16S) and cytochrome oxidase subunit I (COI), as well as the nuclear genes brain‐derived neurotrophic factor gene (BDNF), rhodopsin (RHOD), seventh‐in‐absentia (SIA), tyrosinase (TYR), and recombination activating gene 1 (RAG1). The amplification primers used are listed in Table S3. PCR amplifications were performed in 20‐μL reaction volumes under the following cycling conditions: an initial denaturing step at 95°C for 3 min; 36 cycles of denaturing at 94°C for 1 min, annealing at 55°C (for 12–16S), 50°C (for COI), 57°C (for BDNF, RHOD, TYR, and SIA), or 52°C (for RAG1) for 3 min, and extension at 72°C for 45 s; followed by a final extension step at 72°C for 7 min. PCR products were purified using spin columns. The products were sequenced on an ABI Prism 3730 automated DNA sequencer by TSING KE Biological Technology Co. Ltd (Chengdu, Sichuan, China). All newly obtained sequences have been submitted to GenBank (Table S1).

### 2.3 Phylogenetic analyses and species delimitation

The nucleotide sequences obtained in this study were first assembled and edited using DNASTAR LASERGENE v7.1. They were then aligned in MEGA v7.0 (Kumar et al., 2016) using default settings, after which they were visually inspected for accuracy and trimmed to reduce missing nucleotides. We constructed three datasets for molecular analyses: dataset 1 consisted of three combined mitochondrial fragments (12S, 16S, and COI) and was used for Bayesian inference (BI), dataset 2 contained only 16S sequences and was used for species delimitation, and dataset 3 consisted of the combined mitochondrial and nuclear sequences and was used to infer phylogenetic frameworks and estimate divergence times. Best‐fit partitioning scheme and corresponding evolutionary model for the data sets (by gene fragment) were selected using PartitionFinder v2.1.1 (Lanfear et al., 2016) based on the Bayesian information criterion (Table S4). The nucleotide data used for molecular analysis and the resulting raw tree data were deposited in the Dryad digital repository (https://doi.org/10.5061/dryad.79cnp5j1j).

A multi‐locus phylogenetic reconstruction of dataset 3 was performed using BI and maximum likelihood (ML) methods, both using a combined matrix of three mitochondrial and five nuclear genes. ML analysis was performed using IQ‐tree v2.0.4 (Nguyen et al., 2014) based on the best‐fit model with 2000 ultrafast bootstrap (UFB) replicates and 1000 Shimodaira–Hasewaga likelihood ratio tests (SH‐aLRT) (Hoang et al., 2018). The ML analysis was performed until a correlation coefficient of at least 0.99 was reached. BI was performed using MrBayes v3.2.1 (Ronquist et al., 2012). Each BI analysis was run independently using four Markov Chain Monte Carlo chains (three heated chains and one cold chain) starting with a random tree; each chain was run for 2 × 10^7^ generations and was sampled every 1000 generations. The data runs were estimated to have converged when the average standard deviation of split frequencies was less than 0.01, and Tracer v1.7.1 (Rambaut et al., 2018) indicated that the effective sample size was greater than 200. Outgroups were selected for phylogenetic analyses based on previous studies (Chen et al., 2020; Li et al., 2013). Nodes in the trees were considered well supported when the Bayesian posterior probability (BPP) was ≥0.95 (Ronquist et al., 2012), the ML ultrafast bootstrap value (UFB) was ≥95%, and the SH‐aLRT value was ≥80% (Hoang et al., 2018). Uncorrected *p*‐distance models (1000 replicates) were used to assess inter‐species differences in MEGA v7.0 (Kumar et al., 2016). In addition, we counted the number of variant sites and parsimony informative sites in nucleotide datasets 1 and 3 using MEGA v7.0 (Kumar et al., 2016).

The 16S rRNA gene tree for species delimitation was inferred for all samples using BI analysis. The best model was selected based on the Bayesian information criteria using PartitionFinder v2.1.1 (Lanfear et al., 2016), and Bayesian analysis was performed in MrBayes v3.2.1 (Ronquist et al., 2012). The BI analysis was run for a total of 2 × 10^7^ generations, with sampling every 1000 generations. We used Tracer v 1.7.1 (Rambaut et al., 2018) to assess convergence by checking the average standard deviation of split frequencies (less than 0.01). After BI analysis, consensus trees were generated using the first 10% of the total tree as a burn‐in.

To assess the presence of cryptic species among species represented by released 16S sequences within the genus *Theloderma*, we used four approaches for species delimitation: Automatic Barcode Gap Discovery (ABGD; Puillandre et al., 2012), Assemble Species by Automatic Partitioning (ASAP; Puillandre et al., 2021), Bayesian implementation of the Poisson Tree Processes (bPTP; Zhang et al., 2013), and multi‐rate Poisson Tree Processes (mPTP; Kapli et al., 2017). ABGD and ASAP enable rapid assessment of species boundaries based on sequence differences. Before implementing these two analyses, we estimated the “Transition/Transversion” values (2.53) of dataset 2 using the software MEGA v7.0 (Kumar et al., 2016) based on the Kimura 2‐parameter model. ABGD was run on an online server (https://bioinfo.mnhn.fr/abi/public/abgd/) using Kimura (K80 model) corrected distances, the default maximum intraspecific divergence (0.001–0.1), 100 steps, and relative gap width *X* (0.1). ASAP was run on an online server (https://bioinfo.mnhn.fr/abi/public/asap/) using Kimura (K80 model) corrected distances and default parameters. bPTP and mPTP are two tree‐based methods of species delimitation. bPTP was analyzed using an online server (http://species.h‐its.org/) based on a rooted 16S BI tree and default parameters. For the mPTP analysis, a BI tree constructed using 16S was used as the input tree and the online server (http://mptp.h.its.org) was used for analysis. ML species delimitation inference within the mPTP was implemented with the ‐*multi* option to account for differences in merging rates between species, with a minimum branch length of 0.0001.

### 2.4 Divergence‐time estimation

Divergence times for the *Theloderma* phylogeny based on dataset 3 were estimated using BEAST v1.8.2 (Drummond et al., 2012) with an uncorrelated relaxed clock and a Yule tree prior. The BEAST analysis included the following five calibration constraints: (1) the divergence between the Buergeriinae and Rhacophorinae subfamilies occurred at 50.8 Ma (Li et al., 2013) with a normal prior distribution (mean = 39.1, SD = 3.3); (2) the divergence between the Liuixalini tribe and the Nyctixalini + Rhacophorini tribes occurred at 44.3 Ma (normal prior, SD = 3.52); (3) the divergence between Nyctixalini and Rhacophorini occurred at 40.4 Ma (normal prior, SD = 3.52); (4) the split between the genera *Theloderma* and *Nyctixalus* occurred at 22.28 Ma (normal prior, SD = 3.0); and (5) the divergence of *T. vietnamense* from the remaining species in the genus *Theloderma* occurred at 21.43 Ma (normal prior, SD = 1.5) (Chen et al., 2020). BEAST was run for 2 × 10^7^ generations with all parameters yielding a posterior probability distribution with an effective sample size >200; the effective sample size was checked using Tracer v1.7.2 (Rambaut et al., 2018). Maximum clade credibility (MCC) trees were obtained using Treeannotator v2.4.1 (Drummond et al., 2012) by applying a burn‐in (as states) of 10%, with the posterior probability limited to 0.50.

### 2.5 Biogeographic

Biogeographic history of *Theloderma* was inferred using the R package BioGeoBEARS v1.1.1 (Matzke, 2013a) implemented in RASP v4.0 (Yu et al., 2015) in comparison to the test model and using the BEAST‐generated time tree without outgroups as input. Based on relevant previous biogeographic studies (Chen et al., 2018, 2020; Yuan et al., 2021), five biogeographic regions were used: (A) Malay Peninsula; (B) Borneo; (C) Indochina Peninsula; (D) Southern China; and (E) Himalayas. Maximum range‐size was set to two, because living species of *Theloderma* do not occur in more than two biogeographic areas. An additional jump parameter (+J) was also used because this parameter can reveal founder‐event speciation, which may result from long‐distance dispersal and subsequent colonizing lineage divergence (Matzke, 2013b, 2014). Therefore, six models, namely the Dispersal‐Extinction‐Cladogenesis (DEC) (Ree & Smith, 2008), an ML version of the Dispersal‐Vicariance Analysis (DIVALIKE) (Ronquist, 1997), and a version of the BI of historical biogeography for discrete areas (BAYAREALIKE) (Landis et al., 2013), as well as the corresponding “+J” models, were fitted using the corrected Akaike Information Criterion and Akaike weights to obtain the most suitable model for ancestral range reconstruction.

### 2.6 Speciation and diversification

To assess and visualize trends in speciation rates and lineage accumulation over time, Bayesian analysis of macroevolution mixtures (BAMM) and a lineage‐through‐time (LTT) plot were incorporated into this analysis. We constructed an LTT plot in Tracer v1.7.2 (Rambaut et al., 2018) based on the data obtained from the BEAST analysis. We estimated the dynamics of diversification rates using BAMM v2.5.0 (Rabosky et al., 2013) and BAMMtools v2.2.0 (Rabosky, Donnellan, et al., 2014; Rabosky, Grundler, et al., 2014), both of which explicitly model changes in diversification rates along the tree to explore speciation rates and net diversification rates in the phylogeny. First, we performed a species‐extinction analysis to infer speciation rates, net diversification rates, and potential rate changes based on the time trees generated by BEAST (after the removal of outliers) using BAMM (Rabosky et al., 2013), running the analysis for 1 × 10^7^ generations with sampling every 1000 generations. The output datafile was analyzed using the R package BAMMtools v 2.2.0 (Rabosky, Donnellan, et al., 2014; Rabosky, Grundler, et al., 2014), and the first 10% of the sampled data were discarded as burn‐in. Because of the small number of endpoints in the time tree (<500 tips), we assigned an a priori value of 1.0 to the variable “*expectedNumberOfShifts*.” We ran the dataset with three different configurations, that is, all species (containing *Theloderma* and nine outgroups), only *Theloderma*, and non‐*Theloderma* (i.e., nine outgroups). In the BAMM analysis, considering incomplete and random sampling, we specified a percentage of non‐random sampling for each clade, allowing for different sampling probabilities for each evolutionary branch. We constructed phylorate and rate through time (RTT) plots reflecting speciation rates over evolutionary time. We further conducted a macro‐evolutionary cohort analysis using BAMMtools to summarize the extent to which species shared macro‐evolutionary rate dynamics (Rabosky, Donnellan, et al., 2014).

### 2.7 Ancestral character reconstruction

To explore trends in the evolution of characters in the genus *Theloderma* across temporal and spatial scales, we reconstructed ancestral characters following the selection of three of the four morphological characters body size (average = 40 mm), vomerine teeth (present or absent), and hand webbing (present or absent) and dorsal skin (smooth or rough) by Nguyen et al. (2015). Morphological data used for ancestral character reconstruction were obtained from this study and previously published literature (Table S5). ML analyses of the BEAST‐generated time tree were run in MESQUITE v3.70 (Maddison & Maddison, 2019) using the Markov k‐state 1 (Mk1) parameter model likelihood method (Lewis, 2001) and the “Track character history” option. BI was performed using the Bayesian Binary Method (BBM) as implemented in RASP v4.0 (Yu et al., 2015). The fixed Jukes‐Cantor model was used, the temperature of the heated chains was set to 0.1, and the maximum number of areas was set to 1. Ten MCMC chains were run in parallel for 5 × 10^5^ generations, with sampling every 100 generations and an initial burn‐in of 25%.

### 2.8 Differentiation between elevation and bioclimatic variables

Statistical analyses were conducted to explore whether the three main clades of *Theloderma* were shaped by elevation and environmental factors. We used OvitalMap v9.3.4 (https://www.ovital.com/) to determine elevation based on collected distribution information and actual field survey data (Table S2). After excluding overlapping sites, there were 44, 82, and 83 records of species in Clade A, Clade B, and Clade C, respectively. Because the distribution of elevations among clades was near‐normal, we used the Kruskal–Wallis test (a non‐parametric *H*‐test) to determine the significance of elevation differences. To characterize climatic differences among the three clades, we downloaded data for elevation and 19 bioclimatic variables from the WorldClim database (http://www.worldclim.org) (Hijmans et al., 2005). To reduce the multicollinearity between bioclimatic variables, we used ENMTools v1.3 (Warren et al., 2010) to analyze correlations between the 19 bioclimatic variables (*Pearson*'s *r* < .80; Dormann et al., 2013), resulting in the selection of low‐correlated bioclimatic variables (Table S6). We further used the R package Raster (Hijmans et al., 2015) to extract the values of seven bioclimatic variables (Bio1 = Annual Mean Temperature; Bio2 = Mean Diurnal Range; Bio3 = Isothermality; Bio7 = Temperature Annual Range; Bio12 = Annual Precipitation; Bio14 = Precipitation of Driest Month; Bio15 = Precipitation Seasonality; Bio19 = Precipitation of Coldest Quarter) (Table S6) corresponding to 213 occurrence sites for principal component analysis (PCA) and independent sample *t*‐tests (1000 bootstrap replicates, Levene's test) using SPSS v21.0 (SPSS, Chicago, IL, USA).

## 3 RESULTS

### 3.1 Nucleotide dataset information

The following mitochondrial and nuclear fragments were obtained from six freshly collected specimens: 12–16S (2020 bp), COI (714 bp), RAG1 (1164 bp), BDNF (654 bp), RHOD (315 bp), SIA (441 bp), and TYR (612 bp). Datasets 1 and 3 contained 2734 and 5926 nucleotide sites, respectively. In dataset 1, 1608 sites were variable and 1378 sites were parsimony informative. In dataset 3, 1992 sites were variable and 1502 sites were parsimony informative.

### 3.2 Phylogenetic analyses and species boundaries

**FIGURE 2:**
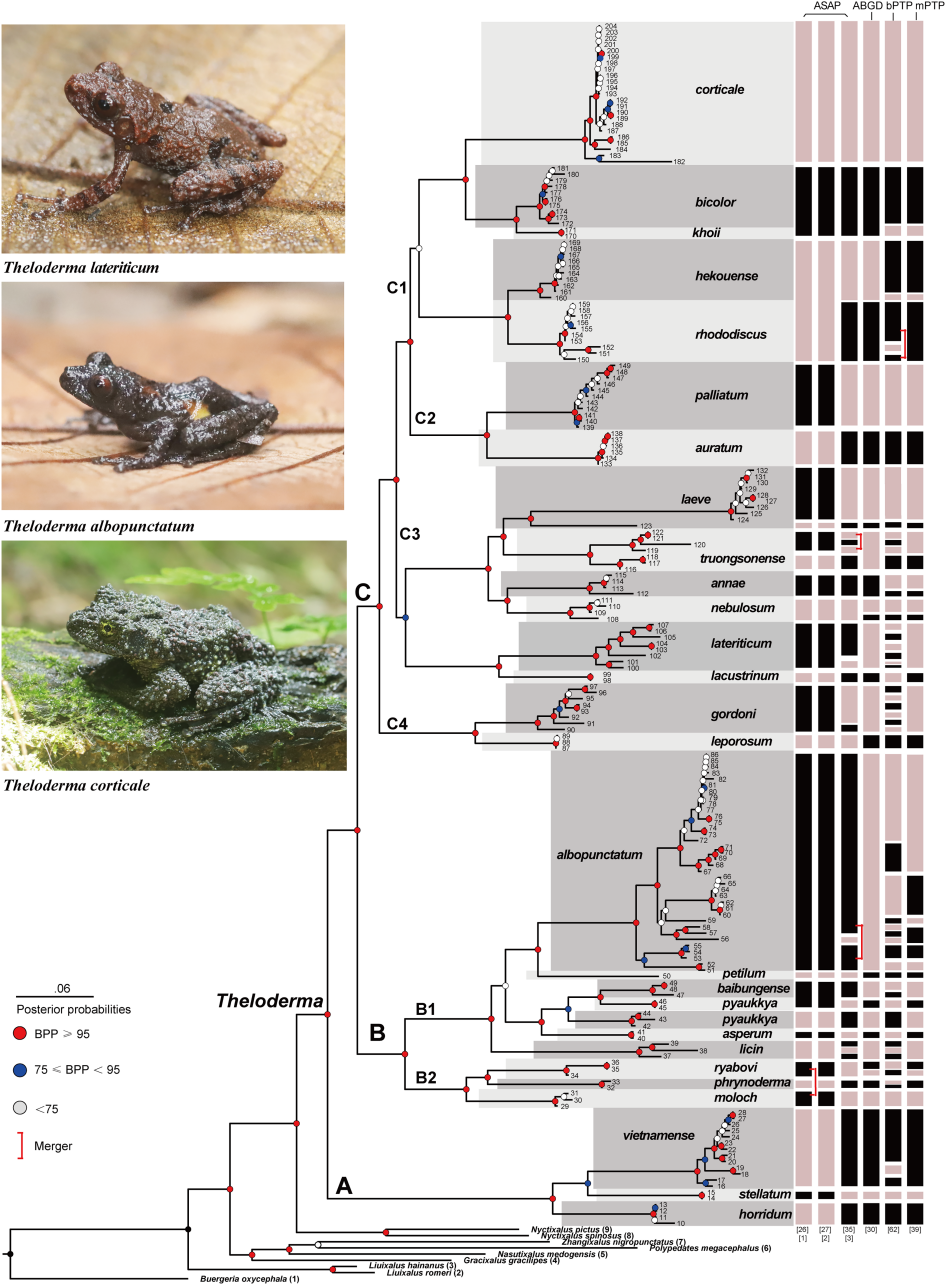
Bayesian inference tree‐based on dataset 1 and an assessment of *Theloderma* species delimitation using ASAP, ABGD, bPTP, and mPTP based on 16S. In the phylogenetic tree, the colored circles at the nodes indicate the BPP values from the BI analysis. The scale bar represents 0.06 nucleotide substitutions per site. The black and light tan boxes to the right of the tree denote the putative species identified by ASAP, ABGD, bPTP, and mPTP. Photographs depict the morphological variation within clades B and C. Numbers at branch tips correspond to the ID numbers given in Table S1.

**FIGURE 3:**
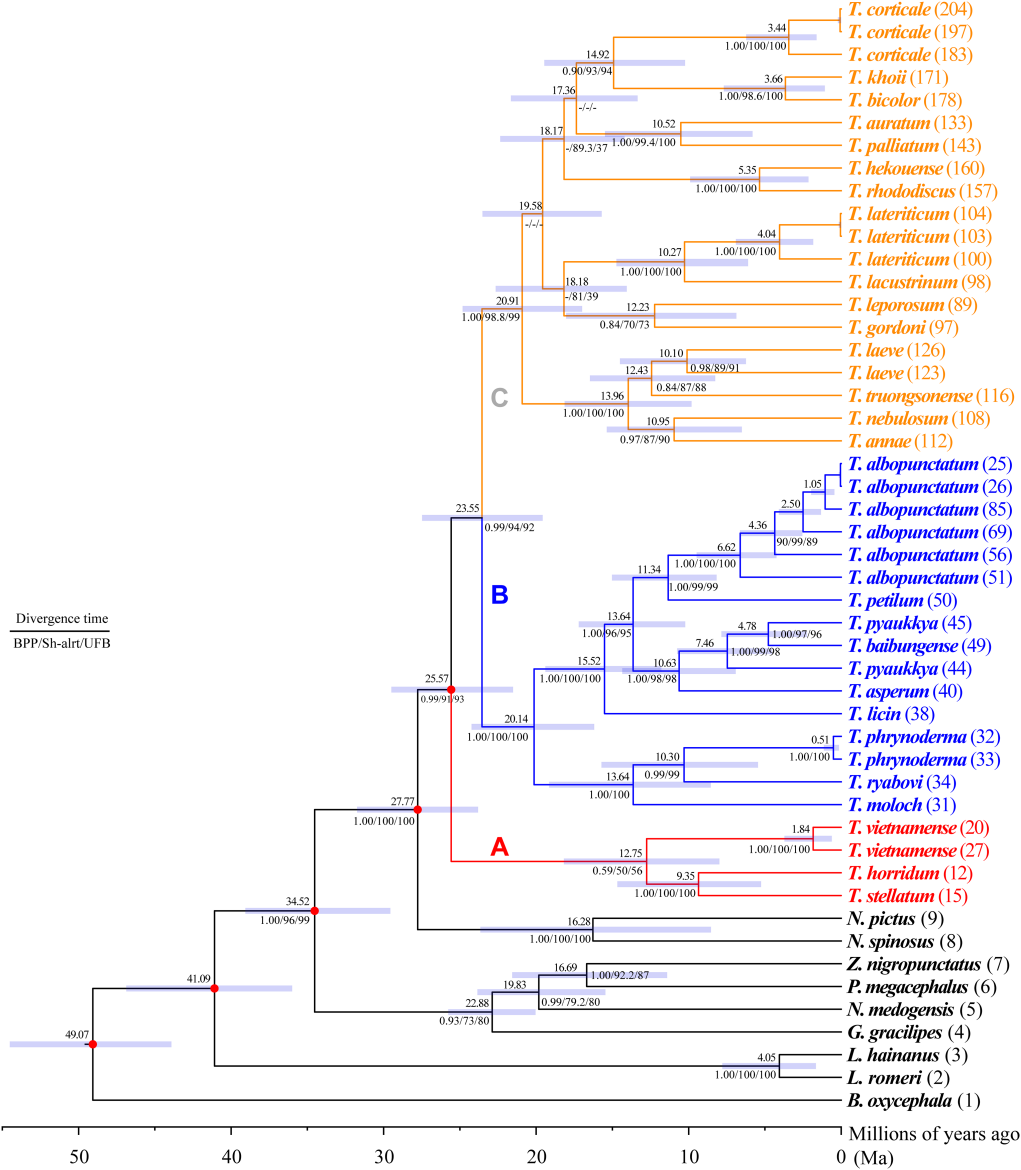
Evolutionary timescales and diversification of *Theloderma*s. Red circles indicate the five calibration points. Number to the top left of each node represents the mean age of the node; numbers to the bottom left of each node represent the BPP value, Shimodaira–Hasewaga likelihood ratio tests (Sh‐alrt) value, and the ultrafast bootstrap support (UFB) value from the BI and ML analyses, respectively. The light blue bar at each node depicts the 95% credibility interval.

Bayesian inference (BI) analysis of dataset 1 recovered a phylogenetic tree containing three well‐resolved major clades (clades A–C; BPP = 1.00) (Figure 2). Clades B and C were further divided into two and four subclades, subclades B1–B2 and subclades C1–C4 (BPP ≥ 0.95). The phylogeny based on the BI and maximum likelihood (ML) generated from dataset 3 obtained a topology consistent with the mitochondrial tree, with three major clades bounded by elevation and geographical distribution and with moderate resolution (Figures 1 and 3) (BPP ≥ 0.95, SH‐aLRT ≥ 91, UFB ≥ 92).

Assemble species by automatic partitioning analysis (scheme 26) recovered two phylogenetically distinct clades within each of the morphological species *T. truongsonense* and *T. laeve*; this scheme also merged the following pairs of morphological species: *T. ryabovi* and *T. moloch*, *T. baibungense* and *T. pyaukkya*, *T. hekouense* and *T. rhododiscus*, and *T. khoii* and *T. bicolor*. In contrast, scheme 27 supported the monophyly of *T. ryabovi* and *T. moloch* (Figure 2; Figure S1). A total of eight partitions were recovered through ABGD analysis, and a prior maximum intra‐species distance (*P*) of 0.001000–0.035938 delimited between 20 and 63 species (Figure 2; Table 1). Thirty species were identified based on a conservative partitioning scheme (i.e., partition 6, distance K80 Kimura MinSlope = 1.50, partition with prior maximal distance *P* = 0.012915) (Figure 2), and this scheme indicated high levels of intra‐specific sequence divergence (Table S7). The bPTP tree‐based species delimitation identified a greater number of species (62) compared to ASAP and ABGD, that is, samples with differences in branch length within the tree were identified as putative species (Figure 2; Figure S2). This occurred in, for example, *T. truongsonense* (four species), *T. lateriticum* (six species), *T. gordoni* (six species), and *T. albopunctatum* (nine species), and thus may have resulted in an inflated number of putative species. Compared to the three previous methods (ABGD, ASAP, and bPTP), the mPTP method proved to be the most stable, as mPTP allows for different evolutionary rates for each species. The species delimitation results of mPTP were in general agreement with the species‐level phylogeny based on dataset 1. In contrast to the bPTP, which is also tree‐based, the mPTP suggested that *T. rhododiscus*, *T. lateriticum*, *T. gordoni*, *T. baibungense*, *T. licin*, *T. phrynoderma*, and *T. vietnamense* were a single species. In the mPTP analysis, *T. hekouense*, *T. laeve*, *T. truongsonense*, *T. annae*, *T. nebulosum*, *T. albopunctatum*, and *T. ryabovi* were divided into two, two, two, two, six, and two species, respectively, strongly suggesting the presence of multiple unidentified cryptic species (Figure 2; Figure S3).

**TABLE 1:** Species delimitation analysis results obtained using ABGD.

Scheme	Number of species	Prior maximal distance
Partition 1	20 (63)	0.001
Partition 2	20 (51)	0.001668
Partition 3	20 (49)	0.002783
Partition 4	20 (41)	0.004642
Partition 5	20 (34)	0.007743
**Partition 6**	**20 (30)**	**0.012915**
Partition 7	20 (20)	0.021544
Partition 8	20 (20)	0.035938

*Note*: Partitions, number of species (initial run, followed by recursive run in parenthesis), and corresponding prior maximal distance obtained from the ABGD analysis using the K80 distance model and a relative gap width *X* = 1.5. Best fit is highlighted in bold.

### 3.3 Elevation and environmental differences among the three major clades

Although the three clades were not strictly geographically distinct (Figure 1a), species in these clades are found at different elevations, and there are significant elevational differences between clades A and C and clades B and C (Figure 1b): the mean elevation of species in Clade A was 261 ± 258 m (*N* = 47), that of species in Clade B was 1009 ± 551 m (*N* = 82), and that of species in Clade C was 1010 ± 541 m (*N* = 84) (Figure 1b,d; Table S9). The non‐parametric *H*‐test identified a statistically significant difference in elevation between Clade A and Clade B (*p*‐value < .05), as well as between Clade A and Clade C (*p*‐value < .05); there was no significant difference in elevation between Clade B and Clade C (Figure 1b).

Principal component analyses (PCA) based on eight bioclimatic variables did not clearly distinguish the three clades, suggesting that these clades occupy similar climatic ecological niches (Figure 1c; Table 2). Consistent with this, all three clades are distributed in the Southeast Asian tropical monsoon climate zone, with similar levels of temperature and rainfall. Independent sample *t*‐tests identified significant differences between Clade A and Clade B, as well as between Clade A and Clade C, with respect to elevation and bioclimatic variables (*p*‐value < .05; Table 3).

**TABLE 2:** Results of the principal component analysis based on 213 occurrence sites and eight low correlation bioclimatic variables.

Variables	PC1	PC2	PC3
BIO1 = Annual mean temperature	0.483	−0.001	0.757
BIO2 = Mean monthly temperature range	0.015	0.969	0.037
BIO3 = Isothermality	0.895	0.251	0.247
BIO7 = Temperature annual range	−0.793	0.249	−0.405
BIO12 = Annual precipitation	0.632	−0.310	−0.167
BIO14 = Precipitation of driest month	0.870	0.023	−0.411
BIO15 = Precipitation seasonality	−0.876	−0.088	0.264
BIO19 = Precipitation of coldest quarter	0.914	0.064	−0.234
Eigenvalues	4.424	1.173	1.121
Percentage of total variance	55.294	14.666	14.012
Cumulative percentage	55.294	69.960	83.972

**TABLE 3:** Independent sample *t*‐tests based on seven bioclimatic variables and elevation for occurrence sites of Clade A, Clade B, and Clade C.

Variables	Clade A versus clade B		Clade B versus clade C		Clade A versus clade C	
	*t*	*p*‐value	*t*	*p*‐value	*t*	*p*‐value
Elevation	−8.686	.000	−1.051	.295	−9.902	.000
BIO1 = Annual mean temperature	9.292	.000	3.416	.001	14.337	.000
BIO2 = Mean monthly temperature range	−0.456	.650	4.172	.000	1.280	.203
BIO3 = Isothermality	−2.180	.031	4.534	.000	9.115	.000
BIO7 = Temperature annual range	−5.848	.000	−2.163	.032	−9.209	.000
BIO12 = Annual precipitation	−0.456	.650	3.270	.001	2.634	.010
BIO14 = Precipitation of driest month	−2.180	.031	2.864	.005	0.233	.817
BIO15 = Precipitation seasonality	−0.520	.604	−1.642	.103	−2.522	.013
BIO19 = Precipitation of coldest quarter	0.832	.409	1.237	.221	1.738	.099

### 3.4 Divergence‐time estimation and historical biogeography

Result from the BEAST analysis assessment suggest that the genus *Theloderma* originated in the Middle Oligocene, ca. 27.77 Ma (95% highest posterior density, HPD: 23.81–31.74 Ma; Figure 3). Subsequently, its most recent common ancestor appeared in the Late Oligocene (ca. 25.57 Ma). Clade B and Clade C diverged in the Early Miocene (23.55 Ma; 95% HPD: 19.57–27.48 Ma; Figure S4).

The models with the “+J” counterpart fit the data significantly better than the models without the “+J” counterpart, suggesting that jump‐dispersal or founder‐event speciation plays an important role in the biogeography of *Theloderma*. Model DIVALIKE+J was selected as the best‐fit model to explain the speciation and dispersal history of *Theloderma*, and this method allows the inclusion of potential dispersal events in the analysis (Table 4; AICc model weight = 0.73). Under the best‐fit model DIVALIKE+J, at least 10 vicariance events and 12 dispersal events were inferred to form the current geographic pattern of species distribution in *Theloderma*. Most of the nodes were inferred as “speciation within areas,” which is defined as two descendants with the same range as the ancestor, that is, in situ diversification.

**TABLE 4:** Statistical comparison of six different models using BioGeoBEARS and the selection of the best‐fit model (DIVALIKE+J) for ancestral area reconstruction.

Model	LnL	Number of parameters	d	e	j	AICc	AICc model weight
DEC	−61.33	2	0.0057	1.00E‐12	0	127	0.0004
DEC+J	−53.74	3	0.0021	1.00E‐12	0.036	114.1	0.24
DIVALIKE	−58.92	2	0.007	2.00E‐09	0	122.1	0.0043
**DIVALIKE+J**	**−52.63**	**3**	**0.0025**	**1.00E‐12**	**0.031**	**111.9**	**0.73**
BAYAREALIKE	−78.79	2	0.0086	0.032	0	161.9	1.00E‐11
BAYAREALIKE+J	−55.95	3	0.0016	1.00E‐07	0.041	118.5	0.026

*Note*: Six models were compared using the corrected Akaike information criterion (AICc) weight method. The best model is highlighted using bold text.

Abbreviations: d, Dispersal rate per million years along branches; e, Extinction rate per million years along branches; j, Likelihood of founder‐event speciation at cladogenesis; LnL, Log likelihood.

**FIGURE 4:**
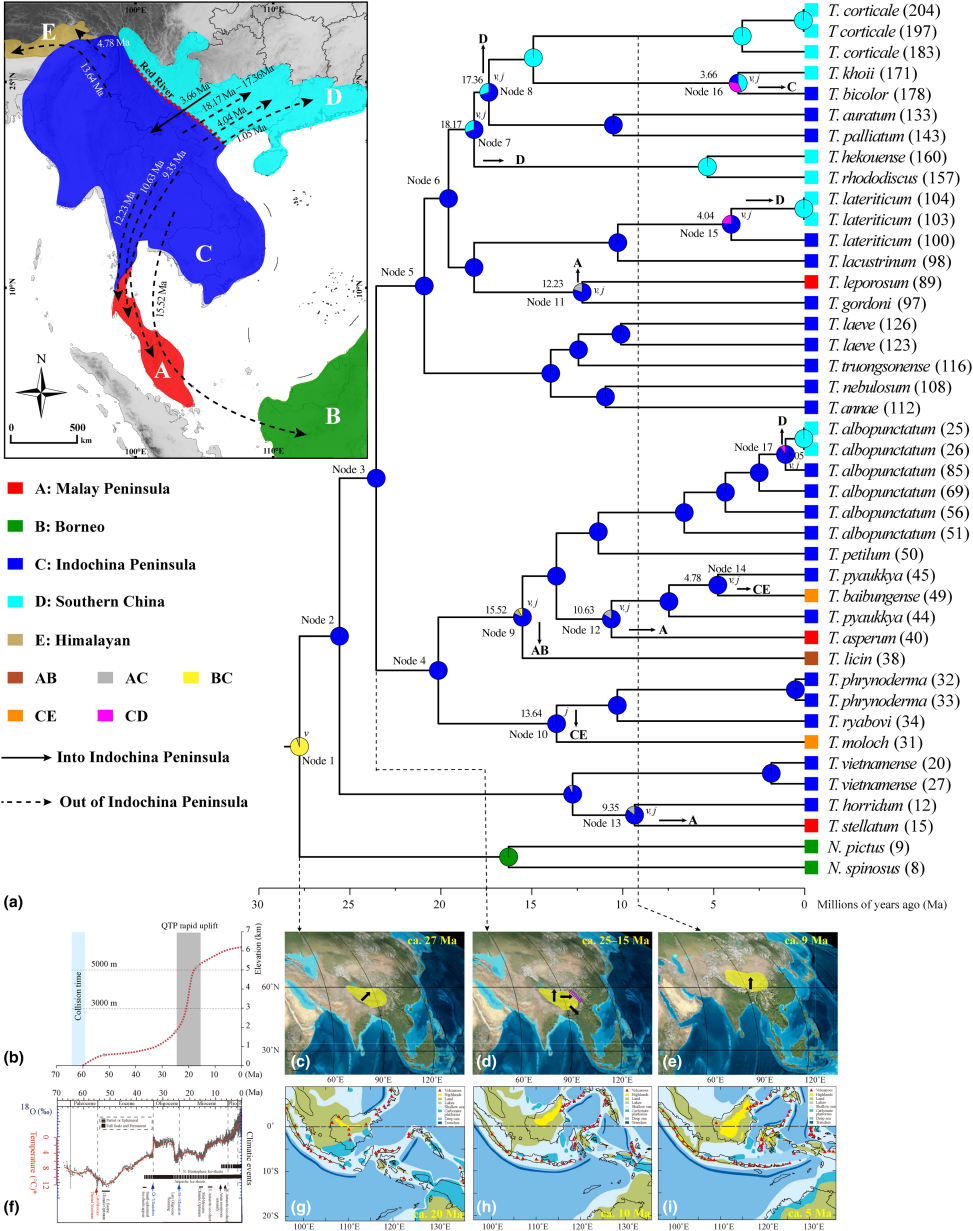
Biogeographic history of *Theloderma*. (a) Current distribution and reconstructed ancestral distribution of *Theloderma* based on best‐fit model (DIVALIKE+J) using BioGeoBEARS. The divergence time tree was estimated with BEAST based on combined mitochondrial and nuclear fragment data. Numbers at nodes represent mean node age. Node colors reflect biogeographic designations (for species at tips) and ancestral area reconstructions (for internal nodes). *v*, vicariance; *j*, jump‐dispersal or founder‐event speciation. (b) History of elevation rise in the Himalayas since the collision of the Indian and Eurasian plates in the Paleocene (modified from Ding et al., 2022). (c) General uplift of west China occurred at ca. 27 Ma due to the post‐collisional action of the Indian and Eurasian plates (modified from Che et al., 2010). (d) Lateral extrusion of the Indochina plate (purple dotted line) occurred simultaneously with the rapid uplift of the Himalayas, ca. 25–15 Ma (modified from Che et al., 2010). (e) The most recent uplift event began about 9 Ma. The maps in panels (c–e) are modified from those generated by TimeScale Creator version 8.0 (Zehady et al., 2020). (f) Main trends in global temperature change from the Paleocene to the Pleistocene (red line), and the global climate events that occurred during the same period. The sequence of climate events is represented by the global average delt ^18^O curve (left axis) derived from benthic foraminifera (modified from Zachos et al., 2001). (g–i) Three Cenozoic reconstructions of land and sea in the Indo‐Australian Archipelago of Southeast Asia.

Based on the DIVALIKE+J, the ancestors of *Theloderma* likely inhabited the Indochina Peninsula; this was consistent with the results of the second‐best‐fit model DEC+J (AICc weight = 0.24; Table 4; Figure S5). At least one vicariance event affected the most recent common ancestor of *Theloderma* and *Nyctixalus*, separating this taxon into two geographically distinct ancestral clades inhabiting the Indochina Peninsula and Borneo. Later, the Indochina Peninsula was the source (or species source) of colonization via dispersal jumps to the Malay Peninsula (ca. 12.23–9.35 Ma), Himalayas (ca. 4.78, 13.64 Ma), Southern China (ca. 18.17, 17.35, 1.05, and 4.04 Ma) and jumps dispersal to Borneo (ca. 15.52 Ma). Diversification within the Indochina Peninsula occurred in situ, that is, speciation within areas (Figure 4a). These colonization events via dispersal jumps occurred mainly during Miocene. Finally, the Indochina Peninsula was recolonized by taxa from Southern China in the Early Pliocene (ca. 3.66 Ma; Figure 4a).

### 3.5 Dynamics of historical diversification and ancestral characters

**FIGURE 5:**
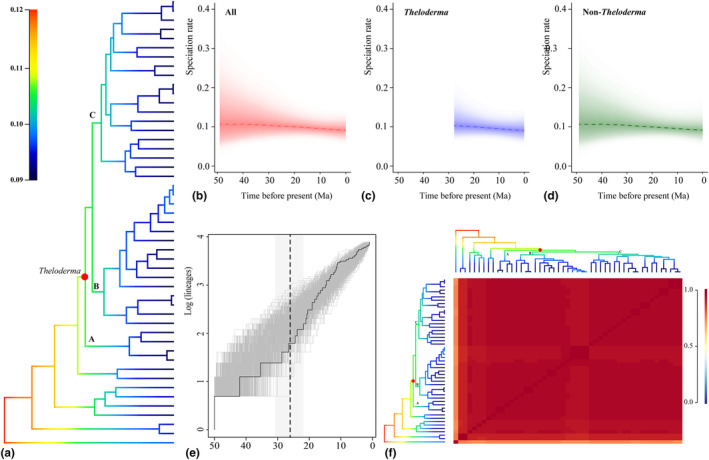
Diversification and speciation rates within the *Theloderma*. (a) Mean phylorate plot showing mean speciation rates. Cold colors denote slower rates, and warm colors denote faster rates. (b–d) Rate through time plots for speciation, with data from three different configurations used for BAMM analysis. Dashed lines denote means and lighter shadows denote 95% confidence intervals [CI]. (e) Lineage‐through‐time (LTT) plot of *Theloderma* showing the lineage accumulation over time in millions of years (Ma). (f) Macro‐evolutionary cohort matrix for diversification (speciation). The color‐range from cool (0.00 scaled value) to warm (1.00 scaled value) reflects the probability that two species share a common macro‐evolutionary rate regime.

BAMM identified a slight increase in speciation rate at the base of the *Theloderma* clade (Figure 5a). Based on BAMM analyses performed on three datasets, that is, all species, *Theloderma*, and non‐*Theloderma*, the plotted RTT shows a gradual slow decline in speciation rates since the Late Oligocene (ca. 25 Ma) (Figure 5b–d). The LTT plot showed that lineage accumulation accelerated during the Early Miocene (ca. 20–12 Ma), was near‐constant during the Late Miocene (ca. 12–7 Ma), accelerated slightly toward the end of the Late Miocene (ca. 6 Ma), and gradually slowed down thereafter (Figure 5e). The macro‐evolutionary cohort matrix revealed that most species in the three major *Theloderma* clades had a very high probability of sharing the same rate dynamics (Figure 5f).

**FIGURE 6:**
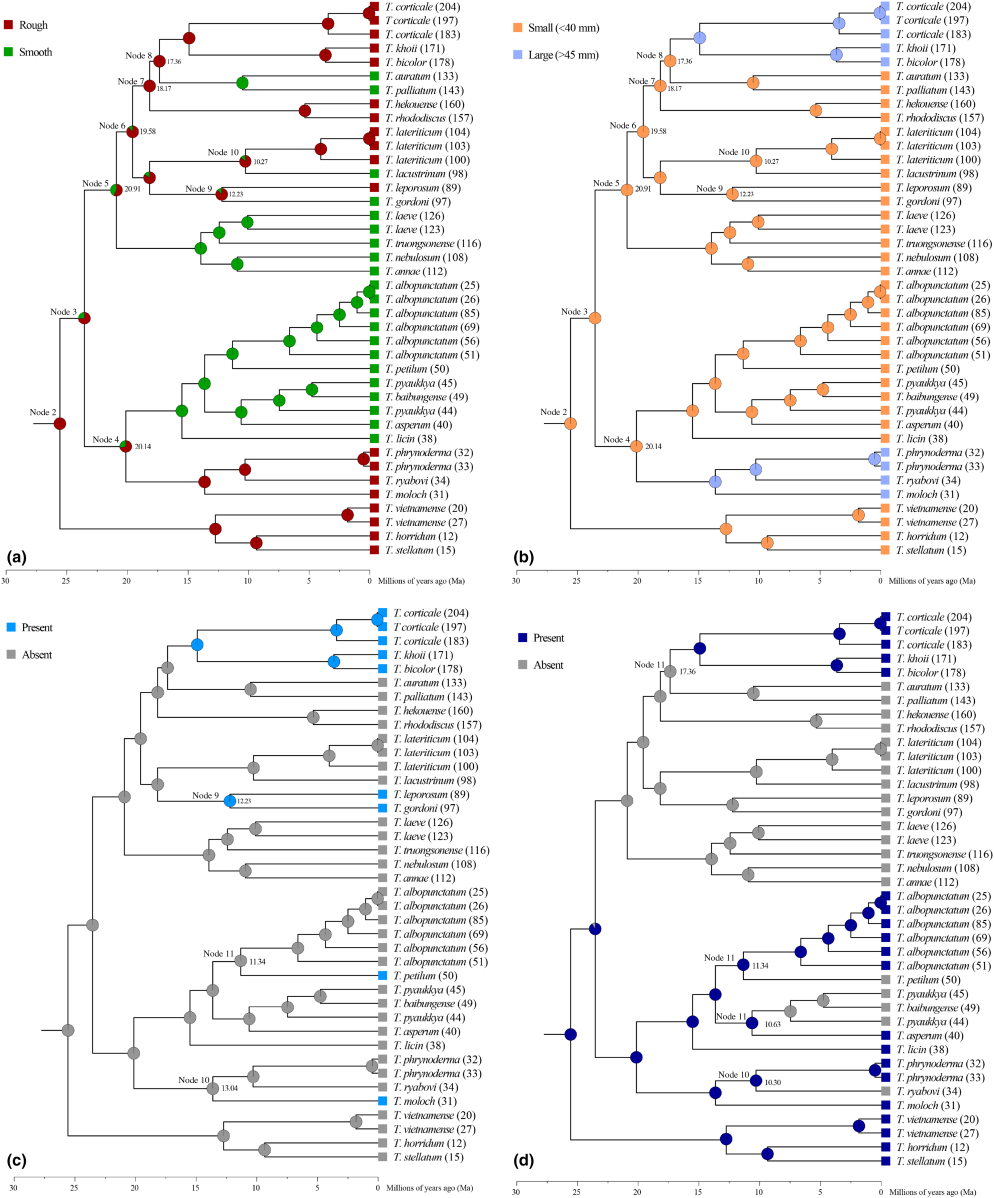
Ancestral characteristics reconstructed using BI, as implemented in RASP version 4.0, with the four key characteristic state reconstructions mapped to the time‐calibrated phylogeny of *Theloderma*. Tip circles denote characteristics used for ancestral state reconstruction, and pie charts at nodes denote posterior probabilities of characteristic states. (a) skin; (b) body size; (c) vomerine teeth; (d) hand webbing.

Ancestral characters reconstruction yielded almost identical results for the four characters (Figure 6; Figure S6). These results suggest that the ancestors of *Theloderma* may have exhibited rough dorsal skin (Figure 6a), a small body size (<40 mm) (Figure 6b), the absence of vomerine teeth (Figure 6c), and the presence of hand webbing (Figure 6d) for the four morphological characters selected for this study. These four characters have undergone multiple independent and near‐simultaneous innovation events, including four changes from rough to smooth dorsal skin, two changes from small to large body size, four gains of vomerine teeth, and at least three losses and one independent development of hand webbing. All these events occurred in the Early to Middle Miocene (ca. 20.91–10.30 Ma) and originated from ancestors inhabiting the Indochina Peninsula (Figures 5 and 6).

## 4 DISCUSSION

### 4.1 Molecular data and species limitations

In this study, we used a total of 27 species for phylogenetic analysis, representing 93.1% of the 29 known species, with only *T. nagalandense* and *T. pseudohorridum* not being included. *T. pseudohorridum* (Kurniawan et al., 2023) is a recently described species from Java, with rough dorsal skin, small body size, absence of vomerine teeth, and hand webbing; genetically, it is at the base of *Theloderma*, close to *T. horridum*, and therefore cannot influences our results on phylogeny, ancestral character reconstruction, and biogeography. *T. nagalandense* is probably morphologically close to *T. corticale*, having rough dorsal skin, a large body size (52.82 mm in snout‐vent length), vomerine teeth present, and hand webbing present (Orlov et al., 2006). Based on these morphological characters, we hypothesize that *T. nagalandense* is genetically close to *T. corticale*, and future studies containing this species will support our conclusions.

To be consistent with previous molecular markers used in phylogenetic analyses (Ellepola et al., 2022; Li et al., 2013; Poyarkov et al., 2015) and the publication of new species (Du et al., 2022; Ninh et al., 2022), we continued to use the same mitochondrial (12S, 16S, and COI) and nuclear genes (BNDF, RHOD, TYR, RAG1, and SIA) to obtain sufficient molecular samples at the species level. Although a matrix of 5926 bp was generated after alignment (Dataset 3), some nodes were still not fully resolved, especially within Clade C (Figure 3). This may be related to the lack of nuclear gene fragments in several species within Clade C (Table S1), and the addition of nuclear gene fragments may help to resolve this issue. The short sequences are a limitation of this study relative to several recent large‐scale studies (Chan et al., 2020; Chen et al., 2020; Ellepola et al., 2022). Despite the shortcomings in species numbers and molecular markers, our study has broadened our understanding and provided key insights into *Theloderma* in terms of diversity, phylogeny, biogeography, and diversification patterns, and thus has contributed to a better understanding of the evolutionary processes that generated the extant diversity.

### 4.2 Systematics and diversity based on molecules

Based on previous phylogenies, *Theloderma* was composed of subgenera *Stelladerma* and *Theloderma*, and seven species groups: *T. horridum* group, *T. moloch* group, *T. asperum* group, *T. leporosum* group, *T. lateriticum* group, *T. laeve* group, and *T. corticale* group (Kurniawan et al., 2023; Poyarkov et al., 2018) (Table S10). Using combined mitochondrial and nuclear gene data, we recovered three well‐supported clades within *Theloderma* that were similar to those delineated in previous revisions (Figure 3): Clade A and Clades B + C corresponded to the subgenera *Stelladerma* and *Theloderma*. Our phylogenetic tree strongly supported the subgenera and species groups previously accepted in the genus *Theloderma* (Table S8).

Compared to other species delimitation methods (e.g., GMYC and bPTP), the mPTP method has been shown to be the most stable, especially when sampling is highly heterogeneous or when the effective population size of species varies widely (Blair & Bryson, 2017; Kapli et al., 2017). Thus, the species delimitation results from mPTP may be more accurate than those from other methods. However, four method of species delimitation suggests that species diversity within the genus *Theloderma* may be underestimated, although there are differences in species delimitation schemes. For example: (1) four method all consistently identified cryptic species in both *T. laeve* and *T. truongsonense*, from central and Southern Vietnam that were shown to be deeply divergent lineages (genetic distance >3%) (Fouquet et al., 2007); (2) ASAP and ABGD merged *T. bicolor* and *T. khoii* as a single species, while bPTP and mPTP supported them as being separate species; and (3) ASAP and ABGD suggested that *T. albopunctatum* is a single species, with bPTP and mPTP suggesting its division into multiple species. Recent taxonomic studies are also supported by the bPTP and mPTP, namely the description of *T. khoii* and *T. hekouense* from the geographical populations of *T. bicolor* and *T. rhododiscus* (Du et al., 2022; Ninh et al., 2022). *T. bicolor* and *T. khoii* were also recovered as different evolutionary lineages (genetic distance >5%) and have significant morphological differentiation (Ninh et al., 2022), but ASAP and ABGD merged these into a single species; this was similar to a previous study of *T. albopunctatum* (Chan et al., 2018). These results suggest that species diversity in the *Theloderma* is underestimated, and that detailed morphological examination, as well as information on geographic distribution, is needed to clarify the taxonomic status of each population.

### 4.3 Dispersal out of the Indochina Peninsula and historical diversification

In this study, the estimated divergence times for the five nodes based on secondary calibration were close to those of previous studies (Chen et al., 2020; Li et al., 2013), with differences of ~1.1 to 5.8 Ma. The “+J” model implemented in BioGeoBEARS (Matzke, 2014) has been criticized by Ree and Sanmartín (2018), with the former highlighting the importance of founder‐event speciation and the latter arguing that the use of “+J” parameter may bias results away from the “true” evolutionary process. Based on model comparisons and previous studies (Chen et al., 2020; Yuan et al., 2021) (Table 4), the best and second‐best models selected from the six models supported the addition of the “+J” parameter.

Orogeny may promote the diversification and outward dispersal of *Theloderma* in the Indochina Peninsula. Our divergence times and elevation change rates confirm this. *Theloderma* originated in the Middle Oligocene (ca. 27.77 Ma), and early splits of the three clades occurred during the Late Oligocene to Early Miocene (ca. 29–19 Ma) (Figure 4a). Subsequently, outward dispersal events from Indochina Peninsula to the Malay Peninsula, Southern China, the Himalayas, and Malay Peninsula‐Borneo, as well as a Pliocene backward migration from southern China to the south‐central Peninsula, occurred during the Miocene (Figure 4a). This origin and divergence are closely related to the early rapid uplift of the QTP (ca. 27–23 Ma) (Che et al., 2010; Ding et al., 2022) and the rapid lateral extrusion of Indochina (ca. 25–22 Ma) (Lacassin et al., 1997; Leloup et al., 2001; Tapponnier et al., 1990). Indeed, the origins or early diversifications of several animal and plant taxa have been identified near the Oligocene/Miocene boundary (Che et al., 2010; Luo & Li, 2022), suggesting the simultaneous occurrence of the early rapid uplift of the QTP and lateral extrusion of Indochina (Luo & Li, 2022). The co‐occurrence of these two geological events led to an increase in elevation that may have led to the early divergence and subsequent outward dispersal of the three major clades of *Theloderma*, as has previously been proposed in *Leptobrachella* (Chen et al., 2018) and *Amolops* (Wu et al., 2020). If the hypothesis that the diversification of *Theloderma* is driven by elevational uplift holds, then our results imply that the average elevation of the Indochina Peninsula may have reached its current height in the Middle Miocene, similar to that of the Hengduan Mountains (Ding et al., 2020).

Ailao Shan‐Red River Shear Zone (ASRSZ) is a weak barrier that formed from the Middle Oligocene to the Early Miocene (ca. 27–17 Ma) (Harrison et al., 1992; Leloup et al., 2001; Tang et al., 2013; Tapponnier et al., 1990). We observe the first dispersal of *Theloderma* from the Indochina Peninsula into Southern China, close to the time of the rapid uplift of the Himalayas (~20 Ma, Ding et al., 2022) and the formation of the ASRSZ. This suggests that, unlike *Polypedates* (Yuan et al., 2021) and *Tylototriton* (Wang et al., 2018), the ASRSZ failed to act as a barrier to the northward dispersal of *Theloderma*, as evidenced by dispersal events during the Miocene to Pleistocene (Figure 4, nodes 16 and 17). The formation and uplift of the Annamite Mountains at ca. 400–300 Ma (Fontaine, 1978), well before the formation and diversification of *Theloderma*, is similar to the ASRSZ, that is, big mountains but small barriers. Such a geographic barrier effect has been echoed in previous biogeographic studies of the region (Chen et al., 2018; Wu et al., 2020; Yuan et al., 2021). On the other hand, the global temperature cooling (Westerhold et al., 2020) during the Miocene resulting in sea level decline (Haq et al., 1987; Miller et al., 2005) may have opened a passageway for the multiple dispersal of *Theloderma* from the Indochina Peninsula to the Malay Peninsula and Borneo.

Diversification of *Theloderma* is closely related to palaeoclimatic shifts. *Theloderma* originated roughly in the Oligocene, a period most clearly marked by a cool, dry climate (Westerhold et al., 2020; Zachos et al., 2001). Early diversification of *Theloderma* was driven by potential geographic separation that may have resulted from the continued contraction and fragmentation of wet forests used as suitable habitat during this period (as well as elevational differentiation due to orogenic movements as described above) (Bain & Hurley, 2011; Ellepola et al., 2022; Li, Xiang, Jabbour, et al., 2022; Xu et al., 2020). Later, most nodes (nodes 4–11, Figure 4) diverged in the Miocene, which is associated with an intensification of the monsoon climate triggered by the sharp and rapid uplift of the QTP at 20 Ma and an Early Miocene climate that became warmer and wetter (Clift et al., 2008; Retallack et al., 2018). The shift to a suitable habitat climate may have largely contributed to the accelerated diversification and lineage accumulation of *Theloderma*. However, the dramatic global climate decline in the Late Miocene (~14 Ma; Westerhold et al., 2020; Zachos et al., 2001) and subsequent persistent aridity and cold may have slowed the accumulation of *Theloderma* lineages (Figure 5e).

In addition, we observed key morphological innovations similar to previous findings (Nguyen et al., 2015), such as dorsal skin from rough to smooth, body size from small to large, vomerine teeth from absent to present, and hand webbing from absent to present, that coincided with the timing of outward dispersal (Figures 4 and 6). For this, we have made a preliminary explanation here. *Theloderma* originated in the Late Oligocene, and ancestral species may have had morphological characteristics such as rough dorsal skin, small body size, absence of vomerine teeth, and the presence of hand webbing. During the rapid warming of the Late Oligocene (Westerhold et al., 2020; Zachos et al., 2001), rough skin and a small body size may have evolved to adapt to the rapidly warming thermal environment, thereby reducing the evaporation process from the skin to better retain moisture. *Theloderma* evolved during the Early Miocene into species with smooth skin, large body sizes, vomerine teeth, and no hand webbing. This may be related to the transitory climatic optimum of the Middle Miocene (Westerhold et al., 2020; Zachos et al., 2001), where smooth skin facilitated the acquisition of moisture from the air, and the presence of large bodies and teeth facilitated dispersal and increased feeding efficiency. The evolution of hand webbing from presence to absence suggests a possible change in locomotion from gliding to jumping and/or climbing, as webbing may have been a necessary adaptation for gliding (Emerson & Koehl, 1990; Wu et al., 2022), which may have expanded the habitat space of *Theloderma* from the forest canopy to the understory. The absence of hand webbing may also suggest that forest vegetation turnover may have occurred in the early Miocene in the Indochina Peninsula and Southern China, as webless forms may have been more adapted to lowland life “without evergreen broadleaf forest”, corresponding to the timing of the peak of cave life and vegetation development in the karst region (Li, Xiang, Jabbour, et al., 2022; Li, Xiang, Zhang, et al., 2022). These morphological innovations may have helped *Theloderma* species to offset or adapt to unfavorable paleoclimates, such as nearly constant diversification rates and low speciation rates (Figure 4), and further contributed to the outward dispersal and diversification of *Theloderma*, which had long been living in “cold‐wet refugia” (Ellepola et al., 2022).

## 5 CONCLUSIONS

We investigated the phylogeny, species boundaries, and biogeography of the Asian warty treefrog, genus *Theloderma*, based on genetically extensive collections of molecular, environmental, and morphological data. Our results support the classification of *Theloderma* into two subgenera and seven species groups, and its species diversity is underestimated. Spatio‐temporal analyses suggest that *Theloderma* originated in the Oligocene and that early diversification driven by elevational uplift and habitat isolation occurred in the Indochina Peninsula from the Late Oligocene to the Early Miocene. During the evolutionary process of outward dispersal and in situ speciation from the Indochina Peninsula, multiple evolutionary innovations in dorsal skin, body size vomerine teeth, and hand webbing have occurred. For *Theloderma*, a series of events during the Miocene, such as orogeny, the intensification of the Asian monsoon and the evolution of palaeoclimatic warming and cooling, as well as key morphological innovations, have contributed to and maintained the current pattern of diversification.

## AUTHOR CONTRIBUTIONS

**Tao Luo:** Software (equal); writing – original draft (lead); writing – review and editing (lead). **Xin‐Rui Zhao:** Investigation (equal); visualization (supporting); writing – original draft (equal). **Chang‐Ting Lan:** Software (equal); visualization (equal); writing – review and editing (equal). **Wei Li:** Investigation (equal); resources (equal); visualization (equal). **Huai‐Qing Deng:** Resources (equal); writing – original draft (equal). **Ning Xiao:** Resources (supporting); writing – review and editing (equal). **Jiang Zhou:** Project administration (equal); writing – original draft (equal); writing – review and editing (equal).

## CONFLICT OF INTEREST STATEMENT

The authors declare no conflicts of interest.

### OPEN RESEARCH BADGES

This article has earned an Open Data badge for making publicly available the digitally‐shareable data necessary to reproduce the reported results. The data is available at https://github.com/istaude/neophytes‐hybrids.

## Supporting information


Data S1

## Data Availability

The mitochondrial and nuclear gene data reported in this paper are available in the nucleotide database of the National Center for Biotechnology Information, USA, under accession numbers OP537823, OP537824, OP537793, OP537794, OP537813, OP537814, OP531848–OP531853, and OP561732–OP561761. All sequence datasets and tree files for phylogenetic and biogeographic analyses were submitted for storage in Dryad (https://doi.org/10.5061/dryad.79cnp5j1j). All supplementary data needed to evaluate the conclusions of the paper are in the paper and/or supplementary materials. Additional data relevant to this paper are available from the authors upon request. We have deposited some data in Dryad with the DOI number. DOI: 10.5061/dryad.02v6wwq97.

## References

[ece310829-bib-0001] Bain, R. H. , & Hurley, M. M. (2011). A biogeographic synthesis of the amphibians and reptiles of Indochina. Bulletin of the American Museum of Natural History, 2011(360), 1–138. 10.1206/360.1

[ece310829-bib-0002] Blair, C. , & Bryson, R. W., Jr. (2017). Cryptic diversity and discordance in single‐locus species delimitation methods within horned lizards (Phrynosomatidae: *Phrynosoma*). Molecular Ecology Resources, 17(6), 1168–1182. 10.1111/1755-0998.12658 28161911

[ece310829-bib-0003] Chan, K. O. , Grismer, L. L. , & Brown, R. M. (2018). Comprehensive multi‐locus phylogeny of Old World tree frogs (Anura: Rhacophoridae) reveals taxonomic uncertainties and potential cases of over‐ and underestimation of species diversity. Molecular Phylogenetics and Evolution, 127, 1010–1019. 10.1016/j.ympev.2018.07.005 30030179

[ece310829-bib-0004] Chan, K. O. , Hutter, C. R. , Wood, P. L., Jr. , Grismer, L. L. , & Brown, R. M. (2020). Target‐capture phylogenomics provide insights on gene and species tree discordances in Old World treefrogs (Anura: Rhacophoridae). Proceedings of the Royal Society B: Biological Sciences, 287(1940), 20202102. 10.1098/rspb.2020.2102 PMC7739936 33290680

[ece310829-bib-0005] Che, J. , Zhou, W.‐W. , Hu, J.‐S. , Yan, F. , Papenfuss, T. J. , Wake, D. B. , & Zhang, Y.‐P. (2010). Spiny frogs (Paini) illuminate the history of the Himalayan region and Southeast Asia. Proceedings of the National Academy of Sciences of the United States of America, 107(31), 13765–13770. 10.1073/pnas.1008415107 20643945 PMC2922240

[ece310829-bib-0006] Chen, J.‐M. , Poyarkov, N. A., Jr. , Suwannapoom, C. , Lathrop, A. , Wu, Y.‐H. , Zhou, W.‐W. , Yuan, Z. Y. , Jin, J. Q. , Chen, H. M. , Liu, H. Q. , Nguyen, T. Q. , Nguyen, S. N. , Duong, T. V. , Eto, K. , Nishikawa, K. , Matsui, M. , Orlov, N. L. , Stuart, B. L. , Brown, R. M. , … Che, J. (2018). Large‐scale phylogenetic analyses provide insights into unrecognized diversity and historical biogeography of Asian leaf‐litter frogs, genus *Leptolalax* (Anura: Megophryidae). Molecular Phylogenetics and Evolution, 124, 162–171. 10.1016/j.ympev.2018.02.020 29530499

[ece310829-bib-0007] Chen, J.‐M. , Prendini, E. , Wu, Y.‐H. , Zhang, B.‐L. , Suwannapoom, C. , Chen, H.‐M. , Jin, J. Q. , Lemmon, E. M. , Lemmon, A. R. , Stuart, B. L. , Raxworthy, C. J. , Murphy, R. W. , Yuan, Z. Y. , & Che, J. (2020). An integrative phylogenomic approach illuminates the evolutionary history of Old World tree frogs (Anura: Rhacophoridae). Molecular Phylogenetics and Evolution, 145, 106724. 10.1016/j.ympev.2019.106724 31881327

[ece310829-bib-0008] Clift, P. D. , Hodges, K. V. , Heslop, D. , Hannigan, R. , Van Long, H. , & Calves, G. (2008). Correlation of Himalayan exhumation rates and Asian monsoon intensity. Nature Geoscience, 1(12), 875–880. 10.1038/ngeo351

[ece310829-bib-0009] Ding, L. , Kapp, P. , Cai, F. , Garzione, C. N. , Xiong, Z. , Wang, H. , & Wang, C. (2022). Timing and mechanisms of Tibetan Plateau uplift. Nature Reviews Earth and Environment, 10(3), 652–667. 10.1038/s43017-022-00318-4

[ece310829-bib-0010] Ding, W.‐N. , Ree, R. H. , Spicer, R. A. , & Xing, Y.‐W. (2020). Ancient orogenic and monsoon‐driven assembly of the world's richest temperate alpine flora. Science, 369(6503), 578–581. 10.1126/science.abb4484 32732426

[ece310829-bib-0011] Dormann, C. F. , Elith, J. , Bacher, S. , Buchmann, C. , Carl, G. , Carré, G. , García Marquéz, J. R. , Gruber, B. , Lafourcade, B. , Leitão, P. J. , Münkemüller, T. , McClean, C. , Osborne, P. E. , Reineking, B. , Schröder, B. , Skidmore, A. K. , Zurell, D. , & Lautenbach, S. (2013). Collinearity: A review of methods to deal with it and a simulation study evaluating their performance. Ecography, 36(1), 27–46. 10.1111/j.1600-0587.2012.07348.x

[ece310829-bib-0012] Drummond, A. J. , Suchard, M. A. , Xie, D. , & Rambaut, A. (2012). Bayesian phylogenetics with BEAUti and the BEAST 1.7. Molecular Biology and Evolution, 29(8), 1969–1973. 10.1093/molbev/mss075 22367748 PMC3408070

[ece310829-bib-0013] Du, L.‐Y. , Wang, J. , Liu, S. , & Yu, G.‐H. (2022). A new cryptic species in the *Theloderma rhododiscus* complex (Anura, Rhacophoridae) from China–Vietnam border regions. ZooKeys, 1099, 123–138. 10.3897/zookeys.1099.80390 36761445 PMC9848745

[ece310829-bib-0014] Ellepola, G. , Pie, M. R. , Pethiyagoda, R. , Hanken, J. , & Meegaskumbura, M. (2022). The role of climate and islands in species diversification and reproductive‐mode evolution of Old World tree frogs. Communications Biology, 5(1), 347. 10.1038/s42003-022-03292-1 35411020 PMC9001633

[ece310829-bib-0015] Emerson, S. B. , & Koehl, M. A. R. (1990). The interaction of behavioral and morphological change in the evolution of a novel locomotor type:“flying” frogs. Evolution, 44(8), 1931–1946. 28564439 10.1111/j.1558-5646.1990.tb04300.x

[ece310829-bib-0016] Fontaine, H. (1978). Review of the geology and mineral resources ogf Kampuchea, Laos and Vietnam. In P. Nutalaya (Ed.), Proceedings of the Third Regional Conference on Geology and Mineral Resources of Southeast Asia, Bangkok (pp. 541–603). Asian Institute of Technology.

[ece310829-bib-0017] Fouquet, A. , Gilles, A. , Vences, M. , Marty, C. , Blanc, M. , & Gemmell, N. J. (2007). Underestimation of species richness in Neotropical frogs revealed by mtDNA analyses. PLoS One, 2(10), e1109. 10.1371/journal.pone.0001109 17971872 PMC2040503

[ece310829-bib-0018] Frost, D. R. (2023). Amphibian species of the world: An online reference . Version 6.2. Retrieved from http://research.amnh.org/herpetology/amphibia/index.html

[ece310829-bib-0019] Haq, B. U. , Hardenbol, J. , & Vail, P. R. (1987). Chronology of fluctuating sea levels since the triassic. Science, 235(4793), 1156–1167. 10.1126/science.235.4793.1156 17818978

[ece310829-bib-0021] Harrison, T. M. , Wenji, C. , Leloup, P. , Ryerson, F. , & Tapponnier, P. (1992). An early Miocene transition in deformation regime within the Red River fault zone, Yunnan, and its significance for Indo‐Asian tectonics. Journal of Geophysical Research: Solid Earth, 97(B5), 7159–7182. 10.1029/92JB00109

[ece310829-bib-0022] Hijmans, R. J. , Cameron, S. E. , Parra, J. L. , Jones, P. G. , & Jarvis, A. (2005). Very high resolution interpolated climate surfaces for global land areas. International Journal of Climatology: A Journal of the Royal Meteorological Society, 25(15), 1965–1978. 10.1002/joc.1276

[ece310829-bib-0023] Hijmans, R. J. , Van Etten, J. , Cheng, J. , Mattiuzzi, M. , Sumner, M. , Greenberg, J. A. , Lamigueiro, O. P. , Bevan, A. , Racine, E. B. , Shortridge, A. , & Hijmans, M. R. J. (2015). Package ‘raster’ . R Package, 734, 473.

[ece310829-bib-0024] Hoang, D. T. , Chernomor, O. , Von Haeseler, A. , Minh, B. Q. , & Vinh, L. S. (2018). UFBoot2: Improving the ultrafast bootstrap approximation. Molecular Biology and Evolution, 35(2), 518–522. 10.1093/molbev/msx281 29077904 PMC5850222

[ece310829-bib-0025] Kapli, P. , Lutteropp, S. , Zhang, J. , Kobert, K. , Pavlidis, P. , Stamatakis, A. , & Flouri, T. (2017). Multi‐rate Poisson tree processes for single‐locus species delimitation under maximum likelihood and Markov chain Monte Carlo. Bioinformatics, 33(11), 1630–1638. 10.1093/bioinformatics/btx025 28108445 PMC5447239

[ece310829-bib-0026] Kumar, S. , Stecher, G. , & Tamura, K. (2016). MEGA7: Molecular evolutionary genetics analysis version 7.0 for bigger datasets. Molecular Biology and Evolution, 33(7), 1870–1874. 10.1093/molbev/msw054 27004904 PMC8210823

[ece310829-bib-0027] Kurniawan, N. , Septiadi, L. , Kadafi, A. M. , Fathoni, M. , Prabasari, K. , & Thammachoti, P. (2023). A new species of *Theloderma* Tschudi, 1838 (Amphibia: Rhacoporidae) from Central Java Allied to *T. horridum* (Boulenger, 1903). Asian Herpetological Research, 14(1), 1–23. 10.16373/j.cnki.ahr.220033

[ece310829-bib-0028] Lacassin, R. , Maluski, H. , Leloup, P. H. , Tapponnier, P. , Hinthong, C. , Siribhakdi, K. , Chuaviroj, S. , & Charoenravat, A. (1997). Tertiary diachronic extrusion and deformation of western Indochina: Structural and 40Ar/39Ar evidence from NW Thailand. Journal of Geophysical Research: Solid Earth, 102(B5), 10013–10037. 10.1029/96JB03831

[ece310829-bib-0029] Landis, M. J. , Matzke, N. J. , Moore, B. R. , & Huelsenbeck, J. P. (2013). Bayesian analysis of biogeography when the number of areas is large. Systematic Biology, 62(6), 789–804. 10.1093/sysbio/syt040 23736102 PMC4064008

[ece310829-bib-0030] Lanfear, R. , Frandsen, P. B. , Wright, A. M. , Senfeld, T. , & Calcott, B. (2016). PartitionFinder 2: New methods for selecting partitioned models of evolution for molecular and morphological phylogenetic analyses. Molecular Biology and Evolution, 34(3), 772–773. 10.1093/molbev/msw260 28013191

[ece310829-bib-0031] Leloup, P. H. , Arnaud, N. , Lacassin, R. , Kienast, J. , Harrison, T. , Trong, T. P. , Replumaz, A. , & Tapponnier, P. (2001). New constraints on the structure, thermochronology, and timing of the Ailao Shan‐Red River shear zone, SE Asia. Journal of Geophysical Research: Solid Earth, 106(B4), 6683–6732. 10.1029/2000jb900322

[ece310829-bib-0032] Lewis, P. O. (2001). A likelihood approach to estimating phylogeny from discrete morphological character data. Systematic Biology, 50(6), 913–925. 10.1080/106351501753462876 12116640

[ece310829-bib-0033] Li, J. , Li, Y. , Klaus, S. , Rao, D. , Hillis, D. M. , & Zhang, Y. (2013). Diversification of rhacophorid frogs provides evidence for accelerated faunal exchange between India and Eurasia during the Oligocene. Proceedings of the National Academy of Sciences of the United States of America, 110(9), 3441–3446. 10.1073/pnas.1300881110 23401521 PMC3587228

[ece310829-bib-0034] Li, X.‐Q. , Xiang, X.‐G. , Jabbour, F. , Hagen, O. , Ortiz, R. C. , Soltis, P. S. , Soltis, D. E. , & Wang, W. (2022). Biotic colonization of subtropical East Asian caves through time. Proceedings of the National Academy of Sciences of the United States of America, 119(34), e2207199119. 10.1073/pnas.2207199119 35969742 PMC9407641

[ece310829-bib-0035] Li, X.‐Q. , Xiang, X.‐G. , Zhang, Q. , Jabbour, F. , Ortiz, R. C. , Erst, A. S. , Li, Z. Y. , & Wang, W. (2022). Immigration dynamics of tropical and subtropical Southeast Asian limestone karst floras. Proceedings of the Royal Society B: Biological Sciences, 289(1966), 20211308. 10.1098/rspb.2021.1308 PMC8727148 34982948

[ece310829-bib-0036] Luo, Y. , & Li, S. (2022). The stepwise Indian–Eurasian collision and uplift of the Himalayan‐Tibetan plateau drove the diversification of high‐elevation *Scytodes* spiders. Cladistics, 38, 582–594. 10.1111/cla.12512 35802675

[ece310829-bib-0037] Maddison, W. , & Maddison, D. (2019). Mesquite: A modular system fo evolutionary analysis . 3.70. Retrieved from http://mesquiteproject.org/

[ece310829-bib-0038] Matzke, N. J. (2013a). *BioGeoBEARS: BioGeography with Bayesian (and likelihood) evolutionary analysis in R scripts* (PhD thesis). University of Californi, Berkley.

[ece310829-bib-0039] Matzke, N. J. (2013b). Probabilistic historical biogeography: New models for founder‐event speciation, imperfect detection, and fossils allow improved accuracy and model‐testing. Frontiers of Biogeography, 5, 242–248.

[ece310829-bib-0040] Matzke, N. J. (2014). Model selection in historical biogeography reveals that founder‐event speciation is a crucial process in Island clades. Systematic Biology, 63(6), 951–970. 10.1093/sysbio/syu056 25123369

[ece310829-bib-0041] Miller, K. G. , Kominz, M. A. , Browning, J. V. , Wright, J. D. , Mountain, G. S. , Katz, M. E. , Sugarman, P. J. , Cramer, B. S. , Christie‐Blick, N. , & Pekar, S. F. (2005). The phanerozoic record of global sea‐level change. Science, 310(5752), 1293–1298. 10.1126/science.1116412 16311326

[ece310829-bib-0042] Nguyen, L.‐T. , Schmidt, H. A. , von Haeseler, A. , & Minh, B. Q. (2014). IQ‐TREE: A fast and effective stochastic algorithm for estimating maximum‐likelihood phylogenies. Molecular Biology and Evolution, 32(1), 268–274. 10.1093/molbev/msu300 25371430 PMC4271533

[ece310829-bib-0043] Nguyen, T. T. , Matsui, M. , & Eto, K. (2015). Mitochondrial phylogeny of an Asian tree frog genus *Theloderma* (Anura: Rhacophoridae). Molecular Phylogenetics and Evolution, 85, 59–67. 10.1016/j.ympev.2015.02.003 25683047

[ece310829-bib-0044] Ninh, H. T. , Nguyen, T. T. , Nguyen, H. Q. , Van Hoang, N. , Siliyavong, S. , Van Nguyen, T. , Le, D. T. , Le, Q. K. , & Ziegler, T. (2022). A new species of mossy frog (Anura: Rhacophoridae) from Northeastern Vietnam. European Journal of Taxonomy, 794, 72–90. 10.5852/ejt.2022.794.1655

[ece310829-bib-0045] Orlov, N. L. , Dutta, S. , Ghate, H. , & Kent, Y. (2006). New species of *Theloderma* from Kon Tum Province (Vietnam) and Nagaland State (India) (Anura: Rhacophoridae). Russian Journal of Herpetology, 13(2), 135–154.

[ece310829-bib-0046] Poyarkov, N. A., Jr. , Orlov, N. L. , Moiseeva, A. V. , Pawangkhanant, P. , Ruangsuwan, T. , Vassilieva, A. B. , Galoyan, E. A. , Nguyen, T. T. , & Gogoleva, S. S. (2015). Sorting out moss frogs: mtDNA data on taxonomic diversity and phylogenetic relationships of the Indochinese species of the genus *Theloderma* (Anura, Rhacophoridae). Russian Journal of Herpetology, 22(4), 241–280.

[ece310829-bib-0047] Poyarkov, N. A., Jr. , Kropachev, I. I. , Gogoleva, S. S. , & Orlov, N. L. (2018). A new species of the genus *Theloderma* Tschudi, 1838 (Amphibia: Anura: Rhacophoridae) from Tay Nguyen Plateau, central Vietnam. Zoological Research, 39(3), 158–184. 10.24272/j.issn.2095-8137.2018.018 29683110 PMC5968860

[ece310829-bib-0048] Puillandre, N. , Brouillet, S. , & Achaz, G. (2021). ASAP: Assemble species by automatic partitioning. Molecular Ecology Resources, 21(2), 609–620. 10.1111/1755-0998.13281 33058550

[ece310829-bib-0049] Puillandre, N. , Lambert, A. , Brouillet, S. , & Achaz, G. (2012). ABGD, automatic barcode gap discovery for primary species delimitation. Molecular Ecology, 21(8), 1864–1877. 10.1111/j.1365-294X.2011.05239.x 21883587

[ece310829-bib-0050] Rabosky, D. L. , Donnellan, S. C. , Grundler, M. , & Lovette, I. J. (2014). Analysis and visualization of complex macroevolutionary dynamics: An example from Australian scincid lizards. Systematic Biology, 63(4), 610–627. 10.1093/sysbio/syu025 24682412

[ece310829-bib-0051] Rabosky, D. L. , Grundler, M. , Anderson, C. , Title, P. , Shi, J. J. , Brown, J. W. , Huang, H. , & Larson, J. G. (2014). BAMM tools: An R package for the analysis of evolutionary dynamics on phylogenetic trees. Methods in Ecology and Evolution, 5(7), 701–707. 10.1111/2041-210X.12199

[ece310829-bib-0052] Rabosky, D. L. , Santini, F. , Eastman, J. , Smith, S. A. , Sidlauskas, B. , Chang, J. , & Alfaro, M. E. (2013). Rates of speciation and morphological evolution are correlated across the largest vertebrate radiation. Nature Communications, 4(1), 1958. 10.1038/ncomms2958 23739623

[ece310829-bib-0053] Rambaut, A. , Drummond, A. J. , Xie, D. , Baele, G. , & Suchard, M. A. (2018). Posterior summarization in Bayesian phylogenetics using tracer 1.7. Systematic Biology, 67(5), 901–904. 10.1093/sysbio/syy032 29718447 PMC6101584

[ece310829-bib-0054] Ree, R. H. , & Sanmartín, I. (2018). Conceptual and statistical problems with the DEC+J model of founder‐event speciation and its comparison with DEC via model selection. Journal of Biogeography, 45(4), 741–749. 10.1111/jbi.13173

[ece310829-bib-0055] Ree, R. H. , & Smith, S. A. (2008). Maximum likelihood inference of geographic range evolution by dispersal, local extinction, and cladogenesis. Systematic Biology, 57(1), 4–14. 10.1080/10635150701883881 18253896

[ece310829-bib-0056] Retallack, G. J. , Bajpai, S. , Liu, X. , Kapur, V. V. , & Pandey, S. K. (2018). Advent of strong South Asian monsoon by 20 million years ago. The Journal of Geology, 126(1), 1–24. 10.1086/694766

[ece310829-bib-0057] Ronquist, F. (1997). Dispersal‐vicariance analysis: A new approach to the quantification of historical biogeography. Systematic Biology, 46(1), 195–203. 10.1093/sysbio/46.1.195

[ece310829-bib-0058] Ronquist, F. , Teslenko, M. , van der Mark, P. , Ayres, D. L. , Darling, A. , Höhna, S. , Larget, B. , Liu, L. , Suchard, M. A. , & Huelsenbeck, J. P. (2012). MrBayes 3.2: Efficient Bayesian phylogenetic inference and model choice across a large model space. Systematic Biology, 61(3), 539–542. 10.1093/sysbio/sys029 22357727 PMC3329765

[ece310829-bib-0059] Tang, Y. , Liu, J. , Tran, M.‐D. , Song, Z. , Wu, W. , Zhang, Z. , Zhao, Z. , & Chen, W. (2013). Timing of left‐lateral shearing along the Ailao Shan‐Red River shear zone: Constraints from zircon U–Pb ages from granitic rocks in the shear zone along the Ailao Shan Range, Western Yunnan, China. International Journal of Earth Sciences, 102(3), 605–626. 10.1007/s00531-012-0831-y

[ece310829-bib-0060] Tapponnier, P. , Lacassin, R. , Leloup, P. H. , Schärer, U. , Dalai, Z. , Haiwei, W. , Xiaohan, L. , Shaocheng, J. , Lianshang, Z. , & Jiayou, Z. (1990). The Ailao Shan/Red River metamorphic belt: Tertiary left‐lateral shear between Indochina and South China. Nature, 343(6257), 431–437. 10.1038/343431a0

[ece310829-bib-0061] Wang, B. , Nishikawa, K. , Matsui, M. , Nguyen, T. Q. , Xie, F. , Li, C. , Khatiwada, J. R. , Zhang, B. , Gong, D. , Mo, Y. , Wei, G. , Chen, X. , Shen, Y. , Yang, D. , Xiong, R. , & Jiang, J. (2018). Phylogenetic surveys on the newt genus *Tylototriton* sensu lato (Salamandridae, Caudata) reveal cryptic diversity and novel diversification promoted by historical climatic shifts. PeerJ, 6, e4384. 10.7717/peerj.4384 29576937 PMC5853667

[ece310829-bib-0062] Warren, D. L. , Glor, R. E. , & Turelli, M. (2010). ENMTools: A toolbox for comparative studies of environmental niche models. Ecography, 33(3), 607–611. 10.1111/j.1600-0587.2009.06142.x

[ece310829-bib-0063] Westerhold, T. , Marwan, N. , Drury, A. J. , Liebrand, D. , Agnini, C. , Anagnostou, E. , Barnet, J. S. K. , Bohaty, S. M. , de Vleeschouwer, D. , Florindo, F. , Frederichs, T. , Hodell, D. A. , Holbourn, A. E. , Kroon, D. , Lauretano, V. , Littler, K. , Lourens, L. J. , Lyle, M. , Pälike, H. , … Zachos, J. C. (2020). An astronomically dated record of Earth's climate and its predictability over the last 66 million years. Science, 369(6509), 1383–1387. 10.1126/science.aba6853 32913105

[ece310829-bib-0064] Wu, F. , Fang, X. , Yang, Y. , Dupont‐Nivet, G. , Nie, J. , Fluteau, F. , Zhang, T. , & Han, W. (2022). Reorganization of Asian climate in relation to Tibetan Plateau uplift. Nature Reviews Earth and Environment, 3(10), 684–700. 10.1038/s43017-022-00331-7

[ece310829-bib-0065] Wu, Y.‐H. , Yan, F. , Stuart, B. L. , Prendini, E. , Suwannapoom, C. , Dahn, H. A. , Zhang, B. L. , Cai, H. X. , Xu, Y. B. , Jiang, K. , Chen, H. M. , Lemmon, A. R. , Lemmon, E. M. , Raxworthy, C. J. , Orlov, N. L. , Murphy, R. W. , & Che, J. (2020). A combined approach of mitochondrial DNA and anchored nuclear phylogenomics sheds light on unrecognized diversity, phylogeny, and historical biogeography of the torrent frogs, genus *Amolops* (Anura: Ranidae). Molecular Phylogenetics and Evolution, 148, 106789. 10.1016/j.ympev.2020.106789 32173414

[ece310829-bib-0066] Xu, W. , Dong, W.‐J. , Fu, T.‐T. , Gao, W. , Lu, C.‐Q. , Yan, F. , Wu, Y. H. , Jiang, K. , Jin, J. Q. , Chen, H. M. , Zhang, Y. P. , Hillis, D. M. , & Che, J. (2020). Herpetological phylogeographic analyses support a Miocene focal point of Himalayan uplift and biological diversification. National Science Review, 8(9), nwaa263. 10.1093/nsr/nwaa263 34691726 PMC8433089

[ece310829-bib-0067] Yu, Y. , Harris, A. J. , Blair, C. , & He, X. (2015). RASP (reconstruct ancestral state in phylogenies): A tool for historical biogeography. Molecular Phylogenetics and Evolution, 87, 46–49. 10.1016/j.ympev.2015.03.008 25819445

[ece310829-bib-0068] Yuan, L. M. , Deng, X. L. , Jiang, D. C. , Klaus, S. , Orlov, N. L. , Yang, K. , & Li, J. T. (2021). Geographical range evolution of the genus *Polypedates* (Anura: Rhacophoridae) from the Oligocene to present. Zoological Research, 42(1), 116–123. 10.24272/j.issn.2095-8137.2020.246 33258337 PMC7840456

[ece310829-bib-0069] Zachos, J. , Pagani, M. , Sloan, L. , Thomas, E. , & Billups, K. (2001). Trends, rhythms, and aberrations in global climate 65 ma to present. Science, 292(5517), 686–693. 10.1126/science.1059412 11326091

[ece310829-bib-0070] Zehady, A. K. , Ogg, J. G. , Fordham, B. G. , Palem, G. , Bobick, J. , & Ogg, G. M. (2020). Visualization of evolutionary relationships through geologic time in Timescale Creator. Applied Computing and Geosciences, 8, 100037. 10.1016/j.acags.2020.100037

[ece310829-bib-0071] Zhang, J. , Kapli, P. , Pavlidis, P. , & Stamatakis, A. (2013). A general species delimitation method with applications to phylogenetic placements. Bioinformatics, 29(22), 2869–2876. 10.1093/bioinformatics/btt499 23990417 PMC3810850

