# Supplementary material for: Integrated phylogenetic analyses reveal the evolutionary, biogeographic, and diversification history of Asian warty treefrog genus Theloderma (Anura, Rhacophoridae)
Source: Ecol Evol. 2023 Dec 21;13(12):e10829. doi: 10.1002/ece3.10829 (PMC10739124; doi:10.1002/ece3.10829)
Supplement: Supplementary file 1 — Data S1 [file ECE3-13-e10829-s001.zip › Supplementary materials.docx]

**Supplemetary Material**


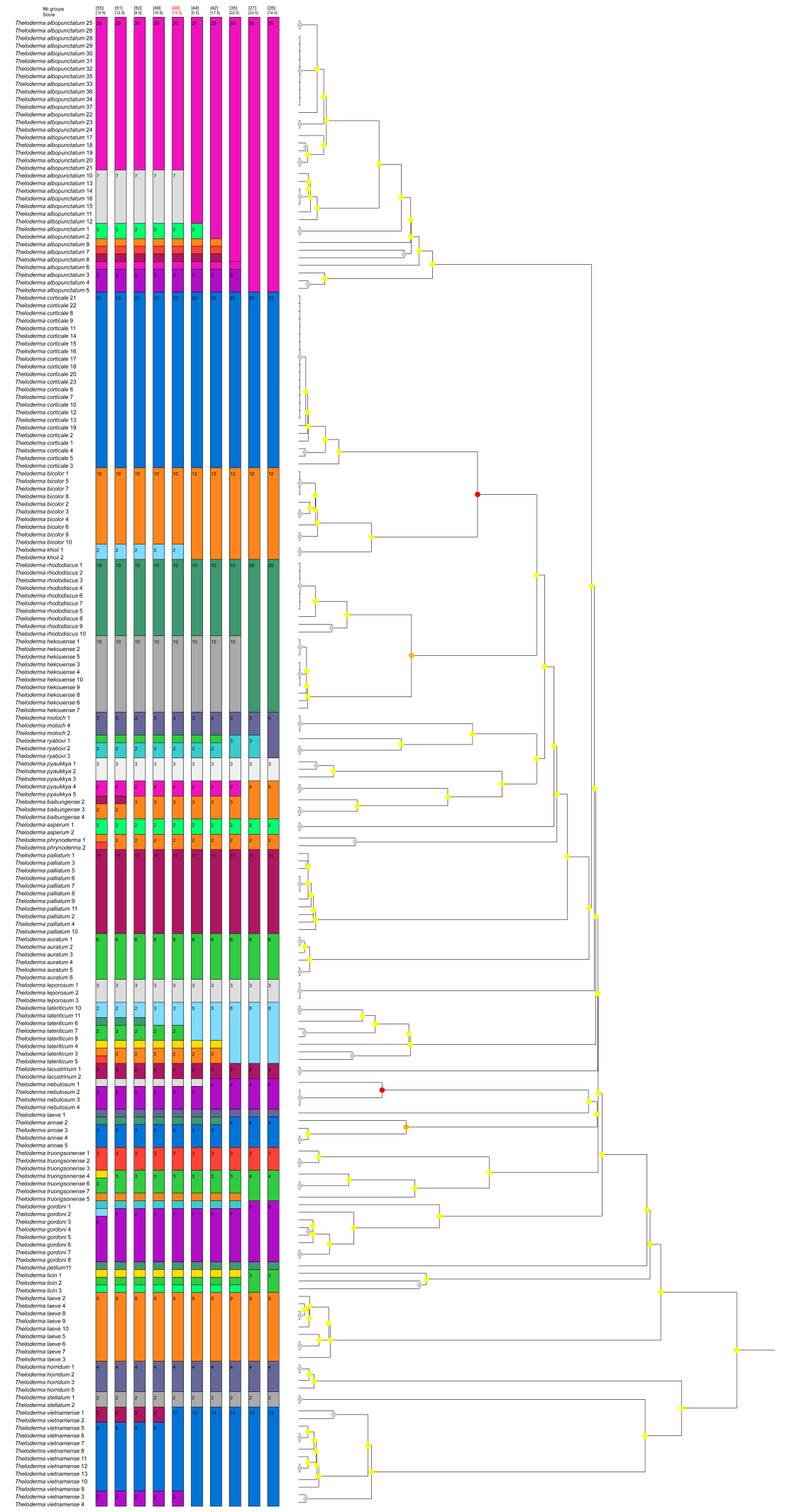


**Figure S1** Results of the analysis of species definition using ASAP for dataset 2. Details regarding the numbers at the tip of the species name are given in Table S1.


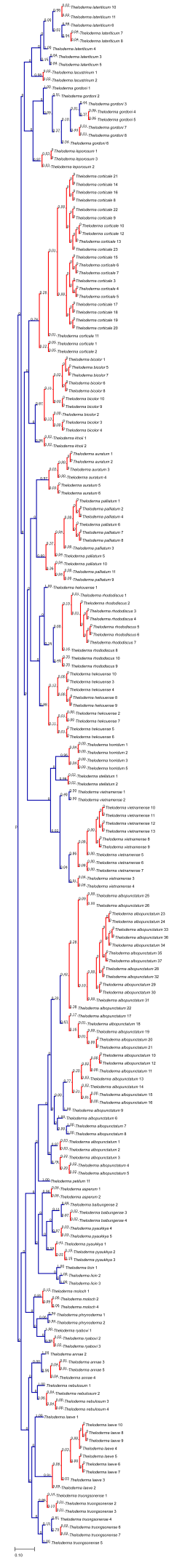


**Figure S2** Results of the analysis of species definition using bPTP for dataset 2. Details regarding the numbers at the tip of the species name are given in Table S1.


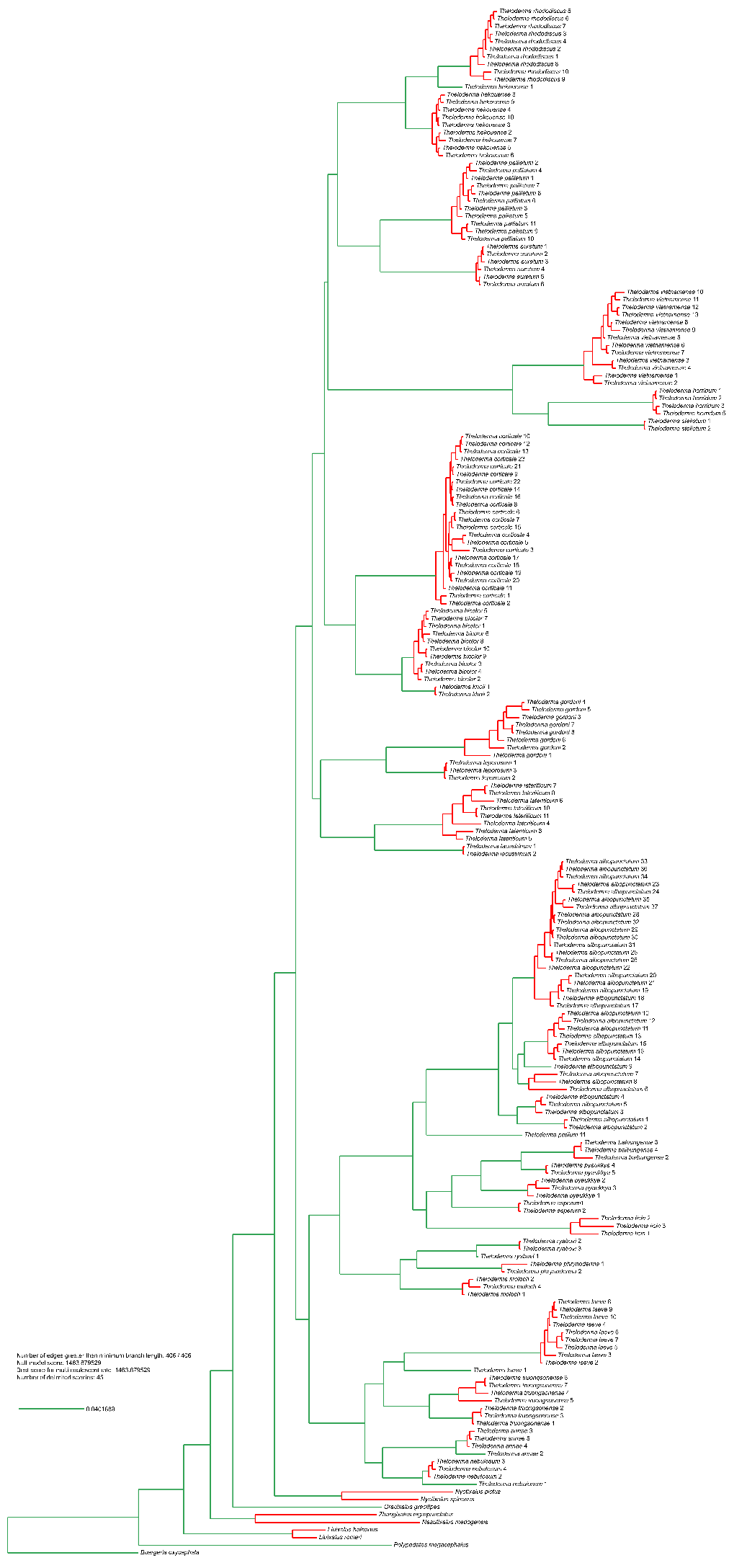


**Figure S3** Results of the analysis of species definition using mPTP for dataset 2. Details regarding the numbers at the tip of the species name are given in Table S1.


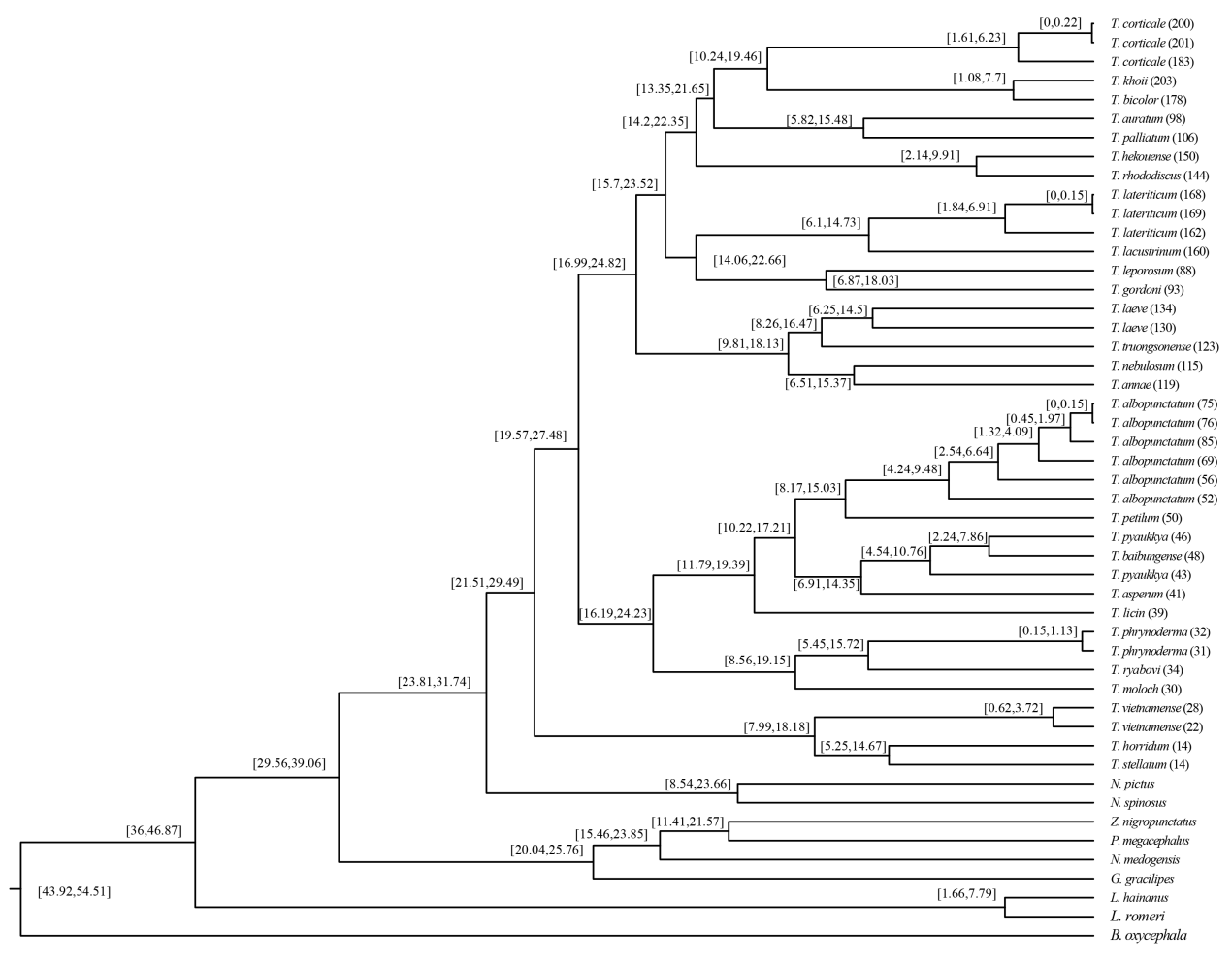


**Figure S4** Evolutionary timescales of the genus *Theloderma* and the outgroups were estimated in BEAST 1.8.2 using mitochondrial and nuclear genes (95% highest posterior density, HPD).


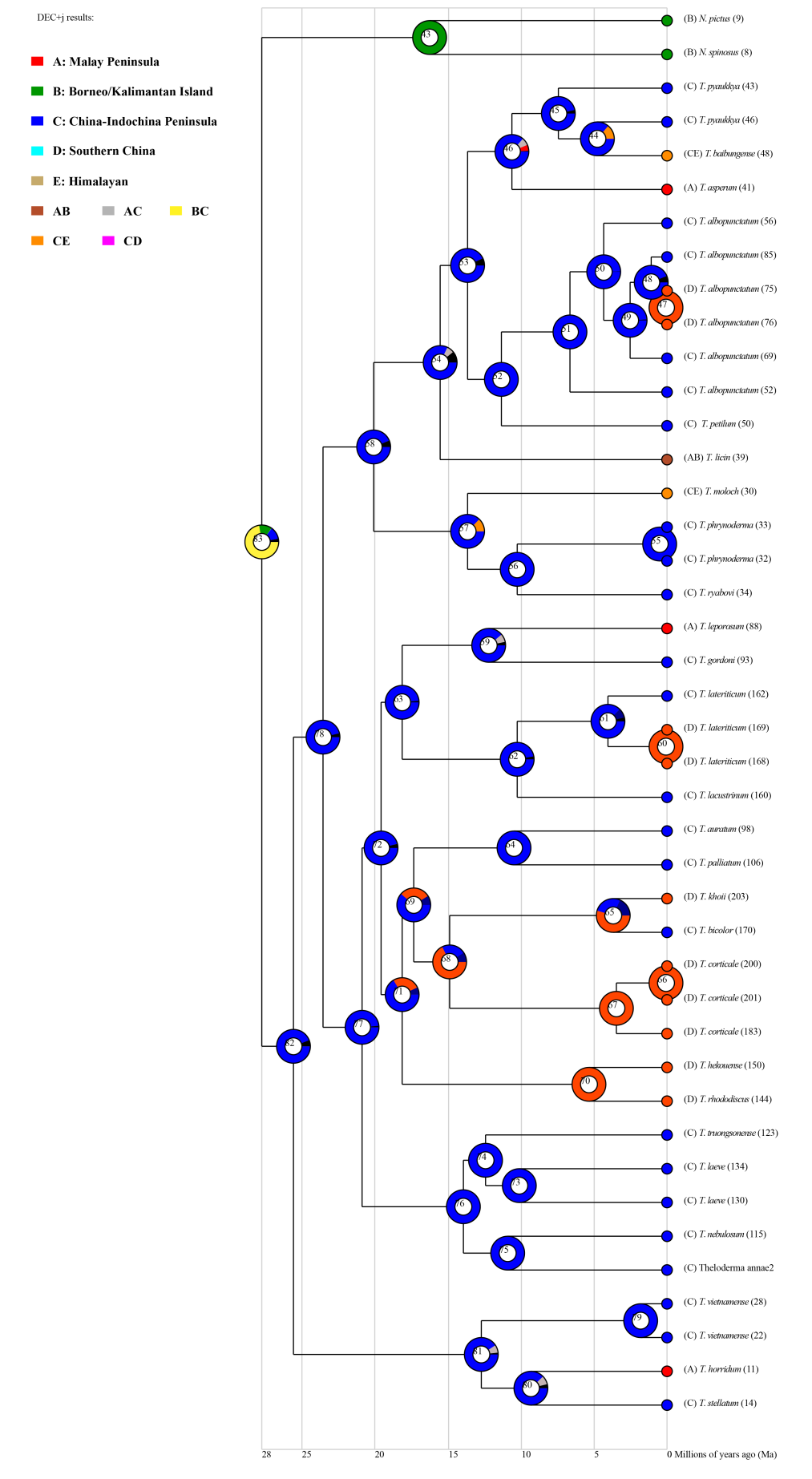


**Figure S5** Current distribution and reconstructed ancestral distribution of the genus *Theloderma*, estimated using BioGeoBEARS based on the second-best-fit model (DEC+j), and divergence time tree, estimated using BEAST based on the combined mtDNA and nuDNA data.


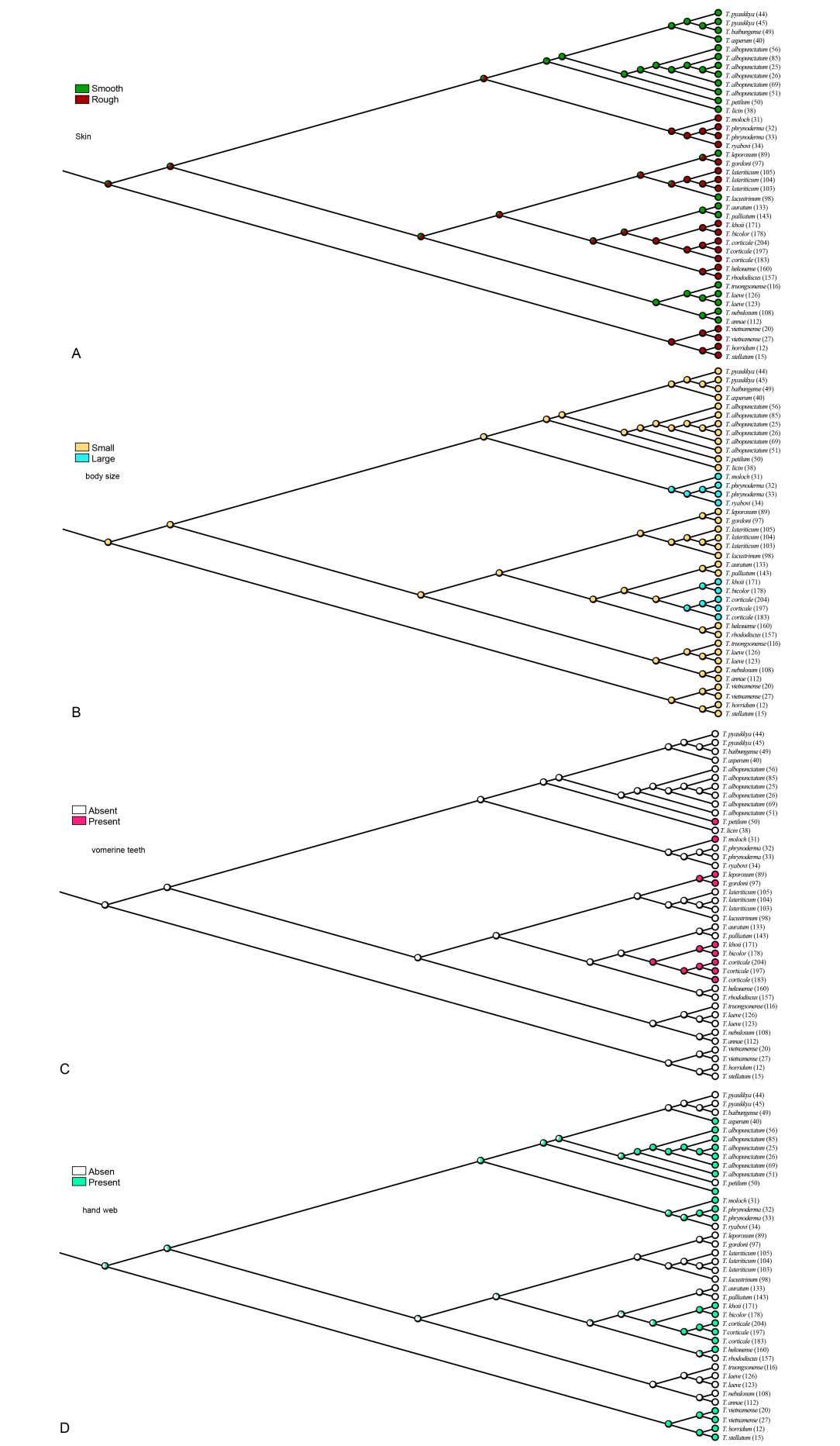


**Figure S6** Ancestral characteristics reconstructed using the ML method from MESQUITE v 3.70, with the four key characteristic state reconstructions mapped to the time-calibrated phylogeny of *Theloderma*. Tip circles denote characteristics used for ancestral state reconstruction, and pie charts at nodes denote the posterior probabilities of characteristic states. (A) Skin; (B) body size; (C) vomerine teeth; (D) hand webbing.

**Table S1** Localities, voucher information, and GenBank numbers for all samples used in this study. Bolded black indicates used for dataset 2.

| **ID** | **Species** | **Locality (* type localities)** | Voucher no  (**^#^ #** indicates from this study) | 16S | 12S | COI | BNDF | RHOD | TYR | RAG1 | SIA |
| --- | --- | --- | --- | --- | --- | --- | --- | --- | --- | --- | --- |
| 1 | *Buergeria oxycephala* | Hainan, China | SCUM 050267YJ | EU215524 | EU215524 |  | GQ285695 | EU215556 | EU215585 | GQ285758 |  |
| 2 | *Liuixalus romeri* | Mt. Wuzhi, Hainan, China | KIZ 061205YP | EU215528 | EU215528 |  | GQ285693 | EU215559 | EU215589 | GQ285756 |  |
| 3 | *Liuixalus hainanus* | Mt. Diaoluo, Hainan, China | 060401L | GQ285671 | GQ285671 |  | GQ285694 | GQ285785 | GQ285803 | GQ285757 |  |
| 4 | *Gracixalus gracilipes* | Pingbian, Yunnan, China | 060821196Rao | GQ285668 | GQ285668 |  | GQ285701 | GQ285789 | GQ285807 | GQ285764 |  |
| 5 | *Nasutixalus medogensis* | Medog, Tibet, China* | 6255Rao | GQ285679 | GQ285679 |  | GQ285690 | GQ285782 | GQ285800 | GQ285753 |  |
| 6 | *Polypedates megacephalus* | Motuo, Xizang, China | 6212Rao | GQ285685 | GQ285685 |  | GQ285706 | GQ285791 | GQ285809 | GQ285769 |  |
| 7 | *Zhangixalus nigropunctatus* | Weining, Guizhou, China | SCUM 070657L | EU215533 | EU215533 |  | GQ285704 | EU215563 | EU215593 | GQ285767 |  |
| 8 | *Nyctixalus spinosus* | Philippines | ACD 1043 | DQ283114 | DQ283114 |  |  | DQ283827 |  |  |  |
| 9 | *Nyctixalus pictus* | Lahad Datu Dist, Sabah, Malaysia | R081203 | AF458135 | AF458135 |  | GQ285698 | GQ285787 | GQ285805 | GQ285761 |  |
| 10 | *Theloderma horridum_*5 | Tha Le Ban, Satun, Thailand | ZMMU NAP-04015 | KT461890 | KT461890 |  |  |  |  |  |  |
| 11 | *Theloderma horridum*_3 | Malaysia | LJT W44 | KC465843 | KC465843 |  |  |  |  |  |  |
| **12** | ***Theloderma horridum*_2** | **Kenaboi, Negeri Sembilan, Malaysia** | **KUHE 52582** | **LC012861** | **LC012861** |  |  |  |  |  |  |
| 13 | *Theloderma horridum*_1 | Malaysia | LJT W45 | KC465842 | KC465842 |  |  |  |  |  |  |
| 14 | *Theloderma stellatum*_2 | Phliu, Chanthaburi, Thailand | stellatum-1 | KT461918 |  |  |  |  |  |  |  |
| **15** | ***Theloderma stellatum*_1** | **Nang Rong, Nakhon Nayok, Thailand** | **ZMMUNAP-03961** | **KT461917** |  |  |  |  |  |  |  |
| 16 | *Theloderma vietnamense*_2 | Mae Yom, Thailand | KUHE 22056 | LC012862 | LC012862 |  |  |  |  |  |  |
| 17 | *Theloderma vietnamense*_1 | Savannakhet, Laos | NCSM 76490 | KU561886 |  | KU244464 |  |  |  |  |  |
| 18 | *Theloderma vietnamense*_4 | Phu Yen, Vietnam | VNMN 3686 | KJ802922 | KJ802922 |  |  |  |  |  |  |
| 19 | *Theloderma vietnamense*_3 | Phu Yen, Vietnam | VNMN 3687 | KJ802923 | KJ802923 |  |  |  |  |  |  |
| **20** | ***Theloderma vietnamense*13** | **Binh Thuan, Vietnam** | **NCSM 80384** | **KU561887** |  | **KU244463** | **KU561888** | **KU561891** | **KU561897** |  | **KU561894** |
| 21 | *Theloderma vietnamense*_12 | Binh Thuan, Vietnam | AMS R 173283 | JN688170 |  |  |  |  |  |  | KU244422 |
| 22 | *Theloderma vietnamense*_9 | Phu Quoc, Kien Giang, Vietnam* | ZMMU NAP-03724 | KT461888 | KT461888 |  |  |  |  |  |  |
| 23 | *Theloderma vietnamense*_8 | Phu Quoc, Kien Giang, Vietnam | ZMMU NAP-03723 | KT461919 |  |  |  |  |  |  |  |
| 24 | *Theloderma vietnamense*_11 | Nam Cat Tien, Dong Nai, Vietnam | ZMMU NAP-00707 | KT461889 | KT461889 |  |  |  |  |  |  |
| 25 | *Theloderma vietnamense*_10 | Koh Kong, Cambodia | FMNH 267765 | KU561885 |  | KU244462 |  |  |  |  |  |
| 26 | *Theloderma vietnamense*_5 | Lo Go – Xa Mat, Tay Ninh, Vietnam | ZMMU NAP-03680 | KT461921 |  |  |  |  |  |  |  |
| **27** | ***Theloderma vietnamense*_7** | **Mondol Kir, Cambodia** | **AMS R 174047** | **JN688171** |  | **KU244460** | **KU244391** | **KU244335** | **KU244356** |  |  |
| 28 | *Theloderma vietnamense*_6 | Mondolkiri, Cambodia | FMNH 262786 | KU561884 |  | KU244461 |  |  |  |  |  |
| 29 | *Theloderma moloch*_1 | Yingjiang Yunnan, China | GXNU YU000115 | MT509809 |  |  |  |  |  |  |  |
| 30 | *Theloderma moloch*_4 | Medog, Tibet, China* | KIZYPX31941 | KU243081 | KU243081 |  |  |  |  |  |  |
| **31** | ***Theloderma moloch*_2** | **Arunachal Pradesh, India** | **SDBDU 2011.345** | **KU169993** | **KU170015** |  |  | **KU169944** |  | **KU169968** |  |
| **32** | ***Theloderma phrynoderma*_2** | **Tanintharyi, Myanmar** | **CAS 243920** | **KJ128282** | **KJ128280** | **KU244448** | **KU244402** | **KJ128278** | **KJ128276** |  | **KU244410** |
| **33** | ***Theloderma phrynoderma*_1** | **Tanintharyi, Myanmar** | **CAS 243910** | **KJ128283** | **KJ128281** | **KU244449** | **KU244404** | **KJ128279** | **KJ128277** |  | **KU244411** |
| **34** | ***Theloderma ryabovi*_1** | **Mang Canh, Kon Tum, Vietnam*** | **VNMN 3924** | **LC012860** | **LC012860** |  |  |  |  |  |  |
| 35 | *Theloderma ryabovi*_3 | Mang Canh, Kon Tum, Vietnam | yabovi-1 | KT461914 |  |  |  |  |  |  |  |
| 36 | *Theloderma ryabovi*_2 | Mang Canh, Kon Tum, Vietnam | yabovi-2 | KT461915 |  |  |  |  |  |  |  |
| 37 | *Theloderma licin*_1 | Nakon Si Tamarat, Thailand | KUHE 19426 | LC012859 | LC012859 |  |  |  |  |  |  |
| **38** | ***Theloderma licin*_3** | **Indonesia** | **MVZ 272184** | **KU244368** |  | **KU244447** | **KU244384** | **KU244333** | **KU244346** |  | **KU244425** |
| 39 | *Theloderma licin*_2 | Selangor, Malaysia | KUHE 52599 | KJ802920 | KJ802920 |  |  |  |  |  |  |
| **40** | ***Theloderma asperum*_2** | **Fraser’s Hill, Pahang, Malaysia** | **ZRC1.1. 9321** | **GQ204725** | **GQ204725** |  |  | **GQ204661** |  | **GQ204606** |  |
| 41 | *Theloderma asperum*_1 | Perak, Malaysia | pet trade | KT461929 |  |  |  |  |  |  |  |
| 42 | *Theloderma pyaukkya*_1 | Yingjiang Yunnan, China | GXNU YU000116 | MT509810 |  | MT522176 |  |  |  |  |  |
| 43 | *Theloderma pyaukkya*_3 | Mohnyin, Kachin, Myanmar* | CAS 226113* | KU244361 |  | KU244443 | KU244399 | KU244331 | KU244339 |  | KU244421 |
| **44** | ***Theloderma pyaukkya*_2** | **Mohnyin, Kachin, Myanmar*** | **CAS 226114*** | **KU244360** |  | **KU244444** | **KU244400** | **KU244330** | **KU244344** |  | **KU244419** |
| **45** | ***Theloderma pyaukkya*_5** | **Chin, Myanmar** | **CAS 234869** | **KU244370** |  | **KU244445** | **KU244398** | **KU244332** | **KU244342** |  | **KU244418** |
| 46 | *Theloderma pyaukkya*_4 | Chin, Myanmar | AS 234857 | KU244371 |  | KU244446 | KU244397 | KU244329 | KU244341 |  | KU244426 |
| 47 | *Theloderma baibungense*_2 | Mizoram, India | MZUHC 466 | MT814052 |  |  |  |  |  |  |  |
| 48 | *Theloderma baibungense*_4 | Beibeng, Medog, Tibet, China* | KIZ-020453 (YPX31940) | KU981089 | KU981089 |  |  |  |  |  |  |
| **49** | ***Theloderma baibungense*_3** | **Beibeng, Medog, Tibet, China*** | **KIZYPX37270** | **KU243080** | **KU243080** |  |  |  |  |  |  |
| **50** | ***Theloderma petilum*_11** | **Muong Nhe, Dien Bien, Vietnam** | **HNUE MNA. 2012.0001** | **KJ802925** | **KJ802925** |  |  |  |  |  |  |
| **51** | ***Theloderma albopunctatum*_2** | **Nghe An, Vietnam** | **AMS R177656** | **KU244376** |  | **KU244442** | **KU244392** | **KU244317** | **KU244349** |  | **KU244429** |
| 52 | *Theloderma albopunctatum*_1 | Xuan Lien, Thanh Hoa, Vietnam | VNMN 4406 | LC012856 | LC012856 |  |  |  |  |  |  |
| 53 | *Theloderma albopunctatum*_3 | Ta, Son La, Vietnam | VNMN PAE 262 | LC012857 | LC012857 |  |  |  |  |  |  |
| 54 | *Theloderma albopunctatum*_5 | Changdao, Doi, Thailand | KUHE 23736 | LC012858 | LC012858 |  |  |  |  |  |  |
| 55 | *Theloderma albopunctatum*_4 | Shan, Myanmar | CAS 241559 | KU244362 |  | KU244441 | KU244401 | KU244322 | KU244352 |  | KU244416 |
| **56** | ***Theloderma albopunctatum*_6** | **Savannakhet, Laos** | **NCSM 76483** | **KU244372** |  | **KU244437** | **KU244386** | **KU244327** | **KU244343** |  | **KU244408** |
| 57 | *Theloderma albopunctatum*_8 | Ngoc Linh, Kon Tum, Vietnam | VNMN 4404 | LC012854 | LC012854 |  |  |  |  |  |  |
| 58 | *Theloderma albopunctatum*_7 | Kon Ka Kinh, Gia Lai, Vietnam | VNMN 4405 | LC012855 | LC012855 |  |  |  |  |  |  |
| 59 | *Theloderma albopunctatum*_9 | Savannakhet, Laos | NCSM 76167 | KU244367 |  | KU244434 | KU244385 | KU244324 | KU244340 |  | KU244409 |
| 60 | *Theloderma albopunctatum*_14 | Phu Luang, Loei, Thailand | JC 02506 | MW405909 |  | MW648780 | MW648786 | MW648777 | MW648789 |  | MW648783 |
| 61 | *Theloderma albopunctatum*_16 | Phu Luang, Loei, Thailand | PT 02499 | MW405910 |  | MW648781 | MW648787 | MW648778 | MW648790 |  | MW648784 |
| 62 | *Theloderma albopunctatum*_15 | Nam Nao, Phetchabun, Thailand | PT 02378 | MW405908 |  | MW648779 | MW648785 | MW648776 | MW648788 |  | MW648782 |
| 63 | *Theloderma albopunctatum*_13 | Luang Phabang | NCSM 79445 | KU244369 |  | KU244439 | KU244388 | KU244323 | KU244337 |  | KU244415 |
| 64 | *Theloderma albopunctatum*_10 | Nan, Thailand | FMNH 270722 | KU244366 |  | KU244440 | KU244405 | KU244325 | KU244345 |  | KU244412 |
| 65 | *Theloderma albopunctatum*_12 | Luang Phabang, Laos | NCSM 79444 | KU244379 |  | KU244438 | KU244387 | KU244326 | KU244347 |  | KU244414 |
| 66 | *Theloderma albopunctatum*_11 | Houey Thao, Luang Prabang, Laos | K3153/2006.2573 | KR828081 |  | KR087944 |  |  |  |  |  |
| 67 | *Theloderma albopunctatum*_17 | Kon Plong, Kon Tum, Vietnam | asperum-2 | KT461908 |  |  |  |  |  |  |  |
| 68 | *Theloderma albopunctatum*_18 | Vinh Phuc, Vietnam | VNMN 3540 | KJ802914 | KJ802914 |  |  |  |  |  |  |
| **69** | ***Theloderma albopunctatum*_19** | **Jinping Yunnan, China** | **060821203Rao** | **GQ285677** | **GQ285677** |  | **GQ285697** | **GQ285786** | **GQ285804** | **GQ285760** |  |
| 70 | *Theloderma albopunctatum*_21 | Jinping, Yunnan, China |  | EF646374 |  |  |  |  |  |  |  |
| 71 | *Theloderma albopunctatum*_20 | Jinping, Yunnan, China | KIZ060821201 | EF564521 | EF564449 |  |  | EU924562 | EU924590 | EU924534 |  |
| 72 | *Theloderma albopunctatum*_22 | Yinggeling, Hainan, China | HN0806100 | GQ285678 | GQ285678 |  |  |  |  |  |  |
| 73 | *Theloderma albopunctatum*_24 | Jinxiu, Guangxi, China |  | EF646375 |  |  |  |  |  |  |  |
| 74 | *Theloderma albopunctatum*_23 | Jinxiu, Guangxi, China* | KIZ060821217 | EF564522 | EF564450 |  |  |  |  |  |  |
| **75** | ***Theloderma albopunctatum*_25** | **Bangliang, Jingxi, Guangxi, China** | **GZNU20210731006^#^** | **OP537793** | **OP537793** | **OP531848** | **OP561734** | **OP561738** | **OP561758** | **OP561746** | **OP561750** |
| **76** | ***Theloderma albopunctatum*_26** | **Bangliang, Jingxi, Guangxi, China** | **GZNU20210731006^#^** | **OP537794** | **OP537794** | **OP531849** | **OP561735** | **OP561739** | **OP561759** | **OP561747** | **OP561751** |
| 77 | *Theloderma albopunctatum*_29 | Cat Ba, Hai Phong, Vietnam | ZMMU NAP-03575 | KT461912 |  |  |  |  |  |  |  |
| 78 | *Theloderma albopunctatum*_30 | Cat Ba, Hai Phong, Vietnam | ZMMU NAP-03557 | KT461910 |  |  |  |  |  |  |  |
| 79 | *Theloderma albopunctatum*_28 | Cat Ba, Hai Phong, Vietnam | ZMMU NAP-03566 | KT461911 |  |  |  |  |  |  |  |
| 80 | *Theloderma albopunctatum*_32 | Kon Plong, Kon Tum, Vietnam | asperum-1 | KT461884 | KT461884 |  |  |  |  |  |  |
| 81 | *Theloderma albopunctatum*_31 | Kon Plong, Kon Tum, Vietnam | asperum-3 | KT461909 |  |  |  |  |  |  |  |
| 82 | *Theloderma albopunctatum*_35 | Tam Dao, Vin Phu, Vietnam | AMS JR2888 | KU244377 |  |  |  |  |  |  |  |
| 83 | *Theloderma albopunctatum*_34 | Tuyen Quang, Vietnam | ROM 30246 | AF458148 | AF458148 |  |  |  |  |  |  |
| 84 | *Theloderma albopunctatum*_37 | Tam Dao, Vin Phu, Vietnam | VNMN JR2887 | KU244375 |  | KU244431 | KU244381 | KU244318 | KU244338 |  | KU244407 |
| **85** | ***Theloderma albopunctatum*_36** | **Tam Dao, Vin Phu, Vietnam** | **VNMN JR2888** | **LC012853** | **LC012853** | **KU244432** | **KU244382** | **KU244319** | **KU244351** |  | **KU244406** |
| 86 | *Theloderma albopunctatum*_33 | Lao Cai, Vietnam | VNMN J2916 | KJ802913 | KJ802913 |  |  |  |  |  |  |
| 87 | *Theloderma leporosum*_1 | Malaysia | LJT W46 | KC465841 | KC465841 |  |  |  |  |  |  |
| 88 | *Theloderma leporosum*_3 | Selangor, Malaysia | leporosum-1 | KT461922 |  |  |  |  |  |  |  |
| **89** | ***Theloderma leporosum*_2** | **Negeri Sembilan, Malaysia** | **KUHE 52581** | **AB847128** | **AB847128** |  |  |  |  |  |  |
| 90 | *Theloderma gordoni*_1 | Ngoc Linh, Kon Tum, Vietnam | VNMN 4407 | LC012852 | LC012852 |  |  |  |  |  |  |
| 91 | *Theloderma gordoni*_2 | Nghe An, Vietnam | VNMN 03013 | JN688167 |  |  |  |  |  |  |  |
| 92 | *Theloderma gordoni*_3 | Son La, Vietnam | VNMN PAE217 | KJ802918 | KJ802918 |  |  |  |  |  |  |
| 93 | *Theloderma gordoni*_8 | Xishuangbanna, Yunnan, China | SYNU607001 | MF467910 | MF467910 |  |  |  |  |  |  |
| 94 | *Theloderma gordoni*_7 | Puer, Yunnan, China | HM-IBE20150901 | MF467909 | MF467909 |  |  |  |  |  |  |
| 95 | *Theloderma gordoni*_6 | Houaphan, Laos | KUHE 32447 | KJ802919 | KJ802919 |  |  |  |  |  |  |
| 96 | *Theloderma gordoni*_5 | / | FMNH 231095 | AF268256 | AF268254 |  |  |  |  |  |  |
| **97** | ***Theloderma gordoni*_4** | **Tam Dao, Vin Phu, Vietnam** | **MVZ 225139** | **KU244363** |  | **KU244451** | **KU244395** | **KU244334** | **KU244358** |  | **KU244423** |
| **98** | ***Theloderma lacustrinum*_2** | **Nam Lik, Feuang, Vientiane, Laos*** | **NCSM84682** | **KX095245** |  |  |  |  |  |  |  |
| 99 | *Theloderma lacustrinum*_1 | Nam Lik, Feuang, Vientiane, Laos* | NCSM84683 | KX095246 |  |  |  |  |  |  |  |
| 100 | *Theloderma lateriticum*_3 | SaPa, Lao Cai, Vietnam* | AMNH 168757/IEBR A. 0860 | LC012848 | LC012848 |  |  |  |  |  |  |
| 101 | *Theloderma lateriticum*_5 | Lao Cai, Vietnam | IEBR 3745 | LC641703 |  |  |  |  |  |  |  |
| 102 | *Theloderma lateriticum*_4 | Ta Sua, Son La, Vietnam | VNMN PAE 226 | LC012849 | LC012849 |  |  |  |  |  |  |
| **103** | *Theloderma lateriticum*_10 | **Bangliang, Jingxi, Guangxi, China** | **GZNU20210731001^#^** | **OP537823** | **OP537823** | **OP531852** | **OP561736** | **OP561740** | **OP561756** | **OP561748** | **OP561755** |
| **104** | *Theloderma lateriticum*_11 | **Bangliang, Jingxi, Guangxi, China** | **GZNU20210731002^#^** | **OP537824** | **OP537824** | **OP531853** | **OP561737** | **OP561741** | **OP561757** | **OP561749** | **OP561756** |
| 105 | *Theloderma lateriticum*_6 | Shiwandashan, Shangsi, Guangxi, China | NHMG201704001 | MH521262 |  |  |  |  |  |  |  |
| 106 | *Theloderma lateriticum*_8 | Yen Tu, Bac Giang, Vietnam | VNMN 1216 | LC012851 | LC012851 |  |  |  |  |  |  |
| 107 | *Theloderma lateriticum*_7 | Yen Tu, Bac Giang, Vietnam | VNMN 1215 | LC012850 | LC012850 |  |  |  |  |  |  |
| **108** | ***Theloderma nebulosum*_1** | **Ngoc Linh, Kon Tum, Vietnam*** | **VNMN 39588** | **LC012845** | **LC012845** |  |  |  |  |  |  |
| 109 | *Theloderma nebulosum*_2 | Ngoc Linh, Kon Tum, Vietnam* | UNS00141 | JN688169 |  |  |  |  |  |  |  |
| 110 | *Theloderma nebulosum*_4 | Ngoc Linh, Kon Tum, Vietnam* | ROM 39588 | KT461887 | KT461887 |  |  |  |  |  |  |
| 111 | *Theloderma nebulosum*_3 | Ngoc Linh, Kon Tum, Vietnam* | AMS R 173409 | JN688168 |  |  |  |  |  |  |  |
| **112** | ***Theloderma annae*_2** | **Lac Son, Hoa Binh, Vietnam** | **ZMMU NAP-05558** | **MG917766** | **MG917766** |  |  |  |  |  |  |
| 113 | *Theloderma annae*_5 | Lac Son District, Hoa Binh, Vietnam* | IEBR 3732* | LC168170 |  |  |  |  |  |  |  |
| 114 | *Theloderma annae*_4 | Lac Son District, Hoa Binh, Vietnam* | IEBR:3733 | LC168171 |  |  |  |  |  |  |  |
| 115 | *Theloderma annae*_3 | Lac Son District, Hoa Binh, Vietnam* | IEBR:3734 | LC168172 |  |  |  |  |  |  |  |
| **116** | ***Theloderma truongsonense*_1** | **Hon ba, Khanh Hoa, Vietnam** | **VNMN 4402** | **LC012847** | **LC012847** |  |  |  |  |  |  |
| 117 | *Theloderma truongsonense*_3 | Hon ba, Khanh Hoa, Vietnam | ZMMUABV-00301 | KT461882 | KT461882 |  |  |  |  |  |  |
| 118 | *Theloderma truongsonense*_2 | Hon ba, Khanh Hoa, Vietnam | ZMMU ABV-00319 | KT461924 |  |  |  |  |  |  |  |
| 119 | *Theloderma truongsonense*_4 | Phong Nha – Ke Bang, Quang Binh, Vietnam | ROM 39363 | KT461925 |  |  |  |  |  |  |  |
| 120 | *Theloderma truongsonense*_5 | Quang Binh, Vietnam | AMS R 171510 | JN688174 |  |  |  |  |  |  |  |
| 121 | *Theloderma truongsonense*_7 | Kon Ka Kinh, Gia Lai, Vietnam | ZMMU NAP-07142 | MG917762 | MG917762 |  |  |  |  |  |  |
| 122 | *Theloderma truongsonense*_6 | Kon Ka Kinh, Gia Lai, Vietnam | ZMMU NAP-07143 | MG917763 | MG917763 |  |  |  |  |  |  |
| **123** | ***Theloderma laeve*_1** | **Mang Yang, Gia Lai, Vietnam** | **VNMN 4403** | **LC012846** | **LC012846** |  |  |  |  |  |  |
| 124 | *Theloderma laeve*_2 | Cat Loc, Lam Dong, Vietnam | ZMMU NAP-01644 | KT461907 | / |  |  |  |  |  |  |
| 125 | *Theloderma laeve*_3 | Cat Loc, Lam Dong, Vietnam | ZMMU NAP-01640 | KT461928 | / |  |  |  |  |  |  |
| **126** | ***Theloderma laeve*_5** | **Bu Gia Map, Binh Phuoc, Vietnam** | **ZMMU NAP-02906** | **KT461883** | **KT461883** |  |  |  |  |  |  |
| 127 | *Theloderma laeve*_7 | Bu Gia Map, Binh Phuoc, Vietnam | ZMMU NAP-02907 | KT461905 | / |  |  |  |  |  |  |
| 128 | *Theloderma laeve*_6 | Bu Gia Map, Binh Phuoc, Vietnam | ZMMU NAP-02908 | KT461906 | / |  |  |  |  |  |  |
| 129 | *Theloderma laeve*_4 | Cat Loc, Lam Dong, Vietnam | ZMMU NAP-01645 | KT461913 | / |  |  |  |  |  |  |
| 130 | *Theloderma laeve*_9 | Cat Loc, Lam Dong, Vietnam | ZMMU NAP-03383 | KT461900 | KT461892 |  |  |  |  |  |  |
| 131 | *Theloderma laeve*_8 | Cat Loc, Lam Dong, Vietnam | ZMMU NAP-03408 | KT461898 | KT461897 |  |  |  |  |  |  |
| 132 | *Theloderma laeve*_10 | Cat Loc, Lam Dong, Vietnam | ZMMU NAP-03409 | KT461920 |  |  |  |  |  |  |  |
| **133** | ***Theloderma auratum*_1** | **Kon Chu Rang, Gia Lai, Vietnam*** | **ZMMU A-5830** | **MG917769** | **MG917769** |  |  |  |  |  |  |
| 134 | *Theloderma auratum*_2 | Kon Chu Rang, Gia Lai, Vietnam* | ZMMU A-5829 | MG917768 | MG917768 |  |  |  |  |  |  |
| 135 | *Theloderma auratum*_3 | Kon Chu Rang, Gia Lai, Vietnam* | ZMMU A-5830* | MG917767 | MG917767 |  |  |  |  |  |  |
| 136 | *Theloderma auratum*_4 | Kon Chu Rang, Gia Lai, Vietnam* | ZMMU A-5831 | MG917770 | MG917770 |  |  |  |  |  |  |
| 137 | *Theloderma auratum*_6 | Thac Nham, Kon Tum, Vietnam | ZMMU A-5832 | MG917771 | MG917771 |  |  |  |  |  |  |
| 138 | *Theloderma auratum*_5 | Thac Nham, Kon Tum, Vietnam | ZMMU NAP-06402 | MG917772 | MG917772 |  |  |  |  |  |  |
| 139 | *Theloderma palliatum*_1 | Chu Yang Sin, Dak Lak, Vietnam | ZMMU NAP-02736 | KT461927 |  |  |  |  |  |  |  |
| 140 | *Theloderma palliatum*_4 | Chu Yang Sin, Dak Lak, Vietnam | ZMMU NAP-02735 | LC012843 |  |  |  |  |  |  |  |
| 141 | *Theloderma palliatum*_2 | Chu Yang Sin, Dak Lak, Vietnam | ZMMU NAP -02736 | LC012844 |  |  |  |  |  |  |  |
| 142 | *Theloderma palliatum*_5 | Bi Doup – Nui Ba, Lam Dong, Vietnam* | AMS R 173131 | JN688173 |  |  |  |  |  |  |  |
| **143** | ***Theloderma palliatum*_3** | **Bi Doup – Nui Ba, Lam Dong, Vietnam** | **AMS R 173130** | **JN688172** |  |  |  |  |  |  |  |
| 144 | *Theloderma palliatum*_6 | Chu Yang Sin, Dak Lak, Vietnam | NAP02735 | KT461926 |  |  |  |  |  |  |  |
| 145 | *Theloderma palliatum*_7 | Chu Yang Sin, Dak Lak, Vietnam | ZMMU NAP -02756 | KT461930 |  |  |  |  |  |  |  |
| 146 | *Theloderma palliatum*_8 | Chu Yang Sin, Dak Lak, Vietnam | ZMMU NAP-02757 | KT461904 | KT461896 |  |  |  |  |  |  |
| 147 | *Theloderma palliatum*_9 | Bi Doup – Nui Ba, Lam Dong, Vietnam | ZMMU NAP-0251 1 | KT461902 | KT461894 |  |  |  |  |  |  |
| 148 | *Theloderma palliatum*_11 | Bi Doup-Nui Ba, Lam Dong, Vietnam* | ZMMU NAP-02516 | KT461903 | KT461895 |  |  |  |  |  |  |
| 149 | *Theloderma palliatum*_10 | Bi Doup – Nui Ba, Lam Dong, Vietnam | ZMMU NAP-01846 | KT461901 | KT461893 |  |  |  |  |  |  |
| 150 | *Theloderma rhododiscus*_8 | Huanjiang, Guangxi, China | GXNU YU000309 | OL843959 |  | OL843974 |  |  |  |  |  |
| 151 | *Theloderma rhododiscus*_9 | Longlin, Guangxi, China | GXNU YU000318 | OL843960 |  | OL843975 |  |  |  |  |  |
| 152 | *Theloderma rhododiscus*_10 | Longlin, Guangxi, China | GXNU YU000319 | OL843961 |  | OL843976 |  |  |  |  |  |
| 153 | *Theloderma rhododiscus*_1 | Guangxi, China | CIB GX200807048 | KJ802921 | KJ802921 |  |  |  |  |  |  |
| 154 | *Theloderma rhododiscus*_2 | Guangxi, China | CIB GX200807017 | LC012842 | LC012842 |  |  |  |  |  |  |
| 155 | *Theloderma rhododiscus*_4 | Jinxiu, Guangxi, China | KIZ 060821063 | EF564533 | EF564461 |  |  | EU924563 | EU924591 | EU924535 |  |
| 156 | *Theloderma rhododiscus*_3 | Jinxiu, Guangxi, China | KIZ 060821170 | EF564534 | EF564462 |  |  |  |  |  |  |
| **157** | ***Theloderma rhododiscus*_5** | **Dayaoshan, Guangxi, China** | **SCUM 061102L** | **EU215530** | **EU215530** |  | **GQ285696** | **EU215555** | **EU215586** | **GQ285759** |  |
| 158 | *Theloderma rhododiscus*_7 | Jinxiu, Guangxi, China | GXNU YU000070 | OL843958 |  | OL843973 |  |  |  |  |  |
| 159 | *Theloderma rhododiscus*_6 | Jinxiu, Guangxi, China | GXNU YU000069 | OL843957 |  | OL843972 |  |  |  |  |  |
| **160** | ***Theloderma hekouense*_1** | **Tay Con Linh, Ha Giang, Vietnam** | **AMNH A163892** | **DQ283392** | **DQ283392** |  |  | **DQ284007** | **DQ282998** |  | **DQ282853** |
| 161 | *Theloderma hekouense*_5 | Hekou Yunnan, China | GXNU YU000397 | OL843962 |  | OL843977 |  |  |  |  |  |
| 162 | *Theloderma hekouense*_2 | Tay Con Linh, Ha Giang, Vietnam | AMNH A163893 | DQ283393 |  |  |  |  |  |  |  |
| 163 | *Theloderma hekouense*_7 | Hekou, Yunnan, China | GXNU YU000412 | OL843964 |  | OL843979 |  |  |  |  |  |
| 164 | *Theloderma hekouense*_6 | Hekou, Yunnan, China | GXNU YU000398 | OL843963 |  | OL843978 |  |  |  |  |  |
| 165 | *Theloderma hekouense*_4 | Hekou, Yunnan, China | HHU-WJHK01 | KY495639 | KY495630 |  |  |  |  |  |  |
| 166 | *Theloderma hekouense*_3 | Hekou, Yunnan, China | HHU-WJHK02 | KY495640 | KY495631 |  |  |  |  |  |  |
| 167 | *Theloderma hekouense*_8 | Hekou, Yunnan, China | GXNU YU000413 | OL843965 |  | OL843980 |  |  |  |  |  |
| 168 | *Theloderma hekouense*_9 | Hekou, Yunnan, China | GXNU YU000495 | OL843966 |  | OL843981 |  |  |  |  |  |
| 169 | *Theloderma hekouense*_10 | Hekou, Yunnan, China | GXNU YU000496 | OL843967 |  | OL843982 |  |  |  |  |  |
| 170 | *Theloderma khoii*_2 | Ha Giang, Viet Nam | VNMN 012758 | LC641702 | LC641702 |  |  |  |  |  |  |
| **171** | ***Theloderma khoii*_1** | **Ha Giang, Viet Nam** | **VNMN 012757** | **LC641701** | **LC641701** |  |  |  |  |  |  |
| 172 | *Theloderma bicolor*_2 | Jingdong, Yunnan, China | KIZ046744 | KY495634 | KY495625 |  |  |  |  |  |  |
| 173 | *Theloderma bicolor*_4 | Luechun, Yunnan, China | LC1 | KY495632 | KY495623 |  |  |  |  |  |  |
| 174 | *Theloderma bicolor*_3 | Luechun, Yunnan, China | LC2 | KY495633 | KY495624 |  |  |  |  |  |  |
| 175 | *Theloderma bicolor*_9 | Lai Chau, Viet Nam | VNMN 010811 | LC641699 | LC641699 |  |  |  |  |  |  |
| 176 | *Theloderma bicolor*_10 | Lai Chau, Viet Nam | VNMN 010821 | LC641700 | LC641700 |  |  |  |  |  |  |
| 177 | *Theloderma bicolor*_8 | Lao Cai, Vietnam | VNMN 3536 | KJ802915 | KJ802915 |  |  |  |  |  |  |
| **178** | ***Theloderma bicolor*_1** | **SaPa, Lao Cai, Vietnam** | **VNMN 1394** | **JX046475** | **KF991263** |  | **KF991305** |  |  | **KF991342** | **KF991366** |
| 179 | *Theloderma bicolor*_7 | Cuc Phuong, Ninh Binh, Vietnam | bicolor-2 | KT461923 |  |  |  |  |  |  |  |
| 180 | *Theloderma bicolor*_6 | Cuc Phuong, Ninh Binh, Vietnam | bicolor-3 | KT461899 | KT461891 |  |  |  |  |  |  |
| 181 | *Theloderma bicolor*_5 | SaPa, Lao Cai, Vietnam | IEBR A.2011.4 | JX046474 |  |  |  |  |  |  |  |
| 182 | *Theloderma corticale*_2 | Tam Dao, Vin Phuc, Vietnam | ZMMU NAP-06328 | MG917764 | MG917764 |  |  |  |  |  |  |
| **183** | ***Theloderma corticale*_1** | **Tam Dao, Vin Phuc, Vietnam** | **MVZ 225131** | **KU244365** |  | **KU244453** | **KU244394** | **KU244315** | **KU244353** |  | **KU244420** |
| 184 | *Theloderma corticale*_3 | Ke Go, Ha Tinh, Vietnam | ZMMU NAP-05936 | MG917765 | MG917765 |  |  |  |  |  |  |
| 185 | *Theloderma corticale*_5 | Yinggeling , Hainan, China | YGL1 | KY495637 | KY495628 |  |  |  |  |  |  |
| 186 | *Theloderma corticale*_4 | Yinggeling, Hainan, China | YGL2 | KY495638 | KY495629 |  |  |  |  |  |  |
| **187** | ***Theloderma corticale*_15** | **Tam Dao, Vin Phuc, Vietnam** | **IEBR E193.15** | **JX046476** |  |  |  |  |  |  |  |
| **188** | ***Theloderma corticale*_11** | **Tam Dao, Vin Phuc, Vietnam** | **IEBR 3267** | **JX046477** |  |  |  |  |  |  |  |
| 189 | *Theloderma corticale*_9 | Jinxiu, Guangxi, China | JXDYS2015042501 | KY290395 | KY290395 |  |  |  |  |  |  |
| 190 | *Theloderma corticale*_8 | Jinxiu, Guangxi, China | NHMG201504001 | MG322123 | MG322123 |  |  |  |  |  |  |
| 191 | *Theloderma corticale*_7 | Dayao Mountain, Guangxi, China | DYS1 | KY495635 | KY495626 |  |  |  |  |  |  |
| 192 | *Theloderma corticale*_6 | Dayao Mountain, Guangxi, China | DYS2 | KY495636 | KY495627 |  |  |  |  |  |  |
| 193 | *Theloderma corticale*_23 | Tam Dao, Vin Phuc, Vietnam | MVZ 223905 | KU244364 |  | KU244452 | KU244393 | KU244316 | KU244354 |  | KU244417 |
| 194 | *Theloderma corticale*_16 | Tam Dao, Vin Phuc, Vietnam | AMNH A161499 | DQ283050 | DQ283050 |  |  | DQ283779 | DQ282904 |  | DQ282659 |
| 195 | *Theloderma corticale*_18 | Cuc Phuong, Ninh Binh, Vietnam | corticale-1 | KT461885 | KT461885 |  |  |  |  |  |  |
| 196 | *Theloderma corticale*_17 | Cuc Phuong, Ninh Binh, Vietnam | corticale-2 | KT461886 | KT461886 |  |  |  |  |  |  |
| **197** | ***Theloderma corticale*_21** | **Bangliang, Jingxi, Guangxi, China** | **GZNU20210731005^#^** | **OP537813** | **OP537813** | **OP531850** | **OP561732** | **OP561742** | **OP561760** | **OP561744** | **OP561752** |
| 198 | *Theloderma corticale*_10 | Shangsi, Guangxi, China | NHMG20161101 | MG322126 | MG322126 |  |  |  |  |  |  |
| 199 | *Theloderma corticale*_13 | Shangsi, Guangxi, China | NHMG20161003 | MG322125 | MG322125 |  |  |  |  |  |  |
| 200 | *Theloderma corticale*_12 | Shangsi, Guangxi, China | HMG201608026 | MG322124 | MG322124 |  |  |  |  |  |  |
| 201 | *Theloderma corticale*_20 | Tuyen Quang, Vietnam | VNMN J2892 | KJ802916 | KJ802916 |  |  |  |  |  |  |
| 202 | *Theloderma corticale*_14 | Tam Dao, Vin Phuc, Vietnam | VNMN 3556 | LC012841 | LC012841 |  |  |  |  |  |  |
| 203 | *Theloderma corticale*_19 | Tam Dao, Vin Phuc, Vietnam | VNMN J2932 | KJ802917 | KJ802917 |  |  |  |  |  |  |
| **204** | ***Theloderma corticale*_22** | **Bangliang, Jingxi, Guangxi, China** | **GZNU20210731006^#^** | **OP537814** | **OP537814** | **OP531851** | **OP561733** | **OP561743** | **OP561761** | **OP561745** | **OP561753** |

**Table S2** Information on geographical distribution records, elevation and eight bioclimates of the genus *Theloderma* species collected in this study.

| Species | Clade | Elevation | Longitude | Latitude | Bio1 | Bio2 | Bio3 | Bio7 | Bio12 | Bio14 | Bio15 | Bio19 |
| --- | --- | --- | --- | --- | --- | --- | --- | --- | --- | --- | --- | --- |
| *T. horridum* | CladeA | 10 | 101.32325490 | 6.76183080 | 27.23400116 | 28.29400063 | 26.05866623 | 74.81741333 | 8.578000069 | 88.7321167 | 33.49200058 | 26.72533417 |
| *T. horridum* | CladeA | 10 | 100.06737400 | 6.62381600 | 27.2626667 | 28.02866554 | 26.60333252 | 50.22066498 | 8.333999634 | 57.94668579 | 33.25999832 | 27.61000061 |
| *T. horridum* | CladeA | 140 | 101.97576600 | 4.21048400 | 26.24583244 | 26.83533287 | 25.42399979 | 26.29740143 | 9.12899971 | 57.31009293 | 31.74799919 | 25.82600021 |
| *T. horridum* | CladeA | 210 | 102.04897910 | 3.12773940 | 22.94166565 | 23.51200104 | 22.32999992 | 29.05904579 | 8.58533287 | 46.76679993 | 28.11199951 | 23.08399963 |
| *T. horridum* | CladeA | 196 | 100.20000000 | 6.70000000 | 25.90716553 | 26.70733261 | 25.21999931 | 45.6673851 | 8.210999489 | 59.5207634 | 31.63999939 | 25.95999908 |
| *T. horridum* | CladeA | 409 | 101.03404999 | 5.79089681 | 24.52899933 | 25.18799973 | 23.92866707 | 38.63526535 | 8.443332672 | 51.87046051 | 29.91200066 | 24.36333275 |
| *T. horridum* | CladeA | 25 | 100.30036926 | 5.43521209 | 27.35520935 | 27.89270782 | 26.90520859 | 41.16057968 | 8.355208397 | 41.41930771 | 32.55625153 | 27.59791756 |
| *T. horridum* | CladeA | 21 | 100.50000000 | 5.40000000 | 27.57933235 | 28.13933372 | 27.14533234 | 37.58652115 | 9.078666687 | 42.45898819 | 33.18799973 | 27.78999901 |
| *T. horridum* | CladeA | 453 | 100.20000000 | 6.80000000 | 24.99916649 | 25.79266739 | 24.29533386 | 45.26979065 | 8.242333412 | 60.07381821 | 30.69599915 | 24.98333359 |
| *T. horridum* | CladeA | 200 | 110.00000000 | -1.20000000 | 27.24416733 | 27.68266678 | 26.84866714 | 25.73141861 | 7.609666824 | 33.1040802 | 31.84399986 | 27.45466614 |
| *T. horridum* | CladeA | 420 | 107.20000000 | 12.20000000 | 23.29183388 | 24.68266678 | 21.50333405 | 79.40933228 | 7.553667068 | 126.6939774 | 29.38800049 | 21.50333405 |
| *T. stellatum* | CladeA | 107 | 102.20352860 | 12.85253720 | 24.90799904 | 26.12199974 | 23.46333313 | 78.82635498 | 8.194000244 | 106.734024 | 30.95999908 | 23.58399963 |
| *T. stellatum* | CladeA | 61 | 102.16735840 | 12.79305320 | 26.35766602 | 27.52866745 | 25.00600052 | 82.30690765 | 8.344666481 | 101.2303162 | 32.38399887 | 25.12199974 |
| *T. stellatum* | CladeA | 10 | 101.22687730 | 14.20383870 | 27.86783218 | 29.5019989 | 25.89333344 | 81.05897522 | 10.16833305 | 143.3358459 | 35.63199997 | 26.18133354 |
| *T. stellatum* | CladeA | 351 | 101.32827759 | 14.33757350 | 25.00883293 | 26.7266655 | 22.58600044 | 78.01644135 | 9.520999908 | 170.1001282 | 32.25600052 | 22.7373333 |
| *T. stellatum* | CladeA | 600 | 101.90000000 | 14.50000000 | 24.87199974 | 26.8793335 | 21.8326664 | 76.95738983 | 9.660000801 | 206.3298492 | 32.67200089 | 22.30666733 |
| *T. stellatum* | CladeA | 646 | 99.40000000 | 12.90000000 | 24.35733414 | 25.7859993 | 22.90066719 | 75.02606964 | 8.18066597 | 112.3635254 | 30.73999977 | 23.12400055 |
| *T. stellatum* | CladeA | 599 | 104.80000000 | 18.00000000 | 23.53866577 | 26.451334 | 19.19199944 | 111.9380646 | 7.976666927 | 304.1686401 | 30.20400047 | 19.68066597 |
| *T. stellatum* | CladeA | 548 | 108.40000000 | 11.50000000 | 20.61100006 | 21.7966671 | 19.00133324 | 66.17259979 | 8.927332878 | 111.6678467 | 27.01199913 | 19.00133324 |
| *T. stellatum* | CladeA | 675 | 106.30000000 | 17.00000000 | 22.11750031 | 24.82133293 | 18.36533356 | 82.88746643 | 7.109000206 | 271.0153809 | 28.6439991 | 18.36533356 |
| *T. stellatum* | CladeA | 115 | 107.40000000 | 11.40000000 | 25.63066673 | 26.99333382 | 24.05200005 | 78.78046417 | 7.406666756 | 117.0269928 | 31.59600067 | 24.8113327 |
| *T. stellatum* | CladeA | 180 | 102.10000000 | 15.00000000 | 27.20233345 | 29.3939991 | 24.03533363 | 80.03607941 | 10.53466702 | 218.4203796 | 35.89599991 | 24.58200073 |
| *T. stellatum* | CladeA | 188 | 107.70000000 | 12.90000000 | 25.61066628 | 27.32533264 | 23.29599953 | 85.50288391 | 7.497333527 | 164.7248993 | 32.19599915 | 24.70533371 |
| *T. stellatum* | CladeA | 594 | 108.50000000 | 14.30000000 | 21.65166664 | 23.74333382 | 18.78000069 | 71.10610962 | 7.191999912 | 207.3657532 | 27.92000008 | 19.7733326 |
| *T. stellatum* | CladeA | 559 | 108.47007751 | 14.30696950 | 22.58550072 | 24.76600075 | 19.60933304 | 70.52961731 | 7.087666035 | 214.7968597 | 28.88800049 | 20.60933304 |
| *T. stellatum* | CladeA | 145 | 108.70000000 | 13.10000000 | 26.24349976 | 28.19933319 | 23.58600044 | 79.14130402 | 7.554333687 | 195.7501373 | 32.4280014 | 24.6206665 |
| *T. stellatum* | CladeA | 565 | 107.10000000 | 12.30000000 | 23.20166588 | 24.61333275 | 21.43266678 | 80.83960724 | 7.730000019 | 127.8725891 | 29.44000053 | 21.43266678 |
| *T. stellatum* | CladeA | 160 | 107.70000000 | 11.10000000 | 25.89900017 | 27.25066757 | 24.45866776 | 80.93027496 | 7.620000362 | 110.0543213 | 32.07600021 | 25.17399979 |
| *T. vietnamense* | CladeA | 110 | 107.42722220 | 11.42861111 | 25.47583389 | 26.85733414 | 23.85933304 | 78.3979187 | 7.478332996 | 118.6721954 | 31.47599983 | 24.6186676 |
| *T. vietnamense* | CladeA | 52 | 99.81967700 | 17.04540140 | 27.71433258 | 30.13000107 | 24.7373333 | 84.86113739 | 10.78533363 | 219.1659088 | 37.51200104 | 24.88400078 |
| *T. vietnamense* | CladeA | 162 | 104.76868680 | 16.57205260 | 26.38199997 | 28.81999969 | 22.95533371 | 97.47298431 | 10.06999969 | 241.8641357 | 35.01200104 | 22.95533371 |
| *T. vietnamense* | CladeA | 168 | 109.09287640 | 13.08818610 | 25.42200089 | 27.6060009 | 22.54133415 | 92.71575928 | 6.741999626 | 211.8509827 | 31.44799995 | 23.36733246 |
| *T. vietnamense* | CladeA | 31 | 108.00659180 | 11.11372728 | 25.01533318 | 26.298666 | 23.67133331 | 75.325737 | 7.690667152 | 102.7721252 | 30.97599983 | 24.28066635 |
| *T. vietnamense* | CladeA | 52 | 103.98402000 | 10.28987900 | 27.0063343 | 28.06599998 | 26.12000084 | 80.35042572 | 6.892000198 | 79.07992554 | 32.41600037 | 26.12000084 |
| *T. vietnamense* | CladeA | 218 | 107.44994040 | 11.40573700 | 25.57016754 | 26.92399979 | 24.01866722 | 78.23604584 | 7.634333611 | 115.5471725 | 31.68799973 | 24.7733326 |
| *T. vietnamense* | CladeA | 12 | 103.74336920 | 11.11760780 | 27.70890236 | 28.98333359 | 26.77348518 | 84.84500885 | 7.59734869 | 88.87506866 | 33.83181763 | 26.84621239 |
| *T. vietnamense* | CladeA | 26 | 105.89949560 | 11.60076220 | 26.87700081 | 28.39666748 | 25.18333435 | 76.35905457 | 7.545333385 | 130.6651459 | 33.20400238 | 25.18333435 |
| *T. vietnamense* | CladeA | 165 | 107.01919560 | 12.90284370 | 25.90716743 | 27.4746666 | 23.79399872 | 89.83220673 | 7.644999981 | 151.0318146 | 32.18000031 | 23.98266602 |
| *T. vietnamense* | CladeA | 75 | 107.00000000 | 11.30000000 | 25.826334 | 27.30533218 | 24.09666634 | 79.47102356 | 6.953333378 | 127.430397 | 31.63999939 | 24.91133308 |
| *T. vietnamense* | CladeA | 80 | 107.00000000 | 11.20000000 | 25.85766602 | 27.30599976 | 24.19866753 | 79.65805817 | 6.789333344 | 124.0689468 | 31.61199951 | 25.00533295 |
| *T. vietnamense* | CladeA | 115 | 107.40000000 | 11.40000000 | 25.63066673 | 26.99333382 | 24.05200005 | 78.78046417 | 7.406666756 | 117.0269928 | 31.59600067 | 24.8113327 |
| *T. vietnamense* | CladeA | 498 | 107.10000000 | 12.50000000 | 23.81666756 | 25.31733322 | 21.89799881 | 83.83470917 | 7.680666924 | 140.2732086 | 30.19599915 | 21.94199944 |
| *T. vietnamense* | CladeA | 156 | 107.40000000 | 11.50000000 | 25.52283287 | 26.88333321 | 23.92066574 | 77.78798676 | 7.684333801 | 117.7279434 | 31.63999939 | 24.67866707 |
| *T. vietnamense* | CladeA | 115 | 107.40000000 | 11.40000000 | 25.63066673 | 26.99333382 | 24.05200005 | 78.78046417 | 7.406666756 | 117.0269928 | 31.59600067 | 24.8113327 |
| *T. vietnamense* | CladeA | 565 | 107.10000000 | 12.30000000 | 23.20166588 | 24.61333275 | 21.43266678 | 80.83960724 | 7.730000019 | 127.8725891 | 29.44000053 | 21.43266678 |
| *T. vietnamense* | CladeA | 160 | 106.60000000 | 8.70000000 | 26.60856438 | 27.77407455 | 25.22870255 | 77.65563202 | 5.171759129 | 99.28178406 | 31.08333397 | 25.582407 |
| *T. vietnamense* | CladeA | 100 | 107.10000000 | 11.40000000 | 25.50883293 | 26.95666695 | 23.77000046 | 79.32261658 | 6.942333221 | 126.0534744 | 31.27600098 | 24.53866577 |
| *T. moloch* | CladeB | 1330 | 97.58726077 | 24.61371105 | 17.83699989 | 21.73333359 | 12.14866638 | 90.14546204 | 10.30000019 | 400.2759094 | 25.71999931 | 12.14866638 |
| *T. moloch* | CladeB | 1103 | 95.33304000 | 29.32520990 | 15.7755003 | 21.98866653 | 8.066666603 | 88.16471863 | 12.07299995 | 575.6551514 | 27.7159996 | 9.489333153 |
| *T. moloch* | CladeB | 2071 | 94.50439453 | 28.36723539 | 14.93516636 | 20.24533272 | 8.541999817 | 86.72284698 | 9.157666206 | 479.2541504 | 24.61199951 | 9.684000015 |
| *T. moloch* | CladeB | 572 | 95.80000000 | 28.20000000 | 20.67650032 | 25.7033329 | 14.32733345 | 84.80912781 | 9.26633358 | 469.0672302 | 29.79599953 | 15.49600029 |
| *T. moloch* | CladeB | 497 | 92.40000000 | 27.00000000 | 20.35000038 | 24.75933456 | 14.51599979 | 96.78296661 | 8.264666557 | 420.4473877 | 28.43200111 | 15.53933334 |
| *T. phrynoderma* | CladeB | 474 | 99.35485840 | 12.21118019 | 25.39049911 | 26.66066742 | 24.13666725 | 69.67185211 | 7.650333405 | 101.6906967 | 31.31200027 | 24.13666725 |
| *T. phrynoderma* | CladeB | 297 | 98.98132324 | 10.53777073 | 26.17983437 | 27.3793335 | 24.98999977 | 63.68054581 | 8.207000732 | 93.34588623 | 32.58000183 | 25.1673336 |
| *T. phrynoderma* | CladeB | 462 | 98.19717407 | 14.69120976 | 26.35066605 | 28.18666649 | 25.03466797 | 107.2703476 | 10.39333344 | 130.2042084 | 35.25999832 | 25.08733368 |
| *T. ryabovi* | CladeB | 1423 | 108.19000000 | 14.69027778 | 19.33950043 | 21.20400047 | 16.71133423 | 81.30268097 | 7.26233387 | 188.1047668 | 25.27599907 | 16.71133423 |
| *T. ryabovi* | CladeB | 1138 | 108.32527778 | 14.69027778 | 20.04999924 | 22.04999924 | 17.28866577 | 74.38619995 | 7.090666771 | 198.4642639 | 26.03999901 | 17.28866577 |
| *T. licin* | CladeB | 672 | 102.91168210 | 3.99030068 | 23.57566643 | 24.28533363 | 22.61466599 | 39.43559647 | 7.908666611 | 65.36776733 | 28.81200027 | 23.92733383 |
| *T. licin* | CladeB | 1173 | 99.69406128 | 8.47508888 | 22.61416626 | 23.51799965 | 21.75333405 | 46.53990555 | 8.528999329 | 71.78983307 | 28.61599922 | 22.45933342 |
| *T. licin* | CladeB | 957 | 113.92132700 | -0.78927500 | 26.99116707 | 27.2840004 | 26.75666618 | 20.60775566 | 9.328999519 | 23.73940849 | 32.13599777 | 26.91666603 |
| *T. licin* | CladeB | 613 | 101.75537110 | 3.34617969 | 22.59083366 | 23.18533325 | 21.92266655 | 28.62377167 | 8.815666199 | 50.03639221 | 27.8920002 | 22.79599953 |
| *T. licin* | CladeB | 463 | 102.7427778 | 3.67 | 24.04599953 | 24.71666718 | 23.21533394 | 34.77093506 | 8.098666191 | 58.86027145 | 29.25200081 | 24.30666733 |
| *T. licin* | CladeB | 556 | 100.9543991 | 5.85203465 | 25.63716698 | 26.30133438 | 25.04266739 | 41.34152603 | 8.503000259 | 51.53882217 | 31.05599976 | 25.55533218 |
| *T. licin* | CladeB | 1434 | 102.1234131 | 3.76559677 | 18.96416664 | 19.66066742 | 17.99533272 | 35.30921936 | 8.569000244 | 65.86820984 | 24.36800003 | 19.34066582 |
| *T. licin* | CladeB | 257 | 100.9575 | 6.01027778 | 24.85000038 | 25.53000069 | 24.20933342 | 40.96591187 | 8.255332947 | 53.85077667 | 30.21199989 | 24.70066643 |
| *T. licin* | CladeB | 612 | 99.71225739 | 8.69948101 | 25.71583366 | 26.78333282 | 24.54199982 | 55.52292252 | 8.454333305 | 90.11226654 | 31.82800102 | 25.26533318 |
| *T. licin* | CladeB | 340 | 100.5212975 | 7.01894958 | 27.13966751 | 28.00799942 | 26.15133286 | 71.28604126 | 8.839333534 | 75.0085907 | 33.33599854 | 27.01600075 |
| *T. licin* | CladeB | 637 | 101.9294357 | 5.79055524 | 25.65100098 | 26.5233345 | 24.6313324 | 51.71935654 | 8.03666687 | 74.01418304 | 30.99600029 | 25.74733353 |
| *T. licin* | CladeB | 411 | 102.00000000 | 5.70000000 | 25.85233307 | 26.72200012 | 24.79933357 | 52.61396408 | 7.912666798 | 75.00798798 | 31.17200089 | 25.93066597 |
| *T. licin* | CladeB | 992 | 114.90000000 | 4.10000000 | 22.80466652 | 23.21733284 | 22.46533394 | 16.38699913 | 7.720666885 | 30.27105522 | 27.30399895 | 22.94799995 |
| *T. licin* | CladeB | 453 | 100.20000000 | 6.80000000 | 24.99916649 | 25.79266739 | 24.29533386 | 45.26979065 | 8.242333412 | 60.07381821 | 30.69599915 | 24.98333359 |
| *T. licin* | CladeB | 1608 | 99.60000000 | 0.80000000 | 24.2341671 | 24.67933273 | 23.95666695 | 22.59000587 | 9.201000214 | 30.71553802 | 29.4279995 | 24.16600037 |
| *T. licin* | CladeB | 2123 | 114.92660522 | 4.04852199 | 20.22816658 | 20.59666634 | 19.92200089 | 16.50902176 | 7.753666878 | 27.85398102 | 24.68000031 | 20.31533241 |
| *T. licin* | CladeB | 1438 | 96.09020233 | 4.95585260 | 21.56433296 | 22.07933426 | 21.15999985 | 19.56022453 | 8.717999458 | 38.06508636 | 26.79599953 | 21.6079998 |
| *T. licin* | CladeB | 402 | 101.80000000 | 5.80000000 | 24.96266747 | 25.84066772 | 23.96333313 | 48.47255325 | 7.876000404 | 73.355896 | 30.23200035 | 25.08799934 |
| *T. licin* | CladeB | 402 | 100.70000000 | -0.10000000 | 22.50883293 | 22.93799973 | 22.24799919 | 30.29007339 | 8.572333336 | 29.66828156 | 27.44400024 | 22.42933273 |
| *T. asperum* | CladeB | 1005 | 100.99182130 | 5.27147784 | 21.86733246 | 22.49399948 | 21.28133392 | 29.90926361 | 8.453333855 | 50.07785797 | 27.13999939 | 21.65200043 |
| *T. asperum* | CladeB | 1232 | 101.73127840 | 3.71816670 | 20.96100044 | 21.60866737 | 20.11199951 | 32.02598572 | 8.766666412 | 58.96518326 | 26.28800011 | 21.27666664 |
| *T. asperum* | CladeB | 1070 | 101.29943850 | 4.71877755 | 19.36650085 | 20.02866745 | 18.63533401 | 28.93980408 | 8.853000641 | 54.45310974 | 24.68799973 | 19.64133263 |
| *T. asperum* | CladeB | 866 | 102.30000000 | 22.10000000 | 20.79816628 | 24.42666626 | 15.57199955 | 87.56122589 | 9.537667274 | 371.8920593 | 28.78800011 | 15.57199955 |
| *T. asperum* | CladeB | 425 | 106.10000000 | 17.00000000 | 24.08416748 | 26.82066727 | 20.19466591 | 84.9445343 | 7.548333168 | 278.5057068 | 30.90800095 | 21.39266777 |
| *T. asperum* | CladeB | 717 | 104.80000000 | 18.90000000 | 22.06816673 | 26.201334 | 16.88066673 | 87.72197723 | 7.212332726 | 382.2458801 | 29.95999908 | 16.88066673 |
| *T. asperum* | CladeB | 1000 | 105.60000000 | 21.50000000 | 19.89216614 | 24.88733292 | 13.70400047 | 91.67219543 | 6.569666862 | 463.955719 | 28.28800011 | 14.84733295 |
| *T. pyaukkya* | CladeB | 1736 | 94.00000000 | 21.60000000 | 22.70166588 | 25.9766674 | 17.91133308 | 94.44112396 | 9.017333031 | 335.5471191 | 32.31200027 | 20.05999947 |
| *T. pyaukkya* | CladeB | 856 | 96.59729004 | 25.22357783 | 20.70316696 | 24.24733353 | 15.11866665 | 96.47982788 | 9.513666153 | 384.259552 | 28.36400032 | 15.11866665 |
| *T. pyaukkya* | CladeB | 1134 | 97.83290863 | 27.30589255 | 18.01250076 | 22.95000076 | 11.73933315 | 97.49261475 | 8.501667023 | 464.2528381 | 26.63199997 | 13.01399994 |
| *T. pyaukkya* | CladeB | 411 | 97.58531889 | 24.71065585 | 21.05699921 | 24.65600014 | 15.37733269 | 97.32682037 | 10.35866642 | 393.0270691 | 29.25600052 | 15.37733269 |
| *T. pyaukkya* | CladeB | 697 | 97.47553800 | 27.16010800 | 19.59333229 | 24.4533329 | 13.22933292 | 106.0808258 | 8.220666885 | 464.7844543 | 27.79599953 | 14.59933281 |
| *T. pyaukkya* | CladeB | 823 | 93.58385800 | 21.30496800 | 18.63349915 | 21.18400002 | 14.51133347 | 102.1899643 | 8.468333244 | 282.6416626 | 26.84399986 | 16.29133224 |
| *T. pyaukkya* | CladeB | 266 | 96.24225000 | 25.05531800 | 23.74716759 | 27.32400131 | 17.78066635 | 96.6616745 | 10.17766666 | 404.6452942 | 32.32400131 | 19.50266647 |
| *T. pyaukkya* | CladeB | 456 | 97.41312800 | 27.17233800 | 18.89800072 | 23.80466652 | 12.51600075 | 104.2205582 | 8.420666695 | 467.7770691 | 27.21999931 | 13.88533306 |
| *T. pyaukkya* | CladeB | 1291 | 93.58236800 | 21.33529800 | 18.82583237 | 21.37133408 | 14.75799942 | 103.097374 | 8.322999954 | 279.3532715 | 26.98800087 | 16.54800034 |
| *T. pyaukkya* | CladeB | 1338 | 93.58385800 | 21.30496800 | 18.63349915 | 21.18400002 | 14.51133347 | 102.1899643 | 8.468333244 | 282.6416626 | 26.84399986 | 16.29133224 |
| *T. baibungense* | CladeB | 1209 | 92.98381805 | 23.17413570 | 21.23483276 | 23.89266586 | 16.28733253 | 88.42150879 | 9.130999565 | 328.6091309 | 29.16399956 | 16.28733253 |
| *T. baibungense* | CladeB | 2805 | 95.10119256 | 29.10705888 | 10.48533344 | 16.43933296 | 3.594000101 | 87.27667999 | 10.66266632 | 532.0108643 | 21.76000023 | 4.907999992 |
| *T. petilum* | CladeB | 1379 | 103.72166670 | 19.86055560 | 19.40633392 | 22.69000053 | 14.71466637 | 93.20374298 | 8.39133358 | 334.3850098 | 26.26399994 | 15.15400028 |
| *T. petilum* | CladeB | 1082 | 102.57570280 | 22.08326380 | 19.85533333 | 23.41799927 | 14.78199959 | 94.49419403 | 9.248666763 | 362.8891296 | 27.55999947 | 14.78199959 |
| *T. petilum* | CladeB | 586 | 102.13611111 | 22.09555556 | 20.86366653 | 24.46333313 | 15.66533375 | 81.36432648 | 9.611999512 | 369.615509 | 28.87199974 | 15.66533375 |
| *T. petilum* | CladeB | 920 | 100.93333333 | 17.54333333 | 23.23566628 | 25.50200081 | 19.95199966 | 80.21121979 | 11.29266644 | 230.7500153 | 32.4960022 | 20.09733391 |
| *T. albopunctatum* | CladeB | 1061 | 102.14246750 | 22.11633618 | 20.86366653 | 24.46333313 | 15.66533375 | 81.36432648 | 9.611999512 | 369.615509 | 28.87199974 | 15.66533375 |
| *T. albopunctatum* | CladeB | 644 | 105.19821170 | 19.88039177 | 20.64550018 | 25.17666626 | 15.15266609 | 89.63700104 | 6.600999832 | 412.4495544 | 28.72800064 | 15.15266609 |
| *T. albopunctatum* | CladeB | 795 | 105.37261960 | 21.05374449 | 21.15633392 | 26.0953331 | 15.19199944 | 91.95333099 | 6.513333797 | 452.7104187 | 29.64800072 | 15.19199944 |
| *T. albopunctatum* | CladeB | 1675 | 99.57305564 | 19.82388891 | 21.48533249 | 24.07733345 | 17.8113327 | 83.63480377 | 11.43066692 | 263.8186646 | 31.45599937 | 17.82600021 |
| *T. albopunctatum* | CladeB | 601 | 94.57031250 | 18.83351540 | 23.64900017 | 26.13466644 | 19.7033329 | 114.8922043 | 9.720666885 | 261.2600403 | 32.15599823 | 20.8686676 |
| *T. albopunctatum* | CladeB | 313 | 104.26025390 | 16.63619188 | 25.18566704 | 27.5933342 | 21.70599937 | 97.2986145 | 9.786000252 | 242.5959473 | 33.86800003 | 21.70599937 |
| *T. albopunctatum* | CladeB | 1881 | 107.90222170 | 15.02570678 | 15.28149986 | 17.01600075 | 12.91266727 | 89.27477264 | 7.927666664 | 170.4957123 | 21.43600082 | 12.91266727 |
| *T. albopunctatum* | CladeB | 1577 | 108.40000000 | 14.31666700 | 18.30183411 | 20.11800003 | 15.76333332 | 79.23459625 | 7.138333321 | 180.5055237 | 24.19199944 | 15.76333332 |
| *T. albopunctatum* | CladeB | 438 | 104.41612240 | 16.86237671 | 24.68300056 | 27.07133293 | 21.20533371 | 99.72036743 | 9.402000427 | 243.2592773 | 33 | 21.20533371 |
| *T. albopunctatum* | CladeB | 815 | 101.50560510 | 17.36222340 | 22.78149986 | 25.05133438 | 19.41133308 | 80.43390656 | 10.87233353 | 235.0700836 | 31.63199997 | 19.41133308 |
| *T. albopunctatum* | CladeB | 838 | 101.57371920 | 16.73995360 | 22.85400009 | 25.0953331 | 19.57400131 | 81.1452179 | 10.7086668 | 228.7474213 | 31.64400101 | 19.57400131 |
| *T. albopunctatum* | CladeB | 950 | 102.62162110 | 20.06562290 | 21.62583351 | 24.38599968 | 17.36000061 | 98.13362122 | 9.694333076 | 302.5080566 | 29.20400047 | 17.57533264 |
| *T. albopunctatum* | CladeB | 800 | 100.77758790 | 19.41997316 | 22.89183235 | 25.22800064 | 19.37866592 | 87.19272614 | 10.99699974 | 251.6308441 | 31.63599968 | 19.37866592 |
| *T. albopunctatum* | CladeB | 1420 | 102.32666020 | 19.88814027 | 21.51616669 | 24.13333321 | 17.44199944 | 94.91524506 | 10.06900024 | 290.1993103 | 29.45999908 | 17.5593338 |
| *T. albopunctatum* | CladeB | 1419 | 101.81304930 | 19.84939396 | 21.54766655 | 24.05533218 | 17.68733215 | 90.26268005 | 10.58399963 | 276.2854614 | 29.77599907 | 17.78733253 |
| *T. albopunctatum* | CladeB | 1201 | 108.28870450 | 14.60383510 | 20.08483315 | 21.97933197 | 17.41600037 | 78.01816559 | 7.267666817 | 190.6903076 | 26.11199951 | 17.41600037 |
| *T. albopunctatum* | CladeB | 958 | 109.07844540 | 12.37018527 | 23.2681675 | 25.00466728 | 20.85466766 | 74.59587097 | 7.180333138 | 173.4780426 | 29.05200005 | 21.56066704 |
| *T. albopunctatum* | CladeB | 1217 | 103.22648000 | 22.77965990 | 17.58716583 | 21.63733292 | 12.06933403 | 89.83654022 | 8.58433342 | 397.1625061 | 25.3920002 | 12.06933403 |
| *T. albopunctatum* | CladeB | 1721 | 102.86773680 | 22.66217531 | 13.77416706 | 17.61066628 | 8.511333466 | 88.21155548 | 8.812999725 | 377.6301575 | 21.13999939 | 9.061332703 |
| *T. albopunctatum* | CladeB | 1194 | 109.39636230 | 18.95564887 | 19.77633286 | 23.20800018 | 15.19733334 | 76.5793457 | 6.608666897 | 331.225769 | 26.50399971 | 15.19733334 |
| *T. albopunctatum* | CladeB | 1139 | 110.26702880 | 24.27701247 | 16.72333336 | 24.04600143 | 8.147999763 | 65.66957092 | 6.968000412 | 655.7416382 | 28.00799942 | 9.920000076 |
| *T. albopunctatum* | CladeB | 1354 | 110.30822750 | 24.22943332 | 16.67116737 | 23.95866585 | 8.161999702 | 65.51777649 | 6.97966671 | 651.6131592 | 27.95599937 | 9.911333084 |
| *T. albopunctatum* | CladeB | 845 | 106.49888990 | 22.93910816 | 20.26666641 | 25.96800041 | 13.09266663 | 88.77693176 | 6.877999783 | 527.569397 | 29.27199936 | 14.33599949 |
| *T. albopunctatum* | CladeB | 201 | 106.99962620 | 20.82191278 | 22.75566673 | 27.72733307 | 16.72400093 | 99.71750641 | 5.585333347 | 458.9318848 | 30.70000076 | 18.04933357 |
| *T. albopunctatum* | CladeB | 1451 | 108.20434570 | 14.62079358 | 19.80916595 | 21.65533257 | 17.2733326 | 83.34222412 | 7.347000122 | 181.4679108 | 25.79999924 | 17.2733326 |
| *T. albopunctatum* | CladeB | 495 | 105.72143560 | 17.94738068 | 23.15800095 | 26.81933403 | 18.38466644 | 73.65847015 | 6.870000362 | 348.1958923 | 30.62800026 | 19.59133339 |
| *T. albopunctatum* | CladeB | 606 | 105.28010450 | 22.15565500 | 23.00733376 | 28.28533363 | 16.60133362 | 89.66104889 | 7.674666882 | 486.4314575 | 32.2480011 | 17.7159996 |
| *T. albopunctatum* | CladeB | 289 | 105.50445560 | 18.76266324 | 23.94783401 | 28.8579998 | 18.32799911 | 84.99729156 | 6.734333038 | 429.9640198 | 32.92399979 | 19.03333282 |
| *T. albopunctatum* | CladeB | 289 | 105.58616640 | 18.71519514 | 24.33483315 | 29.32266617 | 18.72999954 | 84.67870331 | 6.673666477 | 432.4954529 | 33.42799759 | 19.35466576 |
| *T. albopunctatum* | CladeB | 2348 | 103.69995120 | 22.62922252 | 16.39633369 | 20.86400032 | 10.46933365 | 83.99384308 | 7.841333389 | 427.6927795 | 24.29599953 | 11.24733353 |
| *T. leporosum* | CladeC | 458 | 102.18109130 | 3.79026229 | 23.89183426 | 24.52799988 | 23.0233326 | 27.91719246 | 8.456999779 | 60.05183792 | 29.1760006 | 24.15933228 |
| *T. leporosum* | CladeC | 474 | 101.60842900 | 3.24746639 | 26.27266693 | 26.73066711 | 25.79466629 | 28.22306252 | 9.017333031 | 36.53009033 | 31.44400024 | 26.41333389 |
| *T. leporosum* | CladeC | 356 | 102.06161500 | 2.70506445 | 24.0296669 | 24.52866745 | 23.59399986 | 26.40447807 | 8.378666878 | 38.26652145 | 29.0720005 | 24.07066727 |
| *T. gordoni* | CladeC | 1588 | 98.89194400 | 18.81638900 | 21.67583275 | 24.10599899 | 18.23666763 | 82.22180176 | 10.77566624 | 245.158493 | 31.2159996 | 18.23666763 |
| *T. gordoni* | CladeC | 976 | 107.71820070 | 14.46925596 | 20.67749977 | 22.39866638 | 18.47399902 | 97.68873596 | 7.380999565 | 159.5836487 | 26.73600006 | 18.47399902 |
| *T. gordoni* | CladeC | 638 | 104.91119390 | 19.31373538 | 21.64633369 | 26.01399994 | 16.25466728 | 85.07991028 | 7.052000046 | 399.2886658 | 29.73200035 | 16.25466728 |
| *T. gordoni* | CladeC | 1565 | 103.84277340 | 21.18569224 | 18.58433342 | 22.32466698 | 13.47733307 | 94.92980194 | 8.603333473 | 368.354248 | 25.97200012 | 14.02266598 |
| *T. gordoni* | CladeC | 758 | 100.89699000 | 22.08657046 | 21.80983353 | 25.14733315 | 16.83466721 | 78.06208801 | 11.40433311 | 352.1237488 | 31.32799911 | 18.44599915 |
| *T. gordoni* | CladeC | 1559 | 101.08498810 | 22.67387575 | 18.05666733 | 21.61066628 | 13.00399971 | 85.34770203 | 10.82933331 | 360.3581848 | 26.8560009 | 14.52400017 |
| *T. gordoni* | CladeC | 1277 | 104.10013260 | 20.32541750 | 18.64666748 | 22.28466606 | 13.72733307 | 89.86976624 | 8.191333771 | 355.1020813 | 25.88800049 | 14.29933357 |
| *T. gordoni* | CladeC | 888 | 105.64418520 | 21.45323020 | 21.33466721 | 26.36199951 | 15.16266632 | 89.41149902 | 6.708666801 | 466.4740906 | 29.94000053 | 15.16266632 |
| *T. gordoni* | CladeC | 1456 | 98.50000000 | 18.50000000 | 19.9386673 | 22.34133339 | 16.56066704 | 81.10469055 | 11.04533291 | 238.6944733 | 29.61199951 | 16.56066704 |
| *T. gordoni* | CladeC | 2606 | 103.80000000 | 22.30000000 | 14.65866661 | 18.88400078 | 8.975999832 | 87.2206955 | 7.948000431 | 408.3772888 | 22.21999931 | 9.751999855 |
| *T. gordoni* | CladeC | 1000 | 105.60000000 | 21.50000000 | 19.89216614 | 24.88733292 | 13.70400047 | 91.67219543 | 6.569666862 | 463.955719 | 28.28800011 | 14.84733295 |
| *T. gordoni* | CladeC | 594 | 108.50000000 | 14.30000000 | 21.65166664 | 23.74333382 | 18.78000069 | 71.10610962 | 7.191999912 | 207.3657532 | 27.92000008 | 19.7733326 |
| *T. gordoni* | CladeC | 495 | 105.40000000 | 22.40000000 | 22.0878334 | 27.28933334 | 15.69866657 | 91.89775848 | 7.269666672 | 480.3213196 | 31.13999939 | 15.69866657 |
| *T. gordoni* | CladeC | 1887 | 104.70000000 | 19.70000000 | 17.67733383 | 21.32999992 | 12.85000038 | 92.96478271 | 7.914000034 | 349.8643188 | 24.9279995 | 13.42400074 |
| *T. lacustrinum* | CladeC | 326.5 | 102.11791990 | 18.92707243 | 23.43933296 | 25.80400085 | 19.67266655 | 94.57468414 | 10.04466724 | 264.056366 | 31.36400032 | 19.67266655 |
| *T. lacustrinum* | CladeC | 423 | 101.9723511 | 18.71909713 | 24.20416641 | 26.58333206 | 20.47066689 | 93.74971008 | 10.32033253 | 261.4307251 | 32.41199875 | 20.47066689 |
| *T. lateriticum* | CladeC | 1332 | 104.35000000 | 21.90000000 | 17.67200089 | 22.21466637 | 11.82333374 | 93.82143402 | 7.872000217 | 427.4062195 | 25.76799965 | 12.67000008 |
| *T. lateriticum* | CladeC | 1306 | 103.80432130 | 22.48718182 | 17.52116585 | 22.05599976 | 11.5340004 | 85.60071564 | 7.937000275 | 433.2267456 | 25.57600021 | 12.3706665 |
| *T. lateriticum* | CladeC | 1130 | 104.46650720 | 21.27882820 | 19.12249947 | 23.40866661 | 13.65266609 | 92.06616211 | 8.268333435 | 402.3286743 | 27.18400002 | 14.48066616 |
| *T. lateriticum* | CladeC | 849 | 106.49511340 | 22.92661876 | 20.26666641 | 25.96800041 | 13.09266663 | 88.77693176 | 6.877999783 | 527.569397 | 29.27199936 | 14.33599949 |
| *T. lateriticum* | CladeC | 510 | 107.89532690 | 21.84124157 | 19.47383308 | 25.03066635 | 12.59333324 | 82.82811737 | 6.11433363 | 514.1681519 | 28.19599915 | 13.97133255 |
| *T. lateriticum* | CladeC | 225 | 106.23144150 | 21.21402006 | 23.38949966 | 28.57733345 | 17.13733292 | 85.59860992 | 6.501000404 | 474.912323 | 32.12799835 | 17.13733292 |
| *T. lateriticum* | CladeC | 977 | 105.68023682 | 21.44795596 | 20.85050011 | 25.87800026 | 14.6746664 | 90.36290741 | 6.645666122 | 466.1691895 | 29.37599945 | 15.85666656 |
| *T. lateriticum* | CladeC | 1126 | 105.9 | 22.6 | 18.35549927 | 23.64466667 | 11.70399952 | 92.25976563 | 6.93900013 | 489.5769653 | 27.03199959 | 12.76200008 |
| *T. nebulosum* | CladeC | 1775 | 107.86000000 | 15.06000000 | 16.81966591 | 18.61199951 | 14.32999992 | 90.75434113 | 7.724666595 | 178.291748 | 22.8920002 | 14.32999992 |
| *T. nebulosum* | CladeC | 1836 | 107.96000000 | 15.08000000 | 15.06750011 | 16.92133331 | 12.55000019 | 86.69205475 | 7.883000374 | 183.7946167 | 21.2840004 | 12.55000019 |
| *T. nebulosum* | CladeC | 2062 | 108 | 15.1 | 17.93000031 | 19.93933296 | 15.22266674 | 82.52843475 | 7.697333336 | 198.9086304 | 24.21199989 | 15.22266674 |
| *T. nebulosum* | CladeC | 1772 | 107.9 | 15.1 | 16.70666695 | 18.57666779 | 14.13333321 | 88.28035736 | 7.759333611 | 186.0744019 | 22.84399986 | 14.13333321 |
| *T. annae* | CladeC | 415 | 105.42686460 | 20.42701281 | 23.15433311 | 28.1906662 | 17.16666603 | 91.99673462 | 6.588666439 | 456.1766968 | 31.91200066 | 17.16666603 |
| *T. annae* | CladeC | 301 | 105.31836670 | 20.41515000 | 22.21666718 | 27.06599998 | 16.38333321 | 93.63704681 | 6.50666666 | 442.5794067 | 30.70400047 | 16.38333321 |
| *T. annae* | CladeC | 549 | 105.2874756 | 20.49327568 | 20.55550003 | 25.25533295 | 14.85733318 | 94.69343567 | 6.664332867 | 429.3752747 | 28.79199982 | 14.85733318 |
| *T. truongsonense* | CladeC | 451 | 106.58333330 | 16.91666667 | 22.56383324 | 25.41400146 | 18.73399925 | 77.59553528 | 7.161666393 | 278.4729004 | 29.24399948 | 19.80066681 |
| *T. truongsonense* | CladeC | 762 | 109.09355160 | 12.36616101 | 22.81533241 | 24.51000023 | 20.45000076 | 71.98685455 | 7.121999741 | 169.8279114 | 28.49200058 | 21.12199974 |
| *T. truongsonense* | CladeC | 762 | 109.19917300 | 12.26549100 | 26.83068085 | 28.65151596 | 24.46742439 | 94.80484772 | 6.468938828 | 174.5518188 | 32.28181839 | 25.08787918 |
| *T. truongsonense* | CladeC | 637 | 106.35864258 | 17.45547258 | 22.82600021 | 26.14666748 | 18.44599915 | 76.50322723 | 6.60733366 | 319.3294678 | 29.68799973 | 19.56333351 |
| *T. truongsonense* | CladeC | 345 | 106.27761840 | 17.70290339 | 24.48150063 | 28.38199997 | 19.62599945 | 79.2074585 | 6.661000252 | 359.0560913 | 32.30400085 | 22.94400024 |
| *T. truongsonense* | CladeC | 1667 | 108.38287350 | 14.32094141 | 18.30183411 | 20.11800003 | 15.76333332 | 79.23459625 | 7.138333321 | 180.5055237 | 24.19199944 | 15.76333332 |
| *T. truongsonense* | CladeC | 1642 | 107.7 | 15.3 | 19.63216591 | 21.68133354 | 16.90800095 | 89.07860565 | 7.436333179 | 200.8811646 | 25.81999969 | 16.90800095 |
| *T. laeve* | CladeC | 1486 | 108.42355330 | 12.01510820 | 16.89916611 | 18.02000046 | 15.35400009 | 66.82106781 | 9.533666611 | 108.947403 | 23.59600067 | 15.35400009 |
| *T. laeve* | CladeC | 1146 | 108.39111330 | 14.13657565 | 20.01650047 | 21.88800049 | 17.39400101 | 80.09166718 | 7.022333622 | 185.8334198 | 25.93199921 | 17.39400101 |
| *T. laeve* | CladeC | 1628 | 108.35678100 | 12.09438139 | 16.201334 | 17.38999939 | 14.49199963 | 68.30731964 | 8.695333481 | 117.5681763 | 22.52000046 | 14.49199963 |
| *T. laeve* | CladeC | 1443 | 108.41372460 | 11.96651260 | 17.43849945 | 18.55866623 | 15.90733337 | 66.93161011 | 9.717000008 | 108.211525 | 24.28000069 | 15.90733337 |
| *T. laeve* | CladeC | 1608 | 108.35266110 | 12.06215203 | 16.52983284 | 17.6886673 | 14.89333344 | 68.08317566 | 9.019000053 | 113.5384445 | 22.94799995 | 14.89333344 |
| *T. laeve* | CladeC | 1048 | 108.4 | 14.4 | 20.28149986 | 22.2179985 | 17.5359993 | 74.95240784 | 6.998333454 | 195.5203552 | 26.19199944 | 17.5359993 |
| *T. auratum* | CladeC | 1014 | 108.54166670 | 14.50547222 | 20.93966675 | 23.05400085 | 18.01866722 | 68.13842773 | 6.750666618 | 209.4551392 | 26.89599991 | 18.9746666 |
| *T. auratum* | CladeC | 1426 | 108.33147220 | 14.21744444 | 19.44499969 | 21.24466705 | 16.96333313 | 84.02443695 | 7.102666855 | 175.4147186 | 25.36800003 | 16.96333313 |
| *T. auratum* | CladeC | 1219 | 108.30125000 | 14.72566667 | 20.09883308 | 22.16600037 | 17.28933334 | 73.43144226 | 6.93900013 | 202.8002014 | 26.07600021 | 17.28933334 |
| *T. palliatum* | CladeC | 447 | 108.07256340 | 12.82293230 | 24.28800011 | 26.0233326 | 22.07666588 | 79.80519104 | 8.017999649 | 161.0575256 | 31.36800003 | 23.10266685 |
| *T. palliatum* | CladeC | 1638 | 108.71510000 | 12.18650000 | 17.33716583 | 18.69666672 | 15.29933357 | 60.1472168 | 7.91166687 | 140.6358337 | 23.20000076 | 15.98266697 |
| *T. palliatum* | CladeC | 1538 | 108.70000000 | 12.20000000 | 16.88366699 | 18.22933388 | 14.84933376 | 62.28561401 | 8.212666512 | 139.1298676 | 22.93199921 | 14.84933376 |
| *T. rhododiscus* | CladeC | 486 | 107.98289000 | 25.05795000 | 18.01350021 | 25.7733326 | 9.110666275 | 68.94277191 | 7.473666668 | 682.335083 | 30.21199989 | 9.110666275 |
| *T. rhododiscus* | CladeC | 909 | 105.22705080 | 24.32707654 | 18.56383324 | 24.36533356 | 11.17399979 | 84.07853699 | 8.719666481 | 539.3775635 | 28.65200043 | 11.17399979 |
| *T. rhododiscus* | CladeC | 667 | 110.32303900 | 24.03264600 | 18.78633308 | 25.9640007 | 10.3253336 | 65.12729645 | 7.28000021 | 647.194458 | 30.06800079 | 12.13133335 |
| *T. rhododiscus* | CladeC | 725 | 110.25192260 | 24.23945147 | 17.09733391 | 24.38866806 | 8.526000023 | 65.6823349 | 7.075333595 | 654.4470825 | 28.41200066 | 10.29266644 |
| *T. rhododiscus* | CladeC | 1136 | 110.11707300 | 23.97022400 | 15.67416668 | 22.60533333 | 7.524000168 | 65.76712036 | 6.812999725 | 621.7077026 | 26.40399933 | 9.248666763 |
| *T. rhododiscus* | CladeC | 214 | 110.77906232 | 23.78535209 | 20.72266579 | 27.75466728 | 12.32400036 | 66.03482056 | 7.88866663 | 638.5595703 | 32.13600159 | 14.11200047 |
| *T. rhododiscus* | CladeC | 757 | 110.1 | 23.9 | 17.46616745 | 24.41666603 | 9.200666428 | 65.60858917 | 7.031666756 | 628.6798096 | 28.3239994 | 10.98666668 |
| *T. rhododiscus* | CladeC | 540 | 113.9 | 23.6 | 18.91333389 | 25.31933212 | 11.26000023 | 66.98519135 | 7.501333237 | 580.6488037 | 29.42000008 | 12.86800003 |
| *T. rhododiscus* | CladeC | 279 | 110.3080326 | 23.89268985 | 20.49799919 | 27.66199875 | 11.98933315 | 65.11100006 | 7.548666954 | 649.0462036 | 31.9279995 | 13.7913332 |
| *T. hekouense* | CladeC | 2109 | 103.70190730 | 22.89710733 | 15.57250023 | 20.11000061 | 9.596666336 | 83.78720856 | 7.830999851 | 431.6894226 | 23.57999992 | 10.43400002 |
| *T. hekouense* | CladeC | 1304 | 104.83083330 | 22.76888889 | 15.06949997 | 19.96800041 | 8.829999924 | 96.26661682 | 7.440999985 | 455.5104065 | 23.33200073 | 9.801333427 |
| *T. khoii* | CladeC | 1641 | 104.97844940 | 22.80255880 | 22.99200058 | 28.20866776 | 16.61066628 | 91.42478943 | 7.943999767 | 479.6765442 | 32.54000092 | 16.61066628 |
| *T. bicolor* | CladeC | 851 | 104.08859250 | 22.29036709 | 19.53966713 | 24.29933357 | 13.43266678 | 86.64796448 | 7.794000626 | 447.6006775 | 28.04400063 | 14.36266708 |
| *T. bicolor* | CladeC | 1194 | 100.85487080 | 24.43805773 | 20.02666664 | 24.67266655 | 13.92666626 | 85.07879639 | 11.61600018 | 445.6097412 | 29.50799942 | 15.67199993 |
| *T. bicolor* | CladeC | 1510 | 102.39293000 | 22.99345990 | 16.25083351 | 20.08733368 | 10.96199989 | 87.76134491 | 9.696332932 | 381.0263672 | 24.25600052 | 10.96199989 |
| *T. bicolor* | CladeC | 911 | 103.46204780 | 22.38856150 | 19.89066696 | 24.02866745 | 14.3220005 | 89.47354126 | 8.937999725 | 401.9405518 | 27.9640007 | 14.9746666 |
| *T. bicolor* | CladeC | 1130 | 104.02130130 | 22.33991443 | 20.74833298 | 25.54599953 | 14.6079998 | 81.16249847 | 7.817333221 | 450.1387024 | 29.37999916 | 15.54533291 |
| *T. bicolor* | CladeC | 1502 | 103.84378520 | 22.33636080 | 16.77283287 | 21.18533325 | 10.92599964 | 88.10197449 | 8.248332977 | 422.3874207 | 24.76399994 | 11.71133327 |
| *T. bicolor* | CladeC | 316 | 105.60873060 | 20.31674480 | 22.42033386 | 27.41266632 | 16.4659996 | 92.29748535 | 6.307332993 | 452.4957886 | 31.03199959 | 16.4659996 |
| *T. bicolor* | CladeC | 946 | 104.12155150 | 22.26368048 | 19.53966713 | 24.29933357 | 13.43266678 | 86.64796448 | 7.794000626 | 447.6006775 | 28.04400063 | 14.36266708 |
| *T. corticale* | CladeC | 728 | 106.19934080 | 21.85894965 | 20.64900017 | 25.92733383 | 14.08066654 | 86.71405792 | 7.061999321 | 490.8979492 | 29.58399963 | 15.22533321 |
| *T. corticale* | CladeC | 210 | 105.90820313 | 18.09625502 | 23.77750015 | 27.95000076 | 18.62533379 | 74.56789398 | 6.589666367 | 381.8769226 | 31.77999878 | 22.02666664 |
| *T. corticale* | CladeC | 1540 | 109.53197480 | 19.03453337 | 18.75016594 | 22.17399979 | 14.17333317 | 76.52571106 | 6.658333302 | 330.3912048 | 25.44799995 | 14.17333317 |
| *T. corticale* | CladeC | 1515 | 110.09262090 | 23.98123371 | 15.67416668 | 22.60533333 | 7.524000168 | 65.76712036 | 6.812999725 | 621.7077026 | 26.40399933 | 9.248666763 |
| *T. corticale* | CladeC | 1187 | 110.24434300 | 24.16839700 | 15.71850014 | 22.81266594 | 7.395999908 | 65.52026367 | 7.214333534 | 635.1541748 | 26.7159996 | 9.152667046 |
| *T. corticale* | CladeC | 1264 | 110.12832640 | 23.93228930 | 16.53316689 | 23.53733444 | 8.265999794 | 65.89420319 | 7.01766634 | 629.934082 | 27.45199966 | 10.00333309 |
| *T. corticale* | CladeC | 903 | 105.64178470 | 21.47415652 | 20.26133347 | 25.27133369 | 14.06400013 | 90.98410034 | 6.495333195 | 465.5130005 | 28.70400047 | 15.23333359 |
| *T. corticale* | CladeC | 246 | 105.65298300 | 20.29570260 | 22.21633339 | 27.23266602 | 16.22333336 | 92.69338989 | 6.175999641 | 454.890564 | 30.81999969 | 16.22333336 |
| *T. corticale* | CladeC | 612 | 107.89164700 | 21.84404300 | 19.47383308 | 25.03066635 | 12.59333324 | 82.82811737 | 6.11433363 | 514.1681519 | 28.19599915 | 13.97133255 |
| *T. corticale* | CladeC | 253 | 107.61451720 | 21.45818113 | 22.13966751 | 27.26399994 | 15.70600033 | 89.49414825 | 6.377333164 | 481.1777039 | 30.39599991 | 15.70600033 |
| *T. corticale* | CladeC | 789 | 106.50953290 | 22.91649993 | 20.45383263 | 26.16066742 | 13.26866722 | 88.33691406 | 6.910999775 | 528.9903564 | 29.52000046 | 14.5286665 |

**Table S3** Primers used for PCR and sequencing in this study.

| Marker | Primer name and sequence (5'-3') | Annealing  temp (ºC) | Source |
| --- | --- | --- | --- |
| 12S rRNA,  tRNAVal, 16S rRNA | L7270: AGATACCCCACTATGCCAAGTC | 55 | Li et al., 2009 |
|  | L7271: AGATACCCCACTATGCCTAGCC |  |  |
| COI | AH-CO1A-S: CTACAAYCCRCCRCCTRCTCGGCCAC | 50 | Grosjean et al., 2015 |
|  | AH-CO1A-AS1: TADACYTCDGGRTGDCCAAARAATCA |  |  |
| BDNF | BNDF-F: GACCATCCTTTTCCTKACTATGGT TATTTCATACTT | 57 | Li et al., 2009 |
|  | BNDF-R: CTATCTTCCCCTTTTAATGGTCAGTGTACAAAC |  |  |
| RHOD | L2903: ACC ATG AACGGA ACAGAAGGYCC | 57 | Bossuyt and Milinkovitch, 2000 |
|  | H2904: GTA GCGAAGAARCCTTCA AMGTA |  |  |
| TYR | Tyr1A: AGGTCCTCTTRAGCAAGGAATG | 57 | Bossuyt and Milinkovitch, 2000 |
|  | Tyr1G: TGCTGGGCRTCTCTCCARTCCCA |  |  |
| RAG1 | L6300:CTGGTCGTCAGATCTTTCAGC | 52 | Li et al., 2009 |
|  | H6301:GCAAAACGTTGAGAGTGATAAC |  |  |
| SIA | SIA1(T3): TCGAGTGCCCCGTGTGYTTYGAYTA | 57 | Bonacum et al., 2001 |
|  | SIA2(T7): GAAGTGGAAGCCGAAGCAGSWYTGCATCAT |  |  |

**References**

Bonacum, J., DeSalle, R., O’Grady, P., Olivera, D. S. C. G., Wintermute, J., & Zilversmit, M. J. D. I. S. (2001). New nuclear and mitochondrial primers for systematics and comparative genomics in Drosophilidae. Drosophila Information Service, 84, 201–204.

Bossuyt, F., & Milinkovitch, M. C. (2000). Convergent adaptive radiations in Madagascan and Asian ranid frogs reveal covariation between larval and adult traits. Proceedings of the national Academy of Sciences, 97(12), 6585–6590. https://doi.org/10.1073/pnas.97.12.6585

Grosjean, S., Ohler, A., Chuaynkern, Y., Cruaud, C., & Hassanin, A. (2015). Improving biodiversity assessment of anuran amphibians using DNA barcoding of tadpoles. Case studies from Southeast Asia. Comptes rendus biologies, 338(5), 351–361. https://doi.org/10.1016/j.crvi.2015.03.015

Li, J., Che, J., Murphy, R. W., Zhao, H., Zhao, E., Rao, D., & Zhang, Y. (2009). New insights to the molecular phylogenetics and generic assessment in the Rhacophoridae (Amphibia: Anura) based on five nuclear and three mitochondrial genes, with comments on the evolution of reproduction. *Molecular Phylogenetics and Evolution, 53*(2), 509–522. <https://doi.org/10.1016/j.ympev.2009.06.023>

**Table S4** Best-fit partitioning schemes and corresponding evolutionary models for three datasets evaluated using PartitionFinder v 2.1.1.

| Dataset | Subset | Partitions | Sites | Best fit model |
| --- | --- | --- | --- | --- |
| Dataset1 | 1 | 1216 | 2120 | GTR+I+G |
|  | 2 | COI | 714 | GTR+I+G |
| Dataset2 | 1 | 16S | 825 | GTR+I+G |
| Dataset3 | 1 | 1216 | 2120 | GTR+I+G |
|  | 2 | COI | 714 | TVM+I+G |
|  | 3 | RAG1 | 1158 | HKY+G |
|  | 4 | TYR, BNDF, SIA | 1713 | K80+I+G |
|  | 5 | RHOD | 315 | K8O+G |

**Table S5** Four morphological characteristic states for the 27 *Theloderma* species included in this study.

| ID | Species | Skin rough (1); smooth (0) | Body size small (0); large (1) | Vomerine teeth present (1); absent (0) | Hand web present (1); absent (0) | Source |
| --- | --- | --- | --- | --- | --- | --- |
| 1 | *Theloderma albopunctatum* | 0 | 0 | 0 | 1 | AmphibiaChina (2022) |
| 2 | *Theloderma annae* | 0 | 0 | 0 | 0 | Ngo et al. (2016) |
| 3 | *Theloderma asperum* | 0 | 0 | 0 | 1 | AmphibiaWeb 2022 |
| 4 | *Theloderma auratum* | 0 | 0 | 0 | 0 | Poyarkov et al. 2018 |
| 5 | *Theloderma baibungense* | 0 | 0 | 0 | 0 | AmphibiaChina (2022) |
| 6 | *Theloderma bicolor* | 1 | 1 | 1 | 1 | AmphibiaChina (2022) |
| 7 | *Theloderma corticale* | 1 | 1 | 1 | 1 | AmphibiaChina (2022) |
| 8 | *Theloderma gordoni* | 1 | 0 | 1 | 0 | AmphibiaChina (2022) |
| 9 | *Theloderma hekouense* | 1 | 0 | 0 | 0 | Du et al. 2022 |
| 10 | *Theloderma horridum* | 1 | 0 | 0 | 1 | Nguyen et al. 2015a |
| 11 | *Theloderma khoii* | 1 | 1 | 1 | 1 | Ninh et al. 2022 |
| 12 | *Theloderma lacustrinum* | 0 | 0 | 0 | 0 | Sivongxay et al. 2016 |
| 13 | *Theloderma laeve* | 0 | 0 | 0 | 0 | Nguyen et al. 2015a |
| 14 | *Theloderma lateriticum* | 1 | 0 | 0 | 0 | this study |
| 15 | *Theloderma leporosum* | 0 | 0 | 1 | 0 | Nguyen et al. 2015a |
| 16 | *Theloderma licin* | 0 | 0 | 0 | 1 | McLeod and Norhayati (2007) |
| 17 | *Theloderma moloch* | 1 | 1 | 1 | 1 | Li et al. 2016 |
| 18 | *Theloderma nebulosum* | 0 | 0 | 0 | 0 | Rowley et al. 2011 |
| 19 | *Theloderma palliatum* | 0 | 0 | 0 | 0 | Rowley et al. 2011 |
| 20 | *Theloderma petilum* | 0 | 0 | 1 | 0 | Stuart & Heatwole 2004 |
| 21 | *Theloderma phrynoderma* | 1 | 1 | 0 | 1 | Nguyen et al. 2015a |
| 22 | *Theloderma pyaukkya* | 0 | 0 | 0 | 0 | Dever 2017 |
| 23 | *Theloderma rhododiscus* | 1 | 0 | 0 | 0 | AmphibiaChina (2022) |
| 24 | *Theloderma ryabovi* | 1 | 1 | 0 | 0 | Orlov et al. (2006) |
| 25 | *Theloderma stellatum* | 1 | 0 | 0 | 1 | Nguyen et al. 2015a |
| 26 | *Theloderma truongsonense* | 0 | 0 | 0 | 0 | Nguyen et al. 2015a |
| 27 | *Theloderma vietnamense* | 1 | 0 | 0 | 1 | Poyarkov et al. 2015 |

**Table S6** Based on the correlation analysis of 19 environments, pink undertones indicate high correlation and bold denote selected bioclimatic variables.

| Bioclimatic  variables | Bio1 | Bio2 | Bio3 | Bio4 | Bio5 | Bio6 | Bio7 | Bio8 | Bio9 | Bio10 | Bio11 | Bio12 | Bio13 | Bio14 | Bio15 | Bio16 | Bio17 | Bio18 | Bio19 |
| --- | --- | --- | --- | --- | --- | --- | --- | --- | --- | --- | --- | --- | --- | --- | --- | --- | --- | --- | --- |
| **Bio1** |  | 0.59 | 0.70 | -0.54 | 0.97 | 0.97 | -0.36 | 0.95 | 0.92 | 0.98 | 0.98 | 0.54 | 0.54 | 0.31 | -0.39 | 0.56 | 0.33 | 0.52 | 0.32 |
| **Bio2** |  |  | 0.44 | -0.16 | 0.66 | 0.49 | 0.12 | 0.56 | 0.53 | 0.61 | 0.54 | 0.02 | 0.10 | -0.10 | 0.05 | 0.10 | -0.11 | 0.10 | -0.09 |
| **Bio3** |  |  |  | -0.85 | 0.55 | 0.80 | -0.74 | 0.53 | 0.78 | 0.56 | 0.80 | 0.64 | 0.63 | 0.35 | -0.05 | 0.64 | 0.38 | 0.48 | 0.48 |
| Bio4 |  |  |  |  | -0.32 | -0.71 | 0.96 | -0.30 | -0.73 | -0.35 | -0.70 | -0.56 | -0.56 | -0.28 | 0.02 | -0.56 | -0.31 | -0.36 | -0.43 |
| Bio5 |  |  |  |  |  | 0.89 | -0.12 | 0.97 | 0.83 | 1.00 | 0.90 | 0.43 | 0.44 | 0.25 | -0.41 | 0.46 | 0.26 | 0.46 | 0.22 |
| Bio6 |  |  |  |  |  |  | -0.56 | 0.87 | 0.96 | 0.90 | 1.00 | 0.61 | 0.60 | 0.36 | -0.35 | 0.62 | 0.38 | 0.53 | 0.39 |
| **Bio7** |  |  |  |  |  |  |  | -0.13 | -0.58 | -0.16 | -0.54 | -0.54 | -0.51 | -0.31 | 0.02 | -0.51 | -0.35 | -0.32 | -0.45 |
| Bio8 |  |  |  |  |  |  |  |  | 0.76 | 0.98 | 0.88 | 0.47 | 0.47 | 0.29 | -0.47 | 0.49 | 0.30 | 0.53 | 0.23 |
| Bio9 |  |  |  |  |  |  |  |  |  | 0.84 | 0.96 | 0.52 | 0.53 | 0.29 | -0.29 | 0.54 | 0.31 | 0.40 | 0.37 |
| Bio10 |  |  |  |  |  |  |  |  |  |  | 0.92 | 0.46 | 0.46 | 0.28 | -0.43 | 0.48 | 0.29 | 0.48 | 0.24 |
| Bio11 |  |  |  |  |  |  |  |  |  |  |  | 0.59 | 0.59 | 0.33 | -0.33 | 0.61 | 0.35 | 0.52 | 0.37 |
| **Bio12** |  |  |  |  |  |  |  |  |  |  |  |  | 0.91 | 0.75 | -0.33 | 0.94 | 0.78 | 0.84 | 0.76 |
| Bio13 |  |  |  |  |  |  |  |  |  |  |  |  |  | 0.48 | -0.11 | 0.99 | 0.51 | 0.78 | 0.62 |
| Bio14 |  |  |  |  |  |  |  |  |  |  |  |  |  |  | -0.47 | 0.51 | 0.99 | 0.62 | 0.68 |
| **Bio15** |  |  |  |  |  |  |  |  |  |  |  |  |  |  |  | -0.16 | -0.48 | -0.30 | -0.30 |
| Bio16 |  |  |  |  |  |  |  |  |  |  |  |  |  |  |  |  | 0.55 | 0.81 | 0.64 |
| Bio17 |  |  |  |  |  |  |  |  |  |  |  |  |  |  |  |  |  | 0.64 | 0.71 |
| Bio18 |  |  |  |  |  |  |  |  |  |  |  |  |  |  |  |  |  |  | 0.42 |
| **Bio19** |  |  |  |  |  |  |  |  |  |  |  |  |  |  |  |  |  |  |  |

**Table S7** Uncorrected pairwise *p*-distances (%) between *Theloderma* species based on the 16S rRNA gene sequences.

| ID | Species | 1 | 2 | 3 | 4 | 5 | 6 | 7 | 8 | 9 | 10 | 11 | 12 | 13 | 14 | 15 | 16 | 17 | 18 | 19 | 20 | 21 | 22 | 23 | 24 | 25 | 26 | 27 | 28 | 29 | 30 | 31 | 32 | 33 | 34 | 35 | 36 | 37 | 38 | 39 | 40 | 41 | 42 | 43 | 44 | 45 | 46 | 47 | 48 | 49 | 50 |
| --- | --- | --- | --- | --- | --- | --- | --- | --- | --- | --- | --- | --- | --- | --- | --- | --- | --- | --- | --- | --- | --- | --- | --- | --- | --- | --- | --- | --- | --- | --- | --- | --- | --- | --- | --- | --- | --- | --- | --- | --- | --- | --- | --- | --- | --- | --- | --- | --- | --- | --- | --- |
| 1 | *T. albopunctatum_*1 |  |  |  |  |  |  |  |  |  |  |  |  |  |  |  |  |  |  |  |  |  |  |  |  |  |  |  |  |  |  |  |  |  |  |  |  |  |  |  |  |  |  |  |  |  |  |  |  |  |  |
| 2 | *T. albopunctatum_*2 | 0.0 |  |  |  |  |  |  |  |  |  |  |  |  |  |  |  |  |  |  |  |  |  |  |  |  |  |  |  |  |  |  |  |  |  |  |  |  |  |  |  |  |  |  |  |  |  |  |  |  |  |
| 3 | *T. albopunctatum_*3 | 4.5 | 4.5 |  |  |  |  |  |  |  |  |  |  |  |  |  |  |  |  |  |  |  |  |  |  |  |  |  |  |  |  |  |  |  |  |  |  |  |  |  |  |  |  |  |  |  |  |  |  |  |  |
| 4 | *T. albopunctatum_*4 | 4.5 | 4.5 | 0.0 |  |  |  |  |  |  |  |  |  |  |  |  |  |  |  |  |  |  |  |  |  |  |  |  |  |  |  |  |  |  |  |  |  |  |  |  |  |  |  |  |  |  |  |  |  |  |  |
| 5 | *T. albopunctatum_*5 | 4.9 | 4.9 | 0.3 | 0.3 |  |  |  |  |  |  |  |  |  |  |  |  |  |  |  |  |  |  |  |  |  |  |  |  |  |  |  |  |  |  |  |  |  |  |  |  |  |  |  |  |  |  |  |  |  |  |
| 6 | *T. albopunctatum_*6 | 5.6 | 4.5 | 4.5 | 4.5 | 4.9 |  |  |  |  |  |  |  |  |  |  |  |  |  |  |  |  |  |  |  |  |  |  |  |  |  |  |  |  |  |  |  |  |  |  |  |  |  |  |  |  |  |  |  |  |  |
| 7 | *T. albopunctatum_*7 | 3.5 | 3.0 | 3.8 | 3.8 | 3.5 | 4.2 |  |  |  |  |  |  |  |  |  |  |  |  |  |  |  |  |  |  |  |  |  |  |  |  |  |  |  |  |  |  |  |  |  |  |  |  |  |  |  |  |  |  |  |  |
| 8 | *T. albopunctatum_*8 | 4.9 | 4.5 | 4.5 | 4.5 | 4.2 | 3.5 | 2.8 |  |  |  |  |  |  |  |  |  |  |  |  |  |  |  |  |  |  |  |  |  |  |  |  |  |  |  |  |  |  |  |  |  |  |  |  |  |  |  |  |  |  |  |
| 9 | *T. albopunctatum_*9 | 4.9 | 4.9 | 3.1 | 3.1 | 3.5 | 4.9 | 3.8 | 3.8 |  |  |  |  |  |  |  |  |  |  |  |  |  |  |  |  |  |  |  |  |  |  |  |  |  |  |  |  |  |  |  |  |  |  |  |  |  |  |  |  |  |  |
| 10 | *T. albopunctatum_*10 | 5.0 | 4.5 | 4.0 | 4.0 | 4.3 | 3.6 | 3.6 | 3.2 | 2.9 |  |  |  |  |  |  |  |  |  |  |  |  |  |  |  |  |  |  |  |  |  |  |  |  |  |  |  |  |  |  |  |  |  |  |  |  |  |  |  |  |  |
| 11 | *T. albopunctatum_*11 | 4.9 | 4.5 | 3.8 | 3.8 | 4.2 | 4.2 | 3.5 | 3.1 | 2.8 | 0.0 |  |  |  |  |  |  |  |  |  |  |  |  |  |  |  |  |  |  |  |  |  |  |  |  |  |  |  |  |  |  |  |  |  |  |  |  |  |  |  |  |
| 12 | *T. albopunctatum_*12 | 5.2 | 4.5 | 4.2 | 4.2 | 4.5 | 4.5 | 3.8 | 3.5 | 3.1 | 0.4 | 0.3 |  |  |  |  |  |  |  |  |  |  |  |  |  |  |  |  |  |  |  |  |  |  |  |  |  |  |  |  |  |  |  |  |  |  |  |  |  |  |  |
| 13 | *T. albopunctatum_*13 | 5.2 | 4.9 | 3.5 | 3.5 | 3.8 | 3.8 | 3.8 | 3.5 | 3.1 | 0.4 | 0.3 | 0.7 |  |  |  |  |  |  |  |  |  |  |  |  |  |  |  |  |  |  |  |  |  |  |  |  |  |  |  |  |  |  |  |  |  |  |  |  |  |  |
| 14 | *T. albopunctatum_*14 | 5.2 | 4.9 | 3.5 | 3.5 | 3.8 | 3.8 | 3.8 | 3.5 | 3.1 | 0.4 | 0.3 | 0.7 | 0.0 |  |  |  |  |  |  |  |  |  |  |  |  |  |  |  |  |  |  |  |  |  |  |  |  |  |  |  |  |  |  |  |  |  |  |  |  |  |
| 15 | *T. albopunctatum_*15 | 5.2 | 4.9 | 3.5 | 3.5 | 3.8 | 3.8 | 3.8 | 3.5 | 3.1 | 0.4 | 0.3 | 0.7 | 0.0 | 0.0 |  |  |  |  |  |  |  |  |  |  |  |  |  |  |  |  |  |  |  |  |  |  |  |  |  |  |  |  |  |  |  |  |  |  |  |  |
| 16 | *T. albopunctatum_*16 | 5.2 | 4.9 | 3.5 | 3.5 | 3.8 | 3.8 | 3.8 | 3.5 | 3.1 | 0.4 | 0.3 | 0.7 | 0.0 | 0.0 | 0.0 |  |  |  |  |  |  |  |  |  |  |  |  |  |  |  |  |  |  |  |  |  |  |  |  |  |  |  |  |  |  |  |  |  |  |  |
| 17 | *T. albopunctatum_*17 | 3.5 | 3.0 | 4.5 | 4.5 | 4.9 | 4.9 | 3.5 | 4.2 | 4.2 | 2.2 | 2.1 | 2.4 | 2.4 | 2.4 | 2.4 | 2.4 |  |  |  |  |  |  |  |  |  |  |  |  |  |  |  |  |  |  |  |  |  |  |  |  |  |  |  |  |  |  |  |  |  |  |
| 18 | *T. albopunctatum_*18 | 3.5 | 3.0 | 3.8 | 3.8 | 4.2 | 4.2 | 3.5 | 4.2 | 4.2 | 2.9 | 2.8 | 3.1 | 2.4 | 2.4 | 2.4 | 2.4 | 0.7 |  |  |  |  |  |  |  |  |  |  |  |  |  |  |  |  |  |  |  |  |  |  |  |  |  |  |  |  |  |  |  |  |  |
| 19 | *T. albopunctatum_*19 | 3.8 | 3.6 | 3.8 | 3.8 | 3.8 | 4.3 | 2.9 | 3.3 | 3.8 | 2.5 | 2.4 | 2.9 | 1.9 | 1.9 | 1.9 | 1.9 | 1.0 | 0.0 |  |  |  |  |  |  |  |  |  |  |  |  |  |  |  |  |  |  |  |  |  |  |  |  |  |  |  |  |  |  |  |  |
| 20 | *T. albopunctatum_*20 | 3.8 | 3.4 | 4.2 | 4.2 | 4.5 | 4.5 | 3.8 | 4.5 | 4.5 | 3.2 | 3.1 | 3.5 | 2.8 | 2.8 | 2.8 | 2.8 | 1.0 | 0.3 | 0.5 |  |  |  |  |  |  |  |  |  |  |  |  |  |  |  |  |  |  |  |  |  |  |  |  |  |  |  |  |  |  |  |
| 21 | *T. albopunctatum_*21 | 3.8 | 3.4 | 4.2 | 4.2 | 4.5 | 4.5 | 3.8 | 4.5 | 4.5 | 3.2 | 3.1 | 3.5 | 2.8 | 2.8 | 2.8 | 2.8 | 1.0 | 0.3 | 0.5 | 0.0 |  |  |  |  |  |  |  |  |  |  |  |  |  |  |  |  |  |  |  |  |  |  |  |  |  |  |  |  |  |  |
| 22 | *T. albopunctatum_*22 | 2.8 | 2.2 | 3.8 | 3.8 | 4.2 | 4.2 | 2.8 | 3.5 | 3.5 | 2.2 | 2.1 | 2.4 | 2.4 | 2.4 | 2.4 | 2.4 | 0.7 | 1.4 | 1.4 | 1.7 | 1.7 |  |  |  |  |  |  |  |  |  |  |  |  |  |  |  |  |  |  |  |  |  |  |  |  |  |  |  |  |  |
| 23 | *T. albopunctatum_*23 | 3.5 | 3.0 | 3.8 | 3.8 | 4.2 | 4.2 | 3.5 | 4.2 | 4.2 | 2.9 | 2.8 | 3.1 | 2.4 | 2.4 | 2.4 | 2.4 | 1.4 | 1.4 | 1.4 | 1.7 | 1.7 | 0.7 |  |  |  |  |  |  |  |  |  |  |  |  |  |  |  |  |  |  |  |  |  |  |  |  |  |  |  |  |
| 24 | *T. albopunctatum_*24 | 3.5 | 3.0 | 3.8 | 3.8 | 4.2 | 4.2 | 3.5 | 4.2 | 4.2 | 2.9 | 2.8 | 3.1 | 2.4 | 2.4 | 2.4 | 2.4 | 1.4 | 1.4 | 1.4 | 1.7 | 1.7 | 0.7 | 0.0 |  |  |  |  |  |  |  |  |  |  |  |  |  |  |  |  |  |  |  |  |  |  |  |  |  |  |  |
| 25 | *T. albopunctatum_*25 | 3.1 | 2.6 | 4.2 | 4.2 | 4.5 | 4.5 | 3.1 | 3.8 | 3.8 | 2.5 | 2.4 | 2.8 | 2.8 | 2.8 | 2.8 | 2.8 | 1.0 | 1.7 | 1.9 | 2.1 | 2.1 | 0.3 | 1.0 | 1.0 |  |  |  |  |  |  |  |  |  |  |  |  |  |  |  |  |  |  |  |  |  |  |  |  |  |  |
| 26 | *T. albopunctatum_*26 | 3.1 | 2.6 | 4.2 | 4.2 | 4.5 | 4.5 | 3.1 | 3.8 | 3.8 | 2.5 | 2.4 | 2.8 | 2.8 | 2.8 | 2.8 | 2.8 | 1.0 | 1.7 | 1.9 | 2.1 | 2.1 | 0.3 | 1.0 | 1.0 | 0.0 |  |  |  |  |  |  |  |  |  |  |  |  |  |  |  |  |  |  |  |  |  |  |  |  |  |
| 27 | *T. albopunctatum_*28 | 2.8 | 2.2 | 3.8 | 3.8 | 4.2 | 4.2 | 2.8 | 3.5 | 3.5 | 2.2 | 2.1 | 2.4 | 2.4 | 2.4 | 2.4 | 2.4 | 0.7 | 1.4 | 1.4 | 1.7 | 1.7 | 0.0 | 0.7 | 0.7 | 0.3 | 0.3 |  |  |  |  |  |  |  |  |  |  |  |  |  |  |  |  |  |  |  |  |  |  |  |  |
| 28 | *T. albopunctatum_*29 | 2.8 | 2.2 | 3.8 | 3.8 | 4.2 | 4.2 | 2.8 | 3.5 | 3.5 | 2.2 | 2.1 | 2.4 | 2.4 | 2.4 | 2.4 | 2.4 | 0.7 | 1.4 | 1.4 | 1.7 | 1.7 | 0.0 | 0.7 | 0.7 | 0.3 | 0.3 | 0.0 |  |  |  |  |  |  |  |  |  |  |  |  |  |  |  |  |  |  |  |  |  |  |  |
| 29 | *T. albopunctatum_*30 | 2.8 | 2.2 | 3.8 | 3.8 | 4.2 | 4.2 | 2.8 | 3.5 | 3.5 | 2.2 | 2.1 | 2.4 | 2.4 | 2.4 | 2.4 | 2.4 | 0.7 | 1.4 | 1.4 | 1.7 | 1.7 | 0.0 | 0.7 | 0.7 | 0.3 | 0.3 | 0.0 | 0.0 |  |  |  |  |  |  |  |  |  |  |  |  |  |  |  |  |  |  |  |  |  |  |
| 30 | *T. albopunctatum_*31 | 2.8 | 2.2 | 3.8 | 3.8 | 4.2 | 4.2 | 2.8 | 3.5 | 3.5 | 2.2 | 2.1 | 2.4 | 2.4 | 2.4 | 2.4 | 2.4 | 0.7 | 1.4 | 1.4 | 1.7 | 1.7 | 0.0 | 0.7 | 0.7 | 0.3 | 0.3 | 0.0 | 0.0 | 0.0 |  |  |  |  |  |  |  |  |  |  |  |  |  |  |  |  |  |  |  |  |  |
| 31 | *T. albopunctatum_*32 | 2.8 | 2.2 | 3.8 | 3.8 | 4.2 | 4.2 | 2.8 | 3.5 | 3.5 | 2.2 | 2.1 | 2.4 | 2.4 | 2.4 | 2.4 | 2.4 | 0.7 | 1.4 | 1.4 | 1.7 | 1.7 | 0.0 | 0.7 | 0.7 | 0.3 | 0.3 | 0.0 | 0.0 | 0.0 | 0.0 |  |  |  |  |  |  |  |  |  |  |  |  |  |  |  |  |  |  |  |  |
| 32 | *T. albopunctatum_*33 | 2.8 | 2.2 | 3.8 | 3.8 | 4.2 | 4.2 | 2.8 | 3.5 | 3.5 | 2.2 | 2.1 | 2.4 | 2.4 | 2.4 | 2.4 | 2.4 | 0.7 | 1.4 | 1.4 | 1.7 | 1.7 | 0.0 | 0.7 | 0.7 | 0.3 | 0.3 | 0.0 | 0.0 | 0.0 | 0.0 | 0.0 |  |  |  |  |  |  |  |  |  |  |  |  |  |  |  |  |  |  |  |
| 33 | *T. albopunctatum_*34 | 2.8 | 2.2 | 3.8 | 3.8 | 4.2 | 4.2 | 2.8 | 3.5 | 3.5 | 2.2 | 2.1 | 2.4 | 2.4 | 2.4 | 2.4 | 2.4 | 0.7 | 1.4 | 1.4 | 1.7 | 1.7 | 0.0 | 0.7 | 0.7 | 0.3 | 0.3 | 0.0 | 0.0 | 0.0 | 0.0 | 0.0 | 0.0 |  |  |  |  |  |  |  |  |  |  |  |  |  |  |  |  |  |  |
| 34 | *T. albopunctatum_*35 | 2.2 | 2.2 | 3.7 | 3.7 | 4.1 | 3.7 | 3.0 | 3.7 | 3.4 | 2.2 | 2.2 | 2.2 | 2.6 | 2.6 | 2.6 | 2.6 | 0.7 | 1.5 | 1.6 | 1.9 | 1.9 | 0.0 | 0.7 | 0.7 | 0.4 | 0.4 | 0.0 | 0.0 | 0.0 | 0.0 | 0.0 | 0.0 | 0.0 |  |  |  |  |  |  |  |  |  |  |  |  |  |  |  |  |  |
| 35 | *T. albopunctatum_*36 | 2.8 | 2.2 | 3.8 | 3.8 | 4.2 | 4.2 | 2.8 | 3.5 | 3.5 | 2.2 | 2.1 | 2.4 | 2.4 | 2.4 | 2.4 | 2.4 | 0.7 | 1.4 | 1.4 | 1.7 | 1.7 | 0.0 | 0.7 | 0.7 | 0.3 | 0.3 | 0.0 | 0.0 | 0.0 | 0.0 | 0.0 | 0.0 | 0.0 | 0.0 |  |  |  |  |  |  |  |  |  |  |  |  |  |  |  |  |
| 36 | *T. albopunctatum_*37 | 3.5 | 2.2 | 4.5 | 4.5 | 4.9 | 3.5 | 3.5 | 4.2 | 4.2 | 2.2 | 2.8 | 3.1 | 3.1 | 3.1 | 3.1 | 3.1 | 1.4 | 2.1 | 2.4 | 2.4 | 2.4 | 0.7 | 1.4 | 1.4 | 1.0 | 1.0 | 0.7 | 0.7 | 0.7 | 0.7 | 0.7 | 0.7 | 0.7 | 0.0 | 0.7 |  |  |  |  |  |  |  |  |  |  |  |  |  |  |  |
| 37 | *T. annae_*2 | 17.7 | 17.9 | 18.4 | 18.4 | 18.8 | 17.7 | 17.7 | 17.7 | 17.0 | 18.0 | 17.4 | 17.7 | 17.4 | 17.4 | 17.4 | 17.4 | 17.0 | 16.7 | 18.1 | 16.7 | 16.7 | 16.3 | 16.7 | 16.7 | 16.7 | 16.7 | 16.3 | 16.3 | 16.3 | 16.3 | 16.4 | 16.3 | 16.3 | 17.2 | 16.3 | 17.0 |  |  |  |  |  |  |  |  |  |  |  |  |  |  |
| 38 | *T. annae_*3 | 17.0 | 17.2 | 18.4 | 18.4 | 18.8 | 17.7 | 17.7 | 17.7 | 17.0 | 18.0 | 17.4 | 17.7 | 17.4 | 17.4 | 17.4 | 17.4 | 17.0 | 16.7 | 18.1 | 16.7 | 16.7 | 16.3 | 16.7 | 16.7 | 16.7 | 16.7 | 16.3 | 16.3 | 16.3 | 16.3 | 16.4 | 16.3 | 16.3 | 17.2 | 16.3 | 17.0 | 3.5 |  |  |  |  |  |  |  |  |  |  |  |  |  |
| 39 | *T. annae_*4 | 17.0 | 17.2 | 18.4 | 18.4 | 18.8 | 17.7 | 17.7 | 17.7 | 17.0 | 18.0 | 17.4 | 17.7 | 17.4 | 17.4 | 17.4 | 17.4 | 17.0 | 16.7 | 18.1 | 16.7 | 16.7 | 16.3 | 16.7 | 16.7 | 16.7 | 16.7 | 16.3 | 16.3 | 16.3 | 16.3 | 16.4 | 16.3 | 16.3 | 17.2 | 16.3 | 17.0 | 3.5 | 0.0 |  |  |  |  |  |  |  |  |  |  |  |  |
| 40 | *T. annae_*5 | 17.0 | 17.2 | 18.4 | 18.4 | 18.8 | 17.7 | 17.7 | 17.7 | 17.0 | 18.0 | 17.4 | 17.7 | 17.4 | 17.4 | 17.4 | 17.4 | 17.0 | 16.7 | 18.1 | 16.7 | 16.7 | 16.3 | 16.7 | 16.7 | 16.7 | 16.7 | 16.3 | 16.3 | 16.3 | 16.3 | 16.4 | 16.3 | 16.3 | 17.2 | 16.3 | 17.0 | 3.5 | 0.0 | 0.0 |  |  |  |  |  |  |  |  |  |  |  |
| 41 | *T. asperum_*1 | 11.1 | 11.2 | 12.2 | 12.2 | 12.5 | 12.5 | 11.8 | 11.8 | 10.8 | 11.9 | 11.5 | 11.8 | 11.1 | 11.1 | 11.1 | 11.1 | 11.1 | 10.4 | 11.4 | 10.8 | 10.8 | 10.4 | 10.4 | 10.4 | 10.1 | 10.1 | 10.4 | 10.4 | 10.4 | 10.4 | 10.5 | 10.4 | 10.4 | 11.2 | 10.4 | 11.1 | 15.3 | 14.2 | 14.2 | 14.2 |  |  |  |  |  |  |  |  |  |  |
| 42 | *T. asperum_*2 | 11.1 | 11.2 | 12.2 | 12.2 | 12.5 | 12.5 | 11.8 | 11.8 | 10.8 | 11.9 | 11.5 | 11.8 | 11.1 | 11.1 | 11.1 | 11.1 | 11.1 | 10.4 | 11.4 | 10.8 | 10.8 | 10.4 | 10.4 | 10.4 | 10.1 | 10.1 | 10.4 | 10.4 | 10.4 | 10.4 | 10.5 | 10.4 | 10.4 | 11.2 | 10.4 | 11.1 | 15.3 | 14.2 | 14.2 | 14.2 | 0.0 |  |  |  |  |  |  |  |  |  |
| 43 | *T. auratum_*1 | 14.2 | 14.6 | 16.0 | 16.0 | 16.3 | 16.7 | 14.6 | 15.3 | 15.3 | 15.8 | 15.3 | 15.6 | 15.3 | 15.3 | 15.3 | 15.3 | 14.6 | 14.2 | 13.8 | 14.6 | 14.6 | 13.9 | 13.5 | 13.5 | 14.2 | 14.2 | 13.9 | 13.9 | 13.9 | 13.9 | 13.9 | 13.9 | 13.9 | 14.9 | 13.9 | 14.6 | 14.6 | 14.9 | 14.9 | 14.9 | 14.2 | 14.2 |  |  |  |  |  |  |  |  |
| 44 | *T. auratum_*2 | 14.2 | 14.6 | 16.0 | 16.0 | 16.3 | 16.7 | 14.6 | 15.3 | 15.3 | 15.8 | 15.3 | 15.6 | 15.3 | 15.3 | 15.3 | 15.3 | 14.6 | 14.2 | 13.8 | 14.6 | 14.6 | 13.9 | 13.5 | 13.5 | 14.2 | 14.2 | 13.9 | 13.9 | 13.9 | 13.9 | 13.9 | 13.9 | 13.9 | 14.9 | 13.9 | 14.6 | 14.6 | 14.9 | 14.9 | 14.9 | 14.2 | 14.2 | 0.0 |  |  |  |  |  |  |  |
| 45 | *T. auratum_*3 | 14.2 | 14.6 | 16.0 | 16.0 | 16.3 | 16.7 | 14.6 | 15.3 | 15.3 | 15.8 | 15.3 | 15.6 | 15.3 | 15.3 | 15.3 | 15.3 | 14.6 | 14.2 | 13.8 | 14.6 | 14.6 | 13.9 | 13.5 | 13.5 | 14.2 | 14.2 | 13.9 | 13.9 | 13.9 | 13.9 | 13.9 | 13.9 | 13.9 | 14.9 | 13.9 | 14.6 | 14.6 | 14.9 | 14.9 | 14.9 | 14.2 | 14.2 | 0.0 | 0.0 |  |  |  |  |  |  |
| 46 | *T. auratum_*4 | 13.9 | 14.2 | 15.6 | 15.6 | 16.0 | 16.3 | 14.2 | 14.9 | 14.9 | 15.5 | 14.9 | 15.3 | 14.9 | 14.9 | 14.9 | 14.9 | 14.2 | 13.9 | 13.3 | 14.2 | 14.2 | 13.5 | 13.2 | 13.2 | 13.9 | 13.9 | 13.5 | 13.5 | 13.5 | 13.5 | 13.6 | 13.5 | 13.5 | 14.6 | 13.5 | 14.2 | 14.2 | 14.6 | 14.6 | 14.6 | 13.9 | 13.9 | 0.3 | 0.3 | 0.3 |  |  |  |  |  |
| 47 | *T. auratum_*5 | 14.2 | 14.6 | 16.0 | 16.0 | 16.3 | 16.7 | 14.6 | 15.3 | 15.3 | 15.8 | 15.3 | 15.6 | 15.3 | 15.3 | 15.3 | 15.3 | 14.6 | 14.2 | 13.8 | 14.6 | 14.6 | 13.9 | 13.5 | 13.5 | 14.2 | 14.2 | 13.9 | 13.9 | 13.9 | 13.9 | 13.9 | 13.9 | 13.9 | 14.9 | 13.9 | 14.6 | 14.6 | 14.9 | 14.9 | 14.9 | 14.2 | 14.2 | 0.7 | 0.7 | 0.7 | 0.3 |  |  |  |  |
| 48 | *T. auratum_*6 | 14.2 | 14.6 | 16.0 | 16.0 | 16.3 | 16.7 | 14.6 | 15.3 | 15.3 | 15.8 | 15.3 | 15.6 | 15.3 | 15.3 | 15.3 | 15.3 | 14.6 | 14.2 | 13.8 | 14.6 | 14.6 | 13.9 | 13.5 | 13.5 | 14.2 | 14.2 | 13.9 | 13.9 | 13.9 | 13.9 | 13.9 | 13.9 | 13.9 | 14.9 | 13.9 | 14.6 | 14.6 | 14.9 | 14.9 | 14.9 | 14.2 | 14.2 | 0.7 | 0.7 | 0.7 | 0.3 | 0.0 |  |  |  |
| 49 | *T. baibungense_*2 | 11.8 | 11.9 | 12.8 | 12.8 | 13.2 | 13.2 | 13.2 | 12.5 | 11.5 | 13.3 | 12.8 | 13.2 | 12.5 | 12.5 | 12.5 | 12.5 | 12.5 | 11.8 | 12.4 | 12.2 | 12.2 | 11.8 | 11.8 | 11.8 | 11.5 | 11.5 | 11.8 | 11.8 | 11.8 | 11.8 | 11.8 | 11.8 | 11.8 | 11.9 | 11.8 | 12.5 | 14.2 | 12.5 | 12.5 | 12.5 | 8.0 | 8.0 | 14.6 | 14.6 | 14.6 | 14.2 | 14.6 | 14.6 |  |  |
| 50 | *T. baibungense_*3 | 11.1 | 11.2 | 12.8 | 12.8 | 13.2 | 13.2 | 12.5 | 11.8 | 10.8 | 12.6 | 12.2 | 12.5 | 12.5 | 12.5 | 12.5 | 12.5 | 11.8 | 11.8 | 12.4 | 12.2 | 12.2 | 11.1 | 11.8 | 11.8 | 10.8 | 10.8 | 11.1 | 11.1 | 11.1 | 11.1 | 11.1 | 11.1 | 11.1 | 11.2 | 11.1 | 11.8 | 13.9 | 12.2 | 12.2 | 12.2 | 8.0 | 8.0 | 15.3 | 15.3 | 15.3 | 14.9 | 15.3 | 15.3 | 1.4 |  |
| 51 | *T. baibungense_*4 | 11.1 | 11.2 | 12.8 | 12.8 | 13.2 | 13.2 | 12.5 | 11.8 | 10.8 | 12.6 | 12.2 | 12.5 | 12.5 | 12.5 | 12.5 | 12.5 | 11.8 | 11.8 | 12.4 | 12.2 | 12.2 | 11.1 | 11.8 | 11.8 | 10.8 | 10.8 | 11.1 | 11.1 | 11.1 | 11.1 | 11.1 | 11.1 | 11.1 | 11.2 | 11.1 | 11.8 | 13.9 | 12.2 | 12.2 | 12.2 | 8.0 | 8.0 | 15.3 | 15.3 | 15.3 | 14.9 | 15.3 | 15.3 | 1.4 | 0.0 |
| 52 | *T. bicolor_*1 | 12.5 | 12.7 | 13.9 | 13.9 | 14.2 | 14.6 | 13.9 | 13.9 | 12.8 | 13.7 | 13.5 | 13.9 | 13.5 | 13.5 | 13.5 | 13.5 | 11.8 | 11.5 | 11.9 | 11.8 | 11.8 | 11.8 | 11.5 | 11.5 | 12.2 | 12.2 | 11.8 | 11.8 | 11.8 | 11.8 | 11.8 | 11.8 | 11.8 | 11.9 | 11.8 | 12.5 | 11.8 | 12.2 | 12.2 | 12.2 | 12.5 | 12.5 | 8.0 | 8.0 | 8.0 | 7.6 | 8.0 | 8.0 | 12.2 | 12.2 |
| 53 | *T. bicolor_*2 | 12.2 | 12.7 | 13.9 | 13.9 | 14.2 | 14.6 | 13.9 | 13.9 | 12.8 | 14.0 | 13.5 | 13.9 | 13.5 | 13.5 | 13.5 | 13.5 | 11.8 | 11.5 | 11.9 | 11.8 | 11.8 | 11.8 | 11.5 | 11.5 | 12.2 | 12.2 | 11.8 | 11.8 | 11.8 | 11.8 | 11.8 | 11.8 | 11.8 | 12.3 | 11.8 | 12.5 | 11.8 | 11.8 | 11.8 | 11.8 | 12.5 | 12.5 | 8.0 | 8.0 | 8.0 | 7.6 | 8.0 | 8.0 | 11.8 | 11.8 |
| 54 | *T. bicolor_*3 | 12.5 | 12.7 | 14.2 | 14.2 | 14.6 | 14.9 | 14.2 | 14.2 | 13.2 | 14.0 | 13.9 | 14.2 | 13.9 | 13.9 | 13.9 | 13.9 | 12.2 | 11.8 | 12.4 | 12.2 | 12.2 | 12.2 | 11.8 | 11.8 | 12.5 | 12.5 | 12.2 | 12.2 | 12.2 | 12.2 | 12.2 | 12.2 | 12.2 | 12.3 | 12.2 | 12.8 | 12.2 | 12.2 | 12.2 | 12.2 | 12.8 | 12.8 | 8.3 | 8.3 | 8.3 | 8.0 | 8.3 | 8.3 | 12.2 | 12.2 |
| 55 | *T. bicolor_*4 | 12.5 | 12.7 | 14.2 | 14.2 | 14.6 | 14.9 | 14.2 | 14.2 | 13.2 | 14.0 | 13.9 | 14.2 | 13.9 | 13.9 | 13.9 | 13.9 | 12.2 | 11.8 | 12.4 | 12.2 | 12.2 | 12.2 | 11.8 | 11.8 | 12.5 | 12.5 | 12.2 | 12.2 | 12.2 | 12.2 | 12.2 | 12.2 | 12.2 | 12.3 | 12.2 | 12.8 | 12.2 | 12.2 | 12.2 | 12.2 | 12.8 | 12.8 | 8.3 | 8.3 | 8.3 | 8.0 | 8.3 | 8.3 | 12.2 | 12.2 |
| 56 | *T. bicolor_*5 | 12.5 | 12.7 | 13.9 | 13.9 | 14.2 | 14.6 | 13.9 | 13.9 | 12.8 | 13.7 | 13.5 | 13.9 | 13.5 | 13.5 | 13.5 | 13.5 | 11.8 | 11.5 | 11.9 | 11.8 | 11.8 | 11.8 | 11.5 | 11.5 | 12.2 | 12.2 | 11.8 | 11.8 | 11.8 | 11.8 | 11.8 | 11.8 | 11.8 | 11.9 | 11.8 | 12.5 | 11.8 | 12.2 | 12.2 | 12.2 | 12.5 | 12.5 | 8.0 | 8.0 | 8.0 | 7.6 | 8.0 | 8.0 | 12.2 | 12.2 |
| 57 | *T. bicolor_*6 | 13.2 | 13.4 | 14.6 | 14.6 | 14.9 | 15.3 | 14.6 | 14.6 | 13.5 | 14.4 | 14.2 | 14.6 | 14.2 | 14.2 | 14.2 | 14.2 | 12.5 | 12.2 | 12.9 | 12.5 | 12.5 | 12.5 | 12.2 | 12.2 | 12.8 | 12.8 | 12.5 | 12.5 | 12.5 | 12.5 | 12.5 | 12.5 | 12.5 | 12.7 | 12.5 | 13.2 | 11.8 | 12.2 | 12.2 | 12.2 | 13.2 | 13.2 | 8.7 | 8.7 | 8.7 | 8.3 | 8.7 | 8.7 | 12.8 | 12.8 |
| 58 | *T. bicolor_*7 | 12.5 | 12.7 | 13.9 | 13.9 | 14.2 | 14.6 | 13.9 | 13.9 | 12.8 | 13.7 | 13.5 | 13.9 | 13.5 | 13.5 | 13.5 | 13.5 | 11.8 | 11.5 | 11.9 | 11.8 | 11.8 | 11.8 | 11.5 | 11.5 | 12.2 | 12.2 | 11.8 | 11.8 | 11.8 | 11.8 | 11.8 | 11.8 | 11.8 | 11.9 | 11.8 | 12.5 | 11.8 | 12.2 | 12.2 | 12.2 | 12.5 | 12.5 | 8.0 | 8.0 | 8.0 | 7.6 | 8.0 | 8.0 | 12.2 | 12.2 |
| 59 | *T. bicolor_*8 | 12.5 | 12.7 | 13.9 | 13.9 | 14.2 | 14.6 | 13.9 | 13.9 | 12.8 | 13.7 | 13.5 | 13.9 | 13.5 | 13.5 | 13.5 | 13.5 | 11.8 | 11.5 | 11.9 | 11.8 | 11.8 | 11.8 | 11.5 | 11.5 | 12.2 | 12.2 | 11.8 | 11.8 | 11.8 | 11.8 | 11.8 | 11.8 | 11.8 | 11.9 | 11.8 | 12.5 | 11.8 | 12.2 | 12.2 | 12.2 | 12.5 | 12.5 | 8.0 | 8.0 | 8.0 | 7.6 | 8.0 | 8.0 | 12.2 | 12.2 |
| 60 | *T. bicolor_*9 | 12.8 | 13.1 | 14.2 | 14.2 | 14.6 | 14.9 | 14.2 | 14.2 | 13.2 | 14.0 | 13.9 | 14.2 | 13.9 | 13.9 | 13.9 | 13.9 | 12.2 | 11.8 | 12.4 | 12.2 | 12.2 | 12.2 | 11.8 | 11.8 | 12.5 | 12.5 | 12.2 | 12.2 | 12.2 | 12.2 | 12.2 | 12.2 | 12.2 | 12.3 | 12.2 | 12.8 | 11.5 | 11.8 | 11.8 | 11.8 | 13.2 | 13.2 | 7.6 | 7.6 | 7.6 | 7.3 | 7.6 | 7.6 | 12.5 | 12.5 |
| 61 | *T. bicolor_*10 | 12.9 | 13.2 | 14.3 | 14.3 | 14.7 | 15.0 | 14.3 | 14.3 | 13.3 | 14.1 | 14.0 | 14.3 | 14.0 | 14.0 | 14.0 | 14.0 | 12.2 | 11.9 | 12.5 | 12.2 | 12.2 | 12.2 | 11.9 | 11.9 | 12.6 | 12.6 | 12.2 | 12.2 | 12.2 | 12.2 | 12.3 | 12.2 | 12.2 | 12.4 | 12.2 | 12.9 | 11.5 | 11.9 | 11.9 | 11.9 | 13.3 | 13.3 | 7.7 | 7.7 | 7.7 | 7.3 | 7.7 | 7.7 | 12.6 | 12.6 |
| 62 | *T. corticale_*1 | 16.7 | 17.5 | 16.7 | 16.7 | 17.0 | 17.4 | 17.4 | 16.7 | 15.6 | 16.9 | 16.3 | 16.7 | 16.3 | 16.3 | 16.3 | 16.3 | 16.0 | 15.6 | 16.7 | 15.6 | 15.6 | 16.0 | 15.6 | 15.6 | 16.3 | 16.3 | 16.0 | 16.0 | 16.0 | 16.0 | 16.0 | 16.0 | 16.0 | 16.8 | 16.0 | 16.7 | 12.5 | 12.5 | 12.5 | 12.5 | 13.5 | 13.5 | 10.4 | 10.4 | 10.4 | 10.1 | 10.4 | 10.4 | 13.5 | 14.2 |
| 63 | *T. corticale_*2 | 16.3 | 17.2 | 16.3 | 16.3 | 16.7 | 17.0 | 17.0 | 16.3 | 15.3 | 16.5 | 16.0 | 16.3 | 16.0 | 16.0 | 16.0 | 16.0 | 15.6 | 15.3 | 16.2 | 15.3 | 15.3 | 15.6 | 15.3 | 15.3 | 16.0 | 16.0 | 15.6 | 15.6 | 15.6 | 15.6 | 15.7 | 15.6 | 15.6 | 16.4 | 15.6 | 16.3 | 12.2 | 12.2 | 12.2 | 12.2 | 13.9 | 13.9 | 10.1 | 10.1 | 10.1 | 9.7 | 10.1 | 10.1 | 13.2 | 13.9 |
| 64 | *T. corticale_*3 | 17.4 | 18.3 | 17.4 | 17.4 | 17.7 | 18.1 | 18.1 | 17.4 | 16.3 | 16.9 | 16.3 | 16.7 | 16.3 | 16.3 | 16.3 | 16.3 | 16.0 | 15.6 | 16.2 | 15.6 | 15.6 | 16.7 | 16.3 | 16.3 | 17.0 | 17.0 | 16.7 | 16.7 | 16.7 | 16.7 | 16.7 | 16.7 | 16.7 | 17.5 | 16.7 | 17.4 | 11.8 | 12.5 | 12.5 | 12.5 | 13.9 | 13.9 | 10.8 | 10.8 | 10.8 | 10.4 | 10.8 | 10.8 | 14.2 | 14.9 |
| 65 | *T. corticale_*4 | 17.7 | 18.7 | 17.7 | 17.7 | 18.1 | 18.4 | 18.4 | 17.7 | 16.7 | 18.0 | 17.4 | 17.7 | 17.4 | 17.4 | 17.4 | 17.4 | 17.0 | 16.7 | 17.6 | 16.7 | 16.7 | 17.0 | 16.7 | 16.7 | 17.4 | 17.4 | 17.0 | 17.0 | 17.0 | 17.0 | 17.1 | 17.0 | 17.0 | 17.9 | 17.0 | 17.7 | 11.8 | 12.5 | 12.5 | 12.5 | 14.9 | 14.9 | 11.5 | 11.5 | 11.5 | 11.1 | 11.5 | 11.5 | 14.2 | 14.9 |
| 66 | *T. corticale_*5 | 17.7 | 18.7 | 17.7 | 17.7 | 18.1 | 18.4 | 18.4 | 17.7 | 16.7 | 18.0 | 17.4 | 17.7 | 17.4 | 17.4 | 17.4 | 17.4 | 17.0 | 16.7 | 17.6 | 16.7 | 16.7 | 17.0 | 16.7 | 16.7 | 17.4 | 17.4 | 17.0 | 17.0 | 17.0 | 17.0 | 17.1 | 17.0 | 17.0 | 17.9 | 17.0 | 17.7 | 11.8 | 12.5 | 12.5 | 12.5 | 14.9 | 14.9 | 11.5 | 11.5 | 11.5 | 11.1 | 11.5 | 11.5 | 14.2 | 14.9 |
| 67 | *T. corticale_*6 | 16.7 | 17.5 | 16.7 | 16.7 | 17.0 | 17.4 | 17.4 | 16.7 | 15.6 | 16.9 | 16.3 | 16.7 | 16.3 | 16.3 | 16.3 | 16.3 | 16.0 | 15.6 | 16.7 | 15.6 | 15.6 | 16.0 | 15.6 | 15.6 | 16.3 | 16.3 | 16.0 | 16.0 | 16.0 | 16.0 | 16.0 | 16.0 | 16.0 | 16.8 | 16.0 | 16.7 | 11.8 | 11.8 | 11.8 | 11.8 | 14.2 | 14.2 | 10.4 | 10.4 | 10.4 | 10.1 | 10.4 | 10.4 | 13.5 | 14.2 |
| 68 | *T. corticale_*7 | 16.7 | 17.5 | 16.7 | 16.7 | 17.0 | 17.4 | 17.4 | 16.7 | 15.6 | 16.9 | 16.3 | 16.7 | 16.3 | 16.3 | 16.3 | 16.3 | 16.0 | 15.6 | 16.7 | 15.6 | 15.6 | 16.0 | 15.6 | 15.6 | 16.3 | 16.3 | 16.0 | 16.0 | 16.0 | 16.0 | 16.0 | 16.0 | 16.0 | 16.8 | 16.0 | 16.7 | 11.8 | 11.8 | 11.8 | 11.8 | 14.2 | 14.2 | 10.4 | 10.4 | 10.4 | 10.1 | 10.4 | 10.4 | 13.5 | 14.2 |
| 69 | *T. corticale_*8 | 16.7 | 17.5 | 16.7 | 16.7 | 17.0 | 17.4 | 17.4 | 16.7 | 15.6 | 16.9 | 16.3 | 16.7 | 16.3 | 16.3 | 16.3 | 16.3 | 16.0 | 15.6 | 16.7 | 15.6 | 15.6 | 16.0 | 15.6 | 15.6 | 16.3 | 16.3 | 16.0 | 16.0 | 16.0 | 16.0 | 16.0 | 16.0 | 16.0 | 16.8 | 16.0 | 16.7 | 11.8 | 11.8 | 11.8 | 11.8 | 14.2 | 14.2 | 10.4 | 10.4 | 10.4 | 10.1 | 10.4 | 10.4 | 13.5 | 14.2 |
| 70 | *T. corticale_*9 | 16.7 | 17.5 | 16.7 | 16.7 | 17.0 | 17.4 | 17.4 | 16.7 | 15.6 | 16.9 | 16.3 | 16.7 | 16.3 | 16.3 | 16.3 | 16.3 | 16.0 | 15.6 | 16.7 | 15.6 | 15.6 | 16.0 | 15.6 | 15.6 | 16.3 | 16.3 | 16.0 | 16.0 | 16.0 | 16.0 | 16.0 | 16.0 | 16.0 | 16.8 | 16.0 | 16.7 | 11.8 | 11.8 | 11.8 | 11.8 | 14.2 | 14.2 | 10.4 | 10.4 | 10.4 | 10.1 | 10.4 | 10.4 | 13.5 | 14.2 |
| 71 | *T. corticale_*10 | 16.7 | 17.5 | 16.7 | 16.7 | 17.0 | 17.4 | 17.4 | 16.7 | 15.6 | 16.9 | 16.3 | 16.7 | 16.3 | 16.3 | 16.3 | 16.3 | 16.0 | 15.6 | 16.7 | 15.6 | 15.6 | 16.0 | 15.6 | 15.6 | 16.3 | 16.3 | 16.0 | 16.0 | 16.0 | 16.0 | 16.0 | 16.0 | 16.0 | 16.8 | 16.0 | 16.7 | 11.8 | 11.8 | 11.8 | 11.8 | 14.2 | 14.2 | 10.4 | 10.4 | 10.4 | 10.1 | 10.4 | 10.4 | 13.5 | 14.2 |
| 72 | *T. corticale_*11 | 16.7 | 17.5 | 16.7 | 16.7 | 17.0 | 17.4 | 17.4 | 16.7 | 15.6 | 16.9 | 16.3 | 16.7 | 16.3 | 16.3 | 16.3 | 16.3 | 16.0 | 15.6 | 16.7 | 15.6 | 15.6 | 16.0 | 15.6 | 15.6 | 16.3 | 16.3 | 16.0 | 16.0 | 16.0 | 16.0 | 16.0 | 16.0 | 16.0 | 16.8 | 16.0 | 16.7 | 11.8 | 11.8 | 11.8 | 11.8 | 14.2 | 14.2 | 10.4 | 10.4 | 10.4 | 10.1 | 10.4 | 10.4 | 13.5 | 14.2 |
| 73 | *T. corticale_*12 | 16.7 | 17.5 | 16.7 | 16.7 | 17.0 | 17.4 | 17.4 | 16.7 | 15.6 | 16.9 | 16.3 | 16.7 | 16.3 | 16.3 | 16.3 | 16.3 | 16.0 | 15.6 | 16.7 | 15.6 | 15.6 | 16.0 | 15.6 | 15.6 | 16.3 | 16.3 | 16.0 | 16.0 | 16.0 | 16.0 | 16.0 | 16.0 | 16.0 | 16.8 | 16.0 | 16.7 | 11.8 | 11.8 | 11.8 | 11.8 | 14.2 | 14.2 | 10.4 | 10.4 | 10.4 | 10.1 | 10.4 | 10.4 | 13.5 | 14.2 |
| 74 | *T. corticale_*13 | 16.7 | 17.5 | 16.7 | 16.7 | 17.0 | 17.4 | 17.4 | 16.7 | 15.6 | 16.9 | 16.3 | 16.7 | 16.3 | 16.3 | 16.3 | 16.3 | 16.0 | 15.6 | 16.7 | 15.6 | 15.6 | 16.0 | 15.6 | 15.6 | 16.3 | 16.3 | 16.0 | 16.0 | 16.0 | 16.0 | 16.0 | 16.0 | 16.0 | 16.8 | 16.0 | 16.7 | 11.8 | 11.8 | 11.8 | 11.8 | 14.2 | 14.2 | 10.4 | 10.4 | 10.4 | 10.1 | 10.4 | 10.4 | 13.5 | 14.2 |
| 75 | *T. corticale_*14 | 16.7 | 17.5 | 16.7 | 16.7 | 17.0 | 17.4 | 17.4 | 16.7 | 15.6 | 16.9 | 16.3 | 16.7 | 16.3 | 16.3 | 16.3 | 16.3 | 16.0 | 15.6 | 16.7 | 15.6 | 15.6 | 16.0 | 15.6 | 15.6 | 16.3 | 16.3 | 16.0 | 16.0 | 16.0 | 16.0 | 16.0 | 16.0 | 16.0 | 16.8 | 16.0 | 16.7 | 11.8 | 11.8 | 11.8 | 11.8 | 14.2 | 14.2 | 10.4 | 10.4 | 10.4 | 10.1 | 10.4 | 10.4 | 13.5 | 14.2 |
| 76 | *T. corticale_*15 | 16.7 | 17.5 | 16.7 | 16.7 | 17.0 | 17.4 | 17.4 | 16.7 | 15.6 | 16.9 | 16.3 | 16.7 | 16.3 | 16.3 | 16.3 | 16.3 | 16.0 | 15.6 | 16.7 | 15.6 | 15.6 | 16.0 | 15.6 | 15.6 | 16.3 | 16.3 | 16.0 | 16.0 | 16.0 | 16.0 | 16.0 | 16.0 | 16.0 | 16.8 | 16.0 | 16.7 | 11.8 | 11.8 | 11.8 | 11.8 | 14.2 | 14.2 | 10.4 | 10.4 | 10.4 | 10.1 | 10.4 | 10.4 | 13.5 | 14.2 |
| 77 | *T. corticale_*16 | 16.7 | 17.5 | 16.7 | 16.7 | 17.0 | 17.4 | 17.4 | 16.7 | 15.6 | 16.9 | 16.3 | 16.7 | 16.3 | 16.3 | 16.3 | 16.3 | 16.0 | 15.6 | 16.7 | 15.6 | 15.6 | 16.0 | 15.6 | 15.6 | 16.3 | 16.3 | 16.0 | 16.0 | 16.0 | 16.0 | 16.0 | 16.0 | 16.0 | 16.8 | 16.0 | 16.7 | 11.8 | 11.8 | 11.8 | 11.8 | 14.2 | 14.2 | 10.4 | 10.4 | 10.4 | 10.1 | 10.4 | 10.4 | 13.5 | 14.2 |
| 78 | *T. corticale_*17 | 16.7 | 17.5 | 16.7 | 16.7 | 17.0 | 17.4 | 17.4 | 16.7 | 15.6 | 16.9 | 16.3 | 16.7 | 16.3 | 16.3 | 16.3 | 16.3 | 16.0 | 15.6 | 16.7 | 15.6 | 15.6 | 16.0 | 15.6 | 15.6 | 16.3 | 16.3 | 16.0 | 16.0 | 16.0 | 16.0 | 16.0 | 16.0 | 16.0 | 16.8 | 16.0 | 16.7 | 11.8 | 11.8 | 11.8 | 11.8 | 14.2 | 14.2 | 10.4 | 10.4 | 10.4 | 10.1 | 10.4 | 10.4 | 13.5 | 14.2 |
| 79 | *T. corticale_*18 | 16.7 | 17.5 | 16.7 | 16.7 | 17.0 | 17.4 | 17.4 | 16.7 | 15.6 | 16.9 | 16.3 | 16.7 | 16.3 | 16.3 | 16.3 | 16.3 | 16.0 | 15.6 | 16.7 | 15.6 | 15.6 | 16.0 | 15.6 | 15.6 | 16.3 | 16.3 | 16.0 | 16.0 | 16.0 | 16.0 | 16.0 | 16.0 | 16.0 | 16.8 | 16.0 | 16.7 | 11.8 | 11.8 | 11.8 | 11.8 | 14.2 | 14.2 | 10.4 | 10.4 | 10.4 | 10.1 | 10.4 | 10.4 | 13.5 | 14.2 |
| 80 | *T. corticale_*19 | 17.0 | 17.9 | 17.0 | 17.0 | 17.4 | 17.7 | 17.7 | 17.0 | 16.0 | 17.3 | 16.7 | 17.0 | 16.7 | 16.7 | 16.7 | 16.7 | 16.3 | 16.0 | 17.1 | 16.0 | 16.0 | 16.3 | 16.0 | 16.0 | 16.7 | 16.7 | 16.3 | 16.3 | 16.3 | 16.3 | 16.4 | 16.3 | 16.3 | 17.2 | 16.3 | 17.0 | 12.2 | 12.2 | 12.2 | 12.2 | 14.6 | 14.6 | 10.8 | 10.8 | 10.8 | 10.4 | 10.8 | 10.8 | 13.9 | 14.6 |
| 81 | *T. corticale_*20 | 16.7 | 17.5 | 16.7 | 16.7 | 17.0 | 17.4 | 17.4 | 16.7 | 15.6 | 16.9 | 16.3 | 16.7 | 16.3 | 16.3 | 16.3 | 16.3 | 16.0 | 15.6 | 16.7 | 15.6 | 15.6 | 16.0 | 15.6 | 15.6 | 16.3 | 16.3 | 16.0 | 16.0 | 16.0 | 16.0 | 16.0 | 16.0 | 16.0 | 16.8 | 16.0 | 16.7 | 11.8 | 11.8 | 11.8 | 11.8 | 14.2 | 14.2 | 10.4 | 10.4 | 10.4 | 10.1 | 10.4 | 10.4 | 13.5 | 14.2 |
| 82 | *T. corticale_*21 | 16.7 | 17.5 | 16.7 | 16.7 | 17.0 | 17.4 | 17.4 | 16.7 | 15.6 | 16.9 | 16.3 | 16.7 | 16.3 | 16.3 | 16.3 | 16.3 | 16.0 | 15.6 | 16.7 | 15.6 | 15.6 | 16.0 | 15.6 | 15.6 | 16.3 | 16.3 | 16.0 | 16.0 | 16.0 | 16.0 | 16.0 | 16.0 | 16.0 | 16.8 | 16.0 | 16.7 | 11.8 | 11.8 | 11.8 | 11.8 | 14.2 | 14.2 | 10.4 | 10.4 | 10.4 | 10.1 | 10.4 | 10.4 | 13.5 | 14.2 |
| 83 | *T. corticale_*22 | 16.7 | 17.5 | 16.7 | 16.7 | 17.0 | 17.4 | 17.4 | 16.7 | 15.6 | 16.9 | 16.3 | 16.7 | 16.3 | 16.3 | 16.3 | 16.3 | 16.0 | 15.6 | 16.7 | 15.6 | 15.6 | 16.0 | 15.6 | 15.6 | 16.3 | 16.3 | 16.0 | 16.0 | 16.0 | 16.0 | 16.0 | 16.0 | 16.0 | 16.8 | 16.0 | 16.7 | 11.8 | 11.8 | 11.8 | 11.8 | 14.2 | 14.2 | 10.4 | 10.4 | 10.4 | 10.1 | 10.4 | 10.4 | 13.5 | 14.2 |
| 84 | *T. corticale_*23 | 16.7 | 17.5 | 16.7 | 16.7 | 17.0 | 17.4 | 17.4 | 16.7 | 15.6 | 16.9 | 16.3 | 16.7 | 16.3 | 16.3 | 16.3 | 16.3 | 16.0 | 15.6 | 16.7 | 15.6 | 15.6 | 16.0 | 15.6 | 15.6 | 16.3 | 16.3 | 16.0 | 16.0 | 16.0 | 16.0 | 16.0 | 16.0 | 16.0 | 16.8 | 16.0 | 16.7 | 11.8 | 11.8 | 11.8 | 11.8 | 14.2 | 14.2 | 10.4 | 10.4 | 10.4 | 10.1 | 10.4 | 10.4 | 13.5 | 14.2 |
| 85 | *T. gordoni_*1 | 13.9 | 14.2 | 14.9 | 14.9 | 15.3 | 15.3 | 14.6 | 14.6 | 14.2 | 14.7 | 14.2 | 14.6 | 13.9 | 13.9 | 13.9 | 13.9 | 12.5 | 11.8 | 11.9 | 12.2 | 12.2 | 12.5 | 12.5 | 12.5 | 12.8 | 12.8 | 12.5 | 12.5 | 12.5 | 12.5 | 12.5 | 12.5 | 12.5 | 13.4 | 12.5 | 13.2 | 11.5 | 12.2 | 12.2 | 12.2 | 12.5 | 12.5 | 10.4 | 10.4 | 10.4 | 10.1 | 10.4 | 10.4 | 10.8 | 11.1 |
| 86 | *T. gordoni_*2 | 13.5 | 13.9 | 15.2 | 15.2 | 15.6 | 15.6 | 15.6 | 15.6 | 14.5 | 15.5 | 15.2 | 15.6 | 14.9 | 14.9 | 14.9 | 14.9 | 13.5 | 12.8 | 13.2 | 13.1 | 13.1 | 13.5 | 12.8 | 12.8 | 13.8 | 13.8 | 13.5 | 13.5 | 13.5 | 13.5 | 13.5 | 13.5 | 13.5 | 13.9 | 13.5 | 13.5 | 13.8 | 13.5 | 13.5 | 13.5 | 13.5 | 13.5 | 11.0 | 11.0 | 11.0 | 10.6 | 11.0 | 11.0 | 10.3 | 11.0 |
| 87 | *T. gordoni_*3 | 14.3 | 15.0 | 15.7 | 15.7 | 16.1 | 16.8 | 16.4 | 16.1 | 15.0 | 16.3 | 15.7 | 16.1 | 15.4 | 15.4 | 15.4 | 15.4 | 14.3 | 13.6 | 14.4 | 14.0 | 14.0 | 14.3 | 13.6 | 13.6 | 14.7 | 14.7 | 14.3 | 14.3 | 14.3 | 14.3 | 14.4 | 14.3 | 14.3 | 15.0 | 14.3 | 15.0 | 12.9 | 12.9 | 12.9 | 12.9 | 13.6 | 13.6 | 10.8 | 10.8 | 10.8 | 10.5 | 10.8 | 10.8 | 10.5 | 11.2 |
| 88 | *T. gordoni_*4 | 14.0 | 14.7 | 15.4 | 15.4 | 15.7 | 16.4 | 16.1 | 15.7 | 14.7 | 15.9 | 15.4 | 15.7 | 15.0 | 15.0 | 15.0 | 15.0 | 14.0 | 13.3 | 13.9 | 13.6 | 13.6 | 14.0 | 13.3 | 13.3 | 14.3 | 14.3 | 14.0 | 14.0 | 14.0 | 14.0 | 14.0 | 14.0 | 14.0 | 14.7 | 14.0 | 14.7 | 12.6 | 12.6 | 12.6 | 12.6 | 13.3 | 13.3 | 10.8 | 10.8 | 10.8 | 10.5 | 10.8 | 10.8 | 10.1 | 10.8 |
| 89 | *T. gordoni_*5 | 14.0 | 14.7 | 15.4 | 15.4 | 15.8 | 16.1 | 15.8 | 15.4 | 14.7 | 15.6 | 15.1 | 15.4 | 14.7 | 14.7 | 14.7 | 14.7 | 13.7 | 13.0 | 13.9 | 13.3 | 13.3 | 13.7 | 13.0 | 13.0 | 14.0 | 14.0 | 13.7 | 13.7 | 13.7 | 13.7 | 13.7 | 13.7 | 13.7 | 14.7 | 13.7 | 14.4 | 12.3 | 12.3 | 12.3 | 12.3 | 13.0 | 13.0 | 10.5 | 10.5 | 10.5 | 10.2 | 10.5 | 10.5 | 10.2 | 10.9 |
| 90 | *T. gordoni_*6 | 13.7 | 14.4 | 15.5 | 15.5 | 15.8 | 16.2 | 16.2 | 15.5 | 14.8 | 16.1 | 15.5 | 15.8 | 15.1 | 15.1 | 15.1 | 15.1 | 14.1 | 13.4 | 14.1 | 13.7 | 13.7 | 14.1 | 13.4 | 13.4 | 14.4 | 14.4 | 14.1 | 14.1 | 14.1 | 14.1 | 14.1 | 14.1 | 14.1 | 14.8 | 14.1 | 14.8 | 13.0 | 12.7 | 12.7 | 12.7 | 13.4 | 13.4 | 10.6 | 10.6 | 10.6 | 10.2 | 10.6 | 10.6 | 9.5 | 10.2 |
| 91 | *T. gordoni_*7 | 14.0 | 14.7 | 15.4 | 15.4 | 15.7 | 16.4 | 16.1 | 15.7 | 14.7 | 15.9 | 15.4 | 15.7 | 15.0 | 15.0 | 15.0 | 15.0 | 14.0 | 13.3 | 14.4 | 13.6 | 13.6 | 14.0 | 13.3 | 13.3 | 14.3 | 14.3 | 14.0 | 14.0 | 14.0 | 14.0 | 13.7 | 14.0 | 14.0 | 14.7 | 14.0 | 14.7 | 13.3 | 12.6 | 12.6 | 12.6 | 13.3 | 13.3 | 10.8 | 10.8 | 10.8 | 10.5 | 10.8 | 10.8 | 10.1 | 10.8 |
| 92 | *T. gordoni_*8 | 14.0 | 14.7 | 15.4 | 15.4 | 15.7 | 16.4 | 16.1 | 15.7 | 14.7 | 15.9 | 15.4 | 15.7 | 15.0 | 15.0 | 15.0 | 15.0 | 14.0 | 13.3 | 14.4 | 13.6 | 13.6 | 14.0 | 13.3 | 13.3 | 14.3 | 14.3 | 14.0 | 14.0 | 14.0 | 14.0 | 13.7 | 14.0 | 14.0 | 14.7 | 14.0 | 14.7 | 13.3 | 12.6 | 12.6 | 12.6 | 13.3 | 13.3 | 10.8 | 10.8 | 10.8 | 10.5 | 10.8 | 10.8 | 10.1 | 10.8 |
| 93 | *T. hekouense_*1 | 12.8 | 13.4 | 14.6 | 14.6 | 14.9 | 15.3 | 14.2 | 14.2 | 12.8 | 14.7 | 14.2 | 14.6 | 14.2 | 14.2 | 14.2 | 14.2 | 12.5 | 12.2 | 12.9 | 12.5 | 12.5 | 12.5 | 12.2 | 12.2 | 12.2 | 12.2 | 12.5 | 12.5 | 12.5 | 12.5 | 12.5 | 12.5 | 12.5 | 13.1 | 12.5 | 13.2 | 15.3 | 15.6 | 15.6 | 15.6 | 11.8 | 11.8 | 9.7 | 9.7 | 9.7 | 9.4 | 9.7 | 9.7 | 11.8 | 11.1 |
| 94 | *T. hekouense_*2 | 12.8 | 13.4 | 14.6 | 14.6 | 14.9 | 15.3 | 14.2 | 14.2 | 12.8 | 14.7 | 14.2 | 14.6 | 14.2 | 14.2 | 14.2 | 14.2 | 12.5 | 12.2 | 12.9 | 12.5 | 12.5 | 12.5 | 12.2 | 12.2 | 12.2 | 12.2 | 12.5 | 12.5 | 12.5 | 12.5 | 12.5 | 12.5 | 12.5 | 13.1 | 12.5 | 13.2 | 15.3 | 15.6 | 15.6 | 15.6 | 11.8 | 11.8 | 9.7 | 9.7 | 9.7 | 9.4 | 9.7 | 9.7 | 11.8 | 11.1 |
| 95 | *T. hekouense_*3 | 12.5 | 13.1 | 14.2 | 14.2 | 14.6 | 14.9 | 13.9 | 13.9 | 12.5 | 14.4 | 13.9 | 14.2 | 13.9 | 13.9 | 13.9 | 13.9 | 12.2 | 11.8 | 12.4 | 12.2 | 12.2 | 12.2 | 11.8 | 11.8 | 11.8 | 11.8 | 12.2 | 12.2 | 12.2 | 12.2 | 12.2 | 12.2 | 12.2 | 12.7 | 12.2 | 12.8 | 15.3 | 15.6 | 15.6 | 15.6 | 11.5 | 11.5 | 9.4 | 9.4 | 9.4 | 9.0 | 9.4 | 9.4 | 11.8 | 11.1 |
| 96 | *T. hekouense_*4 | 12.5 | 13.1 | 14.2 | 14.2 | 14.6 | 14.9 | 13.9 | 13.9 | 12.5 | 14.4 | 13.9 | 14.2 | 13.9 | 13.9 | 13.9 | 13.9 | 12.2 | 11.8 | 12.4 | 12.2 | 12.2 | 12.2 | 11.8 | 11.8 | 11.8 | 11.8 | 12.2 | 12.2 | 12.2 | 12.2 | 12.2 | 12.2 | 12.2 | 12.7 | 12.2 | 12.8 | 15.3 | 15.6 | 15.6 | 15.6 | 11.5 | 11.5 | 9.4 | 9.4 | 9.4 | 9.0 | 9.4 | 9.4 | 11.8 | 11.1 |
| 97 | *T. hekouense_*5 | 12.8 | 13.4 | 14.6 | 14.6 | 14.9 | 15.3 | 14.2 | 14.2 | 12.8 | 14.7 | 14.2 | 14.6 | 14.2 | 14.2 | 14.2 | 14.2 | 12.5 | 12.2 | 12.9 | 12.5 | 12.5 | 12.5 | 12.2 | 12.2 | 12.2 | 12.2 | 12.5 | 12.5 | 12.5 | 12.5 | 12.5 | 12.5 | 12.5 | 13.1 | 12.5 | 13.2 | 15.3 | 15.6 | 15.6 | 15.6 | 11.8 | 11.8 | 9.7 | 9.7 | 9.7 | 9.4 | 9.7 | 9.7 | 11.8 | 11.1 |
| 98 | *T. hekouense_*6 | 12.8 | 13.4 | 14.6 | 14.6 | 14.9 | 15.3 | 14.2 | 14.2 | 12.8 | 14.7 | 14.2 | 14.6 | 14.2 | 14.2 | 14.2 | 14.2 | 12.5 | 12.2 | 12.9 | 12.5 | 12.5 | 12.5 | 12.2 | 12.2 | 12.2 | 12.2 | 12.5 | 12.5 | 12.5 | 12.5 | 12.5 | 12.5 | 12.5 | 13.1 | 12.5 | 13.2 | 15.3 | 15.6 | 15.6 | 15.6 | 11.8 | 11.8 | 9.7 | 9.7 | 9.7 | 9.4 | 9.7 | 9.7 | 11.8 | 11.1 |
| 99 | *T. hekouense_*7 | 13.2 | 13.8 | 14.9 | 14.9 | 15.3 | 15.6 | 14.6 | 14.6 | 13.2 | 15.1 | 14.6 | 14.9 | 14.6 | 14.6 | 14.6 | 14.6 | 12.8 | 12.5 | 13.3 | 12.8 | 12.8 | 12.8 | 12.5 | 12.5 | 12.5 | 12.5 | 12.8 | 12.8 | 12.8 | 12.8 | 12.9 | 12.8 | 12.8 | 13.4 | 12.8 | 13.5 | 15.6 | 16.0 | 16.0 | 16.0 | 12.2 | 12.2 | 10.1 | 10.1 | 10.1 | 9.7 | 10.1 | 10.1 | 12.2 | 11.5 |
| 100 | *T. hekouense_*8 | 12.5 | 13.1 | 14.2 | 14.2 | 14.6 | 14.9 | 13.9 | 13.9 | 12.5 | 14.4 | 13.9 | 14.2 | 13.9 | 13.9 | 13.9 | 13.9 | 12.2 | 11.8 | 12.4 | 12.2 | 12.2 | 12.2 | 11.8 | 11.8 | 11.8 | 11.8 | 12.2 | 12.2 | 12.2 | 12.2 | 12.2 | 12.2 | 12.2 | 12.7 | 12.2 | 12.8 | 14.9 | 15.3 | 15.3 | 15.3 | 11.5 | 11.5 | 9.0 | 9.0 | 9.0 | 8.7 | 9.0 | 9.0 | 11.8 | 11.1 |
| 101 | *T. hekouense_*9 | 12.5 | 13.1 | 14.2 | 14.2 | 14.6 | 14.9 | 13.9 | 13.9 | 12.5 | 14.4 | 13.9 | 14.2 | 13.9 | 13.9 | 13.9 | 13.9 | 12.2 | 11.8 | 12.4 | 12.2 | 12.2 | 12.2 | 11.8 | 11.8 | 11.8 | 11.8 | 12.2 | 12.2 | 12.2 | 12.2 | 12.2 | 12.2 | 12.2 | 12.7 | 12.2 | 12.8 | 15.3 | 15.6 | 15.6 | 15.6 | 11.5 | 11.5 | 9.4 | 9.4 | 9.4 | 9.0 | 9.4 | 9.4 | 11.8 | 11.1 |
| 102 | *T. hekouense_*10 | 12.5 | 13.1 | 14.2 | 14.2 | 14.6 | 14.9 | 13.9 | 13.9 | 12.5 | 14.4 | 13.9 | 14.2 | 13.9 | 13.9 | 13.9 | 13.9 | 12.2 | 11.8 | 12.4 | 12.2 | 12.2 | 12.2 | 11.8 | 11.8 | 11.8 | 11.8 | 12.2 | 12.2 | 12.2 | 12.2 | 12.2 | 12.2 | 12.2 | 12.7 | 12.2 | 12.8 | 15.3 | 15.6 | 15.6 | 15.6 | 11.5 | 11.5 | 9.4 | 9.4 | 9.4 | 9.0 | 9.4 | 9.4 | 11.8 | 11.1 |
| 103 | *T. horridum_*1 | 18.8 | 19.4 | 19.4 | 19.4 | 19.8 | 20.1 | 20.1 | 19.4 | 18.1 | 19.8 | 19.4 | 19.8 | 19.4 | 19.4 | 19.4 | 19.4 | 18.1 | 18.1 | 19.5 | 18.4 | 18.4 | 18.1 | 17.7 | 17.7 | 17.7 | 17.7 | 18.1 | 18.1 | 18.1 | 18.1 | 18.1 | 18.1 | 18.1 | 18.7 | 18.1 | 18.8 | 17.7 | 18.1 | 18.1 | 18.1 | 17.7 | 17.7 | 16.7 | 16.7 | 16.7 | 16.3 | 16.7 | 16.7 | 18.1 | 18.1 |
| 104 | *T. horridum_*2 | 18.8 | 19.4 | 19.4 | 19.4 | 19.8 | 20.1 | 20.1 | 19.4 | 18.1 | 19.8 | 19.4 | 19.8 | 19.4 | 19.4 | 19.4 | 19.4 | 18.1 | 18.1 | 19.5 | 18.4 | 18.4 | 18.1 | 17.7 | 17.7 | 17.7 | 17.7 | 18.1 | 18.1 | 18.1 | 18.1 | 18.1 | 18.1 | 18.1 | 18.7 | 18.1 | 18.8 | 17.7 | 18.1 | 18.1 | 18.1 | 17.7 | 17.7 | 16.7 | 16.7 | 16.7 | 16.3 | 16.7 | 16.7 | 18.1 | 18.1 |
| 105 | *T. horridum_*3 | 18.8 | 19.4 | 19.4 | 19.4 | 19.8 | 20.1 | 20.1 | 19.4 | 18.1 | 19.8 | 19.4 | 19.8 | 19.4 | 19.4 | 19.4 | 19.4 | 18.1 | 18.1 | 20.0 | 18.4 | 18.4 | 18.1 | 17.7 | 17.7 | 17.7 | 17.7 | 18.1 | 18.1 | 18.1 | 18.1 | 18.1 | 18.1 | 18.1 | 18.7 | 18.1 | 18.8 | 17.7 | 18.1 | 18.1 | 18.1 | 17.7 | 17.7 | 16.0 | 16.0 | 16.0 | 15.6 | 16.0 | 16.0 | 17.4 | 17.4 |
| 106 | *T. horridum5* | 19.1 | 19.8 | 19.8 | 19.8 | 20.1 | 20.1 | 20.5 | 19.8 | 18.4 | 20.1 | 19.8 | 20.1 | 19.8 | 19.8 | 19.8 | 19.8 | 18.4 | 18.4 | 20.0 | 18.8 | 18.8 | 18.4 | 18.1 | 18.1 | 18.1 | 18.1 | 18.4 | 18.4 | 18.4 | 18.4 | 18.5 | 18.4 | 18.4 | 19.0 | 18.4 | 19.1 | 18.4 | 18.8 | 18.8 | 18.8 | 18.1 | 18.1 | 16.7 | 16.7 | 16.7 | 16.3 | 16.7 | 16.7 | 17.7 | 17.7 |
| 107 | *T. khoii_*1 | 14.2 | 14.2 | 14.9 | 14.9 | 15.3 | 14.9 | 14.2 | 14.2 | 13.9 | 14.0 | 13.9 | 14.2 | 13.9 | 13.9 | 13.9 | 13.9 | 12.8 | 12.5 | 12.9 | 12.5 | 12.5 | 12.8 | 12.5 | 12.5 | 13.2 | 13.2 | 12.8 | 12.8 | 12.8 | 12.8 | 12.9 | 12.8 | 12.8 | 13.4 | 12.8 | 13.5 | 11.1 | 12.2 | 12.2 | 12.2 | 13.9 | 13.9 | 8.7 | 8.7 | 8.7 | 8.3 | 8.7 | 8.7 | 13.5 | 13.5 |
| 108 | *T. khoii*2 | 14.2 | 14.2 | 14.9 | 14.9 | 15.3 | 14.9 | 14.2 | 14.2 | 13.9 | 14.0 | 13.9 | 14.2 | 13.9 | 13.9 | 13.9 | 13.9 | 12.8 | 12.5 | 12.9 | 12.5 | 12.5 | 12.8 | 12.5 | 12.5 | 13.2 | 13.2 | 12.8 | 12.8 | 12.8 | 12.8 | 12.9 | 12.8 | 12.8 | 13.4 | 12.8 | 13.5 | 11.1 | 12.2 | 12.2 | 12.2 | 13.9 | 13.9 | 8.7 | 8.7 | 8.7 | 8.3 | 8.7 | 8.7 | 13.5 | 13.5 |
| 109 | *T. lacustrinum_*1 | 14.9 | 15.3 | 15.6 | 15.6 | 16.0 | 15.6 | 16.0 | 15.6 | 13.9 | 15.1 | 14.9 | 15.3 | 14.9 | 14.9 | 14.9 | 14.9 | 14.6 | 14.6 | 14.8 | 14.9 | 14.9 | 13.9 | 13.5 | 13.5 | 14.2 | 14.2 | 13.9 | 13.9 | 13.9 | 13.9 | 13.6 | 13.9 | 13.9 | 14.2 | 13.9 | 14.6 | 12.2 | 11.8 | 11.8 | 11.8 | 14.2 | 14.2 | 10.1 | 10.1 | 10.1 | 9.7 | 10.1 | 10.1 | 13.5 | 13.5 |
| 110 | *T. lacustrinum_*2 | 14.9 | 15.3 | 15.6 | 15.6 | 16.0 | 15.6 | 16.0 | 15.6 | 13.9 | 15.1 | 14.9 | 15.3 | 14.9 | 14.9 | 14.9 | 14.9 | 14.6 | 14.6 | 14.8 | 14.9 | 14.9 | 13.9 | 13.5 | 13.5 | 14.2 | 14.2 | 13.9 | 13.9 | 13.9 | 13.9 | 13.6 | 13.9 | 13.9 | 14.2 | 13.9 | 14.6 | 12.2 | 11.8 | 11.8 | 11.8 | 14.2 | 14.2 | 10.1 | 10.1 | 10.1 | 9.7 | 10.1 | 10.1 | 13.5 | 13.5 |
| 111 | *T. laeve_*1 | 13.5 | 13.8 | 14.2 | 14.2 | 14.6 | 14.6 | 13.9 | 13.9 | 12.8 | 13.7 | 13.2 | 13.5 | 12.8 | 12.8 | 12.8 | 12.8 | 12.5 | 12.2 | 12.4 | 12.5 | 12.5 | 12.5 | 12.5 | 12.5 | 12.8 | 12.8 | 12.5 | 12.5 | 12.5 | 12.5 | 12.5 | 12.5 | 12.5 | 13.4 | 12.5 | 13.2 | 12.8 | 11.8 | 11.8 | 11.8 | 12.2 | 12.2 | 13.2 | 13.2 | 13.2 | 12.8 | 13.2 | 13.2 | 13.2 | 13.2 |
| 112 | *T. laeve_*2 | 15.3 | 15.7 | 16.0 | 16.0 | 16.3 | 16.3 | 14.9 | 15.3 | 13.9 | 15.1 | 14.6 | 14.9 | 14.6 | 14.6 | 14.6 | 14.6 | 13.2 | 13.5 | 13.8 | 13.5 | 13.5 | 13.2 | 13.5 | 13.5 | 13.5 | 13.5 | 13.2 | 13.2 | 13.2 | 13.2 | 13.2 | 13.2 | 13.2 | 14.2 | 13.2 | 13.9 | 14.9 | 14.6 | 14.6 | 14.6 | 14.9 | 14.9 | 16.7 | 16.7 | 16.7 | 16.3 | 16.7 | 16.7 | 15.3 | 15.6 |
| 113 | *T. laeve_*3 | 15.3 | 15.7 | 16.0 | 16.0 | 16.3 | 16.3 | 14.9 | 15.3 | 13.9 | 15.1 | 14.6 | 14.9 | 14.6 | 14.6 | 14.6 | 14.6 | 13.2 | 13.5 | 13.3 | 13.5 | 13.5 | 13.2 | 13.5 | 13.5 | 13.5 | 13.5 | 13.2 | 13.2 | 13.2 | 13.2 | 13.2 | 13.2 | 13.2 | 14.2 | 13.2 | 13.9 | 14.9 | 14.6 | 14.6 | 14.6 | 14.9 | 14.9 | 16.7 | 16.7 | 16.7 | 16.3 | 16.0 | 16.0 | 15.3 | 15.6 |
| 114 | *T. laeve_*4 | 15.6 | 16.0 | 16.3 | 16.3 | 16.7 | 16.7 | 15.3 | 15.6 | 14.2 | 15.5 | 14.9 | 15.3 | 14.9 | 14.9 | 14.9 | 14.9 | 13.5 | 13.9 | 13.8 | 13.9 | 13.9 | 13.5 | 13.9 | 13.9 | 13.9 | 13.9 | 13.5 | 13.5 | 13.5 | 13.5 | 13.6 | 13.5 | 13.5 | 14.6 | 13.5 | 14.2 | 15.6 | 15.3 | 15.3 | 15.3 | 15.3 | 15.3 | 17.4 | 17.4 | 17.4 | 17.0 | 17.4 | 17.4 | 15.6 | 16.0 |
| 115 | *T. laeve_*5 | 14.6 | 14.9 | 15.3 | 15.3 | 15.6 | 15.6 | 14.2 | 14.6 | 13.9 | 14.4 | 13.9 | 14.2 | 13.9 | 13.9 | 13.9 | 13.9 | 12.5 | 12.8 | 12.9 | 12.8 | 12.8 | 12.5 | 12.8 | 12.8 | 12.8 | 12.8 | 12.5 | 12.5 | 12.5 | 12.5 | 12.5 | 12.5 | 12.5 | 13.4 | 12.5 | 13.2 | 16.0 | 15.6 | 15.6 | 15.6 | 14.9 | 14.9 | 17.7 | 17.7 | 17.7 | 17.4 | 17.7 | 17.7 | 15.3 | 15.6 |
| 116 | *T. laeve_*6 | 14.6 | 14.9 | 15.3 | 15.3 | 15.6 | 15.6 | 14.2 | 14.6 | 13.9 | 14.4 | 13.9 | 14.2 | 13.9 | 13.9 | 13.9 | 13.9 | 12.5 | 12.8 | 12.9 | 12.8 | 12.8 | 12.5 | 12.8 | 12.8 | 12.8 | 12.8 | 12.5 | 12.5 | 12.5 | 12.5 | 12.5 | 12.5 | 12.5 | 13.4 | 12.5 | 13.2 | 16.0 | 15.6 | 15.6 | 15.6 | 14.9 | 14.9 | 17.7 | 17.7 | 17.7 | 17.4 | 17.7 | 17.7 | 15.3 | 15.6 |
| 117 | *T. laeve_*7 | 14.6 | 14.9 | 15.3 | 15.3 | 15.6 | 15.6 | 14.2 | 14.6 | 13.9 | 14.4 | 13.9 | 14.2 | 13.9 | 13.9 | 13.9 | 13.9 | 12.5 | 12.8 | 12.9 | 12.8 | 12.8 | 12.5 | 12.8 | 12.8 | 12.8 | 12.8 | 12.5 | 12.5 | 12.5 | 12.5 | 12.5 | 12.5 | 12.5 | 13.4 | 12.5 | 13.2 | 16.0 | 15.6 | 15.6 | 15.6 | 14.9 | 14.9 | 17.7 | 17.7 | 17.7 | 17.4 | 17.7 | 17.7 | 15.3 | 15.6 |
| 118 | *T. laeve_*8 | 16.0 | 16.4 | 16.7 | 16.7 | 17.0 | 17.0 | 15.6 | 16.0 | 14.6 | 15.8 | 15.3 | 15.6 | 15.3 | 15.3 | 15.3 | 15.3 | 13.9 | 14.2 | 14.3 | 14.2 | 14.2 | 13.9 | 14.2 | 14.2 | 14.2 | 14.2 | 13.9 | 13.9 | 13.9 | 13.9 | 13.9 | 13.9 | 13.9 | 14.9 | 13.9 | 14.6 | 16.0 | 15.6 | 15.6 | 15.6 | 15.6 | 15.6 | 17.7 | 17.7 | 17.7 | 17.4 | 17.7 | 17.7 | 16.0 | 16.3 |
| 119 | *T. laeve_*9 | 17.3 | 17.1 | 18.5 | 18.5 | 18.9 | 18.1 | 17.3 | 17.7 | 16.1 | 16.9 | 16.9 | 16.9 | 16.9 | 16.9 | 16.9 | 16.9 | 15.4 | 15.7 | 16.5 | 15.7 | 15.7 | 15.4 | 15.7 | 15.7 | 15.7 | 15.7 | 15.4 | 15.4 | 15.4 | 15.4 | 15.4 | 15.4 | 15.4 | 15.5 | 15.4 | 15.4 | 16.5 | 16.1 | 16.1 | 16.1 | 16.9 | 16.9 | 18.9 | 18.9 | 18.9 | 18.5 | 18.9 | 18.9 | 16.5 | 16.9 |
| 120 | *T. laeve_*10 | 16.0 | 16.4 | 16.7 | 16.7 | 17.0 | 17.0 | 15.6 | 16.0 | 14.6 | 15.8 | 15.3 | 15.6 | 15.3 | 15.3 | 15.3 | 15.3 | 13.9 | 14.2 | 14.8 | 14.2 | 14.2 | 13.9 | 14.2 | 14.2 | 14.2 | 14.2 | 13.9 | 13.9 | 13.9 | 13.9 | 13.9 | 13.9 | 13.9 | 14.9 | 13.9 | 14.6 | 16.0 | 15.6 | 15.6 | 15.6 | 15.6 | 15.6 | 18.4 | 18.4 | 18.4 | 18.1 | 18.4 | 18.4 | 16.0 | 16.3 |
| 121 | *T. lateriticum_*1 | 17.0 | 17.5 | 17.4 | 17.4 | 17.7 | 17.7 | 18.1 | 17.0 | 15.6 | 17.6 | 17.4 | 17.7 | 17.4 | 17.4 | 17.4 | 17.4 | 17.0 | 16.7 | 17.1 | 17.0 | 17.0 | 16.3 | 16.0 | 16.0 | 16.7 | 16.7 | 16.3 | 16.3 | 16.3 | 16.3 | 16.0 | 16.3 | 16.3 | 16.8 | 16.3 | 17.0 | 12.8 | 12.8 | 12.8 | 12.8 | 17.0 | 17.0 | 13.9 | 13.9 | 13.9 | 13.5 | 13.2 | 13.2 | 14.9 | 14.6 |
| 122 | *T. lateriticum_*2 | 16.0 | 16.4 | 16.3 | 16.3 | 16.7 | 16.7 | 17.0 | 16.0 | 14.6 | 16.5 | 16.3 | 16.7 | 16.3 | 16.3 | 16.3 | 16.3 | 16.0 | 15.6 | 16.2 | 16.0 | 16.0 | 15.3 | 14.9 | 14.9 | 15.6 | 15.6 | 15.3 | 15.3 | 15.3 | 15.3 | 15.0 | 15.3 | 15.3 | 15.7 | 15.3 | 16.0 | 13.2 | 13.2 | 13.2 | 13.2 | 17.0 | 17.0 | 13.9 | 13.9 | 13.9 | 13.5 | 13.9 | 13.9 | 13.5 | 13.2 |
| 123 | *T. lateriticum_*3 | 15.6 | 16.0 | 16.0 | 16.0 | 16.3 | 16.3 | 16.7 | 15.6 | 14.2 | 16.2 | 16.0 | 16.3 | 16.0 | 16.0 | 16.0 | 16.0 | 15.6 | 15.3 | 15.7 | 15.6 | 15.6 | 14.9 | 14.6 | 14.6 | 15.3 | 15.3 | 14.9 | 14.9 | 14.9 | 14.9 | 14.6 | 14.9 | 14.9 | 15.3 | 14.9 | 15.6 | 12.5 | 12.5 | 12.5 | 12.5 | 15.6 | 15.6 | 12.5 | 12.5 | 12.5 | 12.2 | 12.5 | 12.5 | 13.5 | 13.2 |
| 124 | *T. lateriticum_*4 | 15.6 | 16.0 | 16.0 | 16.0 | 16.3 | 16.3 | 16.7 | 15.6 | 14.2 | 16.2 | 16.0 | 16.3 | 16.0 | 16.0 | 16.0 | 16.0 | 15.6 | 15.3 | 16.7 | 15.6 | 15.6 | 14.9 | 14.6 | 14.6 | 15.3 | 15.3 | 14.9 | 14.9 | 14.9 | 14.9 | 14.6 | 14.9 | 14.9 | 15.3 | 14.9 | 15.6 | 12.2 | 12.2 | 12.2 | 12.2 | 16.7 | 16.7 | 13.2 | 13.2 | 13.2 | 12.8 | 13.2 | 13.2 | 14.9 | 14.6 |
| 125 | *T. lateriticum_*5 | 15.6 | 16.0 | 16.0 | 16.0 | 16.3 | 16.3 | 16.7 | 15.6 | 14.2 | 16.2 | 16.0 | 16.3 | 16.0 | 16.0 | 16.0 | 16.0 | 15.6 | 15.3 | 16.2 | 15.6 | 15.6 | 14.9 | 14.6 | 14.6 | 15.3 | 15.3 | 14.9 | 14.9 | 14.9 | 14.9 | 14.6 | 14.9 | 14.9 | 15.3 | 14.9 | 15.6 | 12.5 | 12.2 | 12.2 | 12.2 | 16.3 | 16.3 | 12.8 | 12.8 | 12.8 | 12.5 | 12.8 | 12.8 | 14.2 | 13.9 |
| 126 | *T. lateriticum_*6 | 15.6 | 16.0 | 16.0 | 16.0 | 16.3 | 16.3 | 16.7 | 15.6 | 14.2 | 16.2 | 16.0 | 16.3 | 16.0 | 16.0 | 16.0 | 16.0 | 15.6 | 15.3 | 16.2 | 15.6 | 15.6 | 14.9 | 14.6 | 14.6 | 15.3 | 15.3 | 14.9 | 14.9 | 14.9 | 14.9 | 14.6 | 14.9 | 14.9 | 15.3 | 14.9 | 15.6 | 12.5 | 12.2 | 12.2 | 12.2 | 16.3 | 16.3 | 12.8 | 12.8 | 12.8 | 12.5 | 12.8 | 12.8 | 14.2 | 13.9 |
| 127 | *T. lateriticum_*LT01 | 15.3 | 15.7 | 15.6 | 15.6 | 16.0 | 16.0 | 16.3 | 15.3 | 13.9 | 15.8 | 15.6 | 16.0 | 15.6 | 15.6 | 15.6 | 15.6 | 15.3 | 14.9 | 15.7 | 15.3 | 15.3 | 14.6 | 14.2 | 14.2 | 14.9 | 14.9 | 14.6 | 14.6 | 14.6 | 14.6 | 14.3 | 14.6 | 14.6 | 14.9 | 14.6 | 15.3 | 12.2 | 12.2 | 12.2 | 12.2 | 16.3 | 16.3 | 12.8 | 12.8 | 12.8 | 12.5 | 12.8 | 12.8 | 13.9 | 13.5 |
| 128 | *T. lateriticum_*LT011 | 15.3 | 15.7 | 15.6 | 15.6 | 16.0 | 16.0 | 16.3 | 15.3 | 13.9 | 15.8 | 15.6 | 16.0 | 15.6 | 15.6 | 15.6 | 15.6 | 15.3 | 14.9 | 15.7 | 15.3 | 15.3 | 14.6 | 14.2 | 14.2 | 14.9 | 14.9 | 14.6 | 14.6 | 14.6 | 14.6 | 14.3 | 14.6 | 14.6 | 14.9 | 14.6 | 15.3 | 12.2 | 12.2 | 12.2 | 12.2 | 16.3 | 16.3 | 12.8 | 12.8 | 12.8 | 12.5 | 12.8 | 12.8 | 13.9 | 13.5 |
| 129 | *T. leporosum_*1 | 15.3 | 14.9 | 15.6 | 15.6 | 16.0 | 16.0 | 16.7 | 15.3 | 14.9 | 15.1 | 15.6 | 16.0 | 15.3 | 15.3 | 15.3 | 15.3 | 14.9 | 14.2 | 14.3 | 14.6 | 14.6 | 14.6 | 14.6 | 14.6 | 14.9 | 14.9 | 14.6 | 14.6 | 14.6 | 14.6 | 14.6 | 14.6 | 14.6 | 14.2 | 14.6 | 15.3 | 14.6 | 13.5 | 13.5 | 13.5 | 14.6 | 14.6 | 10.8 | 10.8 | 10.8 | 10.4 | 10.8 | 10.8 | 10.8 | 11.1 |
| 130 | *T. leporosum_*2 | 15.3 | 14.9 | 15.6 | 15.6 | 16.0 | 16.0 | 16.7 | 15.3 | 14.9 | 15.1 | 15.6 | 16.0 | 15.3 | 15.3 | 15.3 | 15.3 | 14.9 | 14.2 | 14.3 | 14.6 | 14.6 | 14.6 | 14.6 | 14.6 | 14.9 | 14.9 | 14.6 | 14.6 | 14.6 | 14.6 | 14.6 | 14.6 | 14.6 | 14.2 | 14.6 | 15.3 | 14.6 | 13.5 | 13.5 | 13.5 | 14.6 | 14.6 | 10.8 | 10.8 | 10.8 | 10.4 | 10.8 | 10.8 | 10.8 | 11.1 |
| 131 | *T. leporosum_*3 | 15.3 | 14.9 | 15.6 | 15.6 | 16.0 | 16.0 | 16.7 | 15.3 | 14.9 | 15.1 | 15.6 | 16.0 | 15.3 | 15.3 | 15.3 | 15.3 | 14.9 | 14.2 | 14.3 | 14.6 | 14.6 | 14.6 | 14.6 | 14.6 | 14.9 | 14.9 | 14.6 | 14.6 | 14.6 | 14.6 | 14.6 | 14.6 | 14.6 | 14.2 | 14.6 | 15.3 | 14.6 | 13.5 | 13.5 | 13.5 | 14.6 | 14.6 | 10.8 | 10.8 | 10.8 | 10.4 | 10.8 | 10.8 | 10.8 | 11.1 |
| 132 | *T. licin_*1 | 11.5 | 11.2 | 10.8 | 10.8 | 11.1 | 13.2 | 12.2 | 12.5 | 11.8 | 12.2 | 12.2 | 12.5 | 11.8 | 11.8 | 11.8 | 11.8 | 11.5 | 10.8 | 11.4 | 11.1 | 11.1 | 10.8 | 10.4 | 10.4 | 10.4 | 10.4 | 10.8 | 10.8 | 10.8 | 10.8 | 10.8 | 10.8 | 10.8 | 11.2 | 10.8 | 11.5 | 15.6 | 14.9 | 14.9 | 14.9 | 9.4 | 9.4 | 14.6 | 14.6 | 14.6 | 14.2 | 14.6 | 14.6 | 10.8 | 10.8 |
| 133 | *T. licin_*2 | 12.2 | 11.9 | 11.8 | 11.8 | 12.2 | 13.5 | 12.8 | 12.8 | 12.8 | 11.9 | 11.8 | 12.2 | 11.5 | 11.5 | 11.5 | 11.5 | 10.8 | 10.8 | 11.0 | 11.1 | 11.1 | 10.1 | 9.7 | 9.7 | 9.7 | 9.7 | 10.1 | 10.1 | 10.1 | 10.1 | 10.1 | 10.1 | 10.1 | 10.4 | 10.1 | 10.8 | 17.0 | 16.3 | 16.3 | 16.3 | 10.1 | 10.1 | 16.3 | 16.3 | 16.3 | 16.0 | 16.3 | 16.3 | 11.1 | 11.1 |
| 134 | *T. licin_*3 | 12.2 | 11.6 | 12.2 | 12.2 | 12.5 | 13.9 | 12.8 | 12.8 | 12.8 | 12.6 | 12.5 | 12.8 | 12.5 | 12.5 | 12.5 | 12.5 | 11.5 | 11.1 | 11.4 | 11.5 | 11.5 | 10.8 | 10.8 | 10.8 | 10.4 | 10.4 | 10.8 | 10.8 | 10.8 | 10.8 | 10.8 | 10.8 | 10.8 | 10.8 | 10.8 | 11.5 | 16.0 | 15.3 | 15.3 | 15.3 | 10.4 | 10.4 | 15.3 | 15.3 | 15.3 | 14.9 | 15.3 | 15.3 | 12.2 | 11.8 |
| 135 | *T. moloch_*1 | 11.8 | 12.3 | 12.5 | 12.5 | 12.8 | 13.5 | 12.5 | 12.2 | 10.4 | 12.6 | 12.2 | 12.5 | 11.8 | 11.8 | 11.8 | 11.8 | 10.8 | 10.1 | 10.5 | 10.4 | 10.4 | 10.8 | 10.1 | 10.1 | 11.1 | 11.1 | 10.8 | 10.8 | 10.8 | 10.8 | 10.8 | 10.8 | 10.8 | 11.2 | 10.8 | 11.5 | 14.6 | 14.2 | 14.2 | 14.2 | 11.1 | 11.1 | 10.1 | 10.1 | 10.1 | 9.7 | 10.1 | 10.1 | 11.8 | 12.2 |
| 136 | *T. moloch_*2 | 11.8 | 12.3 | 12.5 | 12.5 | 12.8 | 13.5 | 12.5 | 12.2 | 10.4 | 12.6 | 12.2 | 12.5 | 11.8 | 11.8 | 11.8 | 11.8 | 10.8 | 10.1 | 10.5 | 10.4 | 10.4 | 10.8 | 10.1 | 10.1 | 11.1 | 11.1 | 10.8 | 10.8 | 10.8 | 10.8 | 10.8 | 10.8 | 10.8 | 11.2 | 10.8 | 11.5 | 14.6 | 14.2 | 14.2 | 14.2 | 11.1 | 11.1 | 10.1 | 10.1 | 10.1 | 9.7 | 10.1 | 10.1 | 11.8 | 12.2 |
| 137 | *T. moloch_*4 | 7.9 | 8.2 | 8.8 | 8.8 | 9.3 | 10.2 | 9.3 | 8.4 | 7.4 | 9.3 | 8.8 | 9.3 | 8.8 | 8.8 | 8.8 | 8.8 | 7.4 | 7.4 | 6.6 | 7.4 | 7.4 | 7.4 | 7.4 | 7.4 | 7.4 | 7.4 | 7.4 | 7.4 | 7.4 | 7.4 | 7.4 | 7.4 | 7.4 | 7.7 | 7.4 | 8.4 | 8.8 | 8.4 | 8.4 | 8.4 | 7.4 | 7.4 | 7.9 | 7.9 | 7.9 | 7.4 | 7.9 | 7.9 | 8.4 | 7.9 |
| 138 | *T. nebulosum_*1 | 17.4 | 18.0 | 19.2 | 19.2 | 19.5 | 19.2 | 18.8 | 18.8 | 18.5 | 19.1 | 18.5 | 18.8 | 18.1 | 18.1 | 18.1 | 18.1 | 16.7 | 16.0 | 17.1 | 16.4 | 16.4 | 16.7 | 16.7 | 16.7 | 17.1 | 17.1 | 16.7 | 16.7 | 16.7 | 16.7 | 16.8 | 16.7 | 16.7 | 18.0 | 16.7 | 17.4 | 12.5 | 10.8 | 10.8 | 10.8 | 13.6 | 13.6 | 13.9 | 13.9 | 13.9 | 13.6 | 13.2 | 13.2 | 14.3 | 15.0 |
| 139 | *T. nebulosum_*2 | 14.9 | 15.3 | 16.7 | 16.7 | 17.0 | 16.7 | 16.3 | 16.3 | 16.0 | 16.5 | 16.0 | 16.3 | 15.6 | 15.6 | 15.6 | 15.6 | 14.2 | 13.5 | 13.8 | 13.9 | 13.9 | 14.2 | 14.2 | 14.2 | 14.6 | 14.6 | 14.2 | 14.2 | 14.2 | 14.2 | 14.3 | 14.2 | 14.2 | 15.3 | 14.2 | 14.9 | 9.7 | 8.0 | 8.0 | 8.0 | 11.5 | 11.5 | 11.5 | 11.5 | 11.5 | 11.1 | 11.5 | 11.5 | 11.5 | 12.2 |
| 140 | *T. nebulosum_*3 | 14.9 | 15.3 | 16.7 | 16.7 | 17.0 | 16.7 | 16.3 | 16.3 | 16.0 | 16.5 | 16.0 | 16.3 | 15.6 | 15.6 | 15.6 | 15.6 | 14.2 | 13.5 | 13.8 | 13.9 | 13.9 | 14.2 | 14.2 | 14.2 | 14.6 | 14.6 | 14.2 | 14.2 | 14.2 | 14.2 | 14.3 | 14.2 | 14.2 | 15.3 | 14.2 | 14.9 | 9.7 | 8.0 | 8.0 | 8.0 | 11.5 | 11.5 | 11.5 | 11.5 | 11.5 | 11.1 | 11.5 | 11.5 | 11.5 | 12.2 |
| 141 | *T. nebulosum_*4 | 14.6 | 15.0 | 16.4 | 16.4 | 16.7 | 16.4 | 16.0 | 16.0 | 15.7 | 16.2 | 15.7 | 16.0 | 15.3 | 15.3 | 15.3 | 15.3 | 13.9 | 13.2 | 13.4 | 13.6 | 13.6 | 13.9 | 13.9 | 13.9 | 14.3 | 14.3 | 13.9 | 13.9 | 13.9 | 13.9 | 14.0 | 13.9 | 13.9 | 15.0 | 13.9 | 14.6 | 9.8 | 8.0 | 8.0 | 8.0 | 11.1 | 11.1 | 11.5 | 11.5 | 11.5 | 11.1 | 11.5 | 11.5 | 11.1 | 11.8 |
| 142 | *T. palliatum_*1 | 14.9 | 15.7 | 16.0 | 16.0 | 16.3 | 16.7 | 16.3 | 15.6 | 14.6 | 15.1 | 14.6 | 14.9 | 14.9 | 14.9 | 14.9 | 14.9 | 14.2 | 14.9 | 15.2 | 15.3 | 15.3 | 13.5 | 13.5 | 13.5 | 13.9 | 13.9 | 13.5 | 13.5 | 13.5 | 13.5 | 13.6 | 13.5 | 13.5 | 14.2 | 13.5 | 14.2 | 14.2 | 14.6 | 14.6 | 14.6 | 15.3 | 15.3 | 8.7 | 8.7 | 8.7 | 8.3 | 8.0 | 8.0 | 14.2 | 13.9 |
| 143 | *T. palliatum_*2 | 15.3 | 16.0 | 16.3 | 16.3 | 16.7 | 17.0 | 16.7 | 16.0 | 14.9 | 15.5 | 14.9 | 15.3 | 15.3 | 15.3 | 15.3 | 15.3 | 14.6 | 15.3 | 15.7 | 15.6 | 15.6 | 13.9 | 13.9 | 13.9 | 14.2 | 14.2 | 13.9 | 13.9 | 13.9 | 13.9 | 13.9 | 13.9 | 13.9 | 14.6 | 13.9 | 14.6 | 14.6 | 14.9 | 14.9 | 14.9 | 14.9 | 14.9 | 9.0 | 9.0 | 9.0 | 8.7 | 8.3 | 8.3 | 14.6 | 14.2 |
| 144 | *T. palliatum_*3 | 14.6 | 15.3 | 15.6 | 15.6 | 16.0 | 16.3 | 16.0 | 15.3 | 14.2 | 14.7 | 14.2 | 14.6 | 14.6 | 14.6 | 14.6 | 14.6 | 13.9 | 14.6 | 14.8 | 14.9 | 14.9 | 13.2 | 13.2 | 13.2 | 13.5 | 13.5 | 13.2 | 13.2 | 13.2 | 13.2 | 13.2 | 13.2 | 13.2 | 13.8 | 13.2 | 13.9 | 13.9 | 14.2 | 14.2 | 14.2 | 14.9 | 14.9 | 8.3 | 8.3 | 8.3 | 8.0 | 8.3 | 8.3 | 13.9 | 13.5 |
| 145 | *T. palliatum_*4 | 14.9 | 15.7 | 16.0 | 16.0 | 16.3 | 16.7 | 16.3 | 15.6 | 14.6 | 15.1 | 14.6 | 14.9 | 14.9 | 14.9 | 14.9 | 14.9 | 14.2 | 14.9 | 15.7 | 15.3 | 15.3 | 13.5 | 13.5 | 13.5 | 13.9 | 13.9 | 13.5 | 13.5 | 13.5 | 13.5 | 13.6 | 13.5 | 13.5 | 14.2 | 13.5 | 14.2 | 14.2 | 14.6 | 14.6 | 14.6 | 15.3 | 15.3 | 9.4 | 9.4 | 9.4 | 9.0 | 8.7 | 8.7 | 14.2 | 13.9 |
| 146 | *T. palliatum_*5 | 14.6 | 15.3 | 15.6 | 15.6 | 16.0 | 16.3 | 16.0 | 15.3 | 14.2 | 14.7 | 14.2 | 14.6 | 14.6 | 14.6 | 14.6 | 14.6 | 13.9 | 14.6 | 14.8 | 14.9 | 14.9 | 13.2 | 13.2 | 13.2 | 13.5 | 13.5 | 13.2 | 13.2 | 13.2 | 13.2 | 13.2 | 13.2 | 13.2 | 13.8 | 13.2 | 13.9 | 13.9 | 14.2 | 14.2 | 14.2 | 14.9 | 14.9 | 8.3 | 8.3 | 8.3 | 8.0 | 8.3 | 8.3 | 13.9 | 13.5 |
| 147 | *T. palliatum_*6 | 14.2 | 14.9 | 15.3 | 15.3 | 15.6 | 16.0 | 15.6 | 14.9 | 13.9 | 14.4 | 13.9 | 14.2 | 14.2 | 14.2 | 14.2 | 14.2 | 13.5 | 14.2 | 14.8 | 14.6 | 14.6 | 12.8 | 12.8 | 12.8 | 13.2 | 13.2 | 12.8 | 12.8 | 12.8 | 12.8 | 12.9 | 12.8 | 12.8 | 13.4 | 12.8 | 13.5 | 13.5 | 13.9 | 13.9 | 13.9 | 15.3 | 15.3 | 8.7 | 8.7 | 8.7 | 8.3 | 8.7 | 8.7 | 13.5 | 13.2 |
| 148 | *T. palliatum_*7 | 15.1 | 15.2 | 16.7 | 16.7 | 17.1 | 16.7 | 17.1 | 16.3 | 15.1 | 15.1 | 15.1 | 15.1 | 15.5 | 15.5 | 15.5 | 15.5 | 14.7 | 15.5 | 16.7 | 15.9 | 15.9 | 13.9 | 13.9 | 13.9 | 14.3 | 14.3 | 13.9 | 13.9 | 13.9 | 13.9 | 13.9 | 13.9 | 13.9 | 13.6 | 13.9 | 13.9 | 14.3 | 14.7 | 14.7 | 14.7 | 17.1 | 17.1 | 9.9 | 9.9 | 9.9 | 9.5 | 9.9 | 9.9 | 14.3 | 13.9 |
| 149 | *T. palliatum_*8 | 14.6 | 14.9 | 15.6 | 15.6 | 16.0 | 16.3 | 16.0 | 15.3 | 14.2 | 14.7 | 14.2 | 14.6 | 14.6 | 14.6 | 14.6 | 14.6 | 13.9 | 14.6 | 15.2 | 14.9 | 14.9 | 13.2 | 13.2 | 13.2 | 13.5 | 13.5 | 13.2 | 13.2 | 13.2 | 13.2 | 13.2 | 13.2 | 13.2 | 13.4 | 13.2 | 13.9 | 13.9 | 14.2 | 14.2 | 14.2 | 15.6 | 15.6 | 9.0 | 9.0 | 9.0 | 8.7 | 9.0 | 9.0 | 13.9 | 13.5 |
| 150 | *T. palliatum_*9 | 14.9 | 15.3 | 16.0 | 16.0 | 16.3 | 16.7 | 16.3 | 15.6 | 14.6 | 15.1 | 14.6 | 14.9 | 14.9 | 14.9 | 14.9 | 14.9 | 14.2 | 14.9 | 15.2 | 15.3 | 15.3 | 13.5 | 13.5 | 13.5 | 13.9 | 13.9 | 13.5 | 13.5 | 13.5 | 13.5 | 13.6 | 13.5 | 13.5 | 13.8 | 13.5 | 14.2 | 14.2 | 14.6 | 14.6 | 14.6 | 15.3 | 15.3 | 8.7 | 8.7 | 8.7 | 8.3 | 8.7 | 8.7 | 14.2 | 13.9 |
| 151 | *T. palliatum_*10 | 14.9 | 15.7 | 15.6 | 15.6 | 16.0 | 16.3 | 16.0 | 15.3 | 14.2 | 14.7 | 14.2 | 14.6 | 14.6 | 14.6 | 14.6 | 14.6 | 14.2 | 14.9 | 15.2 | 15.3 | 15.3 | 13.5 | 13.5 | 13.5 | 13.9 | 13.9 | 13.5 | 13.5 | 13.5 | 13.5 | 13.6 | 13.5 | 13.5 | 14.2 | 13.5 | 14.2 | 14.2 | 14.6 | 14.6 | 14.6 | 15.3 | 15.3 | 8.7 | 8.7 | 8.7 | 8.3 | 8.7 | 8.7 | 14.2 | 13.9 |
| 152 | *T. palliatum_*11 | 14.9 | 15.3 | 16.0 | 16.0 | 16.3 | 16.7 | 16.3 | 15.6 | 14.6 | 15.1 | 14.6 | 14.9 | 14.9 | 14.9 | 14.9 | 14.9 | 14.2 | 14.9 | 15.2 | 15.3 | 15.3 | 13.5 | 13.5 | 13.5 | 13.9 | 13.9 | 13.5 | 13.5 | 13.5 | 13.5 | 13.6 | 13.5 | 13.5 | 13.8 | 13.5 | 14.2 | 14.2 | 14.6 | 14.6 | 14.6 | 15.3 | 15.3 | 8.7 | 8.7 | 8.7 | 8.3 | 8.7 | 8.7 | 14.2 | 13.9 |
| 153 | *T. petilum_*11 | 9.4 | 9.7 | 10.8 | 10.8 | 11.1 | 11.8 | 10.8 | 10.8 | 10.1 | 10.8 | 10.4 | 10.8 | 10.4 | 10.4 | 10.4 | 10.4 | 9.4 | 9.0 | 9.0 | 9.4 | 9.4 | 8.7 | 9.0 | 9.0 | 8.3 | 8.3 | 8.7 | 8.7 | 8.7 | 8.7 | 8.4 | 8.7 | 8.7 | 9.0 | 8.7 | 9.4 | 16.0 | 15.6 | 15.6 | 15.6 | 10.8 | 10.8 | 13.9 | 13.9 | 13.9 | 13.5 | 13.9 | 13.9 | 9.7 | 9.4 |
| 154 | *T. phrynoderma_*1 | 14.2 | 13.9 | 14.2 | 14.2 | 14.6 | 13.4 | 14.2 | 11.8 | 12.2 | 12.6 | 12.6 | 12.6 | 12.6 | 12.6 | 12.6 | 12.6 | 13.0 | 13.0 | 14.2 | 13.4 | 13.4 | 12.2 | 12.6 | 12.6 | 12.6 | 12.6 | 12.2 | 12.2 | 12.2 | 12.2 | 12.3 | 12.2 | 12.2 | 12.3 | 12.2 | 12.2 | 14.6 | 13.8 | 13.8 | 13.8 | 12.6 | 12.6 | 14.6 | 14.6 | 14.6 | 14.2 | 14.6 | 14.6 | 11.4 | 12.2 |
| 155 | *T. phrynoderma_*2 | 13.4 | 13.1 | 13.4 | 13.4 | 13.8 | 12.6 | 13.4 | 11.0 | 11.4 | 11.8 | 11.8 | 11.8 | 11.8 | 11.8 | 11.8 | 11.8 | 12.2 | 12.2 | 13.1 | 12.6 | 12.6 | 11.4 | 11.8 | 11.8 | 11.8 | 11.8 | 11.4 | 11.4 | 11.4 | 11.4 | 11.5 | 11.4 | 11.4 | 11.5 | 11.4 | 11.4 | 14.6 | 13.8 | 13.8 | 13.8 | 12.6 | 12.6 | 13.0 | 13.0 | 13.0 | 12.6 | 13.0 | 13.0 | 11.4 | 12.2 |
| 156 | *T. pyaukkya_*1 | 11.1 | 11.2 | 11.5 | 11.5 | 11.8 | 12.5 | 11.8 | 11.8 | 10.1 | 11.2 | 10.8 | 11.1 | 10.4 | 10.4 | 10.4 | 10.4 | 10.4 | 10.4 | 11.0 | 10.8 | 10.8 | 9.7 | 9.0 | 9.0 | 9.4 | 9.4 | 9.7 | 9.7 | 9.7 | 9.7 | 9.8 | 9.7 | 9.7 | 9.7 | 9.7 | 10.4 | 12.8 | 12.5 | 12.5 | 12.5 | 8.0 | 8.0 | 13.9 | 13.9 | 13.9 | 13.5 | 13.9 | 13.9 | 6.3 | 5.6 |
| 157 | *T. pyaukkya_*2 | 11.5 | 11.6 | 12.2 | 12.2 | 12.5 | 13.2 | 12.2 | 12.5 | 10.8 | 11.9 | 11.5 | 11.8 | 11.1 | 11.1 | 11.1 | 11.1 | 10.8 | 10.8 | 11.0 | 11.1 | 11.1 | 10.1 | 9.4 | 9.4 | 9.7 | 9.7 | 10.1 | 10.1 | 10.1 | 10.1 | 10.1 | 10.1 | 10.1 | 10.1 | 10.1 | 10.8 | 12.8 | 12.5 | 12.5 | 12.5 | 8.7 | 8.7 | 13.5 | 13.5 | 13.5 | 13.2 | 13.5 | 13.5 | 6.3 | 5.6 |
| 158 | *T. pyaukkya_*3 | 10.8 | 10.8 | 11.5 | 11.5 | 11.8 | 12.5 | 11.5 | 11.8 | 10.1 | 11.2 | 10.8 | 11.1 | 10.4 | 10.4 | 10.4 | 10.4 | 10.1 | 10.1 | 10.0 | 10.4 | 10.4 | 9.4 | 8.7 | 8.7 | 9.0 | 9.0 | 9.4 | 9.4 | 9.4 | 9.4 | 9.4 | 9.4 | 9.4 | 9.3 | 9.4 | 10.1 | 12.8 | 13.2 | 13.2 | 13.2 | 8.0 | 8.0 | 13.5 | 13.5 | 13.5 | 13.2 | 13.5 | 13.5 | 6.3 | 5.6 |
| 159 | *T. pyaukkya_*4 | 9.7 | 9.7 | 11.1 | 11.1 | 11.5 | 12.2 | 11.1 | 11.5 | 9.7 | 11.5 | 11.1 | 11.5 | 10.8 | 10.8 | 10.8 | 10.8 | 10.4 | 9.7 | 10.0 | 10.1 | 10.1 | 9.7 | 9.7 | 9.7 | 9.4 | 9.4 | 9.7 | 9.7 | 9.7 | 9.7 | 9.8 | 9.7 | 9.7 | 9.7 | 9.7 | 10.4 | 13.2 | 11.8 | 11.8 | 11.8 | 6.3 | 6.3 | 14.6 | 14.6 | 14.6 | 14.2 | 14.6 | 14.6 | 3.5 | 3.1 |
| 160 | *T. pyaukkya_*5 | 9.7 | 9.7 | 11.1 | 11.1 | 11.5 | 12.2 | 11.1 | 11.5 | 9.7 | 11.5 | 11.1 | 11.5 | 10.8 | 10.8 | 10.8 | 10.8 | 10.4 | 9.7 | 10.0 | 10.1 | 10.1 | 9.7 | 9.7 | 9.7 | 9.4 | 9.4 | 9.7 | 9.7 | 9.7 | 9.7 | 9.8 | 9.7 | 9.7 | 9.7 | 9.7 | 10.4 | 13.2 | 11.8 | 11.8 | 11.8 | 6.3 | 6.3 | 14.6 | 14.6 | 14.6 | 14.2 | 14.6 | 14.6 | 3.5 | 3.1 |
| 161 | *T. rhododiscus_*1 | 13.5 | 14.2 | 14.9 | 14.9 | 15.3 | 16.0 | 14.9 | 14.9 | 13.9 | 15.5 | 14.9 | 15.3 | 14.9 | 14.9 | 14.9 | 14.9 | 13.2 | 12.8 | 13.3 | 13.2 | 13.2 | 13.2 | 12.8 | 12.8 | 12.8 | 12.8 | 13.2 | 13.2 | 13.2 | 13.2 | 13.2 | 13.2 | 13.2 | 13.8 | 13.2 | 13.9 | 15.3 | 15.6 | 15.6 | 15.6 | 13.2 | 13.2 | 9.7 | 9.7 | 9.7 | 9.4 | 9.7 | 9.7 | 12.8 | 12.2 |
| 162 | *T. rhododiscus*10 | 14.2 | 14.9 | 15.6 | 15.6 | 16.0 | 16.7 | 15.6 | 15.6 | 14.6 | 16.2 | 15.6 | 16.0 | 15.6 | 15.6 | 15.6 | 15.6 | 13.9 | 13.5 | 13.8 | 13.9 | 13.9 | 13.9 | 13.5 | 13.5 | 13.5 | 13.5 | 13.9 | 13.9 | 13.9 | 13.9 | 13.9 | 13.9 | 13.9 | 14.6 | 13.9 | 14.6 | 15.6 | 15.6 | 15.6 | 15.6 | 13.5 | 13.5 | 10.4 | 10.4 | 10.4 | 10.1 | 10.4 | 10.4 | 12.5 | 11.8 |
| 163 | *T. rhododiscus_*2 | 13.5 | 14.2 | 14.9 | 14.9 | 15.3 | 16.0 | 14.9 | 14.9 | 13.9 | 15.5 | 14.9 | 15.3 | 14.9 | 14.9 | 14.9 | 14.9 | 13.2 | 12.8 | 13.3 | 13.2 | 13.2 | 13.2 | 12.8 | 12.8 | 12.8 | 12.8 | 13.2 | 13.2 | 13.2 | 13.2 | 13.2 | 13.2 | 13.2 | 13.8 | 13.2 | 13.9 | 15.3 | 15.6 | 15.6 | 15.6 | 13.2 | 13.2 | 9.7 | 9.7 | 9.7 | 9.4 | 9.7 | 9.7 | 12.8 | 12.2 |
| 164 | *T. rhododiscu_s*3 | 13.5 | 14.2 | 14.9 | 14.9 | 15.3 | 16.0 | 14.9 | 14.9 | 13.9 | 15.5 | 14.9 | 15.3 | 14.9 | 14.9 | 14.9 | 14.9 | 13.2 | 12.8 | 13.3 | 13.2 | 13.2 | 13.2 | 12.8 | 12.8 | 12.8 | 12.8 | 13.2 | 13.2 | 13.2 | 13.2 | 13.2 | 13.2 | 13.2 | 13.8 | 13.2 | 13.9 | 15.3 | 15.6 | 15.6 | 15.6 | 13.2 | 13.2 | 9.7 | 9.7 | 9.7 | 9.4 | 9.7 | 9.7 | 12.8 | 12.2 |
| 165 | *T. rhododiscus_*4 | 13.5 | 14.2 | 14.9 | 14.9 | 15.3 | 16.0 | 14.9 | 14.9 | 13.9 | 15.5 | 14.9 | 15.3 | 14.9 | 14.9 | 14.9 | 14.9 | 13.2 | 12.8 | 13.3 | 13.2 | 13.2 | 13.2 | 12.8 | 12.8 | 12.8 | 12.8 | 13.2 | 13.2 | 13.2 | 13.2 | 13.2 | 13.2 | 13.2 | 13.8 | 13.2 | 13.9 | 15.3 | 15.6 | 15.6 | 15.6 | 13.2 | 13.2 | 9.7 | 9.7 | 9.7 | 9.4 | 9.7 | 9.7 | 12.8 | 12.2 |
| 166 | *T. rhododiscus_*5 | 13.5 | 14.2 | 14.9 | 14.9 | 15.3 | 16.0 | 14.9 | 14.9 | 13.9 | 15.5 | 14.9 | 15.3 | 14.9 | 14.9 | 14.9 | 14.9 | 13.2 | 12.8 | 13.3 | 13.2 | 13.2 | 13.2 | 12.8 | 12.8 | 12.8 | 12.8 | 13.2 | 13.2 | 13.2 | 13.2 | 13.2 | 13.2 | 13.2 | 13.8 | 13.2 | 13.9 | 15.3 | 15.6 | 15.6 | 15.6 | 13.2 | 13.2 | 9.7 | 9.7 | 9.7 | 9.4 | 9.7 | 9.7 | 12.8 | 12.2 |
| 167 | *T. rhododiscus_*6 | 13.5 | 14.2 | 14.9 | 14.9 | 15.3 | 16.0 | 14.9 | 14.9 | 13.9 | 15.5 | 14.9 | 15.3 | 14.9 | 14.9 | 14.9 | 14.9 | 13.2 | 12.8 | 13.3 | 13.2 | 13.2 | 13.2 | 12.8 | 12.8 | 12.8 | 12.8 | 13.2 | 13.2 | 13.2 | 13.2 | 13.2 | 13.2 | 13.2 | 13.8 | 13.2 | 13.9 | 15.3 | 15.6 | 15.6 | 15.6 | 13.2 | 13.2 | 9.7 | 9.7 | 9.7 | 9.4 | 9.7 | 9.7 | 12.8 | 12.2 |
| 168 | *T. rhododiscus_*7 | 13.5 | 14.2 | 14.9 | 14.9 | 15.3 | 16.0 | 14.9 | 14.9 | 13.9 | 15.5 | 14.9 | 15.3 | 14.9 | 14.9 | 14.9 | 14.9 | 13.2 | 12.8 | 13.3 | 13.2 | 13.2 | 13.2 | 12.8 | 12.8 | 12.8 | 12.8 | 13.2 | 13.2 | 13.2 | 13.2 | 13.2 | 13.2 | 13.2 | 13.8 | 13.2 | 13.9 | 15.3 | 15.6 | 15.6 | 15.6 | 13.2 | 13.2 | 9.7 | 9.7 | 9.7 | 9.4 | 9.7 | 9.7 | 12.8 | 12.2 |
| 169 | *T. rhododiscus_*8 | 13.5 | 14.2 | 14.9 | 14.9 | 15.3 | 16.0 | 14.9 | 14.9 | 13.9 | 15.5 | 14.9 | 15.3 | 14.9 | 14.9 | 14.9 | 14.9 | 13.2 | 12.8 | 13.3 | 13.2 | 13.2 | 13.2 | 12.8 | 12.8 | 12.8 | 12.8 | 13.2 | 13.2 | 13.2 | 13.2 | 13.2 | 13.2 | 13.2 | 13.8 | 13.2 | 13.9 | 15.3 | 15.6 | 15.6 | 15.6 | 13.2 | 13.2 | 9.7 | 9.7 | 9.7 | 9.4 | 9.7 | 9.7 | 12.8 | 12.2 |
| 170 | *T. rhododiscus_*9 | 13.9 | 14.6 | 15.3 | 15.3 | 15.6 | 16.3 | 15.3 | 15.3 | 14.2 | 15.8 | 15.3 | 15.6 | 15.3 | 15.3 | 15.3 | 15.3 | 13.5 | 13.2 | 13.8 | 13.5 | 13.5 | 13.5 | 13.2 | 13.2 | 13.2 | 13.2 | 13.5 | 13.5 | 13.5 | 13.5 | 13.6 | 13.5 | 13.5 | 14.2 | 13.5 | 14.2 | 15.6 | 15.3 | 15.3 | 15.3 | 13.2 | 13.2 | 10.1 | 10.1 | 10.1 | 9.7 | 10.1 | 10.1 | 12.2 | 11.5 |
| 171 | *T. ryabovi_*1 | 14.9 | 15.3 | 16.0 | 16.0 | 16.3 | 17.0 | 15.6 | 15.3 | 13.5 | 15.1 | 14.6 | 14.9 | 14.6 | 14.6 | 14.6 | 14.6 | 13.2 | 13.5 | 13.8 | 13.5 | 13.5 | 13.2 | 12.8 | 12.8 | 13.5 | 13.5 | 13.2 | 13.2 | 13.2 | 13.2 | 13.2 | 13.2 | 13.2 | 13.4 | 13.2 | 13.9 | 14.6 | 13.9 | 13.9 | 13.9 | 12.8 | 12.8 | 11.5 | 11.5 | 11.5 | 11.1 | 11.5 | 11.5 | 12.8 | 12.8 |
| 172 | *T. ryabovi_*2 | 16.7 | 16.0 | 17.7 | 17.7 | 18.1 | 18.8 | 17.4 | 17.0 | 15.3 | 16.2 | 16.3 | 16.7 | 16.3 | 16.3 | 16.3 | 16.3 | 14.9 | 15.3 | 14.8 | 15.3 | 15.3 | 14.9 | 14.6 | 14.6 | 15.3 | 15.3 | 14.9 | 14.9 | 14.9 | 14.9 | 15.0 | 14.9 | 14.9 | 14.2 | 14.9 | 15.6 | 17.7 | 17.0 | 17.0 | 17.0 | 15.3 | 15.3 | 14.6 | 14.6 | 14.6 | 14.2 | 14.6 | 14.6 | 15.3 | 15.3 |
| 173 | *T. ryabovi_*3 | 16.7 | 16.0 | 17.7 | 17.7 | 18.1 | 18.8 | 17.4 | 17.0 | 15.3 | 16.2 | 16.3 | 16.7 | 16.3 | 16.3 | 16.3 | 16.3 | 14.9 | 15.3 | 14.8 | 15.3 | 15.3 | 14.9 | 14.6 | 14.6 | 15.3 | 15.3 | 14.9 | 14.9 | 14.9 | 14.9 | 15.0 | 14.9 | 14.9 | 14.2 | 14.9 | 15.6 | 17.7 | 17.0 | 17.0 | 17.0 | 15.3 | 15.3 | 14.6 | 14.6 | 14.6 | 14.2 | 14.6 | 14.6 | 15.3 | 15.3 |
| 174 | *T. stellatum_*1 | 18.8 | 19.8 | 19.1 | 19.1 | 19.4 | 19.8 | 20.1 | 20.1 | 18.8 | 19.1 | 18.4 | 18.8 | 18.4 | 18.4 | 18.4 | 18.4 | 17.0 | 17.4 | 18.1 | 17.7 | 17.7 | 17.7 | 18.1 | 18.1 | 17.4 | 17.4 | 17.7 | 17.7 | 17.7 | 17.7 | 17.8 | 17.7 | 17.7 | 18.7 | 17.7 | 18.4 | 17.0 | 18.4 | 18.4 | 18.4 | 18.1 | 18.1 | 17.4 | 17.4 | 17.4 | 17.0 | 17.4 | 17.4 | 19.4 | 19.1 |
| 175 | *T. stellatum_*2 | 18.8 | 19.8 | 19.1 | 19.1 | 19.4 | 19.8 | 20.1 | 20.1 | 18.8 | 19.1 | 18.4 | 18.8 | 18.4 | 18.4 | 18.4 | 18.4 | 17.0 | 17.4 | 18.1 | 17.7 | 17.7 | 17.7 | 18.1 | 18.1 | 17.4 | 17.4 | 17.7 | 17.7 | 17.7 | 17.7 | 17.8 | 17.7 | 17.7 | 18.7 | 17.7 | 18.4 | 17.0 | 18.4 | 18.4 | 18.4 | 18.1 | 18.1 | 17.4 | 17.4 | 17.4 | 17.0 | 17.4 | 17.4 | 19.4 | 19.1 |
| 176 | *T. truongsonense_*1 | 14.2 | 14.6 | 14.9 | 14.9 | 15.3 | 15.3 | 14.2 | 14.9 | 13.9 | 15.1 | 14.6 | 14.9 | 14.2 | 14.2 | 14.2 | 14.2 | 14.2 | 13.5 | 13.8 | 13.9 | 13.9 | 13.5 | 13.5 | 13.5 | 13.9 | 13.9 | 13.5 | 13.5 | 13.5 | 13.5 | 13.6 | 13.5 | 13.5 | 14.6 | 13.5 | 14.2 | 11.5 | 11.5 | 11.5 | 11.5 | 12.8 | 12.8 | 12.8 | 12.8 | 12.8 | 12.5 | 12.8 | 12.8 | 13.2 | 13.9 |
| 177 | *T. truongsonense_*2 | 14.9 | 15.7 | 14.9 | 14.9 | 15.3 | 16.0 | 14.9 | 15.6 | 13.9 | 15.8 | 15.3 | 15.6 | 14.9 | 14.9 | 14.9 | 14.9 | 15.3 | 14.6 | 13.8 | 14.9 | 14.9 | 14.9 | 14.9 | 14.9 | 15.3 | 15.3 | 14.9 | 14.9 | 14.9 | 14.9 | 15.0 | 14.9 | 14.9 | 15.7 | 14.9 | 15.6 | 12.8 | 12.8 | 12.8 | 12.8 | 13.5 | 13.5 | 13.5 | 13.5 | 13.5 | 13.2 | 13.5 | 13.5 | 13.9 | 14.6 |
| 178 | *T. truongsonense_*3 | 14.9 | 15.7 | 14.9 | 14.9 | 15.3 | 16.0 | 14.9 | 15.6 | 13.9 | 15.8 | 15.3 | 15.6 | 14.9 | 14.9 | 14.9 | 14.9 | 15.3 | 14.6 | 13.8 | 14.9 | 14.9 | 14.9 | 14.9 | 14.9 | 15.3 | 15.3 | 14.9 | 14.9 | 14.9 | 14.9 | 15.0 | 14.9 | 14.9 | 15.7 | 14.9 | 15.6 | 12.8 | 12.8 | 12.8 | 12.8 | 13.5 | 13.5 | 13.5 | 13.5 | 13.5 | 13.2 | 13.5 | 13.5 | 13.9 | 14.6 |
| 179 | *T. truongsonense_*4 | 15.3 | 15.7 | 15.6 | 15.6 | 16.0 | 15.6 | 15.3 | 14.9 | 14.2 | 14.0 | 13.5 | 13.9 | 13.5 | 13.5 | 13.5 | 13.5 | 13.5 | 13.9 | 13.8 | 14.2 | 14.2 | 14.2 | 14.6 | 14.6 | 14.6 | 14.6 | 14.2 | 14.2 | 14.2 | 14.2 | 14.3 | 14.2 | 14.2 | 15.3 | 14.2 | 14.9 | 11.1 | 11.1 | 11.1 | 11.1 | 13.9 | 13.9 | 11.8 | 11.8 | 11.8 | 11.5 | 11.8 | 11.8 | 14.2 | 14.6 |
| 180 | *T. truongsonense_*5 | 16.2 | 16.0 | 16.6 | 16.6 | 17.0 | 15.9 | 15.9 | 17.0 | 16.2 | 15.9 | 15.9 | 15.9 | 15.5 | 15.5 | 15.5 | 15.5 | 15.5 | 14.8 | 15.0 | 15.1 | 15.1 | 16.2 | 16.2 | 16.2 | 16.6 | 16.6 | 16.2 | 16.2 | 16.2 | 16.2 | 16.3 | 16.2 | 16.2 | 16.4 | 16.2 | 16.2 | 12.2 | 12.9 | 12.9 | 12.9 | 15.9 | 15.9 | 14.0 | 14.0 | 14.0 | 13.7 | 14.0 | 14.0 | 16.2 | 17.0 |
| 181 | *T. truongsonense_*6 | 14.6 | 14.9 | 15.6 | 15.6 | 16.0 | 15.6 | 14.6 | 15.6 | 14.6 | 14.4 | 13.9 | 14.2 | 13.9 | 13.9 | 13.9 | 13.9 | 12.8 | 13.2 | 13.3 | 13.5 | 13.5 | 13.5 | 13.9 | 13.9 | 13.9 | 13.9 | 13.5 | 13.5 | 13.5 | 13.5 | 13.6 | 13.5 | 13.5 | 14.6 | 13.5 | 14.2 | 11.1 | 11.8 | 11.8 | 11.8 | 13.2 | 13.2 | 12.5 | 12.5 | 12.5 | 12.2 | 12.5 | 12.5 | 14.2 | 14.6 |
| 182 | *T. truongsonense_*7 | 14.6 | 14.9 | 15.6 | 15.6 | 16.0 | 15.6 | 14.6 | 15.6 | 14.6 | 14.4 | 13.9 | 14.2 | 13.9 | 13.9 | 13.9 | 13.9 | 12.8 | 13.2 | 13.3 | 13.5 | 13.5 | 13.5 | 13.9 | 13.9 | 13.9 | 13.9 | 13.5 | 13.5 | 13.5 | 13.5 | 13.6 | 13.5 | 13.5 | 14.6 | 13.5 | 14.2 | 11.1 | 11.8 | 11.8 | 11.8 | 13.2 | 13.2 | 12.5 | 12.5 | 12.5 | 12.2 | 12.5 | 12.5 | 14.2 | 14.6 |
| 183 | *T. vietnamense_*1 | 16.7 | 17.2 | 16.0 | 16.0 | 16.3 | 17.4 | 17.4 | 17.4 | 15.6 | 16.5 | 16.3 | 16.7 | 16.3 | 16.3 | 16.3 | 16.3 | 15.6 | 16.0 | 15.2 | 16.3 | 16.3 | 15.6 | 16.0 | 16.0 | 15.3 | 15.3 | 15.6 | 15.6 | 15.6 | 15.6 | 15.7 | 15.6 | 15.6 | 16.0 | 15.6 | 16.3 | 15.3 | 16.3 | 16.3 | 16.3 | 16.3 | 16.3 | 15.3 | 15.3 | 15.3 | 14.9 | 15.3 | 15.3 | 17.7 | 17.4 |
| 184 | *T. vietnamense_*2 | 17.0 | 17.5 | 16.3 | 16.3 | 16.7 | 17.7 | 17.7 | 17.7 | 16.0 | 16.9 | 16.7 | 17.0 | 16.7 | 16.7 | 16.7 | 16.7 | 16.0 | 16.3 | 15.7 | 16.7 | 16.7 | 16.0 | 16.3 | 16.3 | 15.6 | 15.6 | 16.0 | 16.0 | 16.0 | 16.0 | 16.0 | 16.0 | 16.0 | 16.4 | 16.0 | 16.7 | 15.3 | 16.3 | 16.3 | 16.3 | 16.7 | 16.7 | 15.6 | 15.6 | 15.6 | 15.3 | 15.6 | 15.6 | 18.1 | 17.7 |
| 185 | *T. vietnamense_*3 | 18.8 | 19.4 | 18.1 | 18.1 | 18.4 | 19.4 | 19.4 | 19.4 | 17.7 | 18.7 | 18.4 | 18.8 | 18.4 | 18.4 | 18.4 | 18.4 | 17.7 | 18.1 | 17.1 | 18.4 | 18.4 | 17.7 | 17.4 | 17.4 | 17.4 | 17.4 | 17.7 | 17.7 | 17.7 | 17.7 | 17.8 | 17.7 | 17.7 | 18.3 | 17.7 | 18.4 | 16.3 | 17.4 | 17.4 | 17.4 | 18.4 | 18.4 | 15.6 | 15.6 | 15.6 | 15.3 | 15.6 | 15.6 | 19.4 | 19.1 |
| 186 | *T. vietnamense_*4 | 18.8 | 19.5 | 18.1 | 18.1 | 18.5 | 19.5 | 19.5 | 19.5 | 17.8 | 18.8 | 18.5 | 18.8 | 18.5 | 18.5 | 18.5 | 18.5 | 17.8 | 18.1 | 17.2 | 18.5 | 18.5 | 17.8 | 17.4 | 17.4 | 17.4 | 17.4 | 17.8 | 17.8 | 17.8 | 17.8 | 17.8 | 17.8 | 17.8 | 18.4 | 17.8 | 18.5 | 16.0 | 17.1 | 17.1 | 17.1 | 18.5 | 18.5 | 15.7 | 15.7 | 15.7 | 15.3 | 15.7 | 15.7 | 19.5 | 19.2 |
| 187 | *T. vietnamense_*5 | 17.4 | 17.9 | 16.7 | 16.7 | 17.0 | 18.1 | 18.1 | 18.1 | 16.3 | 17.3 | 17.0 | 17.4 | 17.0 | 17.0 | 17.0 | 17.0 | 16.3 | 16.7 | 15.7 | 17.0 | 17.0 | 16.3 | 16.7 | 16.7 | 16.0 | 16.0 | 16.3 | 16.3 | 16.3 | 16.3 | 16.4 | 16.3 | 16.3 | 16.8 | 16.3 | 17.0 | 14.9 | 16.0 | 16.0 | 16.0 | 17.0 | 17.0 | 15.3 | 15.3 | 15.3 | 14.9 | 15.3 | 15.3 | 18.1 | 17.7 |
| 188 | *T. vietnamense_*6 | 17.4 | 17.9 | 16.7 | 16.7 | 17.0 | 18.1 | 18.1 | 18.1 | 16.3 | 17.3 | 17.0 | 17.4 | 17.0 | 17.0 | 17.0 | 17.0 | 16.3 | 16.7 | 15.7 | 17.0 | 17.0 | 16.3 | 16.7 | 16.7 | 16.0 | 16.0 | 16.3 | 16.3 | 16.3 | 16.3 | 16.4 | 16.3 | 16.3 | 16.8 | 16.3 | 17.0 | 14.9 | 16.0 | 16.0 | 16.0 | 17.0 | 17.0 | 15.3 | 15.3 | 15.3 | 14.9 | 15.3 | 15.3 | 18.1 | 17.7 |
| 189 | *T. vietnamense_*7 | 17.4 | 17.9 | 16.7 | 16.7 | 17.0 | 18.1 | 18.1 | 18.1 | 16.3 | 17.3 | 17.0 | 17.4 | 17.0 | 17.0 | 17.0 | 17.0 | 16.3 | 16.7 | 15.7 | 17.0 | 17.0 | 16.3 | 16.7 | 16.7 | 16.0 | 16.0 | 16.3 | 16.3 | 16.3 | 16.3 | 16.4 | 16.3 | 16.3 | 16.8 | 16.3 | 17.0 | 14.9 | 16.0 | 16.0 | 16.0 | 17.0 | 17.0 | 15.3 | 15.3 | 15.3 | 14.9 | 15.3 | 15.3 | 18.1 | 17.7 |
| 190 | *T. vietnamense_*8 | 17.7 | 18.3 | 16.3 | 16.3 | 16.7 | 17.7 | 18.4 | 17.7 | 16.0 | 16.9 | 16.7 | 17.0 | 16.7 | 16.7 | 16.7 | 16.7 | 16.7 | 17.0 | 16.2 | 17.4 | 17.4 | 16.7 | 17.0 | 17.0 | 16.3 | 16.3 | 16.7 | 16.7 | 16.7 | 16.7 | 16.7 | 16.7 | 16.7 | 17.2 | 16.7 | 17.4 | 14.6 | 15.6 | 15.6 | 15.6 | 16.7 | 16.7 | 15.6 | 15.6 | 15.6 | 15.3 | 15.6 | 15.6 | 17.7 | 17.4 |
| 191 | *T. vietnamense_*9 | 17.7 | 18.3 | 16.3 | 16.3 | 16.7 | 17.7 | 18.4 | 17.7 | 16.0 | 16.9 | 16.7 | 17.0 | 16.7 | 16.7 | 16.7 | 16.7 | 16.7 | 17.0 | 16.2 | 17.4 | 17.4 | 16.7 | 17.0 | 17.0 | 16.3 | 16.3 | 16.7 | 16.7 | 16.7 | 16.7 | 16.7 | 16.7 | 16.7 | 17.2 | 16.7 | 17.4 | 14.6 | 15.6 | 15.6 | 15.6 | 16.7 | 16.7 | 15.6 | 15.6 | 15.6 | 15.3 | 15.6 | 15.6 | 17.7 | 17.4 |
| 192 | *T. vietnamense_*10 | 18.1 | 17.9 | 17.4 | 17.4 | 17.7 | 18.8 | 18.8 | 18.8 | 17.0 | 18.0 | 17.7 | 18.1 | 17.7 | 17.7 | 17.7 | 17.7 | 17.0 | 17.4 | 16.7 | 17.7 | 17.7 | 17.0 | 17.4 | 17.4 | 16.7 | 16.7 | 17.0 | 17.0 | 17.0 | 17.0 | 17.1 | 17.0 | 17.0 | 16.8 | 17.0 | 17.7 | 14.9 | 16.0 | 16.0 | 16.0 | 17.7 | 17.7 | 16.0 | 16.0 | 16.0 | 15.6 | 16.0 | 16.0 | 18.4 | 18.1 |
| 193 | *T. vietnamense_*11 | 17.4 | 17.9 | 16.7 | 16.7 | 17.0 | 18.1 | 18.1 | 18.1 | 16.3 | 17.3 | 17.0 | 17.4 | 17.0 | 17.0 | 17.0 | 17.0 | 16.3 | 16.7 | 15.7 | 17.0 | 17.0 | 16.3 | 16.7 | 16.7 | 16.0 | 16.0 | 16.3 | 16.3 | 16.3 | 16.3 | 16.4 | 16.3 | 16.3 | 16.8 | 16.3 | 17.0 | 14.9 | 16.0 | 16.0 | 16.0 | 17.0 | 17.0 | 15.3 | 15.3 | 15.3 | 14.9 | 15.3 | 15.3 | 18.1 | 17.7 |
| 194 | *T. vietnamense_*12 | 17.4 | 17.9 | 16.7 | 16.7 | 17.0 | 18.1 | 18.1 | 18.1 | 16.3 | 17.3 | 17.0 | 17.4 | 17.0 | 17.0 | 17.0 | 17.0 | 16.3 | 16.7 | 15.7 | 17.0 | 17.0 | 16.3 | 16.7 | 16.7 | 16.0 | 16.0 | 16.3 | 16.3 | 16.3 | 16.3 | 16.4 | 16.3 | 16.3 | 16.8 | 16.3 | 17.0 | 14.9 | 16.0 | 16.0 | 16.0 | 17.0 | 17.0 | 15.3 | 15.3 | 15.3 | 14.9 | 15.3 | 15.3 | 18.1 | 17.7 |
| 195 | *T. vietnamense_*13 | 17.4 | 17.9 | 16.7 | 16.7 | 17.0 | 18.1 | 18.1 | 18.1 | 16.3 | 17.3 | 17.0 | 17.4 | 17.0 | 17.0 | 17.0 | 17.0 | 16.3 | 16.7 | 15.7 | 17.0 | 17.0 | 16.3 | 16.7 | 16.7 | 16.0 | 16.0 | 16.3 | 16.3 | 16.3 | 16.3 | 16.4 | 16.3 | 16.3 | 16.8 | 16.3 | 17.0 | 14.9 | 16.0 | 16.0 | 16.0 | 17.0 | 17.0 | 15.3 | 15.3 | 15.3 | 14.9 | 15.3 | 15.3 | 18.1 | 17.7 |

Next page

Continue to

| ID | Species | 51 | 52 | 53 | 54 | 55 | 56 | 57 | 58 | 59 | 60 | 61 | 62 | 63 | 64 | 65 | 66 | 67 | 68 | 69 | 70 | 71 | 72 | 73 | 74 | 75 | 76 | 77 | 78 | 79 | 80 | 81 | 82 | 83 | 84 | 85 | 86 | 87 | 88 | 89 | 90 | 91 | 92 | 93 | 94 | 95 | 96 | 97 | 98 | 99 | 100 |
| --- | --- | --- | --- | --- | --- | --- | --- | --- | --- | --- | --- | --- | --- | --- | --- | --- | --- | --- | --- | --- | --- | --- | --- | --- | --- | --- | --- | --- | --- | --- | --- | --- | --- | --- | --- | --- | --- | --- | --- | --- | --- | --- | --- | --- | --- | --- | --- | --- | --- | --- | --- |
| 1 | *T. albopunctatum_*1 |  |  |  |  |  |  |  |  |  |  |  |  |  |  |  |  |  |  |  |  |  |  |  |  |  |  |  |  |  |  |  |  |  |  |  |  |  |  |  |  |  |  |  |  |  |  |  |  |  |  |
| 2 | *T. albopunctatum_*2 |  |  |  |  |  |  |  |  |  |  |  |  |  |  |  |  |  |  |  |  |  |  |  |  |  |  |  |  |  |  |  |  |  |  |  |  |  |  |  |  |  |  |  |  |  |  |  |  |  |  |
| 3 | *T. albopunctatum_*3 |  |  |  |  |  |  |  |  |  |  |  |  |  |  |  |  |  |  |  |  |  |  |  |  |  |  |  |  |  |  |  |  |  |  |  |  |  |  |  |  |  |  |  |  |  |  |  |  |  |  |
| 4 | *T. albopunctatum_*4 |  |  |  |  |  |  |  |  |  |  |  |  |  |  |  |  |  |  |  |  |  |  |  |  |  |  |  |  |  |  |  |  |  |  |  |  |  |  |  |  |  |  |  |  |  |  |  |  |  |  |
| 5 | *T. albopunctatum_*5 |  |  |  |  |  |  |  |  |  |  |  |  |  |  |  |  |  |  |  |  |  |  |  |  |  |  |  |  |  |  |  |  |  |  |  |  |  |  |  |  |  |  |  |  |  |  |  |  |  |  |
| 6 | *T. albopunctatum_*6 |  |  |  |  |  |  |  |  |  |  |  |  |  |  |  |  |  |  |  |  |  |  |  |  |  |  |  |  |  |  |  |  |  |  |  |  |  |  |  |  |  |  |  |  |  |  |  |  |  |  |
| 7 | *T. albopunctatum_*7 |  |  |  |  |  |  |  |  |  |  |  |  |  |  |  |  |  |  |  |  |  |  |  |  |  |  |  |  |  |  |  |  |  |  |  |  |  |  |  |  |  |  |  |  |  |  |  |  |  |  |
| 8 | *T. albopunctatum_*8 |  |  |  |  |  |  |  |  |  |  |  |  |  |  |  |  |  |  |  |  |  |  |  |  |  |  |  |  |  |  |  |  |  |  |  |  |  |  |  |  |  |  |  |  |  |  |  |  |  |  |
| 9 | *T. albopunctatum_*9 |  |  |  |  |  |  |  |  |  |  |  |  |  |  |  |  |  |  |  |  |  |  |  |  |  |  |  |  |  |  |  |  |  |  |  |  |  |  |  |  |  |  |  |  |  |  |  |  |  |  |
| 10 | *T. albopunctatum_*10 |  |  |  |  |  |  |  |  |  |  |  |  |  |  |  |  |  |  |  |  |  |  |  |  |  |  |  |  |  |  |  |  |  |  |  |  |  |  |  |  |  |  |  |  |  |  |  |  |  |  |
| 11 | *T. albopunctatum_*11 |  |  |  |  |  |  |  |  |  |  |  |  |  |  |  |  |  |  |  |  |  |  |  |  |  |  |  |  |  |  |  |  |  |  |  |  |  |  |  |  |  |  |  |  |  |  |  |  |  |  |
| 12 | *T. albopunctatum_*12 |  |  |  |  |  |  |  |  |  |  |  |  |  |  |  |  |  |  |  |  |  |  |  |  |  |  |  |  |  |  |  |  |  |  |  |  |  |  |  |  |  |  |  |  |  |  |  |  |  |  |
| 13 | *T. albopunctatum_*13 |  |  |  |  |  |  |  |  |  |  |  |  |  |  |  |  |  |  |  |  |  |  |  |  |  |  |  |  |  |  |  |  |  |  |  |  |  |  |  |  |  |  |  |  |  |  |  |  |  |  |
| 14 | *T. albopunctatum_*14 |  |  |  |  |  |  |  |  |  |  |  |  |  |  |  |  |  |  |  |  |  |  |  |  |  |  |  |  |  |  |  |  |  |  |  |  |  |  |  |  |  |  |  |  |  |  |  |  |  |  |
| 15 | *T. albopunctatum_*15 |  |  |  |  |  |  |  |  |  |  |  |  |  |  |  |  |  |  |  |  |  |  |  |  |  |  |  |  |  |  |  |  |  |  |  |  |  |  |  |  |  |  |  |  |  |  |  |  |  |  |
| 16 | *T. albopunctatum_*16 |  |  |  |  |  |  |  |  |  |  |  |  |  |  |  |  |  |  |  |  |  |  |  |  |  |  |  |  |  |  |  |  |  |  |  |  |  |  |  |  |  |  |  |  |  |  |  |  |  |  |
| 17 | *T. albopunctatum_*17 |  |  |  |  |  |  |  |  |  |  |  |  |  |  |  |  |  |  |  |  |  |  |  |  |  |  |  |  |  |  |  |  |  |  |  |  |  |  |  |  |  |  |  |  |  |  |  |  |  |  |
| 18 | *T. albopunctatum_*18 |  |  |  |  |  |  |  |  |  |  |  |  |  |  |  |  |  |  |  |  |  |  |  |  |  |  |  |  |  |  |  |  |  |  |  |  |  |  |  |  |  |  |  |  |  |  |  |  |  |  |
| 19 | *T. albopunctatum_*19 |  |  |  |  |  |  |  |  |  |  |  |  |  |  |  |  |  |  |  |  |  |  |  |  |  |  |  |  |  |  |  |  |  |  |  |  |  |  |  |  |  |  |  |  |  |  |  |  |  |  |
| 20 | *T. albopunctatum_*20 |  |  |  |  |  |  |  |  |  |  |  |  |  |  |  |  |  |  |  |  |  |  |  |  |  |  |  |  |  |  |  |  |  |  |  |  |  |  |  |  |  |  |  |  |  |  |  |  |  |  |
| 21 | *T. albopunctatum_*21 |  |  |  |  |  |  |  |  |  |  |  |  |  |  |  |  |  |  |  |  |  |  |  |  |  |  |  |  |  |  |  |  |  |  |  |  |  |  |  |  |  |  |  |  |  |  |  |  |  |  |
| 22 | *T. albopunctatum_*22 |  |  |  |  |  |  |  |  |  |  |  |  |  |  |  |  |  |  |  |  |  |  |  |  |  |  |  |  |  |  |  |  |  |  |  |  |  |  |  |  |  |  |  |  |  |  |  |  |  |  |
| 23 | *T. albopunctatum_*23 |  |  |  |  |  |  |  |  |  |  |  |  |  |  |  |  |  |  |  |  |  |  |  |  |  |  |  |  |  |  |  |  |  |  |  |  |  |  |  |  |  |  |  |  |  |  |  |  |  |  |
| 24 | *T. albopunctatum_*24 |  |  |  |  |  |  |  |  |  |  |  |  |  |  |  |  |  |  |  |  |  |  |  |  |  |  |  |  |  |  |  |  |  |  |  |  |  |  |  |  |  |  |  |  |  |  |  |  |  |  |
| 25 | *T. albopunctatum_*25 |  |  |  |  |  |  |  |  |  |  |  |  |  |  |  |  |  |  |  |  |  |  |  |  |  |  |  |  |  |  |  |  |  |  |  |  |  |  |  |  |  |  |  |  |  |  |  |  |  |  |
| 26 | *T. albopunctatum_*26 |  |  |  |  |  |  |  |  |  |  |  |  |  |  |  |  |  |  |  |  |  |  |  |  |  |  |  |  |  |  |  |  |  |  |  |  |  |  |  |  |  |  |  |  |  |  |  |  |  |  |
| 27 | *T. albopunctatum_*28 |  |  |  |  |  |  |  |  |  |  |  |  |  |  |  |  |  |  |  |  |  |  |  |  |  |  |  |  |  |  |  |  |  |  |  |  |  |  |  |  |  |  |  |  |  |  |  |  |  |  |
| 28 | *T. albopunctatum_*29 |  |  |  |  |  |  |  |  |  |  |  |  |  |  |  |  |  |  |  |  |  |  |  |  |  |  |  |  |  |  |  |  |  |  |  |  |  |  |  |  |  |  |  |  |  |  |  |  |  |  |
| 29 | *T. albopunctatum_*30 |  |  |  |  |  |  |  |  |  |  |  |  |  |  |  |  |  |  |  |  |  |  |  |  |  |  |  |  |  |  |  |  |  |  |  |  |  |  |  |  |  |  |  |  |  |  |  |  |  |  |
| 30 | *T. albopunctatum_*31 |  |  |  |  |  |  |  |  |  |  |  |  |  |  |  |  |  |  |  |  |  |  |  |  |  |  |  |  |  |  |  |  |  |  |  |  |  |  |  |  |  |  |  |  |  |  |  |  |  |  |
| 31 | *T. albopunctatum_*32 |  |  |  |  |  |  |  |  |  |  |  |  |  |  |  |  |  |  |  |  |  |  |  |  |  |  |  |  |  |  |  |  |  |  |  |  |  |  |  |  |  |  |  |  |  |  |  |  |  |  |
| 32 | *T. albopunctatum_*33 |  |  |  |  |  |  |  |  |  |  |  |  |  |  |  |  |  |  |  |  |  |  |  |  |  |  |  |  |  |  |  |  |  |  |  |  |  |  |  |  |  |  |  |  |  |  |  |  |  |  |
| 33 | *T. albopunctatum_*34 |  |  |  |  |  |  |  |  |  |  |  |  |  |  |  |  |  |  |  |  |  |  |  |  |  |  |  |  |  |  |  |  |  |  |  |  |  |  |  |  |  |  |  |  |  |  |  |  |  |  |
| 34 | *T. albopunctatum_*35 |  |  |  |  |  |  |  |  |  |  |  |  |  |  |  |  |  |  |  |  |  |  |  |  |  |  |  |  |  |  |  |  |  |  |  |  |  |  |  |  |  |  |  |  |  |  |  |  |  |  |
| 35 | *T. albopunctatum_*36 |  |  |  |  |  |  |  |  |  |  |  |  |  |  |  |  |  |  |  |  |  |  |  |  |  |  |  |  |  |  |  |  |  |  |  |  |  |  |  |  |  |  |  |  |  |  |  |  |  |  |
| 36 | *T. albopunctatum_*37 |  |  |  |  |  |  |  |  |  |  |  |  |  |  |  |  |  |  |  |  |  |  |  |  |  |  |  |  |  |  |  |  |  |  |  |  |  |  |  |  |  |  |  |  |  |  |  |  |  |  |
| 37 | *T. annae_*2 |  |  |  |  |  |  |  |  |  |  |  |  |  |  |  |  |  |  |  |  |  |  |  |  |  |  |  |  |  |  |  |  |  |  |  |  |  |  |  |  |  |  |  |  |  |  |  |  |  |  |
| 38 | *T. annae_*3 |  |  |  |  |  |  |  |  |  |  |  |  |  |  |  |  |  |  |  |  |  |  |  |  |  |  |  |  |  |  |  |  |  |  |  |  |  |  |  |  |  |  |  |  |  |  |  |  |  |  |
| 39 | *T. annae_*4 |  |  |  |  |  |  |  |  |  |  |  |  |  |  |  |  |  |  |  |  |  |  |  |  |  |  |  |  |  |  |  |  |  |  |  |  |  |  |  |  |  |  |  |  |  |  |  |  |  |  |
| 40 | *T. annae_*5 |  |  |  |  |  |  |  |  |  |  |  |  |  |  |  |  |  |  |  |  |  |  |  |  |  |  |  |  |  |  |  |  |  |  |  |  |  |  |  |  |  |  |  |  |  |  |  |  |  |  |
| 41 | *T. asperum_*1 |  |  |  |  |  |  |  |  |  |  |  |  |  |  |  |  |  |  |  |  |  |  |  |  |  |  |  |  |  |  |  |  |  |  |  |  |  |  |  |  |  |  |  |  |  |  |  |  |  |  |
| 42 | *T. asperum_*2 |  |  |  |  |  |  |  |  |  |  |  |  |  |  |  |  |  |  |  |  |  |  |  |  |  |  |  |  |  |  |  |  |  |  |  |  |  |  |  |  |  |  |  |  |  |  |  |  |  |  |
| 43 | *T. auratum_*1 |  |  |  |  |  |  |  |  |  |  |  |  |  |  |  |  |  |  |  |  |  |  |  |  |  |  |  |  |  |  |  |  |  |  |  |  |  |  |  |  |  |  |  |  |  |  |  |  |  |  |
| 44 | *T. auratum_*2 |  |  |  |  |  |  |  |  |  |  |  |  |  |  |  |  |  |  |  |  |  |  |  |  |  |  |  |  |  |  |  |  |  |  |  |  |  |  |  |  |  |  |  |  |  |  |  |  |  |  |
| 45 | *T. auratum_*3 |  |  |  |  |  |  |  |  |  |  |  |  |  |  |  |  |  |  |  |  |  |  |  |  |  |  |  |  |  |  |  |  |  |  |  |  |  |  |  |  |  |  |  |  |  |  |  |  |  |  |
| 46 | *T. auratum_*4 |  |  |  |  |  |  |  |  |  |  |  |  |  |  |  |  |  |  |  |  |  |  |  |  |  |  |  |  |  |  |  |  |  |  |  |  |  |  |  |  |  |  |  |  |  |  |  |  |  |  |
| 47 | *T. auratum_*5 |  |  |  |  |  |  |  |  |  |  |  |  |  |  |  |  |  |  |  |  |  |  |  |  |  |  |  |  |  |  |  |  |  |  |  |  |  |  |  |  |  |  |  |  |  |  |  |  |  |  |
| 48 | *T. auratum_*6 |  |  |  |  |  |  |  |  |  |  |  |  |  |  |  |  |  |  |  |  |  |  |  |  |  |  |  |  |  |  |  |  |  |  |  |  |  |  |  |  |  |  |  |  |  |  |  |  |  |  |
| 49 | *T. baibungense_*2 |  |  |  |  |  |  |  |  |  |  |  |  |  |  |  |  |  |  |  |  |  |  |  |  |  |  |  |  |  |  |  |  |  |  |  |  |  |  |  |  |  |  |  |  |  |  |  |  |  |  |
| 50 | *T. baibungense_*3 |  |  |  |  |  |  |  |  |  |  |  |  |  |  |  |  |  |  |  |  |  |  |  |  |  |  |  |  |  |  |  |  |  |  |  |  |  |  |  |  |  |  |  |  |  |  |  |  |  |  |
| 51 | *T. baibungense_*4 |  |  |  |  |  |  |  |  |  |  |  |  |  |  |  |  |  |  |  |  |  |  |  |  |  |  |  |  |  |  |  |  |  |  |  |  |  |  |  |  |  |  |  |  |  |  |  |  |  |  |
| 52 | *T. bicolor_*1 | 12.2 |  |  |  |  |  |  |  |  |  |  |  |  |  |  |  |  |  |  |  |  |  |  |  |  |  |  |  |  |  |  |  |  |  |  |  |  |  |  |  |  |  |  |  |  |  |  |  |  |  |
| 53 | *T. bicolor_*2 | 11.8 | 1.0 |  |  |  |  |  |  |  |  |  |  |  |  |  |  |  |  |  |  |  |  |  |  |  |  |  |  |  |  |  |  |  |  |  |  |  |  |  |  |  |  |  |  |  |  |  |  |  |  |
| 54 | *T. bicolor_*3 | 12.2 | 0.7 | 0.3 |  |  |  |  |  |  |  |  |  |  |  |  |  |  |  |  |  |  |  |  |  |  |  |  |  |  |  |  |  |  |  |  |  |  |  |  |  |  |  |  |  |  |  |  |  |  |  |
| 55 | *T. bicolor_*4 | 12.2 | 0.7 | 0.3 | 0.0 |  |  |  |  |  |  |  |  |  |  |  |  |  |  |  |  |  |  |  |  |  |  |  |  |  |  |  |  |  |  |  |  |  |  |  |  |  |  |  |  |  |  |  |  |  |  |
| 56 | *T. bicolor_*5 | 12.2 | 0.0 | 1.0 | 0.7 | 0.7 |  |  |  |  |  |  |  |  |  |  |  |  |  |  |  |  |  |  |  |  |  |  |  |  |  |  |  |  |  |  |  |  |  |  |  |  |  |  |  |  |  |  |  |  |  |
| 57 | *T. bicolor_*6 | 12.8 | 0.7 | 1.7 | 1.4 | 1.4 | 0.7 |  |  |  |  |  |  |  |  |  |  |  |  |  |  |  |  |  |  |  |  |  |  |  |  |  |  |  |  |  |  |  |  |  |  |  |  |  |  |  |  |  |  |  |  |
| 58 | *T. bicolor_*7 | 12.2 | 0.0 | 1.0 | 0.7 | 0.7 | 0.0 | 0.7 |  |  |  |  |  |  |  |  |  |  |  |  |  |  |  |  |  |  |  |  |  |  |  |  |  |  |  |  |  |  |  |  |  |  |  |  |  |  |  |  |  |  |  |
| 59 | *T. bicolor_*8 | 12.2 | 0.0 | 1.0 | 0.7 | 0.7 | 0.0 | 0.7 | 0.0 |  |  |  |  |  |  |  |  |  |  |  |  |  |  |  |  |  |  |  |  |  |  |  |  |  |  |  |  |  |  |  |  |  |  |  |  |  |  |  |  |  |  |
| 60 | *T. bicolor_*9 | 12.5 | 0.7 | 1.0 | 0.7 | 0.7 | 0.7 | 1.4 | 0.7 | 0.7 |  |  |  |  |  |  |  |  |  |  |  |  |  |  |  |  |  |  |  |  |  |  |  |  |  |  |  |  |  |  |  |  |  |  |  |  |  |  |  |  |  |
| 61 | *T. bicolor_*10 | 12.6 | 0.7 | 1.0 | 0.7 | 0.7 | 0.7 | 1.4 | 0.7 | 0.7 | 0.0 |  |  |  |  |  |  |  |  |  |  |  |  |  |  |  |  |  |  |  |  |  |  |  |  |  |  |  |  |  |  |  |  |  |  |  |  |  |  |  |  |
| 62 | *T. corticale_*1 | 14.2 | 5.9 | 5.6 | 5.9 | 5.9 | 5.9 | 6.6 | 5.9 | 5.9 | 5.2 | 5.2 |  |  |  |  |  |  |  |  |  |  |  |  |  |  |  |  |  |  |  |  |  |  |  |  |  |  |  |  |  |  |  |  |  |  |  |  |  |  |  |
| 63 | *T. corticale_*2 | 13.9 | 5.6 | 5.2 | 5.6 | 5.6 | 5.6 | 6.3 | 5.6 | 5.6 | 4.9 | 4.9 | 0.3 |  |  |  |  |  |  |  |  |  |  |  |  |  |  |  |  |  |  |  |  |  |  |  |  |  |  |  |  |  |  |  |  |  |  |  |  |  |  |
| 64 | *T. corticale_*3 | 14.9 | 6.6 | 6.9 | 7.3 | 7.3 | 6.6 | 7.3 | 6.6 | 6.6 | 6.6 | 6.6 | 2.1 | 2.4 |  |  |  |  |  |  |  |  |  |  |  |  |  |  |  |  |  |  |  |  |  |  |  |  |  |  |  |  |  |  |  |  |  |  |  |  |  |
| 65 | *T. corticale_*4 | 14.9 | 6.6 | 6.3 | 6.6 | 6.6 | 6.6 | 7.3 | 6.6 | 6.6 | 5.9 | 5.9 | 2.1 | 1.7 | 2.8 |  |  |  |  |  |  |  |  |  |  |  |  |  |  |  |  |  |  |  |  |  |  |  |  |  |  |  |  |  |  |  |  |  |  |  |  |
| 66 | *T. corticale_*5 | 14.9 | 6.6 | 6.3 | 6.6 | 6.6 | 6.6 | 7.3 | 6.6 | 6.6 | 5.9 | 5.9 | 2.1 | 1.7 | 2.8 | 0.0 |  |  |  |  |  |  |  |  |  |  |  |  |  |  |  |  |  |  |  |  |  |  |  |  |  |  |  |  |  |  |  |  |  |  |  |
| 67 | *T. corticale_*6 | 14.2 | 5.9 | 5.6 | 5.9 | 5.9 | 5.9 | 6.6 | 5.9 | 5.9 | 5.2 | 5.2 | 0.7 | 0.3 | 2.1 | 1.4 | 1.4 |  |  |  |  |  |  |  |  |  |  |  |  |  |  |  |  |  |  |  |  |  |  |  |  |  |  |  |  |  |  |  |  |  |  |
| 68 | *T. corticale_*7 | 14.2 | 5.9 | 5.6 | 5.9 | 5.9 | 5.9 | 6.6 | 5.9 | 5.9 | 5.2 | 5.2 | 0.7 | 0.3 | 2.1 | 1.4 | 1.4 | 0.0 |  |  |  |  |  |  |  |  |  |  |  |  |  |  |  |  |  |  |  |  |  |  |  |  |  |  |  |  |  |  |  |  |  |
| 69 | *T. corticale_*8 | 14.2 | 5.9 | 5.6 | 5.9 | 5.9 | 5.9 | 6.6 | 5.9 | 5.9 | 5.2 | 5.2 | 0.7 | 0.3 | 2.1 | 1.4 | 1.4 | 0.0 | 0.0 |  |  |  |  |  |  |  |  |  |  |  |  |  |  |  |  |  |  |  |  |  |  |  |  |  |  |  |  |  |  |  |  |
| 70 | *T. corticale_*9 | 14.2 | 5.9 | 5.6 | 5.9 | 5.9 | 5.9 | 6.6 | 5.9 | 5.9 | 5.2 | 5.2 | 0.7 | 0.3 | 2.1 | 1.4 | 1.4 | 0.0 | 0.0 | 0.0 |  |  |  |  |  |  |  |  |  |  |  |  |  |  |  |  |  |  |  |  |  |  |  |  |  |  |  |  |  |  |  |
| 71 | *T. corticale_*10 | 14.2 | 5.9 | 5.6 | 5.9 | 5.9 | 5.9 | 6.6 | 5.9 | 5.9 | 5.2 | 5.2 | 0.7 | 0.3 | 2.1 | 1.4 | 1.4 | 0.0 | 0.0 | 0.0 | 0.0 |  |  |  |  |  |  |  |  |  |  |  |  |  |  |  |  |  |  |  |  |  |  |  |  |  |  |  |  |  |  |
| 72 | *T. corticale_*11 | 14.2 | 5.9 | 5.6 | 5.9 | 5.9 | 5.9 | 6.6 | 5.9 | 5.9 | 5.2 | 5.2 | 0.7 | 0.3 | 2.1 | 1.4 | 1.4 | 0.0 | 0.0 | 0.0 | 0.0 | 0.0 |  |  |  |  |  |  |  |  |  |  |  |  |  |  |  |  |  |  |  |  |  |  |  |  |  |  |  |  |  |
| 73 | *T. corticale_*12 | 14.2 | 5.9 | 5.6 | 5.9 | 5.9 | 5.9 | 6.6 | 5.9 | 5.9 | 5.2 | 5.2 | 0.7 | 0.3 | 2.1 | 1.4 | 1.4 | 0.0 | 0.0 | 0.0 | 0.0 | 0.0 | 0.0 |  |  |  |  |  |  |  |  |  |  |  |  |  |  |  |  |  |  |  |  |  |  |  |  |  |  |  |  |
| 74 | *T. corticale_*13 | 14.2 | 5.9 | 5.6 | 5.9 | 5.9 | 5.9 | 6.6 | 5.9 | 5.9 | 5.2 | 5.2 | 0.7 | 0.3 | 2.1 | 1.4 | 1.4 | 0.0 | 0.0 | 0.0 | 0.0 | 0.0 | 0.0 | 0.0 |  |  |  |  |  |  |  |  |  |  |  |  |  |  |  |  |  |  |  |  |  |  |  |  |  |  |  |
| 75 | *T. corticale_*14 | 14.2 | 5.9 | 5.6 | 5.9 | 5.9 | 5.9 | 6.6 | 5.9 | 5.9 | 5.2 | 5.2 | 0.7 | 0.3 | 2.1 | 1.4 | 1.4 | 0.0 | 0.0 | 0.0 | 0.0 | 0.0 | 0.0 | 0.0 | 0.0 |  |  |  |  |  |  |  |  |  |  |  |  |  |  |  |  |  |  |  |  |  |  |  |  |  |  |
| 76 | *T. corticale_*15 | 14.2 | 5.9 | 5.6 | 5.9 | 5.9 | 5.9 | 6.6 | 5.9 | 5.9 | 5.2 | 5.2 | 0.7 | 0.3 | 2.1 | 1.4 | 1.4 | 0.0 | 0.0 | 0.0 | 0.0 | 0.0 | 0.0 | 0.0 | 0.0 | 0.0 |  |  |  |  |  |  |  |  |  |  |  |  |  |  |  |  |  |  |  |  |  |  |  |  |  |
| 77 | *T. corticale_*16 | 14.2 | 5.9 | 5.6 | 5.9 | 5.9 | 5.9 | 6.6 | 5.9 | 5.9 | 5.2 | 5.2 | 0.7 | 0.3 | 2.1 | 1.4 | 1.4 | 0.0 | 0.0 | 0.0 | 0.0 | 0.0 | 0.0 | 0.0 | 0.0 | 0.0 | 0.0 |  |  |  |  |  |  |  |  |  |  |  |  |  |  |  |  |  |  |  |  |  |  |  |  |
| 78 | *T. corticale_*17 | 14.2 | 5.9 | 5.6 | 5.9 | 5.9 | 5.9 | 6.6 | 5.9 | 5.9 | 5.2 | 5.2 | 0.7 | 0.3 | 2.1 | 1.4 | 1.4 | 0.0 | 0.0 | 0.0 | 0.0 | 0.0 | 0.0 | 0.0 | 0.0 | 0.0 | 0.0 | 0.0 |  |  |  |  |  |  |  |  |  |  |  |  |  |  |  |  |  |  |  |  |  |  |  |
| 79 | *T. corticale_*18 | 14.2 | 5.9 | 5.6 | 5.9 | 5.9 | 5.9 | 6.6 | 5.9 | 5.9 | 5.2 | 5.2 | 0.7 | 0.3 | 2.1 | 1.4 | 1.4 | 0.0 | 0.0 | 0.0 | 0.0 | 0.0 | 0.0 | 0.0 | 0.0 | 0.0 | 0.0 | 0.0 | 0.0 |  |  |  |  |  |  |  |  |  |  |  |  |  |  |  |  |  |  |  |  |  |  |
| 80 | *T. corticale_*19 | 14.6 | 6.3 | 5.9 | 6.3 | 6.3 | 6.3 | 6.9 | 6.3 | 6.3 | 5.6 | 5.6 | 1.0 | 0.7 | 2.4 | 1.7 | 1.7 | 0.3 | 0.3 | 0.3 | 0.3 | 0.3 | 0.3 | 0.3 | 0.3 | 0.3 | 0.3 | 0.3 | 0.3 | 0.3 |  |  |  |  |  |  |  |  |  |  |  |  |  |  |  |  |  |  |  |  |  |
| 81 | *T. corticale_*20 | 14.2 | 5.9 | 5.6 | 5.9 | 5.9 | 5.9 | 6.6 | 5.9 | 5.9 | 5.2 | 5.2 | 0.7 | 0.3 | 2.1 | 1.4 | 1.4 | 0.0 | 0.0 | 0.0 | 0.0 | 0.0 | 0.0 | 0.0 | 0.0 | 0.0 | 0.0 | 0.0 | 0.0 | 0.0 | 0.3 |  |  |  |  |  |  |  |  |  |  |  |  |  |  |  |  |  |  |  |  |
| 82 | *T. corticale_*21 | 14.2 | 5.9 | 5.6 | 5.9 | 5.9 | 5.9 | 6.6 | 5.9 | 5.9 | 5.2 | 5.2 | 0.7 | 0.3 | 2.1 | 1.4 | 1.4 | 0.0 | 0.0 | 0.0 | 0.0 | 0.0 | 0.0 | 0.0 | 0.0 | 0.0 | 0.0 | 0.0 | 0.0 | 0.0 | 0.3 | 0.0 |  |  |  |  |  |  |  |  |  |  |  |  |  |  |  |  |  |  |  |
| 83 | *T. corticale_*22 | 14.2 | 5.9 | 5.6 | 5.9 | 5.9 | 5.9 | 6.6 | 5.9 | 5.9 | 5.2 | 5.2 | 0.7 | 0.3 | 2.1 | 1.4 | 1.4 | 0.0 | 0.0 | 0.0 | 0.0 | 0.0 | 0.0 | 0.0 | 0.0 | 0.0 | 0.0 | 0.0 | 0.0 | 0.0 | 0.3 | 0.0 | 0.0 |  |  |  |  |  |  |  |  |  |  |  |  |  |  |  |  |  |  |
| 84 | *T. corticale_*23 | 14.2 | 5.9 | 5.6 | 5.9 | 5.9 | 5.9 | 6.6 | 5.9 | 5.9 | 5.2 | 5.2 | 0.7 | 0.3 | 2.1 | 1.4 | 1.4 | 0.0 | 0.0 | 0.0 | 0.0 | 0.0 | 0.0 | 0.0 | 0.0 | 0.0 | 0.0 | 0.0 | 0.0 | 0.0 | 0.3 | 0.0 | 0.0 | 0.0 |  |  |  |  |  |  |  |  |  |  |  |  |  |  |  |  |  |
| 85 | *T. gordoni_*1 | 11.1 | 8.7 | 8.7 | 9.0 | 9.0 | 8.7 | 9.4 | 8.7 | 8.7 | 8.3 | 8.4 | 10.8 | 10.4 | 11.1 | 10.8 | 10.8 | 10.8 | 10.8 | 10.8 | 10.8 | 10.8 | 10.8 | 10.8 | 10.8 | 10.8 | 10.8 | 10.8 | 10.8 | 10.8 | 11.1 | 10.8 | 10.8 | 10.8 | 10.8 |  |  |  |  |  |  |  |  |  |  |  |  |  |  |  |  |
| 86 | *T. gordoni_*2 | 11.0 | 8.2 | 8.2 | 8.2 | 8.2 | 8.2 | 8.9 | 8.2 | 8.2 | 7.8 | 7.9 | 10.6 | 10.3 | 11.3 | 10.3 | 10.3 | 10.6 | 10.6 | 10.6 | 10.6 | 10.6 | 10.6 | 10.6 | 10.6 | 10.6 | 10.6 | 10.6 | 10.6 | 10.6 | 11.0 | 10.6 | 10.6 | 10.6 | 10.6 | 4.6 |  |  |  |  |  |  |  |  |  |  |  |  |  |  |  |
| 87 | *T. gordoni_*3 | 11.2 | 9.1 | 8.7 | 9.1 | 9.1 | 9.1 | 9.8 | 9.1 | 9.1 | 8.7 | 8.8 | 10.8 | 10.5 | 11.5 | 10.5 | 10.5 | 10.8 | 10.8 | 10.8 | 10.8 | 10.8 | 10.8 | 10.8 | 10.8 | 10.8 | 10.8 | 10.8 | 10.8 | 10.8 | 11.2 | 10.8 | 10.8 | 10.8 | 10.8 | 4.5 | 1.4 |  |  |  |  |  |  |  |  |  |  |  |  |  |  |
| 88 | *T. gordoni_*4 | 10.8 | 8.7 | 8.4 | 8.7 | 8.7 | 8.7 | 9.4 | 8.7 | 8.7 | 8.4 | 8.5 | 10.5 | 10.1 | 11.2 | 10.1 | 10.1 | 10.5 | 10.5 | 10.5 | 10.5 | 10.5 | 10.5 | 10.5 | 10.5 | 10.5 | 10.5 | 10.5 | 10.5 | 10.5 | 10.8 | 10.5 | 10.5 | 10.5 | 10.5 | 4.2 | 1.1 | 0.3 |  |  |  |  |  |  |  |  |  |  |  |  |  |
| 89 | *T. gordoni_*5 | 10.9 | 8.8 | 8.4 | 8.8 | 8.8 | 8.8 | 9.5 | 8.8 | 8.8 | 8.4 | 8.5 | 10.5 | 10.2 | 11.2 | 10.2 | 10.2 | 10.5 | 10.5 | 10.5 | 10.5 | 10.5 | 10.5 | 10.5 | 10.5 | 10.5 | 10.5 | 10.5 | 10.5 | 10.5 | 10.9 | 10.5 | 10.5 | 10.5 | 10.5 | 3.9 | 1.1 | 0.4 | 0.0 |  |  |  |  |  |  |  |  |  |  |  |  |
| 90 | *T. gordoni_*6 | 10.2 | 9.2 | 8.8 | 9.2 | 9.2 | 9.2 | 9.9 | 9.2 | 9.2 | 8.8 | 8.9 | 10.9 | 10.6 | 11.6 | 10.6 | 10.6 | 10.9 | 10.9 | 10.9 | 10.9 | 10.9 | 10.9 | 10.9 | 10.9 | 10.9 | 10.9 | 10.9 | 10.9 | 10.9 | 11.3 | 10.9 | 10.9 | 10.9 | 10.9 | 4.6 | 1.4 | 0.7 | 0.4 | 0.4 |  |  |  |  |  |  |  |  |  |  |  |
| 91 | *T. gordoni_*7 | 10.8 | 8.7 | 8.4 | 8.7 | 8.7 | 8.7 | 9.4 | 8.7 | 8.7 | 8.4 | 8.5 | 10.5 | 10.1 | 11.9 | 10.8 | 10.8 | 10.5 | 10.5 | 10.5 | 10.5 | 10.5 | 10.5 | 10.5 | 10.5 | 10.5 | 10.5 | 10.5 | 10.5 | 10.5 | 10.8 | 10.5 | 10.5 | 10.5 | 10.5 | 4.9 | 1.8 | 1.0 | 0.7 | 0.7 | 1.1 |  |  |  |  |  |  |  |  |  |  |
| 92 | *T. gordoni_*8 | 10.8 | 8.7 | 8.4 | 8.7 | 8.7 | 8.7 | 9.4 | 8.7 | 8.7 | 8.4 | 8.5 | 10.5 | 10.1 | 11.9 | 10.8 | 10.8 | 10.5 | 10.5 | 10.5 | 10.5 | 10.5 | 10.5 | 10.5 | 10.5 | 10.5 | 10.5 | 10.5 | 10.5 | 10.5 | 10.8 | 10.5 | 10.5 | 10.5 | 10.5 | 4.9 | 1.8 | 1.0 | 0.7 | 0.7 | 1.1 | 0.0 |  |  |  |  |  |  |  |  |  |
| 93 | *T. hekouense_*1 | 11.1 | 7.3 | 7.3 | 7.6 | 7.6 | 7.3 | 8.0 | 7.3 | 7.3 | 7.6 | 7.7 | 11.5 | 11.1 | 11.8 | 12.2 | 12.2 | 11.5 | 11.5 | 11.5 | 11.5 | 11.5 | 11.5 | 11.5 | 11.5 | 11.5 | 11.5 | 11.5 | 11.5 | 11.5 | 11.8 | 11.5 | 11.5 | 11.5 | 11.5 | 10.1 | 10.6 | 10.8 | 10.8 | 10.9 | 10.2 | 10.8 | 10.8 |  |  |  |  |  |  |  |  |
| 94 | *T. hekouense_*2 | 11.1 | 7.3 | 7.3 | 7.6 | 7.6 | 7.3 | 8.0 | 7.3 | 7.3 | 7.6 | 7.7 | 11.5 | 11.1 | 11.8 | 12.2 | 12.2 | 11.5 | 11.5 | 11.5 | 11.5 | 11.5 | 11.5 | 11.5 | 11.5 | 11.5 | 11.5 | 11.5 | 11.5 | 11.5 | 11.8 | 11.5 | 11.5 | 11.5 | 11.5 | 10.1 | 10.6 | 10.8 | 10.8 | 10.9 | 10.2 | 10.8 | 10.8 | 0.0 |  |  |  |  |  |  |  |
| 95 | *T. hekouense_*3 | 11.1 | 6.9 | 6.9 | 7.3 | 7.3 | 6.9 | 7.6 | 6.9 | 6.9 | 7.3 | 7.3 | 11.5 | 11.1 | 11.8 | 12.2 | 12.2 | 11.5 | 11.5 | 11.5 | 11.5 | 11.5 | 11.5 | 11.5 | 11.5 | 11.5 | 11.5 | 11.5 | 11.5 | 11.5 | 11.8 | 11.5 | 11.5 | 11.5 | 11.5 | 10.1 | 10.6 | 10.8 | 10.8 | 10.9 | 10.2 | 10.8 | 10.8 | 0.3 | 0.3 |  |  |  |  |  |  |
| 96 | *T. hekouense_*4 | 11.1 | 6.9 | 6.9 | 7.3 | 7.3 | 6.9 | 7.6 | 6.9 | 6.9 | 7.3 | 7.3 | 11.5 | 11.1 | 11.8 | 12.2 | 12.2 | 11.5 | 11.5 | 11.5 | 11.5 | 11.5 | 11.5 | 11.5 | 11.5 | 11.5 | 11.5 | 11.5 | 11.5 | 11.5 | 11.8 | 11.5 | 11.5 | 11.5 | 11.5 | 10.1 | 10.6 | 10.8 | 10.8 | 10.9 | 10.2 | 10.8 | 10.8 | 0.3 | 0.3 | 0.0 |  |  |  |  |  |
| 97 | *T. hekouense_*5 | 11.1 | 7.3 | 7.3 | 7.6 | 7.6 | 7.3 | 8.0 | 7.3 | 7.3 | 7.6 | 7.7 | 11.5 | 11.1 | 11.8 | 12.2 | 12.2 | 11.5 | 11.5 | 11.5 | 11.5 | 11.5 | 11.5 | 11.5 | 11.5 | 11.5 | 11.5 | 11.5 | 11.5 | 11.5 | 11.8 | 11.5 | 11.5 | 11.5 | 11.5 | 10.1 | 10.6 | 10.8 | 10.8 | 10.9 | 10.2 | 10.8 | 10.8 | 0.0 | 0.0 | 0.3 | 0.3 |  |  |  |  |
| 98 | *T. hekouense_*6 | 11.1 | 7.3 | 7.3 | 7.6 | 7.6 | 7.3 | 8.0 | 7.3 | 7.3 | 7.6 | 7.7 | 11.5 | 11.1 | 11.8 | 12.2 | 12.2 | 11.5 | 11.5 | 11.5 | 11.5 | 11.5 | 11.5 | 11.5 | 11.5 | 11.5 | 11.5 | 11.5 | 11.5 | 11.5 | 11.8 | 11.5 | 11.5 | 11.5 | 11.5 | 10.1 | 10.6 | 10.8 | 10.8 | 10.9 | 10.2 | 10.8 | 10.8 | 0.0 | 0.0 | 0.3 | 0.3 | 0.0 |  |  |  |
| 99 | *T. hekouense_*7 | 11.5 | 7.6 | 7.6 | 8.0 | 8.0 | 7.6 | 8.3 | 7.6 | 7.6 | 8.0 | 8.0 | 11.8 | 11.5 | 12.2 | 12.5 | 12.5 | 11.8 | 11.8 | 11.8 | 11.8 | 11.8 | 11.8 | 11.8 | 11.8 | 11.8 | 11.8 | 11.8 | 11.8 | 11.8 | 12.2 | 11.8 | 11.8 | 11.8 | 11.8 | 10.4 | 11.0 | 11.2 | 11.2 | 11.2 | 10.6 | 11.2 | 11.2 | 0.3 | 0.3 | 0.7 | 0.7 | 0.3 | 0.3 |  |  |
| 100 | *T. hekouense_*8 | 11.1 | 6.6 | 6.6 | 6.9 | 6.9 | 6.6 | 7.3 | 6.6 | 6.6 | 6.9 | 7.0 | 11.1 | 10.8 | 11.5 | 11.8 | 11.8 | 11.1 | 11.1 | 11.1 | 11.1 | 11.1 | 11.1 | 11.1 | 11.1 | 11.1 | 11.1 | 11.1 | 11.1 | 11.1 | 11.5 | 11.1 | 11.1 | 11.1 | 11.1 | 10.4 | 10.3 | 10.5 | 10.5 | 10.5 | 9.9 | 10.5 | 10.5 | 0.7 | 0.7 | 0.3 | 0.3 | 0.7 | 0.7 | 1.0 |  |
| 101 | *T. hekouense_*9 | 11.1 | 6.9 | 6.9 | 7.3 | 7.3 | 6.9 | 7.6 | 6.9 | 6.9 | 7.3 | 7.3 | 11.5 | 11.1 | 11.8 | 12.2 | 12.2 | 11.5 | 11.5 | 11.5 | 11.5 | 11.5 | 11.5 | 11.5 | 11.5 | 11.5 | 11.5 | 11.5 | 11.5 | 11.5 | 11.8 | 11.5 | 11.5 | 11.5 | 11.5 | 10.1 | 10.6 | 10.8 | 10.8 | 10.9 | 10.2 | 10.8 | 10.8 | 0.3 | 0.3 | 0.0 | 0.0 | 0.3 | 0.3 | 0.7 | 0.3 |
| 102 | *T. hekouense_*10 | 11.1 | 6.9 | 6.9 | 7.3 | 7.3 | 6.9 | 7.6 | 6.9 | 6.9 | 7.3 | 7.3 | 11.5 | 11.1 | 11.8 | 12.2 | 12.2 | 11.5 | 11.5 | 11.5 | 11.5 | 11.5 | 11.5 | 11.5 | 11.5 | 11.5 | 11.5 | 11.5 | 11.5 | 11.5 | 11.8 | 11.5 | 11.5 | 11.5 | 11.5 | 10.1 | 10.6 | 10.8 | 10.8 | 10.9 | 10.2 | 10.8 | 10.8 | 0.3 | 0.3 | 0.0 | 0.0 | 0.3 | 0.3 | 0.7 | 0.3 |
| 103 | *T. horridum_*1 | 18.1 | 12.8 | 13.2 | 12.8 | 12.8 | 12.8 | 13.5 | 12.8 | 12.8 | 12.2 | 12.2 | 16.7 | 16.3 | 17.4 | 16.3 | 16.3 | 16.7 | 16.7 | 16.7 | 16.7 | 16.7 | 16.7 | 16.7 | 16.7 | 16.7 | 16.7 | 16.7 | 16.7 | 16.7 | 17.0 | 16.7 | 16.7 | 16.7 | 16.7 | 16.3 | 15.6 | 15.4 | 15.0 | 15.1 | 15.5 | 15.0 | 15.0 | 15.6 | 15.6 | 15.3 | 15.3 | 15.6 | 15.6 | 16.0 | 14.9 |
| 104 | *T. horridum_*2 | 18.1 | 12.8 | 13.2 | 12.8 | 12.8 | 12.8 | 13.5 | 12.8 | 12.8 | 12.2 | 12.2 | 16.7 | 16.3 | 17.4 | 16.3 | 16.3 | 16.7 | 16.7 | 16.7 | 16.7 | 16.7 | 16.7 | 16.7 | 16.7 | 16.7 | 16.7 | 16.7 | 16.7 | 16.7 | 17.0 | 16.7 | 16.7 | 16.7 | 16.7 | 16.3 | 15.6 | 15.4 | 15.0 | 15.1 | 15.5 | 15.0 | 15.0 | 15.6 | 15.6 | 15.3 | 15.3 | 15.6 | 15.6 | 16.0 | 14.9 |
| 105 | *T. horridum_*3 | 17.4 | 12.5 | 12.8 | 12.5 | 12.5 | 12.5 | 13.2 | 12.5 | 12.5 | 11.8 | 11.9 | 16.3 | 16.0 | 17.0 | 16.0 | 16.0 | 16.3 | 16.3 | 16.3 | 16.3 | 16.3 | 16.3 | 16.3 | 16.3 | 16.3 | 16.3 | 16.3 | 16.3 | 16.3 | 16.7 | 16.3 | 16.3 | 16.3 | 16.3 | 15.6 | 14.9 | 14.7 | 14.3 | 14.4 | 14.8 | 14.3 | 14.3 | 15.6 | 15.6 | 15.3 | 15.3 | 15.6 | 15.6 | 16.0 | 14.9 |
| 106 | *T. horridum5* | 17.7 | 13.2 | 13.5 | 13.2 | 13.2 | 13.2 | 13.9 | 13.2 | 13.2 | 12.5 | 12.6 | 17.0 | 16.7 | 17.7 | 16.7 | 16.7 | 17.0 | 17.0 | 17.0 | 17.0 | 17.0 | 17.0 | 17.0 | 17.0 | 17.0 | 17.0 | 17.0 | 17.0 | 17.0 | 17.4 | 17.0 | 17.0 | 17.0 | 17.0 | 16.0 | 15.2 | 15.0 | 14.7 | 14.7 | 15.1 | 14.7 | 14.7 | 16.0 | 16.0 | 15.6 | 15.6 | 16.0 | 16.0 | 16.3 | 15.3 |
| 107 | *T. khoii_*1 | 13.5 | 2.8 | 3.1 | 2.8 | 2.8 | 2.8 | 3.5 | 2.8 | 2.8 | 2.1 | 2.1 | 5.6 | 5.2 | 6.3 | 5.6 | 5.6 | 5.6 | 5.6 | 5.6 | 5.6 | 5.6 | 5.6 | 5.6 | 5.6 | 5.6 | 5.6 | 5.6 | 5.6 | 5.6 | 5.9 | 5.6 | 5.6 | 5.6 | 5.6 | 8.0 | 8.2 | 9.1 | 8.7 | 8.4 | 9.2 | 9.4 | 9.4 | 8.3 | 8.3 | 8.7 | 8.7 | 8.3 | 8.3 | 8.7 | 8.3 |
| 108 | *T. khoii*2 | 13.5 | 2.8 | 3.1 | 2.8 | 2.8 | 2.8 | 3.5 | 2.8 | 2.8 | 2.1 | 2.1 | 5.6 | 5.2 | 6.3 | 5.6 | 5.6 | 5.6 | 5.6 | 5.6 | 5.6 | 5.6 | 5.6 | 5.6 | 5.6 | 5.6 | 5.6 | 5.6 | 5.6 | 5.6 | 5.9 | 5.6 | 5.6 | 5.6 | 5.6 | 8.0 | 8.2 | 9.1 | 8.7 | 8.4 | 9.2 | 9.4 | 9.4 | 8.3 | 8.3 | 8.7 | 8.7 | 8.3 | 8.3 | 8.7 | 8.3 |
| 109 | *T. lacustrinum_*1 | 13.5 | 8.0 | 8.7 | 8.3 | 8.3 | 8.0 | 8.0 | 8.0 | 8.0 | 8.3 | 8.4 | 11.5 | 11.1 | 12.2 | 12.5 | 12.5 | 11.5 | 11.5 | 11.5 | 11.5 | 11.5 | 11.5 | 11.5 | 11.5 | 11.5 | 11.5 | 11.5 | 11.5 | 11.5 | 11.8 | 11.5 | 11.5 | 11.5 | 11.5 | 11.1 | 11.3 | 12.2 | 11.9 | 11.9 | 12.3 | 11.2 | 11.2 | 10.4 | 10.4 | 10.8 | 10.8 | 10.4 | 10.4 | 10.8 | 10.8 |
| 110 | *T. lacustrinum_*2 | 13.5 | 8.0 | 8.7 | 8.3 | 8.3 | 8.0 | 8.0 | 8.0 | 8.0 | 8.3 | 8.4 | 11.5 | 11.1 | 12.2 | 12.5 | 12.5 | 11.5 | 11.5 | 11.5 | 11.5 | 11.5 | 11.5 | 11.5 | 11.5 | 11.5 | 11.5 | 11.5 | 11.5 | 11.5 | 11.8 | 11.5 | 11.5 | 11.5 | 11.5 | 11.1 | 11.3 | 12.2 | 11.9 | 11.9 | 12.3 | 11.2 | 11.2 | 10.4 | 10.4 | 10.8 | 10.8 | 10.4 | 10.4 | 10.8 | 10.8 |
| 111 | *T. laeve_*1 | 13.2 | 11.8 | 11.5 | 11.8 | 11.8 | 11.8 | 11.8 | 11.8 | 11.8 | 11.5 | 11.5 | 13.5 | 13.2 | 14.2 | 14.6 | 14.6 | 13.5 | 13.5 | 13.5 | 13.5 | 13.5 | 13.5 | 13.5 | 13.5 | 13.5 | 13.5 | 13.5 | 13.5 | 13.5 | 13.9 | 13.5 | 13.5 | 13.5 | 13.5 | 11.1 | 11.7 | 11.9 | 11.5 | 11.2 | 12.0 | 11.5 | 11.5 | 12.8 | 12.8 | 12.5 | 12.5 | 12.8 | 12.8 | 13.2 | 12.2 |
| 112 | *T. laeve_*2 | 15.6 | 15.3 | 15.3 | 15.6 | 15.6 | 15.3 | 15.3 | 15.3 | 15.3 | 15.6 | 15.7 | 15.6 | 15.3 | 16.0 | 16.3 | 16.3 | 15.6 | 15.6 | 15.6 | 15.6 | 15.6 | 15.6 | 15.6 | 15.6 | 15.6 | 15.6 | 15.6 | 15.6 | 15.6 | 16.0 | 15.6 | 15.6 | 15.6 | 15.6 | 14.9 | 15.6 | 15.4 | 15.0 | 14.7 | 15.5 | 15.0 | 15.0 | 14.9 | 14.9 | 14.6 | 14.6 | 14.9 | 14.9 | 15.3 | 14.2 |
| 113 | *T. laeve_*3 | 15.6 | 15.3 | 15.3 | 15.6 | 15.6 | 15.3 | 15.3 | 15.3 | 15.3 | 15.6 | 15.7 | 15.6 | 15.3 | 16.0 | 16.3 | 16.3 | 15.6 | 15.6 | 15.6 | 15.6 | 15.6 | 15.6 | 15.6 | 15.6 | 15.6 | 15.6 | 15.6 | 15.6 | 15.6 | 16.0 | 15.6 | 15.6 | 15.6 | 15.6 | 14.9 | 15.6 | 15.4 | 15.0 | 14.7 | 15.5 | 15.0 | 15.0 | 15.6 | 15.6 | 15.3 | 15.3 | 15.6 | 15.6 | 16.0 | 14.9 |
| 114 | *T. laeve_*4 | 16.0 | 16.0 | 16.0 | 16.3 | 16.3 | 16.0 | 16.0 | 16.0 | 16.0 | 16.3 | 16.4 | 16.3 | 16.0 | 16.7 | 17.0 | 17.0 | 16.3 | 16.3 | 16.3 | 16.3 | 16.3 | 16.3 | 16.3 | 16.3 | 16.3 | 16.3 | 16.3 | 16.3 | 16.3 | 16.7 | 16.3 | 16.3 | 16.3 | 16.3 | 14.9 | 16.3 | 16.1 | 15.7 | 15.4 | 16.2 | 15.7 | 15.7 | 14.9 | 14.9 | 14.6 | 14.6 | 14.9 | 14.9 | 15.3 | 14.9 |
| 115 | *T. laeve_*5 | 15.6 | 15.6 | 15.6 | 16.0 | 16.0 | 15.6 | 15.6 | 15.6 | 15.6 | 16.0 | 16.1 | 16.0 | 15.6 | 16.3 | 16.7 | 16.7 | 16.0 | 16.0 | 16.0 | 16.0 | 16.0 | 16.0 | 16.0 | 16.0 | 16.0 | 16.0 | 16.0 | 16.0 | 16.0 | 16.3 | 16.0 | 16.0 | 16.0 | 16.0 | 15.3 | 16.7 | 16.4 | 16.1 | 15.8 | 16.5 | 16.1 | 16.1 | 14.6 | 14.6 | 14.2 | 14.2 | 14.6 | 14.6 | 14.9 | 14.6 |
| 116 | *T. laeve_*6 | 15.6 | 15.6 | 15.6 | 16.0 | 16.0 | 15.6 | 15.6 | 15.6 | 15.6 | 16.0 | 16.1 | 16.0 | 15.6 | 16.3 | 16.7 | 16.7 | 16.0 | 16.0 | 16.0 | 16.0 | 16.0 | 16.0 | 16.0 | 16.0 | 16.0 | 16.0 | 16.0 | 16.0 | 16.0 | 16.3 | 16.0 | 16.0 | 16.0 | 16.0 | 15.3 | 16.7 | 16.4 | 16.1 | 15.8 | 16.5 | 16.1 | 16.1 | 14.6 | 14.6 | 14.2 | 14.2 | 14.6 | 14.6 | 14.9 | 14.6 |
| 117 | *T. laeve_*7 | 15.6 | 15.6 | 15.6 | 16.0 | 16.0 | 15.6 | 15.6 | 15.6 | 15.6 | 16.0 | 16.1 | 16.0 | 15.6 | 16.3 | 16.7 | 16.7 | 16.0 | 16.0 | 16.0 | 16.0 | 16.0 | 16.0 | 16.0 | 16.0 | 16.0 | 16.0 | 16.0 | 16.0 | 16.0 | 16.3 | 16.0 | 16.0 | 16.0 | 16.0 | 15.3 | 16.7 | 16.4 | 16.1 | 15.8 | 16.5 | 16.1 | 16.1 | 14.6 | 14.6 | 14.2 | 14.2 | 14.6 | 14.6 | 14.9 | 14.6 |
| 118 | *T. laeve_*8 | 16.3 | 16.3 | 16.3 | 16.7 | 16.7 | 16.3 | 16.3 | 16.3 | 16.3 | 16.7 | 16.8 | 16.7 | 16.3 | 17.0 | 17.4 | 17.4 | 16.7 | 16.7 | 16.7 | 16.7 | 16.7 | 16.7 | 16.7 | 16.7 | 16.7 | 16.7 | 16.7 | 16.7 | 16.7 | 17.0 | 16.7 | 16.7 | 16.7 | 16.7 | 15.3 | 16.7 | 16.4 | 16.1 | 15.8 | 16.5 | 16.1 | 16.1 | 15.3 | 15.3 | 14.9 | 14.9 | 15.3 | 15.3 | 15.6 | 15.3 |
| 119 | *T. laeve_*9 | 16.9 | 17.3 | 17.7 | 17.7 | 17.7 | 17.3 | 17.3 | 17.3 | 17.3 | 17.7 | 17.9 | 17.7 | 17.3 | 18.1 | 18.5 | 18.5 | 17.7 | 17.7 | 17.7 | 17.7 | 17.7 | 17.7 | 17.7 | 17.7 | 17.7 | 17.7 | 17.7 | 17.7 | 17.7 | 18.1 | 17.7 | 17.7 | 17.7 | 17.7 | 16.5 | 17.8 | 17.9 | 17.5 | 17.1 | 18.0 | 17.5 | 17.5 | 16.5 | 16.5 | 16.1 | 16.1 | 16.5 | 16.5 | 16.5 | 16.5 |
| 120 | *T. laeve_*10 | 16.3 | 16.3 | 16.3 | 16.7 | 16.7 | 16.3 | 16.3 | 16.3 | 16.3 | 16.7 | 16.8 | 16.7 | 16.3 | 17.0 | 17.4 | 17.4 | 16.7 | 16.7 | 16.7 | 16.7 | 16.7 | 16.7 | 16.7 | 16.7 | 16.7 | 16.7 | 16.7 | 16.7 | 16.7 | 17.0 | 16.7 | 16.7 | 16.7 | 16.7 | 16.0 | 17.4 | 17.1 | 16.8 | 16.5 | 17.3 | 16.8 | 16.8 | 15.3 | 15.3 | 14.9 | 14.9 | 15.3 | 15.3 | 15.6 | 15.3 |
| 121 | *T. lateriticum_*1 | 14.6 | 9.4 | 10.1 | 9.7 | 9.7 | 9.4 | 9.4 | 9.4 | 9.4 | 9.7 | 9.8 | 13.2 | 12.8 | 13.9 | 13.9 | 13.9 | 12.8 | 12.8 | 12.8 | 12.8 | 12.8 | 12.8 | 12.8 | 12.8 | 12.8 | 12.8 | 12.8 | 12.8 | 12.8 | 13.2 | 12.8 | 12.8 | 12.8 | 12.8 | 14.2 | 12.8 | 13.3 | 12.9 | 13.0 | 13.0 | 12.2 | 12.2 | 12.8 | 12.8 | 12.5 | 12.5 | 12.8 | 12.8 | 13.2 | 12.2 |
| 122 | *T. lateriticum_*2 | 13.2 | 9.7 | 10.4 | 10.1 | 10.1 | 9.7 | 9.7 | 9.7 | 9.7 | 10.1 | 10.1 | 14.2 | 13.9 | 14.2 | 14.2 | 14.2 | 13.9 | 13.9 | 13.9 | 13.9 | 13.9 | 13.9 | 13.9 | 13.9 | 13.9 | 13.9 | 13.9 | 13.9 | 13.9 | 14.2 | 13.9 | 13.9 | 13.9 | 13.9 | 14.2 | 13.1 | 13.6 | 13.3 | 13.3 | 13.4 | 12.6 | 12.6 | 12.5 | 12.5 | 12.2 | 12.2 | 12.5 | 12.5 | 12.8 | 11.8 |
| 123 | *T. lateriticum_*3 | 13.2 | 8.7 | 9.4 | 9.0 | 9.0 | 8.7 | 8.7 | 8.7 | 8.7 | 9.0 | 9.1 | 13.2 | 12.8 | 13.2 | 13.9 | 13.9 | 12.8 | 12.8 | 12.8 | 12.8 | 12.8 | 12.8 | 12.8 | 12.8 | 12.8 | 12.8 | 12.8 | 12.8 | 12.8 | 13.2 | 12.8 | 12.8 | 12.8 | 12.8 | 13.2 | 12.1 | 12.6 | 12.2 | 12.3 | 12.3 | 11.5 | 11.5 | 10.8 | 10.8 | 10.4 | 10.4 | 10.8 | 10.8 | 11.1 | 10.1 |
| 124 | *T. lateriticum_*4 | 14.6 | 8.7 | 9.4 | 9.0 | 9.0 | 8.7 | 8.7 | 8.7 | 8.7 | 8.3 | 8.4 | 12.5 | 12.2 | 13.2 | 13.2 | 13.2 | 12.2 | 12.2 | 12.2 | 12.2 | 12.2 | 12.2 | 12.2 | 12.2 | 12.2 | 12.2 | 12.2 | 12.2 | 12.2 | 12.5 | 12.2 | 12.2 | 12.2 | 12.2 | 13.5 | 12.1 | 12.6 | 12.2 | 12.3 | 12.3 | 11.5 | 11.5 | 12.8 | 12.8 | 12.5 | 12.5 | 12.8 | 12.8 | 13.2 | 12.2 |
| 125 | *T. lateriticum_*5 | 13.9 | 8.0 | 8.7 | 8.3 | 8.3 | 8.0 | 8.0 | 8.0 | 8.0 | 8.3 | 8.4 | 12.5 | 12.2 | 13.2 | 13.2 | 13.2 | 12.2 | 12.2 | 12.2 | 12.2 | 12.2 | 12.2 | 12.2 | 12.2 | 12.2 | 12.2 | 12.2 | 12.2 | 12.2 | 12.5 | 12.2 | 12.2 | 12.2 | 12.2 | 13.9 | 12.8 | 12.9 | 12.6 | 12.6 | 12.7 | 11.9 | 11.9 | 11.8 | 11.8 | 11.5 | 11.5 | 11.8 | 11.8 | 12.2 | 11.1 |
| 126 | *T. lateriticum_*6 | 13.9 | 8.0 | 8.7 | 8.3 | 8.3 | 8.0 | 8.0 | 8.0 | 8.0 | 8.3 | 8.4 | 12.5 | 12.2 | 13.2 | 13.2 | 13.2 | 12.2 | 12.2 | 12.2 | 12.2 | 12.2 | 12.2 | 12.2 | 12.2 | 12.2 | 12.2 | 12.2 | 12.2 | 12.2 | 12.5 | 12.2 | 12.2 | 12.2 | 12.2 | 13.9 | 12.8 | 12.9 | 12.6 | 12.6 | 12.7 | 11.9 | 11.9 | 11.8 | 11.8 | 11.5 | 11.5 | 11.8 | 11.8 | 12.2 | 11.1 |
| 127 | *T. lateriticum_*LT01 | 13.5 | 8.3 | 9.0 | 8.7 | 8.7 | 8.3 | 8.3 | 8.3 | 8.3 | 8.7 | 8.7 | 12.8 | 12.5 | 12.8 | 13.5 | 13.5 | 12.5 | 12.5 | 12.5 | 12.5 | 12.5 | 12.5 | 12.5 | 12.5 | 12.5 | 12.5 | 12.5 | 12.5 | 12.5 | 12.8 | 12.5 | 12.5 | 12.5 | 12.5 | 13.5 | 12.4 | 12.9 | 12.6 | 12.6 | 12.7 | 11.9 | 11.9 | 11.8 | 11.8 | 11.5 | 11.5 | 11.8 | 11.8 | 12.2 | 11.1 |
| 128 | *T. lateriticum_*LT011 | 13.5 | 8.3 | 9.0 | 8.7 | 8.7 | 8.3 | 8.3 | 8.3 | 8.3 | 8.7 | 8.7 | 12.8 | 12.5 | 12.8 | 13.5 | 13.5 | 12.5 | 12.5 | 12.5 | 12.5 | 12.5 | 12.5 | 12.5 | 12.5 | 12.5 | 12.5 | 12.5 | 12.5 | 12.5 | 12.8 | 12.5 | 12.5 | 12.5 | 12.5 | 13.5 | 12.4 | 12.9 | 12.6 | 12.6 | 12.7 | 11.9 | 11.9 | 11.8 | 11.8 | 11.5 | 11.5 | 11.8 | 11.8 | 12.2 | 11.1 |
| 129 | *T. leporosum_*1 | 11.1 | 8.7 | 9.4 | 9.0 | 9.0 | 8.7 | 9.4 | 8.7 | 8.7 | 8.3 | 8.4 | 10.1 | 9.7 | 10.8 | 11.1 | 11.1 | 10.1 | 10.1 | 10.1 | 10.1 | 10.1 | 10.1 | 10.1 | 10.1 | 10.1 | 10.1 | 10.1 | 10.1 | 10.1 | 10.4 | 10.1 | 10.1 | 10.1 | 10.1 | 8.0 | 7.1 | 9.1 | 8.7 | 8.8 | 8.5 | 8.7 | 8.7 | 11.5 | 11.5 | 11.5 | 11.5 | 11.5 | 11.5 | 11.8 | 11.1 |
| 130 | *T. leporosum_*2 | 11.1 | 8.7 | 9.4 | 9.0 | 9.0 | 8.7 | 9.4 | 8.7 | 8.7 | 8.3 | 8.4 | 10.1 | 9.7 | 10.8 | 11.1 | 11.1 | 10.1 | 10.1 | 10.1 | 10.1 | 10.1 | 10.1 | 10.1 | 10.1 | 10.1 | 10.1 | 10.1 | 10.1 | 10.1 | 10.4 | 10.1 | 10.1 | 10.1 | 10.1 | 8.0 | 7.1 | 9.1 | 8.7 | 8.8 | 8.5 | 8.7 | 8.7 | 11.5 | 11.5 | 11.5 | 11.5 | 11.5 | 11.5 | 11.8 | 11.1 |
| 131 | *T. leporosum_*3 | 11.1 | 8.7 | 9.4 | 9.0 | 9.0 | 8.7 | 9.4 | 8.7 | 8.7 | 8.3 | 8.4 | 10.1 | 9.7 | 10.8 | 11.1 | 11.1 | 10.1 | 10.1 | 10.1 | 10.1 | 10.1 | 10.1 | 10.1 | 10.1 | 10.1 | 10.1 | 10.1 | 10.1 | 10.1 | 10.4 | 10.1 | 10.1 | 10.1 | 10.1 | 8.0 | 7.1 | 9.1 | 8.7 | 8.8 | 8.5 | 8.7 | 8.7 | 11.5 | 11.5 | 11.5 | 11.5 | 11.5 | 11.5 | 11.8 | 11.1 |
| 132 | *T. licin_*1 | 10.8 | 12.5 | 12.8 | 12.5 | 12.5 | 12.5 | 13.2 | 12.5 | 12.5 | 11.8 | 11.9 | 14.6 | 14.2 | 15.6 | 15.3 | 15.3 | 14.6 | 14.6 | 14.6 | 14.6 | 14.6 | 14.6 | 14.6 | 14.6 | 14.6 | 14.6 | 14.6 | 14.6 | 14.6 | 14.9 | 14.6 | 14.6 | 14.6 | 14.6 | 12.8 | 13.1 | 13.6 | 13.3 | 13.0 | 13.4 | 13.3 | 13.3 | 12.8 | 12.8 | 12.8 | 12.8 | 12.8 | 12.8 | 13.2 | 12.8 |
| 133 | *T. licin_*2 | 11.1 | 12.8 | 13.2 | 12.8 | 12.8 | 12.8 | 13.5 | 12.8 | 12.8 | 12.8 | 12.9 | 16.0 | 15.6 | 17.0 | 16.7 | 16.7 | 16.0 | 16.0 | 16.0 | 16.0 | 16.0 | 16.0 | 16.0 | 16.0 | 16.0 | 16.0 | 16.0 | 16.0 | 16.0 | 16.3 | 16.0 | 16.0 | 16.0 | 16.0 | 13.5 | 13.8 | 14.7 | 14.3 | 14.0 | 14.4 | 14.3 | 14.3 | 13.2 | 13.2 | 13.2 | 13.2 | 13.2 | 13.2 | 13.5 | 13.2 |
| 134 | *T. licin_*3 | 11.8 | 12.2 | 13.2 | 12.8 | 12.8 | 12.2 | 12.8 | 12.2 | 12.2 | 12.2 | 12.2 | 15.6 | 15.3 | 15.6 | 16.3 | 16.3 | 15.6 | 15.6 | 15.6 | 15.6 | 15.6 | 15.6 | 15.6 | 15.6 | 15.6 | 15.6 | 15.6 | 15.6 | 15.6 | 16.0 | 15.6 | 15.6 | 15.6 | 15.6 | 13.5 | 13.8 | 14.7 | 14.3 | 14.0 | 14.4 | 14.3 | 14.3 | 12.8 | 12.8 | 12.8 | 12.8 | 12.8 | 12.8 | 13.2 | 12.8 |
| 135 | *T. moloch_*1 | 12.2 | 9.4 | 9.4 | 9.7 | 9.7 | 9.4 | 10.1 | 9.4 | 9.4 | 9.0 | 9.1 | 10.8 | 10.4 | 11.5 | 11.8 | 11.8 | 10.8 | 10.8 | 10.8 | 10.8 | 10.8 | 10.8 | 10.8 | 10.8 | 10.8 | 10.8 | 10.8 | 10.8 | 10.8 | 11.1 | 10.8 | 10.8 | 10.8 | 10.8 | 9.4 | 8.9 | 10.1 | 9.8 | 9.8 | 9.9 | 9.8 | 9.8 | 9.4 | 9.4 | 9.4 | 9.4 | 9.4 | 9.4 | 9.7 | 9.0 |
| 136 | *T. moloch_*2 | 12.2 | 9.4 | 9.4 | 9.7 | 9.7 | 9.4 | 10.1 | 9.4 | 9.4 | 9.0 | 9.1 | 10.8 | 10.4 | 11.5 | 11.8 | 11.8 | 10.8 | 10.8 | 10.8 | 10.8 | 10.8 | 10.8 | 10.8 | 10.8 | 10.8 | 10.8 | 10.8 | 10.8 | 10.8 | 11.1 | 10.8 | 10.8 | 10.8 | 10.8 | 9.4 | 8.9 | 10.1 | 9.8 | 9.8 | 9.9 | 9.8 | 9.8 | 9.4 | 9.4 | 9.4 | 9.4 | 9.4 | 9.4 | 9.7 | 9.0 |
| 137 | *T. moloch_*4 | 7.9 | 6.5 | 6.5 | 7.0 | 7.0 | 6.5 | 7.4 | 6.5 | 6.5 | 6.5 | 6.6 | 7.0 | 7.0 | 7.4 | 7.9 | 7.9 | 7.0 | 7.0 | 7.0 | 7.0 | 7.0 | 7.0 | 7.0 | 7.0 | 7.0 | 7.0 | 7.0 | 7.0 | 7.0 | 7.4 | 7.0 | 7.0 | 7.0 | 7.0 | 7.0 | 6.7 | 7.9 | 7.4 | 7.5 | 7.4 | 7.0 | 7.0 | 6.0 | 6.0 | 6.0 | 6.0 | 6.0 | 6.0 | 6.5 | 6.0 |
| 138 | *T. nebulosum_*1 | 15.0 | 12.5 | 12.2 | 12.5 | 12.5 | 12.5 | 12.5 | 12.5 | 12.5 | 12.2 | 12.3 | 13.2 | 12.9 | 13.9 | 13.9 | 13.9 | 13.2 | 13.2 | 13.2 | 13.2 | 13.2 | 13.2 | 13.2 | 13.2 | 13.2 | 13.2 | 13.2 | 13.2 | 13.2 | 13.6 | 13.2 | 13.2 | 13.2 | 13.2 | 12.5 | 12.8 | 12.6 | 12.6 | 12.3 | 13.1 | 12.6 | 12.6 | 15.3 | 15.3 | 15.3 | 15.3 | 15.3 | 15.3 | 15.0 | 15.0 |
| 139 | *T. nebulosum_*2 | 12.2 | 9.7 | 9.4 | 9.7 | 9.7 | 9.7 | 9.7 | 9.7 | 9.7 | 9.4 | 9.4 | 10.4 | 10.1 | 11.1 | 11.1 | 11.1 | 10.4 | 10.4 | 10.4 | 10.4 | 10.4 | 10.4 | 10.4 | 10.4 | 10.4 | 10.4 | 10.4 | 10.4 | 10.4 | 10.8 | 10.4 | 10.4 | 10.4 | 10.4 | 9.7 | 10.3 | 10.1 | 10.1 | 9.8 | 10.6 | 10.1 | 10.1 | 12.2 | 12.2 | 12.2 | 12.2 | 12.2 | 12.2 | 12.5 | 11.8 |
| 140 | *T. nebulosum_*3 | 12.2 | 9.7 | 9.4 | 9.7 | 9.7 | 9.7 | 9.7 | 9.7 | 9.7 | 9.4 | 9.4 | 10.4 | 10.1 | 11.1 | 11.1 | 11.1 | 10.4 | 10.4 | 10.4 | 10.4 | 10.4 | 10.4 | 10.4 | 10.4 | 10.4 | 10.4 | 10.4 | 10.4 | 10.4 | 10.8 | 10.4 | 10.4 | 10.4 | 10.4 | 9.7 | 10.3 | 10.1 | 10.1 | 9.8 | 10.6 | 10.1 | 10.1 | 12.2 | 12.2 | 12.2 | 12.2 | 12.2 | 12.2 | 12.5 | 11.8 |
| 141 | *T. nebulosum_*4 | 11.8 | 9.8 | 9.4 | 9.8 | 9.8 | 9.8 | 9.8 | 9.8 | 9.8 | 9.4 | 9.5 | 10.5 | 10.1 | 11.1 | 11.1 | 11.1 | 10.5 | 10.5 | 10.5 | 10.5 | 10.5 | 10.5 | 10.5 | 10.5 | 10.5 | 10.5 | 10.5 | 10.5 | 10.5 | 10.8 | 10.5 | 10.5 | 10.5 | 10.5 | 9.4 | 10.3 | 10.2 | 10.2 | 9.9 | 10.6 | 10.2 | 10.2 | 11.8 | 11.8 | 11.8 | 11.8 | 11.8 | 11.8 | 12.2 | 11.8 |
| 142 | *T. palliatum_*1 | 13.9 | 7.3 | 7.3 | 7.6 | 7.6 | 7.3 | 8.0 | 7.3 | 7.3 | 7.6 | 7.7 | 9.7 | 9.4 | 10.4 | 10.4 | 10.4 | 9.7 | 9.7 | 9.7 | 9.7 | 9.7 | 9.7 | 9.7 | 9.7 | 9.7 | 9.7 | 9.7 | 9.7 | 9.7 | 10.1 | 9.7 | 9.7 | 9.7 | 9.7 | 11.8 | 12.1 | 11.2 | 11.2 | 11.2 | 11.6 | 11.2 | 11.2 | 9.7 | 9.7 | 9.4 | 9.4 | 9.7 | 9.7 | 10.1 | 9.7 |
| 143 | *T. palliatum_*2 | 14.2 | 7.6 | 7.6 | 8.0 | 8.0 | 7.6 | 8.3 | 7.6 | 7.6 | 8.0 | 8.0 | 10.1 | 9.7 | 10.8 | 10.8 | 10.8 | 10.1 | 10.1 | 10.1 | 10.1 | 10.1 | 10.1 | 10.1 | 10.1 | 10.1 | 10.1 | 10.1 | 10.1 | 10.1 | 10.4 | 10.1 | 10.1 | 10.1 | 10.1 | 12.2 | 12.4 | 11.5 | 11.5 | 11.6 | 12.0 | 11.5 | 11.5 | 10.1 | 10.1 | 9.7 | 9.7 | 10.1 | 10.1 | 10.4 | 10.1 |
| 144 | *T. palliatum_*3 | 13.5 | 6.9 | 6.9 | 7.3 | 7.3 | 6.9 | 7.6 | 6.9 | 6.9 | 7.3 | 7.3 | 9.4 | 9.0 | 10.1 | 10.1 | 10.1 | 9.4 | 9.4 | 9.4 | 9.4 | 9.4 | 9.4 | 9.4 | 9.4 | 9.4 | 9.4 | 9.4 | 9.4 | 9.4 | 9.7 | 9.4 | 9.4 | 9.4 | 9.4 | 11.5 | 11.7 | 10.8 | 10.8 | 10.9 | 11.3 | 10.8 | 10.8 | 9.4 | 9.4 | 9.0 | 9.0 | 9.4 | 9.4 | 9.7 | 9.4 |
| 145 | *T. palliatum_*4 | 13.9 | 8.0 | 8.0 | 8.3 | 8.3 | 8.0 | 8.7 | 8.0 | 8.0 | 8.3 | 8.4 | 10.4 | 10.1 | 11.1 | 11.1 | 11.1 | 10.4 | 10.4 | 10.4 | 10.4 | 10.4 | 10.4 | 10.4 | 10.4 | 10.4 | 10.4 | 10.4 | 10.4 | 10.4 | 10.8 | 10.4 | 10.4 | 10.4 | 10.4 | 11.8 | 12.1 | 11.2 | 11.2 | 11.2 | 11.6 | 11.2 | 11.2 | 10.4 | 10.4 | 10.1 | 10.1 | 10.4 | 10.4 | 10.8 | 10.4 |
| 146 | *T. palliatum_*5 | 13.5 | 6.9 | 6.9 | 7.3 | 7.3 | 6.9 | 7.6 | 6.9 | 6.9 | 7.3 | 7.3 | 9.4 | 9.0 | 10.1 | 10.1 | 10.1 | 9.4 | 9.4 | 9.4 | 9.4 | 9.4 | 9.4 | 9.4 | 9.4 | 9.4 | 9.4 | 9.4 | 9.4 | 9.4 | 9.7 | 9.4 | 9.4 | 9.4 | 9.4 | 11.5 | 11.7 | 10.8 | 10.8 | 10.9 | 11.3 | 10.8 | 10.8 | 9.4 | 9.4 | 9.0 | 9.0 | 9.4 | 9.4 | 9.7 | 9.4 |
| 147 | *T. palliatum_*6 | 13.2 | 7.3 | 7.3 | 7.6 | 7.6 | 7.3 | 8.0 | 7.3 | 7.3 | 7.6 | 7.7 | 9.7 | 9.4 | 10.4 | 10.4 | 10.4 | 9.7 | 9.7 | 9.7 | 9.7 | 9.7 | 9.7 | 9.7 | 9.7 | 9.7 | 9.7 | 9.7 | 9.7 | 9.7 | 10.1 | 9.7 | 9.7 | 9.7 | 9.7 | 11.1 | 11.3 | 10.5 | 10.5 | 10.5 | 10.9 | 10.5 | 10.5 | 9.7 | 9.7 | 9.4 | 9.4 | 9.7 | 9.7 | 10.1 | 9.7 |
| 148 | *T. palliatum_*7 | 13.9 | 7.5 | 7.9 | 7.9 | 7.9 | 7.5 | 8.3 | 7.5 | 7.5 | 7.9 | 8.0 | 11.1 | 10.7 | 11.9 | 11.9 | 11.9 | 11.1 | 11.1 | 11.1 | 11.1 | 11.1 | 11.1 | 11.1 | 11.1 | 11.1 | 11.1 | 11.1 | 11.1 | 11.1 | 11.5 | 11.1 | 11.1 | 11.1 | 11.1 | 12.3 | 12.4 | 11.6 | 11.6 | 11.6 | 12.1 | 11.6 | 11.6 | 10.7 | 10.7 | 10.3 | 10.3 | 10.7 | 10.7 | 10.7 | 10.7 |
| 149 | *T. palliatum_*8 | 13.5 | 7.6 | 7.6 | 8.0 | 8.0 | 7.6 | 8.3 | 7.6 | 7.6 | 8.0 | 8.0 | 10.1 | 9.7 | 10.8 | 10.8 | 10.8 | 10.1 | 10.1 | 10.1 | 10.1 | 10.1 | 10.1 | 10.1 | 10.1 | 10.1 | 10.1 | 10.1 | 10.1 | 10.1 | 10.4 | 10.1 | 10.1 | 10.1 | 10.1 | 11.5 | 11.7 | 10.8 | 10.8 | 10.9 | 11.3 | 10.8 | 10.8 | 10.1 | 10.1 | 9.7 | 9.7 | 10.1 | 10.1 | 10.4 | 10.1 |
| 150 | *T. palliatum_*9 | 13.9 | 7.3 | 7.3 | 7.6 | 7.6 | 7.3 | 8.0 | 7.3 | 7.3 | 7.6 | 7.7 | 9.7 | 9.4 | 10.4 | 10.4 | 10.4 | 9.7 | 9.7 | 9.7 | 9.7 | 9.7 | 9.7 | 9.7 | 9.7 | 9.7 | 9.7 | 9.7 | 9.7 | 9.7 | 10.1 | 9.7 | 9.7 | 9.7 | 9.7 | 11.8 | 12.1 | 11.2 | 11.2 | 11.2 | 11.6 | 11.2 | 11.2 | 9.7 | 9.7 | 9.4 | 9.4 | 9.7 | 9.7 | 10.1 | 9.7 |
| 151 | *T. palliatum_*10 | 13.9 | 7.3 | 7.3 | 7.6 | 7.6 | 7.3 | 8.0 | 7.3 | 7.3 | 7.6 | 7.7 | 9.7 | 9.4 | 10.4 | 10.4 | 10.4 | 9.7 | 9.7 | 9.7 | 9.7 | 9.7 | 9.7 | 9.7 | 9.7 | 9.7 | 9.7 | 9.7 | 9.7 | 9.7 | 10.1 | 9.7 | 9.7 | 9.7 | 9.7 | 11.8 | 12.1 | 11.2 | 11.2 | 11.2 | 11.6 | 11.2 | 11.2 | 9.7 | 9.7 | 9.4 | 9.4 | 9.7 | 9.7 | 10.1 | 9.7 |
| 152 | *T. palliatum_*11 | 13.9 | 7.3 | 7.3 | 7.6 | 7.6 | 7.3 | 8.0 | 7.3 | 7.3 | 7.6 | 7.7 | 9.7 | 9.4 | 10.4 | 10.4 | 10.4 | 9.7 | 9.7 | 9.7 | 9.7 | 9.7 | 9.7 | 9.7 | 9.7 | 9.7 | 9.7 | 9.7 | 9.7 | 9.7 | 10.1 | 9.7 | 9.7 | 9.7 | 9.7 | 11.8 | 12.1 | 11.2 | 11.2 | 11.2 | 11.6 | 11.2 | 11.2 | 9.7 | 9.7 | 9.4 | 9.4 | 9.7 | 9.7 | 10.1 | 9.7 |
| 153 | *T. petilum_*11 | 9.4 | 11.5 | 11.5 | 11.8 | 11.8 | 11.5 | 12.2 | 11.5 | 11.5 | 11.8 | 11.9 | 16.0 | 15.6 | 16.3 | 16.7 | 16.7 | 16.0 | 16.0 | 16.0 | 16.0 | 16.0 | 16.0 | 16.0 | 16.0 | 16.0 | 16.0 | 16.0 | 16.0 | 16.0 | 16.3 | 16.0 | 16.0 | 16.0 | 16.0 | 12.2 | 13.1 | 13.6 | 13.3 | 13.3 | 13.4 | 12.6 | 12.6 | 11.8 | 11.8 | 11.5 | 11.5 | 11.8 | 11.8 | 12.2 | 11.5 |
| 154 | *T. phrynoderma_*1 | 12.2 | 13.8 | 14.2 | 14.2 | 14.2 | 13.8 | 14.6 | 13.8 | 13.8 | 13.4 | 13.5 | 13.4 | 13.0 | 14.2 | 15.0 | 15.0 | 13.4 | 13.4 | 13.4 | 13.4 | 13.4 | 13.4 | 13.4 | 13.4 | 13.4 | 13.4 | 13.4 | 13.4 | 13.4 | 13.8 | 13.4 | 13.4 | 13.4 | 13.4 | 13.0 | 13.4 | 14.2 | 13.8 | 13.5 | 13.8 | 13.8 | 13.8 | 15.7 | 15.7 | 15.7 | 15.7 | 15.7 | 15.7 | 16.1 | 15.7 |
| 155 | *T. phrynoderma_*2 | 12.2 | 12.6 | 13.0 | 13.0 | 13.0 | 12.6 | 13.4 | 12.6 | 12.6 | 12.2 | 12.3 | 13.0 | 12.6 | 13.8 | 14.6 | 14.6 | 13.0 | 13.0 | 13.0 | 13.0 | 13.0 | 13.0 | 13.0 | 13.0 | 13.0 | 13.0 | 13.0 | 13.0 | 13.0 | 13.4 | 13.0 | 13.0 | 13.0 | 13.0 | 12.2 | 12.6 | 13.4 | 13.0 | 12.7 | 13.0 | 13.0 | 13.0 | 15.0 | 15.0 | 15.0 | 15.0 | 15.0 | 15.0 | 15.4 | 15.0 |
| 156 | *T. pyaukkya_*1 | 5.6 | 11.5 | 11.5 | 11.8 | 11.8 | 11.5 | 12.2 | 11.5 | 11.5 | 11.1 | 11.2 | 13.5 | 13.2 | 14.2 | 14.6 | 14.6 | 13.5 | 13.5 | 13.5 | 13.5 | 13.5 | 13.5 | 13.5 | 13.5 | 13.5 | 13.5 | 13.5 | 13.5 | 13.5 | 13.9 | 13.5 | 13.5 | 13.5 | 13.5 | 11.1 | 11.0 | 11.5 | 11.2 | 11.2 | 10.9 | 11.2 | 11.2 | 11.5 | 11.5 | 11.1 | 11.1 | 11.5 | 11.5 | 11.8 | 11.1 |
| 157 | *T. pyaukkya_*2 | 5.6 | 11.1 | 11.1 | 11.5 | 11.5 | 11.1 | 11.8 | 11.1 | 11.1 | 10.8 | 10.8 | 13.5 | 13.2 | 14.2 | 14.6 | 14.6 | 13.5 | 13.5 | 13.5 | 13.5 | 13.5 | 13.5 | 13.5 | 13.5 | 13.5 | 13.5 | 13.5 | 13.5 | 13.5 | 13.9 | 13.5 | 13.5 | 13.5 | 13.5 | 10.8 | 10.6 | 11.2 | 10.8 | 10.9 | 10.6 | 10.8 | 10.8 | 11.1 | 11.1 | 10.8 | 10.8 | 11.1 | 11.1 | 11.5 | 10.8 |
| 158 | *T. pyaukkya_*3 | 5.6 | 10.4 | 10.4 | 10.8 | 10.8 | 10.4 | 11.1 | 10.4 | 10.4 | 10.8 | 10.8 | 13.5 | 13.2 | 14.2 | 14.6 | 14.6 | 13.5 | 13.5 | 13.5 | 13.5 | 13.5 | 13.5 | 13.5 | 13.5 | 13.5 | 13.5 | 13.5 | 13.5 | 13.5 | 13.9 | 13.5 | 13.5 | 13.5 | 13.5 | 10.8 | 10.6 | 11.2 | 10.8 | 10.9 | 10.9 | 10.8 | 10.8 | 10.4 | 10.4 | 10.1 | 10.1 | 10.4 | 10.4 | 10.8 | 10.1 |
| 159 | *T. pyaukkya_*4 | 3.1 | 11.1 | 10.8 | 11.1 | 11.1 | 11.1 | 11.8 | 11.1 | 11.1 | 11.5 | 11.5 | 13.5 | 13.2 | 14.2 | 14.2 | 14.2 | 13.5 | 13.5 | 13.5 | 13.5 | 13.5 | 13.5 | 13.5 | 13.5 | 13.5 | 13.5 | 13.5 | 13.5 | 13.5 | 13.9 | 13.5 | 13.5 | 13.5 | 13.5 | 10.1 | 10.3 | 10.5 | 10.1 | 10.2 | 10.2 | 10.1 | 10.1 | 10.1 | 10.1 | 10.1 | 10.1 | 10.1 | 10.1 | 10.4 | 10.1 |
| 160 | *T. pyaukkya_*5 | 3.1 | 11.1 | 10.8 | 11.1 | 11.1 | 11.1 | 11.8 | 11.1 | 11.1 | 11.5 | 11.5 | 13.5 | 13.2 | 14.2 | 14.2 | 14.2 | 13.5 | 13.5 | 13.5 | 13.5 | 13.5 | 13.5 | 13.5 | 13.5 | 13.5 | 13.5 | 13.5 | 13.5 | 13.5 | 13.9 | 13.5 | 13.5 | 13.5 | 13.5 | 10.1 | 10.3 | 10.5 | 10.1 | 10.2 | 10.2 | 10.1 | 10.1 | 10.1 | 10.1 | 10.1 | 10.1 | 10.1 | 10.1 | 10.4 | 10.1 |
| 161 | *T. rhododiscus_*1 | 12.2 | 8.3 | 8.3 | 8.7 | 8.7 | 8.3 | 9.0 | 8.3 | 8.3 | 8.7 | 8.7 | 12.5 | 12.2 | 12.8 | 13.2 | 13.2 | 12.5 | 12.5 | 12.5 | 12.5 | 12.5 | 12.5 | 12.5 | 12.5 | 12.5 | 12.5 | 12.5 | 12.5 | 12.5 | 12.8 | 12.5 | 12.5 | 12.5 | 12.5 | 9.4 | 10.6 | 10.1 | 10.1 | 10.2 | 9.5 | 10.1 | 10.1 | 2.4 | 2.4 | 2.8 | 2.8 | 2.4 | 2.4 | 2.8 | 3.1 |
| 162 | *T. rhododiscus*10 | 11.8 | 8.3 | 8.3 | 8.7 | 8.7 | 8.3 | 9.0 | 8.3 | 8.3 | 8.7 | 8.7 | 12.2 | 11.8 | 12.5 | 12.8 | 12.8 | 12.2 | 12.2 | 12.2 | 12.2 | 12.2 | 12.2 | 12.2 | 12.2 | 12.2 | 12.2 | 12.2 | 12.2 | 12.2 | 12.5 | 12.2 | 12.2 | 12.2 | 12.2 | 9.4 | 9.9 | 10.1 | 10.1 | 10.2 | 9.5 | 10.1 | 10.1 | 3.1 | 3.1 | 3.1 | 3.1 | 3.1 | 3.1 | 3.5 | 3.5 |
| 163 | *T. rhododiscus_*2 | 12.2 | 8.3 | 8.3 | 8.7 | 8.7 | 8.3 | 9.0 | 8.3 | 8.3 | 8.7 | 8.7 | 12.5 | 12.2 | 12.8 | 13.2 | 13.2 | 12.5 | 12.5 | 12.5 | 12.5 | 12.5 | 12.5 | 12.5 | 12.5 | 12.5 | 12.5 | 12.5 | 12.5 | 12.5 | 12.8 | 12.5 | 12.5 | 12.5 | 12.5 | 9.4 | 10.6 | 10.1 | 10.1 | 10.2 | 9.5 | 10.1 | 10.1 | 2.4 | 2.4 | 2.8 | 2.8 | 2.4 | 2.4 | 2.8 | 3.1 |
| 164 | *T. rhododiscu_s*3 | 12.2 | 8.3 | 8.3 | 8.7 | 8.7 | 8.3 | 9.0 | 8.3 | 8.3 | 8.7 | 8.7 | 12.5 | 12.2 | 12.8 | 13.2 | 13.2 | 12.5 | 12.5 | 12.5 | 12.5 | 12.5 | 12.5 | 12.5 | 12.5 | 12.5 | 12.5 | 12.5 | 12.5 | 12.5 | 12.8 | 12.5 | 12.5 | 12.5 | 12.5 | 9.4 | 10.6 | 10.1 | 10.1 | 10.2 | 9.5 | 10.1 | 10.1 | 2.4 | 2.4 | 2.8 | 2.8 | 2.4 | 2.4 | 2.8 | 3.1 |
| 165 | *T. rhododiscus_*4 | 12.2 | 8.3 | 8.3 | 8.7 | 8.7 | 8.3 | 9.0 | 8.3 | 8.3 | 8.7 | 8.7 | 12.5 | 12.2 | 12.8 | 13.2 | 13.2 | 12.5 | 12.5 | 12.5 | 12.5 | 12.5 | 12.5 | 12.5 | 12.5 | 12.5 | 12.5 | 12.5 | 12.5 | 12.5 | 12.8 | 12.5 | 12.5 | 12.5 | 12.5 | 9.4 | 10.6 | 10.1 | 10.1 | 10.2 | 9.5 | 10.1 | 10.1 | 2.4 | 2.4 | 2.8 | 2.8 | 2.4 | 2.4 | 2.8 | 3.1 |
| 166 | *T. rhododiscus_*5 | 12.2 | 8.3 | 8.3 | 8.7 | 8.7 | 8.3 | 9.0 | 8.3 | 8.3 | 8.7 | 8.7 | 12.5 | 12.2 | 12.8 | 13.2 | 13.2 | 12.5 | 12.5 | 12.5 | 12.5 | 12.5 | 12.5 | 12.5 | 12.5 | 12.5 | 12.5 | 12.5 | 12.5 | 12.5 | 12.8 | 12.5 | 12.5 | 12.5 | 12.5 | 9.4 | 10.6 | 10.1 | 10.1 | 10.2 | 9.5 | 10.1 | 10.1 | 2.4 | 2.4 | 2.8 | 2.8 | 2.4 | 2.4 | 2.8 | 3.1 |
| 167 | *T. rhododiscus_*6 | 12.2 | 8.3 | 8.3 | 8.7 | 8.7 | 8.3 | 9.0 | 8.3 | 8.3 | 8.7 | 8.7 | 12.5 | 12.2 | 12.8 | 13.2 | 13.2 | 12.5 | 12.5 | 12.5 | 12.5 | 12.5 | 12.5 | 12.5 | 12.5 | 12.5 | 12.5 | 12.5 | 12.5 | 12.5 | 12.8 | 12.5 | 12.5 | 12.5 | 12.5 | 9.4 | 10.6 | 10.1 | 10.1 | 10.2 | 9.5 | 10.1 | 10.1 | 2.4 | 2.4 | 2.8 | 2.8 | 2.4 | 2.4 | 2.8 | 3.1 |
| 168 | *T. rhododiscus_*7 | 12.2 | 8.3 | 8.3 | 8.7 | 8.7 | 8.3 | 9.0 | 8.3 | 8.3 | 8.7 | 8.7 | 12.5 | 12.2 | 12.8 | 13.2 | 13.2 | 12.5 | 12.5 | 12.5 | 12.5 | 12.5 | 12.5 | 12.5 | 12.5 | 12.5 | 12.5 | 12.5 | 12.5 | 12.5 | 12.8 | 12.5 | 12.5 | 12.5 | 12.5 | 9.4 | 10.6 | 10.1 | 10.1 | 10.2 | 9.5 | 10.1 | 10.1 | 2.4 | 2.4 | 2.8 | 2.8 | 2.4 | 2.4 | 2.8 | 3.1 |
| 169 | *T. rhododiscus_*8 | 12.2 | 8.3 | 8.3 | 8.7 | 8.7 | 8.3 | 9.0 | 8.3 | 8.3 | 8.7 | 8.7 | 12.5 | 12.2 | 12.8 | 13.2 | 13.2 | 12.5 | 12.5 | 12.5 | 12.5 | 12.5 | 12.5 | 12.5 | 12.5 | 12.5 | 12.5 | 12.5 | 12.5 | 12.5 | 12.8 | 12.5 | 12.5 | 12.5 | 12.5 | 9.4 | 10.6 | 10.1 | 10.1 | 10.2 | 9.5 | 10.1 | 10.1 | 2.4 | 2.4 | 2.8 | 2.8 | 2.4 | 2.4 | 2.8 | 3.1 |
| 170 | *T. rhododiscus_*9 | 11.5 | 8.0 | 8.0 | 8.3 | 8.3 | 8.0 | 8.7 | 8.0 | 8.0 | 8.3 | 8.4 | 12.5 | 12.2 | 12.8 | 13.2 | 13.2 | 12.5 | 12.5 | 12.5 | 12.5 | 12.5 | 12.5 | 12.5 | 12.5 | 12.5 | 12.5 | 12.5 | 12.5 | 12.5 | 12.8 | 12.5 | 12.5 | 12.5 | 12.5 | 9.7 | 10.3 | 10.5 | 10.5 | 10.5 | 9.5 | 10.5 | 10.5 | 3.1 | 3.1 | 2.8 | 2.8 | 3.1 | 3.1 | 3.5 | 3.1 |
| 171 | *T. ryabovi_*1 | 12.8 | 11.8 | 11.8 | 12.2 | 12.2 | 11.8 | 12.5 | 11.8 | 11.8 | 11.5 | 11.5 | 11.5 | 11.1 | 11.8 | 12.5 | 12.5 | 11.5 | 11.5 | 11.5 | 11.5 | 11.5 | 11.5 | 11.5 | 11.5 | 11.5 | 11.5 | 11.5 | 11.5 | 11.5 | 11.8 | 11.5 | 11.5 | 11.5 | 11.5 | 12.2 | 11.7 | 12.2 | 11.9 | 11.9 | 12.0 | 11.9 | 11.9 | 11.5 | 11.5 | 11.5 | 11.5 | 11.5 | 11.5 | 11.8 | 11.1 |
| 172 | *T. ryabovi_*2 | 15.3 | 14.9 | 14.9 | 15.3 | 15.3 | 14.9 | 15.6 | 14.9 | 14.9 | 14.6 | 14.7 | 14.6 | 14.2 | 14.9 | 15.6 | 15.6 | 14.6 | 14.6 | 14.6 | 14.6 | 14.6 | 14.6 | 14.6 | 14.6 | 14.6 | 14.6 | 14.6 | 14.6 | 14.6 | 14.9 | 14.6 | 14.6 | 14.6 | 14.6 | 14.6 | 13.8 | 14.7 | 14.3 | 14.4 | 14.4 | 14.3 | 14.3 | 14.6 | 14.6 | 14.6 | 14.6 | 14.6 | 14.6 | 14.9 | 14.2 |
| 173 | *T. ryabovi_*3 | 15.3 | 14.9 | 14.9 | 15.3 | 15.3 | 14.9 | 15.6 | 14.9 | 14.9 | 14.6 | 14.7 | 14.6 | 14.2 | 14.9 | 15.6 | 15.6 | 14.6 | 14.6 | 14.6 | 14.6 | 14.6 | 14.6 | 14.6 | 14.6 | 14.6 | 14.6 | 14.6 | 14.6 | 14.6 | 14.9 | 14.6 | 14.6 | 14.6 | 14.6 | 14.6 | 13.8 | 14.7 | 14.3 | 14.4 | 14.4 | 14.3 | 14.3 | 14.6 | 14.6 | 14.6 | 14.6 | 14.6 | 14.6 | 14.9 | 14.2 |
| 174 | *T. stellatum_*1 | 19.1 | 13.9 | 13.5 | 13.9 | 13.9 | 13.9 | 14.6 | 13.9 | 13.9 | 13.9 | 14.0 | 17.4 | 17.0 | 17.4 | 18.1 | 18.1 | 17.4 | 17.4 | 17.4 | 17.4 | 17.4 | 17.4 | 17.4 | 17.4 | 17.4 | 17.4 | 17.4 | 17.4 | 17.4 | 17.7 | 17.4 | 17.4 | 17.4 | 17.4 | 17.0 | 18.1 | 17.5 | 17.1 | 17.2 | 17.6 | 17.1 | 17.1 | 16.0 | 16.0 | 15.6 | 15.6 | 16.0 | 16.0 | 16.3 | 15.3 |
| 175 | *T. stellatum_*2 | 19.1 | 13.9 | 13.5 | 13.9 | 13.9 | 13.9 | 14.6 | 13.9 | 13.9 | 13.9 | 14.0 | 17.4 | 17.0 | 17.4 | 18.1 | 18.1 | 17.4 | 17.4 | 17.4 | 17.4 | 17.4 | 17.4 | 17.4 | 17.4 | 17.4 | 17.4 | 17.4 | 17.4 | 17.4 | 17.7 | 17.4 | 17.4 | 17.4 | 17.4 | 17.0 | 18.1 | 17.5 | 17.1 | 17.2 | 17.6 | 17.1 | 17.1 | 16.0 | 16.0 | 15.6 | 15.6 | 16.0 | 16.0 | 16.3 | 15.3 |
| 176 | *T. truongsonense_*1 | 13.9 | 11.8 | 11.8 | 12.2 | 12.2 | 11.8 | 11.8 | 11.8 | 11.8 | 12.2 | 12.2 | 14.2 | 13.9 | 13.9 | 14.2 | 14.2 | 13.9 | 13.9 | 13.9 | 13.9 | 13.9 | 13.9 | 13.9 | 13.9 | 13.9 | 13.9 | 13.9 | 13.9 | 13.9 | 14.2 | 13.9 | 13.9 | 13.9 | 13.9 | 12.5 | 14.5 | 15.0 | 14.7 | 14.4 | 15.1 | 15.4 | 15.4 | 13.2 | 13.2 | 12.8 | 12.8 | 13.2 | 13.2 | 13.5 | 13.2 |
| 177 | *T. truongsonense_*2 | 14.6 | 11.5 | 11.5 | 11.8 | 11.8 | 11.5 | 11.5 | 11.5 | 11.5 | 11.8 | 11.9 | 13.2 | 12.8 | 12.8 | 13.2 | 13.2 | 12.8 | 12.8 | 12.8 | 12.8 | 12.8 | 12.8 | 12.8 | 12.8 | 12.8 | 12.8 | 12.8 | 12.8 | 12.8 | 13.2 | 12.8 | 12.8 | 12.8 | 12.8 | 13.5 | 14.9 | 15.4 | 15.0 | 15.1 | 15.5 | 15.7 | 15.7 | 12.8 | 12.8 | 12.5 | 12.5 | 12.8 | 12.8 | 13.2 | 12.8 |
| 178 | *T. truongsonense_*3 | 14.6 | 11.5 | 11.5 | 11.8 | 11.8 | 11.5 | 11.5 | 11.5 | 11.5 | 11.8 | 11.9 | 13.2 | 12.8 | 12.8 | 13.2 | 13.2 | 12.8 | 12.8 | 12.8 | 12.8 | 12.8 | 12.8 | 12.8 | 12.8 | 12.8 | 12.8 | 12.8 | 12.8 | 12.8 | 13.2 | 12.8 | 12.8 | 12.8 | 12.8 | 13.5 | 14.9 | 15.4 | 15.0 | 15.1 | 15.5 | 15.7 | 15.7 | 12.8 | 12.8 | 12.5 | 12.5 | 12.8 | 12.8 | 13.2 | 12.8 |
| 179 | *T. truongsonense_*4 | 14.6 | 11.5 | 11.5 | 11.8 | 11.8 | 11.5 | 11.5 | 11.5 | 11.5 | 11.8 | 11.9 | 13.5 | 13.2 | 12.2 | 13.5 | 13.5 | 13.2 | 13.2 | 13.2 | 13.2 | 13.2 | 13.2 | 13.2 | 13.2 | 13.2 | 13.2 | 13.2 | 13.2 | 13.2 | 13.5 | 13.2 | 13.2 | 13.2 | 13.2 | 12.8 | 14.2 | 14.3 | 14.0 | 13.7 | 14.4 | 14.7 | 14.7 | 14.2 | 14.2 | 13.9 | 13.9 | 14.2 | 14.2 | 14.6 | 13.5 |
| 180 | *T. truongsonense_*5 | 17.0 | 12.5 | 12.9 | 12.9 | 12.9 | 12.5 | 12.5 | 12.5 | 12.5 | 12.9 | 13.0 | 14.8 | 14.4 | 13.7 | 14.8 | 14.8 | 14.4 | 14.4 | 14.4 | 14.4 | 14.4 | 14.4 | 14.4 | 14.4 | 14.4 | 14.4 | 14.4 | 14.4 | 14.4 | 14.8 | 14.4 | 14.4 | 14.4 | 14.4 | 14.0 | 15.2 | 16.0 | 15.6 | 15.3 | 16.1 | 16.4 | 16.4 | 15.5 | 15.5 | 15.1 | 15.1 | 15.5 | 15.5 | 15.9 | 14.8 |
| 181 | *T. truongsonense_*6 | 14.6 | 11.1 | 11.1 | 11.5 | 11.5 | 11.1 | 11.1 | 11.1 | 11.1 | 11.5 | 11.5 | 13.9 | 13.5 | 12.5 | 13.9 | 13.9 | 13.5 | 13.5 | 13.5 | 13.5 | 13.5 | 13.5 | 13.5 | 13.5 | 13.5 | 13.5 | 13.5 | 13.5 | 13.5 | 13.9 | 13.5 | 13.5 | 13.5 | 13.5 | 12.8 | 14.2 | 14.7 | 14.3 | 14.0 | 14.8 | 15.0 | 15.0 | 13.5 | 13.5 | 13.2 | 13.2 | 13.5 | 13.5 | 13.9 | 12.8 |
| 182 | *T. truongsonense_*7 | 14.6 | 11.1 | 11.1 | 11.5 | 11.5 | 11.1 | 11.1 | 11.1 | 11.1 | 11.5 | 11.5 | 13.9 | 13.5 | 12.5 | 13.9 | 13.9 | 13.5 | 13.5 | 13.5 | 13.5 | 13.5 | 13.5 | 13.5 | 13.5 | 13.5 | 13.5 | 13.5 | 13.5 | 13.5 | 13.9 | 13.5 | 13.5 | 13.5 | 13.5 | 12.8 | 14.2 | 14.7 | 14.3 | 14.0 | 14.8 | 15.0 | 15.0 | 13.5 | 13.5 | 13.2 | 13.2 | 13.5 | 13.5 | 13.9 | 12.8 |
| 183 | *T. vietnamense_*1 | 17.4 | 11.1 | 11.1 | 11.1 | 11.1 | 11.1 | 11.8 | 11.1 | 11.1 | 11.1 | 11.2 | 14.2 | 13.9 | 14.2 | 13.9 | 13.9 | 14.2 | 14.2 | 14.2 | 14.2 | 14.2 | 14.2 | 14.2 | 14.2 | 14.2 | 14.2 | 14.2 | 14.2 | 14.2 | 14.6 | 14.2 | 14.2 | 14.2 | 14.2 | 14.9 | 14.9 | 15.7 | 15.4 | 15.4 | 15.8 | 16.1 | 16.1 | 14.2 | 14.2 | 13.9 | 13.9 | 14.2 | 14.2 | 14.6 | 13.5 |
| 184 | *T. vietnamense_*2 | 17.7 | 11.5 | 11.8 | 11.5 | 11.5 | 11.5 | 12.2 | 11.5 | 11.5 | 11.5 | 11.5 | 14.9 | 14.6 | 14.9 | 14.6 | 14.6 | 14.9 | 14.9 | 14.9 | 14.9 | 14.9 | 14.9 | 14.9 | 14.9 | 14.9 | 14.9 | 14.9 | 14.9 | 14.9 | 15.3 | 14.9 | 14.9 | 14.9 | 14.9 | 14.9 | 15.6 | 15.7 | 15.4 | 15.4 | 15.8 | 16.1 | 16.1 | 14.6 | 14.6 | 14.2 | 14.2 | 14.6 | 14.6 | 14.9 | 13.9 |
| 185 | *T. vietnamense_*3 | 19.1 | 11.8 | 12.2 | 11.8 | 11.8 | 11.8 | 12.5 | 11.8 | 11.8 | 11.1 | 11.2 | 14.6 | 14.2 | 14.6 | 14.2 | 14.2 | 14.6 | 14.6 | 14.6 | 14.6 | 14.6 | 14.6 | 14.6 | 14.6 | 14.6 | 14.6 | 14.6 | 14.6 | 14.6 | 14.9 | 14.6 | 14.6 | 14.6 | 14.6 | 16.0 | 15.2 | 16.1 | 15.7 | 15.8 | 16.2 | 16.4 | 16.4 | 15.3 | 15.3 | 14.9 | 14.9 | 15.3 | 15.3 | 15.6 | 14.6 |
| 186 | *T. vietnamense_*4 | 19.2 | 11.8 | 12.2 | 11.8 | 11.8 | 11.8 | 12.5 | 11.8 | 11.8 | 11.1 | 11.2 | 14.3 | 13.9 | 14.3 | 13.9 | 13.9 | 14.3 | 14.3 | 14.3 | 14.3 | 14.3 | 14.3 | 14.3 | 14.3 | 14.3 | 14.3 | 14.3 | 14.3 | 14.3 | 14.6 | 14.3 | 14.3 | 14.3 | 14.3 | 16.0 | 15.3 | 16.1 | 15.8 | 15.8 | 16.3 | 16.5 | 16.5 | 15.3 | 15.3 | 15.0 | 15.0 | 15.3 | 15.3 | 15.7 | 14.6 |
| 187 | *T. vietnamense_*5 | 17.7 | 11.8 | 12.2 | 11.8 | 11.8 | 11.8 | 12.5 | 11.8 | 11.8 | 11.1 | 11.2 | 14.6 | 14.2 | 14.6 | 14.2 | 14.2 | 14.6 | 14.6 | 14.6 | 14.6 | 14.6 | 14.6 | 14.6 | 14.6 | 14.6 | 14.6 | 14.6 | 14.6 | 14.6 | 14.9 | 14.6 | 14.6 | 14.6 | 14.6 | 14.6 | 14.5 | 14.7 | 14.3 | 14.4 | 14.8 | 15.0 | 15.0 | 15.3 | 15.3 | 14.9 | 14.9 | 15.3 | 15.3 | 15.6 | 14.6 |
| 188 | *T. vietnamense_*6 | 17.7 | 11.8 | 12.2 | 11.8 | 11.8 | 11.8 | 12.5 | 11.8 | 11.8 | 11.1 | 11.2 | 14.6 | 14.2 | 14.6 | 14.2 | 14.2 | 14.6 | 14.6 | 14.6 | 14.6 | 14.6 | 14.6 | 14.6 | 14.6 | 14.6 | 14.6 | 14.6 | 14.6 | 14.6 | 14.9 | 14.6 | 14.6 | 14.6 | 14.6 | 14.6 | 14.5 | 14.7 | 14.3 | 14.4 | 14.8 | 15.0 | 15.0 | 15.3 | 15.3 | 14.9 | 14.9 | 15.3 | 15.3 | 15.6 | 14.6 |
| 189 | *T. vietnamense_*7 | 17.7 | 11.8 | 12.2 | 11.8 | 11.8 | 11.8 | 12.5 | 11.8 | 11.8 | 11.1 | 11.2 | 14.6 | 14.2 | 14.6 | 14.2 | 14.2 | 14.6 | 14.6 | 14.6 | 14.6 | 14.6 | 14.6 | 14.6 | 14.6 | 14.6 | 14.6 | 14.6 | 14.6 | 14.6 | 14.9 | 14.6 | 14.6 | 14.6 | 14.6 | 14.6 | 14.5 | 14.7 | 14.3 | 14.4 | 14.8 | 15.0 | 15.0 | 15.3 | 15.3 | 14.9 | 14.9 | 15.3 | 15.3 | 15.6 | 14.6 |
| 190 | *T. vietnamense_*8 | 17.4 | 12.2 | 12.5 | 12.2 | 12.2 | 12.2 | 12.8 | 12.2 | 12.2 | 11.5 | 11.5 | 14.2 | 13.9 | 14.2 | 13.9 | 13.9 | 14.2 | 14.2 | 14.2 | 14.2 | 14.2 | 14.2 | 14.2 | 14.2 | 14.2 | 14.2 | 14.2 | 14.2 | 14.2 | 14.6 | 14.2 | 14.2 | 14.2 | 14.2 | 14.9 | 14.9 | 14.7 | 14.3 | 14.4 | 14.8 | 15.0 | 15.0 | 15.6 | 15.6 | 15.3 | 15.3 | 15.6 | 15.6 | 16.0 | 14.9 |
| 191 | *T. vietnamense_*9 | 17.4 | 12.2 | 12.5 | 12.2 | 12.2 | 12.2 | 12.8 | 12.2 | 12.2 | 11.5 | 11.5 | 14.2 | 13.9 | 14.2 | 13.9 | 13.9 | 14.2 | 14.2 | 14.2 | 14.2 | 14.2 | 14.2 | 14.2 | 14.2 | 14.2 | 14.2 | 14.2 | 14.2 | 14.2 | 14.6 | 14.2 | 14.2 | 14.2 | 14.2 | 14.9 | 14.9 | 14.7 | 14.3 | 14.4 | 14.8 | 15.0 | 15.0 | 15.6 | 15.6 | 15.3 | 15.3 | 15.6 | 15.6 | 16.0 | 14.9 |
| 192 | *T. vietnamense_*10 | 18.1 | 12.5 | 12.8 | 12.5 | 12.5 | 12.5 | 13.2 | 12.5 | 12.5 | 11.8 | 11.9 | 15.3 | 14.9 | 15.3 | 14.9 | 14.9 | 15.3 | 15.3 | 15.3 | 15.3 | 15.3 | 15.3 | 15.3 | 15.3 | 15.3 | 15.3 | 15.3 | 15.3 | 15.3 | 15.6 | 15.3 | 15.3 | 15.3 | 15.3 | 15.3 | 15.2 | 15.4 | 15.0 | 15.1 | 15.5 | 15.7 | 15.7 | 16.0 | 16.0 | 15.6 | 15.6 | 16.0 | 16.0 | 16.3 | 15.3 |
| 193 | *T. vietnamense_*11 | 17.7 | 11.8 | 12.2 | 11.8 | 11.8 | 11.8 | 12.5 | 11.8 | 11.8 | 11.1 | 11.2 | 14.6 | 14.2 | 14.6 | 14.2 | 14.2 | 14.6 | 14.6 | 14.6 | 14.6 | 14.6 | 14.6 | 14.6 | 14.6 | 14.6 | 14.6 | 14.6 | 14.6 | 14.6 | 14.9 | 14.6 | 14.6 | 14.6 | 14.6 | 14.6 | 14.5 | 14.7 | 14.3 | 14.4 | 14.8 | 15.0 | 15.0 | 15.3 | 15.3 | 14.9 | 14.9 | 15.3 | 15.3 | 15.6 | 14.6 |
| 194 | *T. vietnamense_*12 | 17.7 | 11.8 | 12.2 | 11.8 | 11.8 | 11.8 | 12.5 | 11.8 | 11.8 | 11.1 | 11.2 | 14.6 | 14.2 | 14.6 | 14.2 | 14.2 | 14.6 | 14.6 | 14.6 | 14.6 | 14.6 | 14.6 | 14.6 | 14.6 | 14.6 | 14.6 | 14.6 | 14.6 | 14.6 | 14.9 | 14.6 | 14.6 | 14.6 | 14.6 | 14.6 | 14.5 | 14.7 | 14.3 | 14.4 | 14.8 | 15.0 | 15.0 | 15.3 | 15.3 | 14.9 | 14.9 | 15.3 | 15.3 | 15.6 | 14.6 |
| 195 | *T. vietnamense_*13 | 17.7 | 11.8 | 12.2 | 11.8 | 11.8 | 11.8 | 12.5 | 11.8 | 11.8 | 11.1 | 11.2 | 14.6 | 14.2 | 14.6 | 14.2 | 14.2 | 14.6 | 14.6 | 14.6 | 14.6 | 14.6 | 14.6 | 14.6 | 14.6 | 14.6 | 14.6 | 14.6 | 14.6 | 14.6 | 14.9 | 14.6 | 14.6 | 14.6 | 14.6 | 14.6 | 14.5 | 14.7 | 14.3 | 14.4 | 14.8 | 15.0 | 15.0 | 15.3 | 15.3 | 14.9 | 14.9 | 15.3 | 15.3 | 15.6 | 14.6 |

Next page

Continue to

| ID | Species | 101 | 102 | 103 | 104 | 105 | 106 | 107 | 108 | 109 | 110 | 111 | 112 | 113 | 114 | 115 | 116 | 117 | 118 | 119 | 120 | 121 | 122 | 123 | 124 | 125 | 126 | 127 | 128 | 129 | 130 | 131 | 132 | 133 | 134 | 135 | 136 | 137 | 138 | 139 | 140 | 141 | 142 | 143 | 144 | 145 | 146 | 147 | 148 | 149 | 150 |
| --- | --- | --- | --- | --- | --- | --- | --- | --- | --- | --- | --- | --- | --- | --- | --- | --- | --- | --- | --- | --- | --- | --- | --- | --- | --- | --- | --- | --- | --- | --- | --- | --- | --- | --- | --- | --- | --- | --- | --- | --- | --- | --- | --- | --- | --- | --- | --- | --- | --- | --- | --- |
| 1 | *T. albopunctatum_*1 |  |  |  |  |  |  |  |  |  |  |  |  |  |  |  |  |  |  |  |  |  |  |  |  |  |  |  |  |  |  |  |  |  |  |  |  |  |  |  |  |  |  |  |  |  |  |  |  |  |  |
| 2 | *T. albopunctatum_*2 |  |  |  |  |  |  |  |  |  |  |  |  |  |  |  |  |  |  |  |  |  |  |  |  |  |  |  |  |  |  |  |  |  |  |  |  |  |  |  |  |  |  |  |  |  |  |  |  |  |  |
| 3 | *T. albopunctatum_*3 |  |  |  |  |  |  |  |  |  |  |  |  |  |  |  |  |  |  |  |  |  |  |  |  |  |  |  |  |  |  |  |  |  |  |  |  |  |  |  |  |  |  |  |  |  |  |  |  |  |  |
| 4 | *T. albopunctatum_*4 |  |  |  |  |  |  |  |  |  |  |  |  |  |  |  |  |  |  |  |  |  |  |  |  |  |  |  |  |  |  |  |  |  |  |  |  |  |  |  |  |  |  |  |  |  |  |  |  |  |  |
| 5 | *T. albopunctatum_*5 |  |  |  |  |  |  |  |  |  |  |  |  |  |  |  |  |  |  |  |  |  |  |  |  |  |  |  |  |  |  |  |  |  |  |  |  |  |  |  |  |  |  |  |  |  |  |  |  |  |  |
| 6 | *T. albopunctatum_*6 |  |  |  |  |  |  |  |  |  |  |  |  |  |  |  |  |  |  |  |  |  |  |  |  |  |  |  |  |  |  |  |  |  |  |  |  |  |  |  |  |  |  |  |  |  |  |  |  |  |  |
| 7 | *T. albopunctatum_*7 |  |  |  |  |  |  |  |  |  |  |  |  |  |  |  |  |  |  |  |  |  |  |  |  |  |  |  |  |  |  |  |  |  |  |  |  |  |  |  |  |  |  |  |  |  |  |  |  |  |  |
| 8 | *T. albopunctatum_*8 |  |  |  |  |  |  |  |  |  |  |  |  |  |  |  |  |  |  |  |  |  |  |  |  |  |  |  |  |  |  |  |  |  |  |  |  |  |  |  |  |  |  |  |  |  |  |  |  |  |  |
| 9 | *T. albopunctatum_*9 |  |  |  |  |  |  |  |  |  |  |  |  |  |  |  |  |  |  |  |  |  |  |  |  |  |  |  |  |  |  |  |  |  |  |  |  |  |  |  |  |  |  |  |  |  |  |  |  |  |  |
| 10 | *T. albopunctatum_*10 |  |  |  |  |  |  |  |  |  |  |  |  |  |  |  |  |  |  |  |  |  |  |  |  |  |  |  |  |  |  |  |  |  |  |  |  |  |  |  |  |  |  |  |  |  |  |  |  |  |  |
| 11 | *T. albopunctatum_*11 |  |  |  |  |  |  |  |  |  |  |  |  |  |  |  |  |  |  |  |  |  |  |  |  |  |  |  |  |  |  |  |  |  |  |  |  |  |  |  |  |  |  |  |  |  |  |  |  |  |  |
| 12 | *T. albopunctatum_*12 |  |  |  |  |  |  |  |  |  |  |  |  |  |  |  |  |  |  |  |  |  |  |  |  |  |  |  |  |  |  |  |  |  |  |  |  |  |  |  |  |  |  |  |  |  |  |  |  |  |  |
| 13 | *T. albopunctatum_*13 |  |  |  |  |  |  |  |  |  |  |  |  |  |  |  |  |  |  |  |  |  |  |  |  |  |  |  |  |  |  |  |  |  |  |  |  |  |  |  |  |  |  |  |  |  |  |  |  |  |  |
| 14 | *T. albopunctatum_*14 |  |  |  |  |  |  |  |  |  |  |  |  |  |  |  |  |  |  |  |  |  |  |  |  |  |  |  |  |  |  |  |  |  |  |  |  |  |  |  |  |  |  |  |  |  |  |  |  |  |  |
| 15 | *T. albopunctatum_*15 |  |  |  |  |  |  |  |  |  |  |  |  |  |  |  |  |  |  |  |  |  |  |  |  |  |  |  |  |  |  |  |  |  |  |  |  |  |  |  |  |  |  |  |  |  |  |  |  |  |  |
| 16 | *T. albopunctatum_*16 |  |  |  |  |  |  |  |  |  |  |  |  |  |  |  |  |  |  |  |  |  |  |  |  |  |  |  |  |  |  |  |  |  |  |  |  |  |  |  |  |  |  |  |  |  |  |  |  |  |  |
| 17 | *T. albopunctatum_*17 |  |  |  |  |  |  |  |  |  |  |  |  |  |  |  |  |  |  |  |  |  |  |  |  |  |  |  |  |  |  |  |  |  |  |  |  |  |  |  |  |  |  |  |  |  |  |  |  |  |  |
| 18 | *T. albopunctatum_*18 |  |  |  |  |  |  |  |  |  |  |  |  |  |  |  |  |  |  |  |  |  |  |  |  |  |  |  |  |  |  |  |  |  |  |  |  |  |  |  |  |  |  |  |  |  |  |  |  |  |  |
| 19 | *T. albopunctatum_*19 |  |  |  |  |  |  |  |  |  |  |  |  |  |  |  |  |  |  |  |  |  |  |  |  |  |  |  |  |  |  |  |  |  |  |  |  |  |  |  |  |  |  |  |  |  |  |  |  |  |  |
| 20 | *T. albopunctatum_*20 |  |  |  |  |  |  |  |  |  |  |  |  |  |  |  |  |  |  |  |  |  |  |  |  |  |  |  |  |  |  |  |  |  |  |  |  |  |  |  |  |  |  |  |  |  |  |  |  |  |  |
| 21 | *T. albopunctatum_*21 |  |  |  |  |  |  |  |  |  |  |  |  |  |  |  |  |  |  |  |  |  |  |  |  |  |  |  |  |  |  |  |  |  |  |  |  |  |  |  |  |  |  |  |  |  |  |  |  |  |  |
| 22 | *T. albopunctatum_*22 |  |  |  |  |  |  |  |  |  |  |  |  |  |  |  |  |  |  |  |  |  |  |  |  |  |  |  |  |  |  |  |  |  |  |  |  |  |  |  |  |  |  |  |  |  |  |  |  |  |  |
| 23 | *T. albopunctatum_*23 |  |  |  |  |  |  |  |  |  |  |  |  |  |  |  |  |  |  |  |  |  |  |  |  |  |  |  |  |  |  |  |  |  |  |  |  |  |  |  |  |  |  |  |  |  |  |  |  |  |  |
| 24 | *T. albopunctatum_*24 |  |  |  |  |  |  |  |  |  |  |  |  |  |  |  |  |  |  |  |  |  |  |  |  |  |  |  |  |  |  |  |  |  |  |  |  |  |  |  |  |  |  |  |  |  |  |  |  |  |  |
| 25 | *T. albopunctatum_*25 |  |  |  |  |  |  |  |  |  |  |  |  |  |  |  |  |  |  |  |  |  |  |  |  |  |  |  |  |  |  |  |  |  |  |  |  |  |  |  |  |  |  |  |  |  |  |  |  |  |  |
| 26 | *T. albopunctatum_*26 |  |  |  |  |  |  |  |  |  |  |  |  |  |  |  |  |  |  |  |  |  |  |  |  |  |  |  |  |  |  |  |  |  |  |  |  |  |  |  |  |  |  |  |  |  |  |  |  |  |  |
| 27 | *T. albopunctatum_*28 |  |  |  |  |  |  |  |  |  |  |  |  |  |  |  |  |  |  |  |  |  |  |  |  |  |  |  |  |  |  |  |  |  |  |  |  |  |  |  |  |  |  |  |  |  |  |  |  |  |  |
| 28 | *T. albopunctatum_*29 |  |  |  |  |  |  |  |  |  |  |  |  |  |  |  |  |  |  |  |  |  |  |  |  |  |  |  |  |  |  |  |  |  |  |  |  |  |  |  |  |  |  |  |  |  |  |  |  |  |  |
| 29 | *T. albopunctatum_*30 |  |  |  |  |  |  |  |  |  |  |  |  |  |  |  |  |  |  |  |  |  |  |  |  |  |  |  |  |  |  |  |  |  |  |  |  |  |  |  |  |  |  |  |  |  |  |  |  |  |  |
| 30 | *T. albopunctatum_*31 |  |  |  |  |  |  |  |  |  |  |  |  |  |  |  |  |  |  |  |  |  |  |  |  |  |  |  |  |  |  |  |  |  |  |  |  |  |  |  |  |  |  |  |  |  |  |  |  |  |  |
| 31 | *T. albopunctatum_*32 |  |  |  |  |  |  |  |  |  |  |  |  |  |  |  |  |  |  |  |  |  |  |  |  |  |  |  |  |  |  |  |  |  |  |  |  |  |  |  |  |  |  |  |  |  |  |  |  |  |  |
| 32 | *T. albopunctatum_*33 |  |  |  |  |  |  |  |  |  |  |  |  |  |  |  |  |  |  |  |  |  |  |  |  |  |  |  |  |  |  |  |  |  |  |  |  |  |  |  |  |  |  |  |  |  |  |  |  |  |  |
| 33 | *T. albopunctatum_*34 |  |  |  |  |  |  |  |  |  |  |  |  |  |  |  |  |  |  |  |  |  |  |  |  |  |  |  |  |  |  |  |  |  |  |  |  |  |  |  |  |  |  |  |  |  |  |  |  |  |  |
| 34 | *T. albopunctatum_*35 |  |  |  |  |  |  |  |  |  |  |  |  |  |  |  |  |  |  |  |  |  |  |  |  |  |  |  |  |  |  |  |  |  |  |  |  |  |  |  |  |  |  |  |  |  |  |  |  |  |  |
| 35 | *T. albopunctatum_*36 |  |  |  |  |  |  |  |  |  |  |  |  |  |  |  |  |  |  |  |  |  |  |  |  |  |  |  |  |  |  |  |  |  |  |  |  |  |  |  |  |  |  |  |  |  |  |  |  |  |  |
| 36 | *T. albopunctatum_*37 |  |  |  |  |  |  |  |  |  |  |  |  |  |  |  |  |  |  |  |  |  |  |  |  |  |  |  |  |  |  |  |  |  |  |  |  |  |  |  |  |  |  |  |  |  |  |  |  |  |  |
| 37 | *T. annae_*2 |  |  |  |  |  |  |  |  |  |  |  |  |  |  |  |  |  |  |  |  |  |  |  |  |  |  |  |  |  |  |  |  |  |  |  |  |  |  |  |  |  |  |  |  |  |  |  |  |  |  |
| 38 | *T. annae_*3 |  |  |  |  |  |  |  |  |  |  |  |  |  |  |  |  |  |  |  |  |  |  |  |  |  |  |  |  |  |  |  |  |  |  |  |  |  |  |  |  |  |  |  |  |  |  |  |  |  |  |
| 39 | *T. annae_*4 |  |  |  |  |  |  |  |  |  |  |  |  |  |  |  |  |  |  |  |  |  |  |  |  |  |  |  |  |  |  |  |  |  |  |  |  |  |  |  |  |  |  |  |  |  |  |  |  |  |  |
| 40 | *T. annae_*5 |  |  |  |  |  |  |  |  |  |  |  |  |  |  |  |  |  |  |  |  |  |  |  |  |  |  |  |  |  |  |  |  |  |  |  |  |  |  |  |  |  |  |  |  |  |  |  |  |  |  |
| 41 | *T. asperum_*1 |  |  |  |  |  |  |  |  |  |  |  |  |  |  |  |  |  |  |  |  |  |  |  |  |  |  |  |  |  |  |  |  |  |  |  |  |  |  |  |  |  |  |  |  |  |  |  |  |  |  |
| 42 | *T. asperum_*2 |  |  |  |  |  |  |  |  |  |  |  |  |  |  |  |  |  |  |  |  |  |  |  |  |  |  |  |  |  |  |  |  |  |  |  |  |  |  |  |  |  |  |  |  |  |  |  |  |  |  |
| 43 | *T. auratum_*1 |  |  |  |  |  |  |  |  |  |  |  |  |  |  |  |  |  |  |  |  |  |  |  |  |  |  |  |  |  |  |  |  |  |  |  |  |  |  |  |  |  |  |  |  |  |  |  |  |  |  |
| 44 | *T. auratum_*2 |  |  |  |  |  |  |  |  |  |  |  |  |  |  |  |  |  |  |  |  |  |  |  |  |  |  |  |  |  |  |  |  |  |  |  |  |  |  |  |  |  |  |  |  |  |  |  |  |  |  |
| 45 | *T. auratum_*3 |  |  |  |  |  |  |  |  |  |  |  |  |  |  |  |  |  |  |  |  |  |  |  |  |  |  |  |  |  |  |  |  |  |  |  |  |  |  |  |  |  |  |  |  |  |  |  |  |  |  |
| 46 | *T. auratum_*4 |  |  |  |  |  |  |  |  |  |  |  |  |  |  |  |  |  |  |  |  |  |  |  |  |  |  |  |  |  |  |  |  |  |  |  |  |  |  |  |  |  |  |  |  |  |  |  |  |  |  |
| 47 | *T. auratum_*5 |  |  |  |  |  |  |  |  |  |  |  |  |  |  |  |  |  |  |  |  |  |  |  |  |  |  |  |  |  |  |  |  |  |  |  |  |  |  |  |  |  |  |  |  |  |  |  |  |  |  |
| 48 | *T. auratum_*6 |  |  |  |  |  |  |  |  |  |  |  |  |  |  |  |  |  |  |  |  |  |  |  |  |  |  |  |  |  |  |  |  |  |  |  |  |  |  |  |  |  |  |  |  |  |  |  |  |  |  |
| 49 | *T. baibungense_*2 |  |  |  |  |  |  |  |  |  |  |  |  |  |  |  |  |  |  |  |  |  |  |  |  |  |  |  |  |  |  |  |  |  |  |  |  |  |  |  |  |  |  |  |  |  |  |  |  |  |  |
| 50 | *T. baibungense_*3 |  |  |  |  |  |  |  |  |  |  |  |  |  |  |  |  |  |  |  |  |  |  |  |  |  |  |  |  |  |  |  |  |  |  |  |  |  |  |  |  |  |  |  |  |  |  |  |  |  |  |
| 51 | *T. baibungense_*4 |  |  |  |  |  |  |  |  |  |  |  |  |  |  |  |  |  |  |  |  |  |  |  |  |  |  |  |  |  |  |  |  |  |  |  |  |  |  |  |  |  |  |  |  |  |  |  |  |  |  |
| 52 | *T. bicolor_*1 |  |  |  |  |  |  |  |  |  |  |  |  |  |  |  |  |  |  |  |  |  |  |  |  |  |  |  |  |  |  |  |  |  |  |  |  |  |  |  |  |  |  |  |  |  |  |  |  |  |  |
| 53 | *T. bicolor_*2 |  |  |  |  |  |  |  |  |  |  |  |  |  |  |  |  |  |  |  |  |  |  |  |  |  |  |  |  |  |  |  |  |  |  |  |  |  |  |  |  |  |  |  |  |  |  |  |  |  |  |
| 54 | *T. bicolor_*3 |  |  |  |  |  |  |  |  |  |  |  |  |  |  |  |  |  |  |  |  |  |  |  |  |  |  |  |  |  |  |  |  |  |  |  |  |  |  |  |  |  |  |  |  |  |  |  |  |  |  |
| 55 | *T. bicolor_*4 |  |  |  |  |  |  |  |  |  |  |  |  |  |  |  |  |  |  |  |  |  |  |  |  |  |  |  |  |  |  |  |  |  |  |  |  |  |  |  |  |  |  |  |  |  |  |  |  |  |  |
| 56 | *T. bicolor_*5 |  |  |  |  |  |  |  |  |  |  |  |  |  |  |  |  |  |  |  |  |  |  |  |  |  |  |  |  |  |  |  |  |  |  |  |  |  |  |  |  |  |  |  |  |  |  |  |  |  |  |
| 57 | *T. bicolor_*6 |  |  |  |  |  |  |  |  |  |  |  |  |  |  |  |  |  |  |  |  |  |  |  |  |  |  |  |  |  |  |  |  |  |  |  |  |  |  |  |  |  |  |  |  |  |  |  |  |  |  |
| 58 | *T. bicolor_*7 |  |  |  |  |  |  |  |  |  |  |  |  |  |  |  |  |  |  |  |  |  |  |  |  |  |  |  |  |  |  |  |  |  |  |  |  |  |  |  |  |  |  |  |  |  |  |  |  |  |  |
| 59 | *T. bicolor_*8 |  |  |  |  |  |  |  |  |  |  |  |  |  |  |  |  |  |  |  |  |  |  |  |  |  |  |  |  |  |  |  |  |  |  |  |  |  |  |  |  |  |  |  |  |  |  |  |  |  |  |
| 60 | *T. bicolor_*9 |  |  |  |  |  |  |  |  |  |  |  |  |  |  |  |  |  |  |  |  |  |  |  |  |  |  |  |  |  |  |  |  |  |  |  |  |  |  |  |  |  |  |  |  |  |  |  |  |  |  |
| 61 | *T. bicolor_*10 |  |  |  |  |  |  |  |  |  |  |  |  |  |  |  |  |  |  |  |  |  |  |  |  |  |  |  |  |  |  |  |  |  |  |  |  |  |  |  |  |  |  |  |  |  |  |  |  |  |  |
| 62 | *T. corticale_*1 |  |  |  |  |  |  |  |  |  |  |  |  |  |  |  |  |  |  |  |  |  |  |  |  |  |  |  |  |  |  |  |  |  |  |  |  |  |  |  |  |  |  |  |  |  |  |  |  |  |  |
| 63 | *T. corticale_*2 |  |  |  |  |  |  |  |  |  |  |  |  |  |  |  |  |  |  |  |  |  |  |  |  |  |  |  |  |  |  |  |  |  |  |  |  |  |  |  |  |  |  |  |  |  |  |  |  |  |  |
| 64 | *T. corticale_*3 |  |  |  |  |  |  |  |  |  |  |  |  |  |  |  |  |  |  |  |  |  |  |  |  |  |  |  |  |  |  |  |  |  |  |  |  |  |  |  |  |  |  |  |  |  |  |  |  |  |  |
| 65 | *T. corticale_*4 |  |  |  |  |  |  |  |  |  |  |  |  |  |  |  |  |  |  |  |  |  |  |  |  |  |  |  |  |  |  |  |  |  |  |  |  |  |  |  |  |  |  |  |  |  |  |  |  |  |  |
| 66 | *T. corticale_*5 |  |  |  |  |  |  |  |  |  |  |  |  |  |  |  |  |  |  |  |  |  |  |  |  |  |  |  |  |  |  |  |  |  |  |  |  |  |  |  |  |  |  |  |  |  |  |  |  |  |  |
| 67 | *T. corticale_*6 |  |  |  |  |  |  |  |  |  |  |  |  |  |  |  |  |  |  |  |  |  |  |  |  |  |  |  |  |  |  |  |  |  |  |  |  |  |  |  |  |  |  |  |  |  |  |  |  |  |  |
| 68 | *T. corticale_*7 |  |  |  |  |  |  |  |  |  |  |  |  |  |  |  |  |  |  |  |  |  |  |  |  |  |  |  |  |  |  |  |  |  |  |  |  |  |  |  |  |  |  |  |  |  |  |  |  |  |  |
| 69 | *T. corticale_*8 |  |  |  |  |  |  |  |  |  |  |  |  |  |  |  |  |  |  |  |  |  |  |  |  |  |  |  |  |  |  |  |  |  |  |  |  |  |  |  |  |  |  |  |  |  |  |  |  |  |  |
| 70 | *T. corticale_*9 |  |  |  |  |  |  |  |  |  |  |  |  |  |  |  |  |  |  |  |  |  |  |  |  |  |  |  |  |  |  |  |  |  |  |  |  |  |  |  |  |  |  |  |  |  |  |  |  |  |  |
| 71 | *T. corticale_*10 |  |  |  |  |  |  |  |  |  |  |  |  |  |  |  |  |  |  |  |  |  |  |  |  |  |  |  |  |  |  |  |  |  |  |  |  |  |  |  |  |  |  |  |  |  |  |  |  |  |  |
| 72 | *T. corticale_*11 |  |  |  |  |  |  |  |  |  |  |  |  |  |  |  |  |  |  |  |  |  |  |  |  |  |  |  |  |  |  |  |  |  |  |  |  |  |  |  |  |  |  |  |  |  |  |  |  |  |  |
| 73 | *T. corticale_*12 |  |  |  |  |  |  |  |  |  |  |  |  |  |  |  |  |  |  |  |  |  |  |  |  |  |  |  |  |  |  |  |  |  |  |  |  |  |  |  |  |  |  |  |  |  |  |  |  |  |  |
| 74 | *T. corticale_*13 |  |  |  |  |  |  |  |  |  |  |  |  |  |  |  |  |  |  |  |  |  |  |  |  |  |  |  |  |  |  |  |  |  |  |  |  |  |  |  |  |  |  |  |  |  |  |  |  |  |  |
| 75 | *T. corticale_*14 |  |  |  |  |  |  |  |  |  |  |  |  |  |  |  |  |  |  |  |  |  |  |  |  |  |  |  |  |  |  |  |  |  |  |  |  |  |  |  |  |  |  |  |  |  |  |  |  |  |  |
| 76 | *T. corticale_*15 |  |  |  |  |  |  |  |  |  |  |  |  |  |  |  |  |  |  |  |  |  |  |  |  |  |  |  |  |  |  |  |  |  |  |  |  |  |  |  |  |  |  |  |  |  |  |  |  |  |  |
| 77 | *T. corticale_*16 |  |  |  |  |  |  |  |  |  |  |  |  |  |  |  |  |  |  |  |  |  |  |  |  |  |  |  |  |  |  |  |  |  |  |  |  |  |  |  |  |  |  |  |  |  |  |  |  |  |  |
| 78 | *T. corticale_*17 |  |  |  |  |  |  |  |  |  |  |  |  |  |  |  |  |  |  |  |  |  |  |  |  |  |  |  |  |  |  |  |  |  |  |  |  |  |  |  |  |  |  |  |  |  |  |  |  |  |  |
| 79 | *T. corticale_*18 |  |  |  |  |  |  |  |  |  |  |  |  |  |  |  |  |  |  |  |  |  |  |  |  |  |  |  |  |  |  |  |  |  |  |  |  |  |  |  |  |  |  |  |  |  |  |  |  |  |  |
| 80 | *T. corticale_*19 |  |  |  |  |  |  |  |  |  |  |  |  |  |  |  |  |  |  |  |  |  |  |  |  |  |  |  |  |  |  |  |  |  |  |  |  |  |  |  |  |  |  |  |  |  |  |  |  |  |  |
| 81 | *T. corticale_*20 |  |  |  |  |  |  |  |  |  |  |  |  |  |  |  |  |  |  |  |  |  |  |  |  |  |  |  |  |  |  |  |  |  |  |  |  |  |  |  |  |  |  |  |  |  |  |  |  |  |  |
| 82 | *T. corticale_*21 |  |  |  |  |  |  |  |  |  |  |  |  |  |  |  |  |  |  |  |  |  |  |  |  |  |  |  |  |  |  |  |  |  |  |  |  |  |  |  |  |  |  |  |  |  |  |  |  |  |  |
| 83 | *T. corticale_*22 |  |  |  |  |  |  |  |  |  |  |  |  |  |  |  |  |  |  |  |  |  |  |  |  |  |  |  |  |  |  |  |  |  |  |  |  |  |  |  |  |  |  |  |  |  |  |  |  |  |  |
| 84 | *T. corticale_*23 |  |  |  |  |  |  |  |  |  |  |  |  |  |  |  |  |  |  |  |  |  |  |  |  |  |  |  |  |  |  |  |  |  |  |  |  |  |  |  |  |  |  |  |  |  |  |  |  |  |  |
| 85 | *T. gordoni_*1 |  |  |  |  |  |  |  |  |  |  |  |  |  |  |  |  |  |  |  |  |  |  |  |  |  |  |  |  |  |  |  |  |  |  |  |  |  |  |  |  |  |  |  |  |  |  |  |  |  |  |
| 86 | *T. gordoni_*2 |  |  |  |  |  |  |  |  |  |  |  |  |  |  |  |  |  |  |  |  |  |  |  |  |  |  |  |  |  |  |  |  |  |  |  |  |  |  |  |  |  |  |  |  |  |  |  |  |  |  |
| 87 | *T. gordoni_*3 |  |  |  |  |  |  |  |  |  |  |  |  |  |  |  |  |  |  |  |  |  |  |  |  |  |  |  |  |  |  |  |  |  |  |  |  |  |  |  |  |  |  |  |  |  |  |  |  |  |  |
| 88 | *T. gordoni_*4 |  |  |  |  |  |  |  |  |  |  |  |  |  |  |  |  |  |  |  |  |  |  |  |  |  |  |  |  |  |  |  |  |  |  |  |  |  |  |  |  |  |  |  |  |  |  |  |  |  |  |
| 89 | *T. gordoni_*5 |  |  |  |  |  |  |  |  |  |  |  |  |  |  |  |  |  |  |  |  |  |  |  |  |  |  |  |  |  |  |  |  |  |  |  |  |  |  |  |  |  |  |  |  |  |  |  |  |  |  |
| 90 | *T. gordoni_*6 |  |  |  |  |  |  |  |  |  |  |  |  |  |  |  |  |  |  |  |  |  |  |  |  |  |  |  |  |  |  |  |  |  |  |  |  |  |  |  |  |  |  |  |  |  |  |  |  |  |  |
| 91 | *T. gordoni_*7 |  |  |  |  |  |  |  |  |  |  |  |  |  |  |  |  |  |  |  |  |  |  |  |  |  |  |  |  |  |  |  |  |  |  |  |  |  |  |  |  |  |  |  |  |  |  |  |  |  |  |
| 92 | *T. gordoni_*8 |  |  |  |  |  |  |  |  |  |  |  |  |  |  |  |  |  |  |  |  |  |  |  |  |  |  |  |  |  |  |  |  |  |  |  |  |  |  |  |  |  |  |  |  |  |  |  |  |  |  |
| 93 | *T. hekouense_*1 |  |  |  |  |  |  |  |  |  |  |  |  |  |  |  |  |  |  |  |  |  |  |  |  |  |  |  |  |  |  |  |  |  |  |  |  |  |  |  |  |  |  |  |  |  |  |  |  |  |  |
| 94 | *T. hekouense_*2 |  |  |  |  |  |  |  |  |  |  |  |  |  |  |  |  |  |  |  |  |  |  |  |  |  |  |  |  |  |  |  |  |  |  |  |  |  |  |  |  |  |  |  |  |  |  |  |  |  |  |
| 95 | *T. hekouense_*3 |  |  |  |  |  |  |  |  |  |  |  |  |  |  |  |  |  |  |  |  |  |  |  |  |  |  |  |  |  |  |  |  |  |  |  |  |  |  |  |  |  |  |  |  |  |  |  |  |  |  |
| 96 | *T. hekouense_*4 |  |  |  |  |  |  |  |  |  |  |  |  |  |  |  |  |  |  |  |  |  |  |  |  |  |  |  |  |  |  |  |  |  |  |  |  |  |  |  |  |  |  |  |  |  |  |  |  |  |  |
| 97 | *T. hekouense_*5 |  |  |  |  |  |  |  |  |  |  |  |  |  |  |  |  |  |  |  |  |  |  |  |  |  |  |  |  |  |  |  |  |  |  |  |  |  |  |  |  |  |  |  |  |  |  |  |  |  |  |
| 98 | *T. hekouense_*6 |  |  |  |  |  |  |  |  |  |  |  |  |  |  |  |  |  |  |  |  |  |  |  |  |  |  |  |  |  |  |  |  |  |  |  |  |  |  |  |  |  |  |  |  |  |  |  |  |  |  |
| 99 | *T. hekouense_*7 |  |  |  |  |  |  |  |  |  |  |  |  |  |  |  |  |  |  |  |  |  |  |  |  |  |  |  |  |  |  |  |  |  |  |  |  |  |  |  |  |  |  |  |  |  |  |  |  |  |  |
| 100 | *T. hekouense_*8 |  |  |  |  |  |  |  |  |  |  |  |  |  |  |  |  |  |  |  |  |  |  |  |  |  |  |  |  |  |  |  |  |  |  |  |  |  |  |  |  |  |  |  |  |  |  |  |  |  |  |
| 101 | *T. hekouense_*9 |  |  |  |  |  |  |  |  |  |  |  |  |  |  |  |  |  |  |  |  |  |  |  |  |  |  |  |  |  |  |  |  |  |  |  |  |  |  |  |  |  |  |  |  |  |  |  |  |  |  |
| 102 | *T. hekouense_*10 | 0.0 |  |  |  |  |  |  |  |  |  |  |  |  |  |  |  |  |  |  |  |  |  |  |  |  |  |  |  |  |  |  |  |  |  |  |  |  |  |  |  |  |  |  |  |  |  |  |  |  |  |
| 103 | *T. horridum_*1 | 15.3 | 15.3 |  |  |  |  |  |  |  |  |  |  |  |  |  |  |  |  |  |  |  |  |  |  |  |  |  |  |  |  |  |  |  |  |  |  |  |  |  |  |  |  |  |  |  |  |  |  |  |  |
| 104 | *T. horridum_*2 | 15.3 | 15.3 | 0.0 |  |  |  |  |  |  |  |  |  |  |  |  |  |  |  |  |  |  |  |  |  |  |  |  |  |  |  |  |  |  |  |  |  |  |  |  |  |  |  |  |  |  |  |  |  |  |  |
| 105 | *T. horridum_*3 | 15.3 | 15.3 | 0.7 | 0.7 |  |  |  |  |  |  |  |  |  |  |  |  |  |  |  |  |  |  |  |  |  |  |  |  |  |  |  |  |  |  |  |  |  |  |  |  |  |  |  |  |  |  |  |  |  |  |
| 106 | *T. horridum5* | 15.6 | 15.6 | 0.7 | 0.7 | 0.7 |  |  |  |  |  |  |  |  |  |  |  |  |  |  |  |  |  |  |  |  |  |  |  |  |  |  |  |  |  |  |  |  |  |  |  |  |  |  |  |  |  |  |  |  |  |
| 107 | *T. khoii_*1 | 8.7 | 8.7 | 13.9 | 13.9 | 13.5 | 14.2 |  |  |  |  |  |  |  |  |  |  |  |  |  |  |  |  |  |  |  |  |  |  |  |  |  |  |  |  |  |  |  |  |  |  |  |  |  |  |  |  |  |  |  |  |
| 108 | *T. khoii*2 | 8.7 | 8.7 | 13.9 | 13.9 | 13.5 | 14.2 | 0.0 |  |  |  |  |  |  |  |  |  |  |  |  |  |  |  |  |  |  |  |  |  |  |  |  |  |  |  |  |  |  |  |  |  |  |  |  |  |  |  |  |  |  |  |
| 109 | *T. lacustrinum_*1 | 10.8 | 10.8 | 16.0 | 16.0 | 16.0 | 16.7 | 9.0 | 9.0 |  |  |  |  |  |  |  |  |  |  |  |  |  |  |  |  |  |  |  |  |  |  |  |  |  |  |  |  |  |  |  |  |  |  |  |  |  |  |  |  |  |  |
| 110 | *T. lacustrinum_*2 | 10.8 | 10.8 | 16.0 | 16.0 | 16.0 | 16.7 | 9.0 | 9.0 | 0.0 |  |  |  |  |  |  |  |  |  |  |  |  |  |  |  |  |  |  |  |  |  |  |  |  |  |  |  |  |  |  |  |  |  |  |  |  |  |  |  |  |  |
| 111 | *T. laeve_*1 | 12.5 | 12.5 | 18.8 | 18.8 | 18.8 | 19.1 | 12.2 | 12.2 | 12.8 | 12.8 |  |  |  |  |  |  |  |  |  |  |  |  |  |  |  |  |  |  |  |  |  |  |  |  |  |  |  |  |  |  |  |  |  |  |  |  |  |  |  |  |
| 112 | *T. laeve_*2 | 14.6 | 14.6 | 21.2 | 21.2 | 21.2 | 21.5 | 14.9 | 14.9 | 16.7 | 16.7 | 9.7 |  |  |  |  |  |  |  |  |  |  |  |  |  |  |  |  |  |  |  |  |  |  |  |  |  |  |  |  |  |  |  |  |  |  |  |  |  |  |  |
| 113 | *T. laeve_*3 | 15.3 | 15.3 | 21.2 | 21.2 | 21.2 | 21.5 | 14.9 | 14.9 | 16.7 | 16.7 | 10.4 | 1.4 |  |  |  |  |  |  |  |  |  |  |  |  |  |  |  |  |  |  |  |  |  |  |  |  |  |  |  |  |  |  |  |  |  |  |  |  |  |  |
| 114 | *T. laeve_*4 | 14.6 | 14.6 | 21.9 | 21.9 | 21.9 | 22.2 | 15.6 | 15.6 | 17.0 | 17.0 | 10.4 | 0.7 | 1.4 |  |  |  |  |  |  |  |  |  |  |  |  |  |  |  |  |  |  |  |  |  |  |  |  |  |  |  |  |  |  |  |  |  |  |  |  |  |
| 115 | *T. laeve_*5 | 14.2 | 14.2 | 22.2 | 22.2 | 22.2 | 22.6 | 15.3 | 15.3 | 17.4 | 17.4 | 10.8 | 1.7 | 2.4 | 1.0 |  |  |  |  |  |  |  |  |  |  |  |  |  |  |  |  |  |  |  |  |  |  |  |  |  |  |  |  |  |  |  |  |  |  |  |  |
| 116 | *T. laeve_*6 | 14.2 | 14.2 | 22.2 | 22.2 | 22.2 | 22.6 | 15.3 | 15.3 | 17.4 | 17.4 | 10.8 | 1.7 | 2.4 | 1.0 | 0.0 |  |  |  |  |  |  |  |  |  |  |  |  |  |  |  |  |  |  |  |  |  |  |  |  |  |  |  |  |  |  |  |  |  |  |  |
| 117 | *T. laeve_*7 | 14.2 | 14.2 | 22.2 | 22.2 | 22.2 | 22.6 | 15.3 | 15.3 | 17.4 | 17.4 | 10.8 | 1.7 | 2.4 | 1.0 | 0.0 | 0.0 |  |  |  |  |  |  |  |  |  |  |  |  |  |  |  |  |  |  |  |  |  |  |  |  |  |  |  |  |  |  |  |  |  |  |
| 118 | *T. laeve_*8 | 14.9 | 14.9 | 22.2 | 22.2 | 22.2 | 22.6 | 16.0 | 16.0 | 17.4 | 17.4 | 10.8 | 1.0 | 1.7 | 0.3 | 1.4 | 1.4 | 1.4 |  |  |  |  |  |  |  |  |  |  |  |  |  |  |  |  |  |  |  |  |  |  |  |  |  |  |  |  |  |  |  |  |  |
| 119 | *T. laeve_*9 | 16.1 | 16.1 | 24.0 | 24.0 | 24.0 | 24.4 | 16.9 | 16.9 | 18.1 | 18.1 | 11.4 | 1.2 | 2.0 | 0.4 | 1.6 | 1.6 | 1.6 | 0.0 |  |  |  |  |  |  |  |  |  |  |  |  |  |  |  |  |  |  |  |  |  |  |  |  |  |  |  |  |  |  |  |  |
| 120 | *T. laeve_*10 | 14.9 | 14.9 | 22.9 | 22.9 | 22.9 | 23.3 | 16.0 | 16.0 | 18.1 | 18.1 | 11.5 | 1.7 | 2.4 | 1.0 | 1.4 | 1.4 | 1.4 | 0.7 | 0.8 |  |  |  |  |  |  |  |  |  |  |  |  |  |  |  |  |  |  |  |  |  |  |  |  |  |  |  |  |  |  |  |
| 121 | *T. lateriticum_*1 | 12.5 | 12.5 | 17.4 | 17.4 | 17.4 | 18.1 | 11.1 | 11.1 | 8.0 | 8.0 | 12.8 | 16.7 | 16.0 | 17.4 | 17.7 | 17.7 | 17.7 | 17.7 | 18.9 | 18.4 |  |  |  |  |  |  |  |  |  |  |  |  |  |  |  |  |  |  |  |  |  |  |  |  |  |  |  |  |  |  |
| 122 | *T. lateriticum_*2 | 12.2 | 12.2 | 16.3 | 16.3 | 16.3 | 17.0 | 11.5 | 11.5 | 9.0 | 9.0 | 13.2 | 15.6 | 16.3 | 16.3 | 16.7 | 16.7 | 16.7 | 16.7 | 17.3 | 17.4 | 3.1 |  |  |  |  |  |  |  |  |  |  |  |  |  |  |  |  |  |  |  |  |  |  |  |  |  |  |  |  |  |
| 123 | *T. lateriticum_*3 | 10.4 | 10.4 | 16.0 | 16.0 | 16.0 | 16.7 | 10.4 | 10.4 | 6.9 | 6.9 | 12.2 | 15.3 | 16.0 | 16.0 | 16.3 | 16.3 | 16.3 | 16.3 | 17.3 | 17.0 | 2.1 | 2.4 |  |  |  |  |  |  |  |  |  |  |  |  |  |  |  |  |  |  |  |  |  |  |  |  |  |  |  |  |
| 124 | *T. lateriticum_*4 | 12.5 | 12.5 | 15.3 | 15.3 | 15.3 | 16.0 | 10.4 | 10.4 | 8.3 | 8.3 | 13.5 | 16.7 | 17.4 | 17.4 | 17.7 | 17.7 | 17.7 | 17.7 | 18.9 | 18.4 | 4.2 | 4.5 | 2.8 |  |  |  |  |  |  |  |  |  |  |  |  |  |  |  |  |  |  |  |  |  |  |  |  |  |  |  |
| 125 | *T. lateriticum_*5 | 11.5 | 11.5 | 15.6 | 15.6 | 15.6 | 16.3 | 10.4 | 10.4 | 6.9 | 6.9 | 13.5 | 16.0 | 16.7 | 16.7 | 17.0 | 17.0 | 17.0 | 17.0 | 18.1 | 17.7 | 3.5 | 3.8 | 2.8 | 2.1 |  |  |  |  |  |  |  |  |  |  |  |  |  |  |  |  |  |  |  |  |  |  |  |  |  |  |
| 126 | *T. lateriticum_*6 | 11.5 | 11.5 | 15.6 | 15.6 | 15.6 | 16.3 | 10.4 | 10.4 | 6.9 | 6.9 | 13.5 | 16.0 | 16.7 | 16.7 | 17.0 | 17.0 | 17.0 | 17.0 | 18.1 | 17.7 | 3.5 | 3.8 | 2.8 | 2.1 | 0.0 |  |  |  |  |  |  |  |  |  |  |  |  |  |  |  |  |  |  |  |  |  |  |  |  |  |
| 127 | *T. lateriticum_*LT01 | 11.5 | 11.5 | 15.3 | 15.3 | 15.3 | 16.0 | 10.8 | 10.8 | 7.6 | 7.6 | 12.5 | 15.6 | 16.3 | 16.3 | 16.7 | 16.7 | 16.7 | 16.7 | 17.7 | 17.4 | 3.1 | 2.8 | 1.0 | 1.7 | 1.7 | 1.7 |  |  |  |  |  |  |  |  |  |  |  |  |  |  |  |  |  |  |  |  |  |  |  |  |
| 128 | *T. lateriticum_*LT011 | 11.5 | 11.5 | 15.3 | 15.3 | 15.3 | 16.0 | 10.8 | 10.8 | 7.6 | 7.6 | 12.5 | 15.6 | 16.3 | 16.3 | 16.7 | 16.7 | 16.7 | 16.7 | 17.7 | 17.4 | 3.1 | 2.8 | 1.0 | 1.7 | 1.7 | 1.7 | 0.0 |  |  |  |  |  |  |  |  |  |  |  |  |  |  |  |  |  |  |  |  |  |  |  |
| 129 | *T. leporosum_*1 | 11.5 | 11.5 | 17.0 | 17.0 | 16.3 | 16.7 | 9.4 | 9.4 | 11.1 | 11.1 | 11.8 | 17.0 | 17.0 | 17.7 | 18.1 | 18.1 | 18.1 | 18.1 | 18.1 | 18.8 | 11.1 | 11.5 | 10.4 | 11.1 | 11.8 | 11.8 | 10.8 | 10.8 |  |  |  |  |  |  |  |  |  |  |  |  |  |  |  |  |  |  |  |  |  |  |
| 130 | *T. leporosum_*2 | 11.5 | 11.5 | 17.0 | 17.0 | 16.3 | 16.7 | 9.4 | 9.4 | 11.1 | 11.1 | 11.8 | 17.0 | 17.0 | 17.7 | 18.1 | 18.1 | 18.1 | 18.1 | 18.1 | 18.8 | 11.1 | 11.5 | 10.4 | 11.1 | 11.8 | 11.8 | 10.8 | 10.8 | 0.0 |  |  |  |  |  |  |  |  |  |  |  |  |  |  |  |  |  |  |  |  |  |
| 131 | *T. leporosum_*3 | 11.5 | 11.5 | 17.0 | 17.0 | 16.3 | 16.7 | 9.4 | 9.4 | 11.1 | 11.1 | 11.8 | 17.0 | 17.0 | 17.7 | 18.1 | 18.1 | 18.1 | 18.1 | 18.1 | 18.8 | 11.1 | 11.5 | 10.4 | 11.1 | 11.8 | 11.8 | 10.8 | 10.8 | 0.0 | 0.0 |  |  |  |  |  |  |  |  |  |  |  |  |  |  |  |  |  |  |  |  |
| 132 | *T. licin_*1 | 12.8 | 12.8 | 16.3 | 16.3 | 16.3 | 16.7 | 12.8 | 12.8 | 14.2 | 14.2 | 13.2 | 16.7 | 16.0 | 16.3 | 16.0 | 16.0 | 16.0 | 16.7 | 17.7 | 16.7 | 17.4 | 17.0 | 16.0 | 15.3 | 14.9 | 14.9 | 14.9 | 14.9 | 14.6 | 14.6 | 14.6 |  |  |  |  |  |  |  |  |  |  |  |  |  |  |  |  |  |  |  |
| 133 | *T. licin_*2 | 13.2 | 13.2 | 17.4 | 17.4 | 17.4 | 17.7 | 13.9 | 13.9 | 14.6 | 14.6 | 14.6 | 16.7 | 16.0 | 16.3 | 16.0 | 16.0 | 16.0 | 16.7 | 17.7 | 16.7 | 17.4 | 17.0 | 16.0 | 15.6 | 14.9 | 14.9 | 14.9 | 14.9 | 14.9 | 14.9 | 14.9 | 2.8 |  |  |  |  |  |  |  |  |  |  |  |  |  |  |  |  |  |  |
| 134 | *T. licin_*3 | 12.8 | 12.8 | 17.0 | 17.0 | 17.0 | 17.4 | 13.2 | 13.2 | 14.6 | 14.6 | 14.6 | 17.0 | 16.3 | 16.7 | 16.3 | 16.3 | 16.3 | 17.0 | 17.7 | 17.0 | 17.7 | 17.4 | 16.3 | 15.3 | 15.3 | 15.3 | 15.3 | 15.3 | 15.6 | 15.6 | 15.6 | 2.8 | 2.8 |  |  |  |  |  |  |  |  |  |  |  |  |  |  |  |  |  |
| 135 | *T. moloch_*1 | 9.4 | 9.4 | 16.0 | 16.0 | 16.0 | 16.3 | 10.1 | 10.1 | 10.8 | 10.8 | 9.4 | 12.8 | 12.8 | 13.5 | 13.9 | 13.9 | 13.9 | 13.9 | 14.6 | 14.6 | 13.5 | 13.2 | 12.2 | 12.2 | 12.2 | 12.2 | 11.8 | 11.8 | 9.0 | 9.0 | 9.0 | 10.8 | 11.8 | 11.8 |  |  |  |  |  |  |  |  |  |  |  |  |  |  |  |  |
| 136 | *T. moloch_*2 | 9.4 | 9.4 | 16.0 | 16.0 | 16.0 | 16.3 | 10.1 | 10.1 | 10.8 | 10.8 | 9.4 | 12.8 | 12.8 | 13.5 | 13.9 | 13.9 | 13.9 | 13.9 | 14.6 | 14.6 | 13.5 | 13.2 | 12.2 | 12.2 | 12.2 | 12.2 | 11.8 | 11.8 | 9.0 | 9.0 | 9.0 | 10.8 | 11.8 | 11.8 | 0.0 |  |  |  |  |  |  |  |  |  |  |  |  |  |  |  |
| 137 | *T. moloch_*4 | 6.0 | 6.0 | 11.6 | 11.6 | 11.6 | 12.1 | 7.9 | 7.9 | 7.4 | 7.4 | 7.0 | 9.8 | 9.8 | 10.2 | 10.7 | 10.7 | 10.7 | 10.7 | 11.0 | 11.2 | 8.4 | 8.8 | 7.4 | 7.9 | 7.4 | 7.4 | 7.0 | 7.0 | 6.0 | 6.0 | 6.0 | 7.9 | 8.8 | 9.3 | 0.0 | 0.0 |  |  |  |  |  |  |  |  |  |  |  |  |  |  |
| 138 | *T. nebulosum_*1 | 15.3 | 15.3 | 19.2 | 19.2 | 19.2 | 19.9 | 13.2 | 13.2 | 16.4 | 16.4 | 10.8 | 14.3 | 13.6 | 15.0 | 15.3 | 15.3 | 15.3 | 15.3 | 14.6 | 16.0 | 15.3 | 16.4 | 15.3 | 15.3 | 15.3 | 15.3 | 15.0 | 15.0 | 13.2 | 13.2 | 13.2 | 15.3 | 15.3 | 16.0 | 13.2 | 13.2 | 11.2 |  |  |  |  |  |  |  |  |  |  |  |  |  |
| 139 | *T. nebulosum_*2 | 12.2 | 12.2 | 17.0 | 17.0 | 17.0 | 17.7 | 10.4 | 10.4 | 13.5 | 13.5 | 8.0 | 11.1 | 11.8 | 11.8 | 12.2 | 12.2 | 12.2 | 12.2 | 13.0 | 12.8 | 13.2 | 12.8 | 11.8 | 11.8 | 11.8 | 11.8 | 11.5 | 11.5 | 10.4 | 10.4 | 10.4 | 12.8 | 12.8 | 13.5 | 10.4 | 10.4 | 7.4 | 3.5 |  |  |  |  |  |  |  |  |  |  |  |  |
| 140 | *T. nebulosum_*3 | 12.2 | 12.2 | 17.0 | 17.0 | 17.0 | 17.7 | 10.4 | 10.4 | 13.5 | 13.5 | 8.0 | 11.1 | 11.8 | 11.8 | 12.2 | 12.2 | 12.2 | 12.2 | 13.0 | 12.8 | 13.2 | 12.8 | 11.8 | 11.8 | 11.8 | 11.8 | 11.5 | 11.5 | 10.4 | 10.4 | 10.4 | 12.8 | 12.8 | 13.5 | 10.4 | 10.4 | 7.4 | 3.5 | 0.0 |  |  |  |  |  |  |  |  |  |  |  |
| 141 | *T. nebulosum_*4 | 11.8 | 11.8 | 17.1 | 17.1 | 17.1 | 17.8 | 10.5 | 10.5 | 13.2 | 13.2 | 8.0 | 11.1 | 11.8 | 11.5 | 11.8 | 11.8 | 11.8 | 11.8 | 12.6 | 12.5 | 13.2 | 12.9 | 11.8 | 11.8 | 11.8 | 11.8 | 11.5 | 11.5 | 10.5 | 10.5 | 10.5 | 12.5 | 12.5 | 13.2 | 10.5 | 10.5 | 7.4 | 3.5 | 0.0 | 0.0 |  |  |  |  |  |  |  |  |  |  |
| 142 | *T. palliatum_*1 | 9.4 | 9.4 | 15.6 | 15.6 | 15.3 | 16.0 | 9.4 | 9.4 | 9.7 | 9.7 | 14.6 | 17.0 | 16.3 | 17.0 | 16.7 | 16.7 | 16.7 | 17.4 | 18.5 | 16.7 | 11.1 | 10.8 | 10.4 | 11.5 | 11.1 | 11.1 | 10.4 | 10.4 | 11.1 | 11.1 | 11.1 | 16.0 | 16.0 | 16.7 | 12.5 | 12.5 | 8.4 | 15.0 | 13.2 | 13.2 | 12.9 |  |  |  |  |  |  |  |  |  |
| 143 | *T. palliatum_*2 | 9.7 | 9.7 | 15.3 | 15.3 | 14.9 | 15.6 | 9.7 | 9.7 | 10.1 | 10.1 | 14.9 | 17.4 | 16.7 | 17.4 | 17.0 | 17.0 | 17.0 | 17.7 | 18.9 | 17.0 | 11.5 | 11.1 | 10.8 | 11.8 | 11.5 | 11.5 | 10.8 | 10.8 | 11.5 | 11.5 | 11.5 | 16.3 | 16.3 | 17.0 | 12.8 | 12.8 | 8.8 | 14.6 | 13.5 | 13.5 | 13.2 | 0.3 |  |  |  |  |  |  |  |  |
| 144 | *T. palliatum_*3 | 9.0 | 9.0 | 15.3 | 15.3 | 14.9 | 15.6 | 9.0 | 9.0 | 9.4 | 9.4 | 14.2 | 16.7 | 16.7 | 16.7 | 16.3 | 16.3 | 16.3 | 17.0 | 18.1 | 16.3 | 11.5 | 10.4 | 10.1 | 11.1 | 10.8 | 10.8 | 10.1 | 10.1 | 10.8 | 10.8 | 10.8 | 15.6 | 15.6 | 16.3 | 12.2 | 12.2 | 7.9 | 15.3 | 12.8 | 12.8 | 12.5 | 0.3 | 0.7 |  |  |  |  |  |  |  |
| 145 | *T. palliatum_*4 | 10.1 | 10.1 | 15.6 | 15.6 | 15.3 | 16.0 | 10.1 | 10.1 | 9.7 | 9.7 | 14.6 | 17.0 | 16.3 | 17.0 | 16.7 | 16.7 | 16.7 | 17.4 | 18.5 | 16.7 | 11.1 | 10.8 | 10.4 | 11.5 | 11.1 | 11.1 | 10.4 | 10.4 | 11.8 | 11.8 | 11.8 | 16.0 | 16.0 | 16.7 | 12.5 | 12.5 | 8.4 | 15.0 | 13.9 | 13.9 | 13.6 | 0.7 | 0.3 | 1.0 |  |  |  |  |  |  |
| 146 | *T. palliatum_*5 | 9.0 | 9.0 | 15.3 | 15.3 | 14.9 | 15.6 | 9.0 | 9.0 | 9.4 | 9.4 | 14.2 | 16.7 | 16.7 | 16.7 | 16.3 | 16.3 | 16.3 | 17.0 | 18.1 | 16.3 | 11.5 | 10.4 | 10.1 | 11.1 | 10.8 | 10.8 | 10.1 | 10.1 | 10.8 | 10.8 | 10.8 | 15.6 | 15.6 | 16.3 | 12.2 | 12.2 | 7.9 | 15.3 | 12.8 | 12.8 | 12.5 | 0.3 | 0.7 | 0.0 | 1.0 |  |  |  |  |  |
| 147 | *T. palliatum_*6 | 9.4 | 9.4 | 15.6 | 15.6 | 15.3 | 16.0 | 9.4 | 9.4 | 9.0 | 9.0 | 13.9 | 16.3 | 16.3 | 16.3 | 16.0 | 16.0 | 16.0 | 16.7 | 17.7 | 16.0 | 11.1 | 10.1 | 9.7 | 10.8 | 10.4 | 10.4 | 9.7 | 9.7 | 11.1 | 11.1 | 11.1 | 15.3 | 15.3 | 16.0 | 11.8 | 11.8 | 7.4 | 15.7 | 13.2 | 13.2 | 12.9 | 0.7 | 1.0 | 0.3 | 0.7 | 0.3 |  |  |  |  |
| 148 | *T. palliatum_*7 | 10.3 | 10.3 | 16.7 | 16.7 | 16.3 | 17.1 | 9.9 | 9.9 | 9.9 | 9.9 | 15.5 | 17.1 | 17.1 | 17.1 | 17.1 | 17.1 | 17.1 | 17.5 | 17.5 | 16.7 | 11.9 | 10.3 | 10.3 | 11.5 | 11.1 | 11.1 | 10.3 | 10.3 | 11.5 | 11.5 | 11.5 | 16.7 | 16.7 | 17.1 | 13.5 | 13.5 | 8.9 | 15.5 | 14.7 | 14.7 | 14.3 | 0.8 | 1.2 | 0.4 | 0.8 | 0.4 | 0.0 |  |  |  |
| 149 | *T. palliatum_*8 | 9.7 | 9.7 | 16.0 | 16.0 | 15.6 | 16.3 | 9.7 | 9.7 | 9.4 | 9.4 | 14.2 | 16.7 | 16.7 | 16.7 | 16.3 | 16.3 | 16.3 | 17.0 | 17.7 | 16.3 | 11.5 | 10.4 | 10.1 | 11.1 | 10.8 | 10.8 | 10.1 | 10.1 | 11.5 | 11.5 | 11.5 | 15.6 | 15.6 | 16.3 | 12.2 | 12.2 | 7.9 | 16.0 | 13.5 | 13.5 | 13.2 | 1.0 | 1.4 | 0.7 | 1.0 | 0.7 | 0.3 | 0.0 |  |  |
| 150 | *T. palliatum_*9 | 9.4 | 9.4 | 15.6 | 15.6 | 15.3 | 16.0 | 9.4 | 9.4 | 9.7 | 9.7 | 14.6 | 17.0 | 17.0 | 17.0 | 16.7 | 16.7 | 16.7 | 17.4 | 18.1 | 16.7 | 11.8 | 10.8 | 10.4 | 11.5 | 11.1 | 11.1 | 10.4 | 10.4 | 11.1 | 11.1 | 11.1 | 16.0 | 16.0 | 16.7 | 12.5 | 12.5 | 8.4 | 15.7 | 13.2 | 13.2 | 12.9 | 0.7 | 1.0 | 0.3 | 1.4 | 0.3 | 0.7 | 0.4 | 0.3 |  |
| 151 | *T. palliatum_*10 | 9.4 | 9.4 | 15.3 | 15.3 | 14.9 | 15.6 | 9.4 | 9.4 | 9.7 | 9.7 | 14.2 | 17.0 | 17.0 | 17.0 | 16.7 | 16.7 | 16.7 | 17.4 | 18.5 | 16.7 | 11.8 | 10.8 | 10.4 | 11.5 | 11.1 | 11.1 | 10.4 | 10.4 | 11.1 | 11.1 | 11.1 | 16.0 | 16.0 | 16.7 | 12.5 | 12.5 | 8.4 | 15.7 | 13.2 | 13.2 | 12.9 | 0.7 | 1.0 | 0.3 | 1.4 | 0.3 | 0.7 | 0.8 | 1.0 | 0.7 |
| 152 | *T. palliatum_*11 | 9.4 | 9.4 | 15.6 | 15.6 | 15.3 | 16.0 | 9.4 | 9.4 | 9.7 | 9.7 | 14.6 | 17.0 | 17.0 | 17.0 | 16.7 | 16.7 | 16.7 | 17.4 | 18.1 | 16.7 | 11.8 | 10.8 | 10.4 | 11.5 | 11.1 | 11.1 | 10.4 | 10.4 | 11.1 | 11.1 | 11.1 | 16.0 | 16.0 | 16.7 | 12.5 | 12.5 | 8.4 | 15.7 | 13.2 | 13.2 | 12.9 | 0.7 | 1.0 | 0.3 | 1.4 | 0.3 | 0.7 | 0.4 | 0.3 | 0.0 |
| 153 | *T. petilum_*11 | 11.5 | 11.5 | 16.0 | 16.0 | 15.3 | 15.6 | 13.2 | 13.2 | 14.2 | 14.2 | 14.2 | 13.5 | 13.5 | 13.9 | 13.5 | 13.5 | 13.5 | 14.2 | 15.7 | 14.2 | 15.6 | 14.6 | 14.2 | 14.9 | 14.2 | 14.2 | 13.9 | 13.9 | 13.5 | 13.5 | 13.5 | 11.1 | 11.1 | 10.8 | 11.8 | 11.8 | 7.4 | 16.7 | 13.9 | 13.9 | 13.6 | 14.6 | 14.9 | 14.2 | 14.6 | 14.2 | 13.9 | 15.1 | 14.2 | 14.6 |
| 154 | *T. phrynoderma_*1 | 15.7 | 15.7 | 18.1 | 18.1 | 17.7 | 18.1 | 13.8 | 13.8 | 16.5 | 16.5 | 11.8 | 13.8 | 14.2 | 14.2 | 13.8 | 13.8 | 13.8 | 14.6 | 14.8 | 14.6 | 16.1 | 16.1 | 15.7 | 16.5 | 16.9 | 16.9 | 16.1 | 16.1 | 11.8 | 11.8 | 11.8 | 13.4 | 14.6 | 14.2 | 9.4 | 9.4 | 8.9 | 14.2 | 11.8 | 11.8 | 11.8 | 15.4 | 15.7 | 15.0 | 15.4 | 15.0 | 14.6 | 15.3 | 14.6 | 15.0 |
| 155 | *T. phrynoderma_*2 | 15.0 | 15.0 | 17.3 | 17.3 | 16.9 | 17.3 | 12.6 | 12.6 | 15.0 | 15.0 | 10.6 | 13.4 | 13.4 | 13.8 | 13.4 | 13.4 | 13.4 | 14.2 | 14.3 | 14.2 | 14.6 | 14.6 | 14.2 | 15.0 | 15.4 | 15.4 | 14.6 | 14.6 | 10.2 | 10.2 | 10.2 | 12.6 | 13.8 | 13.4 | 7.9 | 7.9 | 7.9 | 14.2 | 11.8 | 11.8 | 11.8 | 13.8 | 14.2 | 13.4 | 13.8 | 13.4 | 13.0 | 13.6 | 13.0 | 13.4 |
| 156 | *T. pyaukkya_*1 | 11.1 | 11.1 | 17.4 | 17.4 | 16.7 | 17.0 | 12.5 | 12.5 | 13.2 | 13.2 | 12.2 | 15.3 | 15.3 | 15.6 | 15.3 | 15.3 | 15.3 | 16.0 | 16.5 | 15.3 | 14.9 | 14.2 | 13.5 | 14.2 | 14.9 | 14.9 | 13.9 | 13.9 | 11.8 | 11.8 | 11.8 | 9.7 | 10.8 | 11.1 | 9.4 | 9.4 | 5.6 | 16.4 | 13.5 | 13.5 | 13.2 | 12.2 | 12.5 | 11.8 | 12.2 | 11.8 | 11.5 | 11.9 | 11.8 | 12.2 |
| 157 | *T. pyaukkya_*2 | 10.8 | 10.8 | 17.0 | 17.0 | 16.3 | 16.7 | 12.2 | 12.2 | 13.2 | 13.2 | 11.5 | 15.6 | 15.6 | 16.0 | 15.6 | 15.6 | 15.6 | 16.3 | 16.9 | 15.6 | 14.9 | 14.2 | 13.5 | 14.2 | 14.9 | 14.9 | 13.9 | 13.9 | 11.8 | 11.8 | 11.8 | 9.7 | 11.1 | 11.5 | 9.7 | 9.7 | 6.0 | 16.0 | 13.2 | 13.2 | 12.9 | 12.2 | 12.5 | 11.8 | 12.2 | 11.8 | 11.5 | 11.9 | 11.8 | 12.2 |
| 158 | *T. pyaukkya_*3 | 10.1 | 10.1 | 17.0 | 17.0 | 16.3 | 16.7 | 12.2 | 12.2 | 12.5 | 12.5 | 11.5 | 14.9 | 14.9 | 15.3 | 14.9 | 14.9 | 14.9 | 15.6 | 16.1 | 14.9 | 14.2 | 13.5 | 12.8 | 14.2 | 14.2 | 14.2 | 13.2 | 13.2 | 12.5 | 12.5 | 12.5 | 9.7 | 10.4 | 11.5 | 9.7 | 9.7 | 6.0 | 16.0 | 13.2 | 13.2 | 12.9 | 11.5 | 11.8 | 11.1 | 11.5 | 11.1 | 10.8 | 11.1 | 11.1 | 11.5 |
| 159 | *T. pyaukkya_*4 | 10.1 | 10.1 | 17.0 | 17.0 | 17.0 | 17.4 | 12.5 | 12.5 | 12.5 | 12.5 | 10.8 | 13.9 | 13.9 | 14.2 | 13.9 | 13.9 | 13.9 | 14.6 | 15.4 | 14.6 | 14.6 | 13.5 | 13.2 | 14.6 | 13.9 | 13.9 | 13.5 | 13.5 | 11.8 | 11.8 | 11.8 | 8.0 | 8.7 | 9.7 | 9.7 | 9.7 | 6.5 | 12.9 | 10.1 | 10.1 | 9.8 | 13.5 | 13.9 | 13.2 | 13.5 | 13.2 | 12.8 | 13.9 | 13.2 | 13.5 |
| 160 | *T. pyaukkya_*5 | 10.1 | 10.1 | 17.0 | 17.0 | 17.0 | 17.4 | 12.5 | 12.5 | 12.5 | 12.5 | 10.8 | 13.9 | 13.9 | 14.2 | 13.9 | 13.9 | 13.9 | 14.6 | 15.4 | 14.6 | 14.6 | 13.5 | 13.2 | 14.6 | 13.9 | 13.9 | 13.5 | 13.5 | 11.8 | 11.8 | 11.8 | 8.0 | 8.7 | 9.7 | 9.7 | 9.7 | 6.5 | 12.9 | 10.1 | 10.1 | 9.8 | 13.5 | 13.9 | 13.2 | 13.5 | 13.2 | 12.8 | 13.9 | 13.2 | 13.5 |
| 161 | *T. rhododiscus_*1 | 2.8 | 2.8 | 15.6 | 15.6 | 15.6 | 16.0 | 9.4 | 9.4 | 10.8 | 10.8 | 12.5 | 14.6 | 15.3 | 14.6 | 14.6 | 14.6 | 14.6 | 14.9 | 16.1 | 15.6 | 13.5 | 12.5 | 11.5 | 12.8 | 11.8 | 11.8 | 11.8 | 11.8 | 11.5 | 11.5 | 11.5 | 13.9 | 14.2 | 13.9 | 9.4 | 9.4 | 6.5 | 15.7 | 12.5 | 12.5 | 12.2 | 10.4 | 10.8 | 10.1 | 11.1 | 10.1 | 10.4 | 11.5 | 10.8 | 10.4 |
| 162 | *T. rhododiscus*10 | 3.1 | 3.1 | 15.6 | 15.6 | 16.3 | 16.0 | 9.7 | 9.7 | 11.5 | 11.5 | 12.5 | 14.6 | 15.3 | 14.6 | 14.6 | 14.6 | 14.6 | 14.9 | 16.1 | 15.6 | 13.2 | 12.2 | 11.1 | 12.5 | 12.2 | 12.2 | 11.5 | 11.5 | 10.8 | 10.8 | 10.8 | 13.9 | 14.2 | 13.9 | 9.4 | 9.4 | 6.5 | 15.3 | 12.2 | 12.2 | 11.8 | 10.1 | 10.4 | 9.7 | 10.8 | 9.7 | 10.1 | 11.1 | 10.4 | 10.1 |
| 163 | *T. rhododiscus_*2 | 2.8 | 2.8 | 15.6 | 15.6 | 15.6 | 16.0 | 9.4 | 9.4 | 10.8 | 10.8 | 12.5 | 14.6 | 15.3 | 14.6 | 14.6 | 14.6 | 14.6 | 14.9 | 16.1 | 15.6 | 13.5 | 12.5 | 11.5 | 12.8 | 11.8 | 11.8 | 11.8 | 11.8 | 11.5 | 11.5 | 11.5 | 13.9 | 14.2 | 13.9 | 9.4 | 9.4 | 6.5 | 15.7 | 12.5 | 12.5 | 12.2 | 10.4 | 10.8 | 10.1 | 11.1 | 10.1 | 10.4 | 11.5 | 10.8 | 10.4 |
| 164 | *T. rhododiscu_s*3 | 2.8 | 2.8 | 15.6 | 15.6 | 15.6 | 16.0 | 9.4 | 9.4 | 10.8 | 10.8 | 12.5 | 14.6 | 15.3 | 14.6 | 14.6 | 14.6 | 14.6 | 14.9 | 16.1 | 15.6 | 13.5 | 12.5 | 11.5 | 12.8 | 11.8 | 11.8 | 11.8 | 11.8 | 11.5 | 11.5 | 11.5 | 13.9 | 14.2 | 13.9 | 9.4 | 9.4 | 6.5 | 15.7 | 12.5 | 12.5 | 12.2 | 10.4 | 10.8 | 10.1 | 11.1 | 10.1 | 10.4 | 11.5 | 10.8 | 10.4 |
| 165 | *T. rhododiscus_*4 | 2.8 | 2.8 | 15.6 | 15.6 | 15.6 | 16.0 | 9.4 | 9.4 | 10.8 | 10.8 | 12.5 | 14.6 | 15.3 | 14.6 | 14.6 | 14.6 | 14.6 | 14.9 | 16.1 | 15.6 | 13.5 | 12.5 | 11.5 | 12.8 | 11.8 | 11.8 | 11.8 | 11.8 | 11.5 | 11.5 | 11.5 | 13.9 | 14.2 | 13.9 | 9.4 | 9.4 | 6.5 | 15.7 | 12.5 | 12.5 | 12.2 | 10.4 | 10.8 | 10.1 | 11.1 | 10.1 | 10.4 | 11.5 | 10.8 | 10.4 |
| 166 | *T. rhododiscus_*5 | 2.8 | 2.8 | 15.6 | 15.6 | 15.6 | 16.0 | 9.4 | 9.4 | 10.8 | 10.8 | 12.5 | 14.6 | 15.3 | 14.6 | 14.6 | 14.6 | 14.6 | 14.9 | 16.1 | 15.6 | 13.5 | 12.5 | 11.5 | 12.8 | 11.8 | 11.8 | 11.8 | 11.8 | 11.5 | 11.5 | 11.5 | 13.9 | 14.2 | 13.9 | 9.4 | 9.4 | 6.5 | 15.7 | 12.5 | 12.5 | 12.2 | 10.4 | 10.8 | 10.1 | 11.1 | 10.1 | 10.4 | 11.5 | 10.8 | 10.4 |
| 167 | *T. rhododiscus_*6 | 2.8 | 2.8 | 15.6 | 15.6 | 15.6 | 16.0 | 9.4 | 9.4 | 10.8 | 10.8 | 12.5 | 14.6 | 15.3 | 14.6 | 14.6 | 14.6 | 14.6 | 14.9 | 16.1 | 15.6 | 13.5 | 12.5 | 11.5 | 12.8 | 11.8 | 11.8 | 11.8 | 11.8 | 11.5 | 11.5 | 11.5 | 13.9 | 14.2 | 13.9 | 9.4 | 9.4 | 6.5 | 15.7 | 12.5 | 12.5 | 12.2 | 10.4 | 10.8 | 10.1 | 11.1 | 10.1 | 10.4 | 11.5 | 10.8 | 10.4 |
| 168 | *T. rhododiscus_*7 | 2.8 | 2.8 | 15.6 | 15.6 | 15.6 | 16.0 | 9.4 | 9.4 | 10.8 | 10.8 | 12.5 | 14.6 | 15.3 | 14.6 | 14.6 | 14.6 | 14.6 | 14.9 | 16.1 | 15.6 | 13.5 | 12.5 | 11.5 | 12.8 | 11.8 | 11.8 | 11.8 | 11.8 | 11.5 | 11.5 | 11.5 | 13.9 | 14.2 | 13.9 | 9.4 | 9.4 | 6.5 | 15.7 | 12.5 | 12.5 | 12.2 | 10.4 | 10.8 | 10.1 | 11.1 | 10.1 | 10.4 | 11.5 | 10.8 | 10.4 |
| 169 | *T. rhododiscus_*8 | 2.8 | 2.8 | 15.6 | 15.6 | 15.6 | 16.0 | 9.4 | 9.4 | 10.8 | 10.8 | 12.5 | 14.6 | 15.3 | 14.6 | 14.6 | 14.6 | 14.6 | 14.9 | 16.1 | 15.6 | 13.5 | 12.5 | 11.5 | 12.8 | 11.8 | 11.8 | 11.8 | 11.8 | 11.5 | 11.5 | 11.5 | 13.9 | 14.2 | 13.9 | 9.4 | 9.4 | 6.5 | 15.7 | 12.5 | 12.5 | 12.2 | 10.4 | 10.8 | 10.1 | 11.1 | 10.1 | 10.4 | 11.5 | 10.8 | 10.4 |
| 170 | *T. rhododiscus_*9 | 2.8 | 2.8 | 16.0 | 16.0 | 16.0 | 16.3 | 9.7 | 9.7 | 11.5 | 11.5 | 12.2 | 14.2 | 14.9 | 14.2 | 14.2 | 14.2 | 14.2 | 14.6 | 15.7 | 15.3 | 12.8 | 11.8 | 10.8 | 12.2 | 11.8 | 11.8 | 11.1 | 11.1 | 10.4 | 10.4 | 10.4 | 14.2 | 14.6 | 14.2 | 9.7 | 9.7 | 6.0 | 15.7 | 12.5 | 12.5 | 12.2 | 9.7 | 10.1 | 9.4 | 10.4 | 9.4 | 9.7 | 10.7 | 10.1 | 9.7 |
| 171 | *T. ryabovi_*1 | 11.5 | 11.5 | 18.4 | 18.4 | 18.4 | 18.8 | 11.8 | 11.8 | 12.8 | 12.8 | 9.4 | 10.4 | 10.4 | 11.1 | 11.5 | 11.5 | 11.5 | 11.5 | 11.4 | 12.2 | 14.6 | 14.9 | 14.6 | 15.3 | 14.6 | 14.6 | 14.9 | 14.9 | 11.8 | 11.8 | 11.8 | 13.2 | 13.5 | 13.5 | 4.9 | 4.9 | 3.3 | 14.3 | 11.8 | 11.8 | 11.8 | 13.5 | 13.9 | 13.2 | 13.5 | 13.2 | 12.8 | 14.3 | 13.2 | 13.5 |
| 172 | *T. ryabovi_*2 | 14.6 | 14.6 | 21.2 | 21.2 | 21.2 | 21.5 | 14.9 | 14.9 | 15.3 | 15.3 | 12.5 | 12.2 | 12.2 | 12.8 | 13.2 | 13.2 | 13.2 | 13.2 | 12.6 | 13.9 | 17.7 | 18.1 | 17.7 | 18.4 | 17.7 | 17.7 | 18.1 | 18.1 | 14.6 | 14.6 | 14.6 | 15.6 | 16.0 | 16.0 | 8.0 | 8.0 | 7.4 | 17.4 | 14.9 | 14.9 | 15.0 | 16.7 | 17.0 | 16.3 | 16.7 | 16.3 | 16.0 | 16.3 | 16.3 | 16.7 |
| 173 | *T. ryabovi_*3 | 14.6 | 14.6 | 21.2 | 21.2 | 21.2 | 21.5 | 14.9 | 14.9 | 15.3 | 15.3 | 12.5 | 12.2 | 12.2 | 12.8 | 13.2 | 13.2 | 13.2 | 13.2 | 12.6 | 13.9 | 17.7 | 18.1 | 17.7 | 18.4 | 17.7 | 17.7 | 18.1 | 18.1 | 14.6 | 14.6 | 14.6 | 15.6 | 16.0 | 16.0 | 8.0 | 8.0 | 7.4 | 17.4 | 14.9 | 14.9 | 15.0 | 16.7 | 17.0 | 16.3 | 16.7 | 16.3 | 16.0 | 16.3 | 16.3 | 16.7 |
| 174 | *T. stellatum_*1 | 15.6 | 15.6 | 9.4 | 9.4 | 9.7 | 9.0 | 15.6 | 15.6 | 17.4 | 17.4 | 19.4 | 21.5 | 21.5 | 22.2 | 22.2 | 22.2 | 22.2 | 22.6 | 24.4 | 23.3 | 17.7 | 18.1 | 16.3 | 16.7 | 16.7 | 16.7 | 16.3 | 16.3 | 18.4 | 18.4 | 18.4 | 19.1 | 18.4 | 19.1 | 18.4 | 18.4 | 13.0 | 19.5 | 17.4 | 17.4 | 17.4 | 16.0 | 15.6 | 15.6 | 16.0 | 15.6 | 16.0 | 17.1 | 16.3 | 16.0 |
| 175 | *T. stellatum_*2 | 15.6 | 15.6 | 9.4 | 9.4 | 9.7 | 9.0 | 15.6 | 15.6 | 17.4 | 17.4 | 19.4 | 21.5 | 21.5 | 22.2 | 22.2 | 22.2 | 22.2 | 22.6 | 24.4 | 23.3 | 17.7 | 18.1 | 16.3 | 16.7 | 16.7 | 16.7 | 16.3 | 16.3 | 18.4 | 18.4 | 18.4 | 19.1 | 18.4 | 19.1 | 18.4 | 18.4 | 13.0 | 19.5 | 17.4 | 17.4 | 17.4 | 16.0 | 15.6 | 15.6 | 16.0 | 15.6 | 16.0 | 17.1 | 16.3 | 16.0 |
| 176 | *T. truongsonense_*1 | 12.8 | 12.8 | 18.4 | 18.4 | 18.1 | 18.8 | 12.5 | 12.5 | 13.2 | 13.2 | 9.0 | 12.2 | 13.5 | 12.2 | 12.5 | 12.5 | 12.5 | 12.5 | 13.0 | 13.2 | 13.9 | 13.2 | 12.5 | 13.2 | 12.2 | 12.2 | 12.5 | 12.5 | 14.9 | 14.9 | 14.9 | 13.9 | 14.6 | 14.9 | 12.5 | 12.5 | 9.3 | 11.8 | 8.3 | 8.3 | 8.0 | 13.9 | 14.2 | 13.5 | 13.9 | 13.5 | 13.2 | 14.3 | 13.5 | 13.9 |
| 177 | *T. truongsonense_*2 | 12.5 | 12.5 | 18.1 | 18.1 | 17.7 | 18.4 | 12.2 | 12.2 | 13.9 | 13.9 | 10.1 | 12.5 | 13.9 | 12.5 | 12.8 | 12.8 | 12.8 | 12.8 | 13.4 | 13.5 | 14.6 | 13.9 | 13.2 | 13.9 | 12.8 | 12.8 | 13.2 | 13.2 | 14.2 | 14.2 | 14.2 | 15.3 | 16.0 | 16.3 | 12.8 | 12.8 | 9.8 | 12.2 | 8.7 | 8.7 | 8.4 | 13.9 | 14.2 | 13.5 | 14.6 | 13.5 | 13.9 | 15.1 | 14.2 | 13.9 |
| 178 | *T. truongsonense_*3 | 12.5 | 12.5 | 18.1 | 18.1 | 17.7 | 18.4 | 12.2 | 12.2 | 13.9 | 13.9 | 10.1 | 12.5 | 13.9 | 12.5 | 12.8 | 12.8 | 12.8 | 12.8 | 13.4 | 13.5 | 14.6 | 13.9 | 13.2 | 13.9 | 12.8 | 12.8 | 13.2 | 13.2 | 14.2 | 14.2 | 14.2 | 15.3 | 16.0 | 16.3 | 12.8 | 12.8 | 9.8 | 12.2 | 8.7 | 8.7 | 8.4 | 13.9 | 14.2 | 13.5 | 14.6 | 13.5 | 13.9 | 15.1 | 14.2 | 13.9 |
| 179 | *T. truongsonense_*4 | 13.9 | 13.9 | 18.4 | 18.4 | 18.1 | 18.8 | 11.8 | 11.8 | 11.5 | 11.5 | 8.7 | 11.8 | 11.8 | 12.5 | 12.8 | 12.8 | 12.8 | 12.8 | 13.8 | 13.5 | 12.8 | 13.2 | 12.2 | 13.5 | 12.2 | 12.2 | 12.5 | 12.5 | 14.2 | 14.2 | 14.2 | 14.9 | 15.6 | 16.0 | 11.5 | 11.5 | 7.4 | 12.5 | 9.7 | 9.7 | 9.8 | 13.9 | 14.2 | 13.5 | 13.9 | 13.5 | 13.2 | 14.7 | 13.5 | 13.9 |
| 180 | *T. truongsonense_*5 | 15.1 | 15.1 | 19.6 | 19.6 | 19.2 | 19.9 | 12.9 | 12.9 | 14.8 | 14.8 | 11.1 | 13.7 | 14.4 | 14.4 | 14.4 | 14.4 | 14.4 | 14.8 | 15.0 | 15.5 | 15.9 | 15.5 | 14.4 | 15.1 | 13.7 | 13.7 | 14.0 | 14.0 | 15.1 | 15.1 | 15.1 | 15.5 | 16.6 | 16.6 | 14.0 | 14.0 | 10.6 | 13.0 | 9.2 | 9.2 | 9.3 | 17.3 | 17.7 | 17.0 | 17.3 | 17.0 | 16.6 | 17.5 | 16.6 | 17.0 |
| 181 | *T. truongsonense_*6 | 13.2 | 13.2 | 17.0 | 17.0 | 16.7 | 17.4 | 11.5 | 11.5 | 12.2 | 12.2 | 9.7 | 12.5 | 12.5 | 13.2 | 13.5 | 13.5 | 13.5 | 13.5 | 14.6 | 14.2 | 14.2 | 13.9 | 12.8 | 14.2 | 12.8 | 12.8 | 13.2 | 13.2 | 15.6 | 15.6 | 15.6 | 13.9 | 14.2 | 14.6 | 12.2 | 12.2 | 8.8 | 12.5 | 9.7 | 9.7 | 9.8 | 14.6 | 14.9 | 14.2 | 14.6 | 14.2 | 13.9 | 15.5 | 14.2 | 14.6 |
| 182 | *T. truongsonense_*7 | 13.2 | 13.2 | 17.0 | 17.0 | 16.7 | 17.4 | 11.5 | 11.5 | 12.2 | 12.2 | 9.7 | 12.5 | 12.5 | 13.2 | 13.5 | 13.5 | 13.5 | 13.5 | 14.6 | 14.2 | 14.2 | 13.9 | 12.8 | 14.2 | 12.8 | 12.8 | 13.2 | 13.2 | 15.6 | 15.6 | 15.6 | 13.9 | 14.2 | 14.6 | 12.2 | 12.2 | 8.8 | 12.5 | 9.7 | 9.7 | 9.8 | 14.6 | 14.9 | 14.2 | 14.6 | 14.2 | 13.9 | 15.5 | 14.2 | 14.6 |
| 183 | *T. vietnamense_*1 | 13.9 | 13.9 | 11.1 | 11.1 | 11.8 | 11.8 | 11.5 | 11.5 | 14.6 | 14.6 | 17.4 | 19.1 | 18.8 | 19.4 | 19.8 | 19.8 | 19.8 | 19.8 | 20.9 | 19.8 | 15.6 | 15.3 | 14.9 | 14.6 | 13.9 | 13.9 | 14.2 | 14.2 | 16.0 | 16.0 | 16.0 | 16.7 | 16.3 | 17.0 | 16.0 | 16.0 | 10.2 | 17.8 | 15.6 | 15.6 | 15.7 | 14.6 | 14.2 | 14.2 | 14.6 | 14.2 | 14.6 | 15.1 | 14.9 | 14.6 |
| 184 | *T. vietnamense_*2 | 14.2 | 14.2 | 10.8 | 10.8 | 11.5 | 11.5 | 11.8 | 11.8 | 14.9 | 14.9 | 17.7 | 18.8 | 18.4 | 19.1 | 19.4 | 19.4 | 19.4 | 19.4 | 20.5 | 19.4 | 16.0 | 15.6 | 15.3 | 14.9 | 14.2 | 14.2 | 14.6 | 14.6 | 16.3 | 16.3 | 16.3 | 16.7 | 16.3 | 17.0 | 16.7 | 16.7 | 11.2 | 18.1 | 16.0 | 16.0 | 16.0 | 15.3 | 14.9 | 14.9 | 15.3 | 14.9 | 15.3 | 15.9 | 15.6 | 15.3 |
| 185 | *T. vietnamense_*3 | 14.9 | 14.9 | 10.8 | 10.8 | 11.5 | 11.5 | 11.5 | 11.5 | 15.3 | 15.3 | 17.7 | 20.1 | 19.8 | 20.5 | 20.8 | 20.8 | 20.8 | 20.8 | 22.0 | 20.8 | 16.3 | 16.0 | 15.6 | 14.9 | 14.6 | 14.6 | 14.9 | 14.9 | 16.7 | 16.7 | 16.7 | 17.0 | 17.4 | 17.4 | 16.3 | 16.3 | 11.6 | 18.8 | 16.7 | 16.7 | 16.7 | 15.6 | 15.3 | 15.3 | 15.6 | 15.3 | 15.6 | 16.3 | 16.0 | 15.6 |
| 186 | *T. vietnamense_*4 | 15.0 | 15.0 | 10.8 | 10.8 | 11.5 | 11.5 | 11.5 | 11.5 | 15.3 | 15.3 | 17.4 | 19.9 | 19.5 | 20.2 | 20.6 | 20.6 | 20.6 | 20.6 | 21.7 | 20.6 | 16.0 | 16.0 | 15.7 | 15.0 | 14.6 | 14.6 | 15.0 | 15.0 | 16.4 | 16.4 | 16.4 | 17.1 | 17.4 | 17.4 | 16.4 | 16.4 | 11.6 | 18.5 | 16.4 | 16.4 | 16.4 | 15.7 | 15.3 | 15.3 | 15.7 | 15.3 | 15.7 | 16.3 | 16.0 | 15.7 |
| 187 | *T. vietnamense_*5 | 14.9 | 14.9 | 10.1 | 10.1 | 10.8 | 10.8 | 11.5 | 11.5 | 15.3 | 15.3 | 16.7 | 19.1 | 18.8 | 19.4 | 19.8 | 19.8 | 19.8 | 19.8 | 20.9 | 19.8 | 17.0 | 16.7 | 15.6 | 14.6 | 15.3 | 15.3 | 14.9 | 14.9 | 16.0 | 16.0 | 16.0 | 16.3 | 16.7 | 16.7 | 16.3 | 16.3 | 11.2 | 17.8 | 15.6 | 15.6 | 15.7 | 15.6 | 15.3 | 15.3 | 15.6 | 15.3 | 15.6 | 16.3 | 16.0 | 15.6 |
| 188 | *T. vietnamense_*6 | 14.9 | 14.9 | 10.1 | 10.1 | 10.8 | 10.8 | 11.5 | 11.5 | 15.3 | 15.3 | 16.7 | 19.1 | 18.8 | 19.4 | 19.8 | 19.8 | 19.8 | 19.8 | 20.9 | 19.8 | 17.0 | 16.7 | 15.6 | 14.6 | 15.3 | 15.3 | 14.9 | 14.9 | 16.0 | 16.0 | 16.0 | 16.3 | 16.7 | 16.7 | 16.3 | 16.3 | 11.2 | 17.8 | 15.6 | 15.6 | 15.7 | 15.6 | 15.3 | 15.3 | 15.6 | 15.3 | 15.6 | 16.3 | 16.0 | 15.6 |
| 189 | *T. vietnamense_*7 | 14.9 | 14.9 | 10.1 | 10.1 | 10.8 | 10.8 | 11.5 | 11.5 | 15.3 | 15.3 | 16.7 | 19.1 | 18.8 | 19.4 | 19.8 | 19.8 | 19.8 | 19.8 | 20.9 | 19.8 | 17.0 | 16.7 | 15.6 | 14.6 | 15.3 | 15.3 | 14.9 | 14.9 | 16.0 | 16.0 | 16.0 | 16.3 | 16.7 | 16.7 | 16.3 | 16.3 | 11.2 | 17.8 | 15.6 | 15.6 | 15.7 | 15.6 | 15.3 | 15.3 | 15.6 | 15.3 | 15.6 | 16.3 | 16.0 | 15.6 |
| 190 | *T. vietnamense_*8 | 15.3 | 15.3 | 10.4 | 10.4 | 11.1 | 11.1 | 11.8 | 11.8 | 14.9 | 14.9 | 16.7 | 19.4 | 19.1 | 19.8 | 20.1 | 20.1 | 20.1 | 20.1 | 21.3 | 20.1 | 16.7 | 16.3 | 15.3 | 14.2 | 14.9 | 14.9 | 14.6 | 14.6 | 15.6 | 15.6 | 15.6 | 16.3 | 17.0 | 17.0 | 16.7 | 16.7 | 11.2 | 18.1 | 16.0 | 16.0 | 16.0 | 15.3 | 14.9 | 14.9 | 15.3 | 14.9 | 15.3 | 15.9 | 15.6 | 15.3 |
| 191 | *T. vietnamense_*9 | 15.3 | 15.3 | 10.4 | 10.4 | 11.1 | 11.1 | 11.8 | 11.8 | 14.9 | 14.9 | 16.7 | 19.4 | 19.1 | 19.8 | 20.1 | 20.1 | 20.1 | 20.1 | 21.3 | 20.1 | 16.7 | 16.3 | 15.3 | 14.2 | 14.9 | 14.9 | 14.6 | 14.6 | 15.6 | 15.6 | 15.6 | 16.3 | 17.0 | 17.0 | 16.7 | 16.7 | 11.2 | 18.1 | 16.0 | 16.0 | 16.0 | 15.3 | 14.9 | 14.9 | 15.3 | 14.9 | 15.3 | 15.9 | 15.6 | 15.3 |
| 192 | *T. vietnamense_*10 | 15.6 | 15.6 | 10.8 | 10.8 | 11.5 | 11.5 | 12.2 | 12.2 | 16.0 | 16.0 | 17.4 | 19.8 | 19.4 | 20.1 | 20.5 | 20.5 | 20.5 | 20.5 | 20.9 | 20.5 | 17.7 | 17.4 | 16.3 | 15.3 | 16.0 | 16.0 | 15.6 | 15.6 | 16.7 | 16.7 | 16.7 | 17.0 | 17.4 | 16.7 | 17.0 | 17.0 | 12.1 | 18.5 | 16.3 | 16.3 | 16.4 | 16.3 | 16.0 | 16.0 | 16.3 | 16.0 | 16.3 | 16.3 | 16.7 | 16.3 |
| 193 | *T. vietnamense_*11 | 14.9 | 14.9 | 10.1 | 10.1 | 10.8 | 10.8 | 11.5 | 11.5 | 15.3 | 15.3 | 16.7 | 19.1 | 18.8 | 19.4 | 19.8 | 19.8 | 19.8 | 19.8 | 20.9 | 19.8 | 17.0 | 16.7 | 15.6 | 14.6 | 15.3 | 15.3 | 14.9 | 14.9 | 16.0 | 16.0 | 16.0 | 16.3 | 16.7 | 16.7 | 16.3 | 16.3 | 11.2 | 17.8 | 15.6 | 15.6 | 15.7 | 15.6 | 15.3 | 15.3 | 15.6 | 15.3 | 15.6 | 16.3 | 16.0 | 15.6 |
| 194 | *T. vietnamense_*12 | 14.9 | 14.9 | 10.1 | 10.1 | 10.8 | 10.8 | 11.5 | 11.5 | 15.3 | 15.3 | 16.7 | 19.1 | 18.8 | 19.4 | 19.8 | 19.8 | 19.8 | 19.8 | 20.9 | 19.8 | 17.0 | 16.7 | 15.6 | 14.6 | 15.3 | 15.3 | 14.9 | 14.9 | 16.0 | 16.0 | 16.0 | 16.3 | 16.7 | 16.7 | 16.3 | 16.3 | 11.2 | 17.8 | 15.6 | 15.6 | 15.7 | 15.6 | 15.3 | 15.3 | 15.6 | 15.3 | 15.6 | 16.3 | 16.0 | 15.6 |
| 195 | *T. vietnamense_*13 | 14.9 | 14.9 | 10.1 | 10.1 | 10.8 | 10.8 | 11.5 | 11.5 | 15.3 | 15.3 | 16.7 | 19.1 | 18.8 | 19.4 | 19.8 | 19.8 | 19.8 | 19.8 | 20.9 | 19.8 | 17.0 | 16.7 | 15.6 | 14.6 | 15.3 | 15.3 | 14.9 | 14.9 | 16.0 | 16.0 | 16.0 | 16.3 | 16.7 | 16.7 | 16.3 | 16.3 | 11.2 | 17.8 | 15.6 | 15.6 | 15.7 | 15.6 | 15.3 | 15.3 | 15.6 | 15.3 | 15.6 | 16.3 | 16.0 | 15.6 |

Next page

Continue to

| ID | Species | 151 | 152 | 153 | 154 | 155 | 156 | 157 | 158 | 159 | 160 | 161 | 162 | 163 | 164 | 165 | 166 | 167 | 168 | 169 | 170 | 171 | 172 | 173 | 174 | 175 | 176 | 177 | 178 | 179 | 180 | 181 | 182 | 183 | 184 | 185 | 186 | 187 | 188 | 189 | 190 | 191 | 192 | 193 | 194 | 195 |
| --- | --- | --- | --- | --- | --- | --- | --- | --- | --- | --- | --- | --- | --- | --- | --- | --- | --- | --- | --- | --- | --- | --- | --- | --- | --- | --- | --- | --- | --- | --- | --- | --- | --- | --- | --- | --- | --- | --- | --- | --- | --- | --- | --- | --- | --- | --- |
| 1 | *T. albopunctatum_*1 |  |  |  |  |  |  |  |  |  |  |  |  |  |  |  |  |  |  |  |  |  |  |  |  |  |  |  |  |  |  |  |  |  |  |  |  |  |  |  |  |  |  |  |  |  |
| 2 | *T. albopunctatum_*2 |  |  |  |  |  |  |  |  |  |  |  |  |  |  |  |  |  |  |  |  |  |  |  |  |  |  |  |  |  |  |  |  |  |  |  |  |  |  |  |  |  |  |  |  |  |
| 3 | *T. albopunctatum_*3 |  |  |  |  |  |  |  |  |  |  |  |  |  |  |  |  |  |  |  |  |  |  |  |  |  |  |  |  |  |  |  |  |  |  |  |  |  |  |  |  |  |  |  |  |  |
| 4 | *T. albopunctatum_*4 |  |  |  |  |  |  |  |  |  |  |  |  |  |  |  |  |  |  |  |  |  |  |  |  |  |  |  |  |  |  |  |  |  |  |  |  |  |  |  |  |  |  |  |  |  |
| 5 | *T. albopunctatum_*5 |  |  |  |  |  |  |  |  |  |  |  |  |  |  |  |  |  |  |  |  |  |  |  |  |  |  |  |  |  |  |  |  |  |  |  |  |  |  |  |  |  |  |  |  |  |
| 6 | *T. albopunctatum_*6 |  |  |  |  |  |  |  |  |  |  |  |  |  |  |  |  |  |  |  |  |  |  |  |  |  |  |  |  |  |  |  |  |  |  |  |  |  |  |  |  |  |  |  |  |  |
| 7 | *T. albopunctatum_*7 |  |  |  |  |  |  |  |  |  |  |  |  |  |  |  |  |  |  |  |  |  |  |  |  |  |  |  |  |  |  |  |  |  |  |  |  |  |  |  |  |  |  |  |  |  |
| 8 | *T. albopunctatum_*8 |  |  |  |  |  |  |  |  |  |  |  |  |  |  |  |  |  |  |  |  |  |  |  |  |  |  |  |  |  |  |  |  |  |  |  |  |  |  |  |  |  |  |  |  |  |
| 9 | *T. albopunctatum_*9 |  |  |  |  |  |  |  |  |  |  |  |  |  |  |  |  |  |  |  |  |  |  |  |  |  |  |  |  |  |  |  |  |  |  |  |  |  |  |  |  |  |  |  |  |  |
| 10 | *T. albopunctatum_*10 |  |  |  |  |  |  |  |  |  |  |  |  |  |  |  |  |  |  |  |  |  |  |  |  |  |  |  |  |  |  |  |  |  |  |  |  |  |  |  |  |  |  |  |  |  |
| 11 | *T. albopunctatum_*11 |  |  |  |  |  |  |  |  |  |  |  |  |  |  |  |  |  |  |  |  |  |  |  |  |  |  |  |  |  |  |  |  |  |  |  |  |  |  |  |  |  |  |  |  |  |
| 12 | *T. albopunctatum_*12 |  |  |  |  |  |  |  |  |  |  |  |  |  |  |  |  |  |  |  |  |  |  |  |  |  |  |  |  |  |  |  |  |  |  |  |  |  |  |  |  |  |  |  |  |  |
| 13 | *T. albopunctatum_*13 |  |  |  |  |  |  |  |  |  |  |  |  |  |  |  |  |  |  |  |  |  |  |  |  |  |  |  |  |  |  |  |  |  |  |  |  |  |  |  |  |  |  |  |  |  |
| 14 | *T. albopunctatum_*14 |  |  |  |  |  |  |  |  |  |  |  |  |  |  |  |  |  |  |  |  |  |  |  |  |  |  |  |  |  |  |  |  |  |  |  |  |  |  |  |  |  |  |  |  |  |
| 15 | *T. albopunctatum_*15 |  |  |  |  |  |  |  |  |  |  |  |  |  |  |  |  |  |  |  |  |  |  |  |  |  |  |  |  |  |  |  |  |  |  |  |  |  |  |  |  |  |  |  |  |  |
| 16 | *T. albopunctatum_*16 |  |  |  |  |  |  |  |  |  |  |  |  |  |  |  |  |  |  |  |  |  |  |  |  |  |  |  |  |  |  |  |  |  |  |  |  |  |  |  |  |  |  |  |  |  |
| 17 | *T. albopunctatum_*17 |  |  |  |  |  |  |  |  |  |  |  |  |  |  |  |  |  |  |  |  |  |  |  |  |  |  |  |  |  |  |  |  |  |  |  |  |  |  |  |  |  |  |  |  |  |
| 18 | *T. albopunctatum_*18 |  |  |  |  |  |  |  |  |  |  |  |  |  |  |  |  |  |  |  |  |  |  |  |  |  |  |  |  |  |  |  |  |  |  |  |  |  |  |  |  |  |  |  |  |  |
| 19 | *T. albopunctatum_*19 |  |  |  |  |  |  |  |  |  |  |  |  |  |  |  |  |  |  |  |  |  |  |  |  |  |  |  |  |  |  |  |  |  |  |  |  |  |  |  |  |  |  |  |  |  |
| 20 | *T. albopunctatum_*20 |  |  |  |  |  |  |  |  |  |  |  |  |  |  |  |  |  |  |  |  |  |  |  |  |  |  |  |  |  |  |  |  |  |  |  |  |  |  |  |  |  |  |  |  |  |
| 21 | *T. albopunctatum_*21 |  |  |  |  |  |  |  |  |  |  |  |  |  |  |  |  |  |  |  |  |  |  |  |  |  |  |  |  |  |  |  |  |  |  |  |  |  |  |  |  |  |  |  |  |  |
| 22 | *T. albopunctatum_*22 |  |  |  |  |  |  |  |  |  |  |  |  |  |  |  |  |  |  |  |  |  |  |  |  |  |  |  |  |  |  |  |  |  |  |  |  |  |  |  |  |  |  |  |  |  |
| 23 | *T. albopunctatum_*23 |  |  |  |  |  |  |  |  |  |  |  |  |  |  |  |  |  |  |  |  |  |  |  |  |  |  |  |  |  |  |  |  |  |  |  |  |  |  |  |  |  |  |  |  |  |
| 24 | *T. albopunctatum_*24 |  |  |  |  |  |  |  |  |  |  |  |  |  |  |  |  |  |  |  |  |  |  |  |  |  |  |  |  |  |  |  |  |  |  |  |  |  |  |  |  |  |  |  |  |  |
| 25 | *T. albopunctatum_*25 |  |  |  |  |  |  |  |  |  |  |  |  |  |  |  |  |  |  |  |  |  |  |  |  |  |  |  |  |  |  |  |  |  |  |  |  |  |  |  |  |  |  |  |  |  |
| 26 | *T. albopunctatum_*26 |  |  |  |  |  |  |  |  |  |  |  |  |  |  |  |  |  |  |  |  |  |  |  |  |  |  |  |  |  |  |  |  |  |  |  |  |  |  |  |  |  |  |  |  |  |
| 27 | *T. albopunctatum_*28 |  |  |  |  |  |  |  |  |  |  |  |  |  |  |  |  |  |  |  |  |  |  |  |  |  |  |  |  |  |  |  |  |  |  |  |  |  |  |  |  |  |  |  |  |  |
| 28 | *T. albopunctatum_*29 |  |  |  |  |  |  |  |  |  |  |  |  |  |  |  |  |  |  |  |  |  |  |  |  |  |  |  |  |  |  |  |  |  |  |  |  |  |  |  |  |  |  |  |  |  |
| 29 | *T. albopunctatum_*30 |  |  |  |  |  |  |  |  |  |  |  |  |  |  |  |  |  |  |  |  |  |  |  |  |  |  |  |  |  |  |  |  |  |  |  |  |  |  |  |  |  |  |  |  |  |
| 30 | *T. albopunctatum_*31 |  |  |  |  |  |  |  |  |  |  |  |  |  |  |  |  |  |  |  |  |  |  |  |  |  |  |  |  |  |  |  |  |  |  |  |  |  |  |  |  |  |  |  |  |  |
| 31 | *T. albopunctatum_*32 |  |  |  |  |  |  |  |  |  |  |  |  |  |  |  |  |  |  |  |  |  |  |  |  |  |  |  |  |  |  |  |  |  |  |  |  |  |  |  |  |  |  |  |  |  |
| 32 | *T. albopunctatum_*33 |  |  |  |  |  |  |  |  |  |  |  |  |  |  |  |  |  |  |  |  |  |  |  |  |  |  |  |  |  |  |  |  |  |  |  |  |  |  |  |  |  |  |  |  |  |
| 33 | *T. albopunctatum_*34 |  |  |  |  |  |  |  |  |  |  |  |  |  |  |  |  |  |  |  |  |  |  |  |  |  |  |  |  |  |  |  |  |  |  |  |  |  |  |  |  |  |  |  |  |  |
| 34 | *T. albopunctatum_*35 |  |  |  |  |  |  |  |  |  |  |  |  |  |  |  |  |  |  |  |  |  |  |  |  |  |  |  |  |  |  |  |  |  |  |  |  |  |  |  |  |  |  |  |  |  |
| 35 | *T. albopunctatum_*36 |  |  |  |  |  |  |  |  |  |  |  |  |  |  |  |  |  |  |  |  |  |  |  |  |  |  |  |  |  |  |  |  |  |  |  |  |  |  |  |  |  |  |  |  |  |
| 36 | *T. albopunctatum_*37 |  |  |  |  |  |  |  |  |  |  |  |  |  |  |  |  |  |  |  |  |  |  |  |  |  |  |  |  |  |  |  |  |  |  |  |  |  |  |  |  |  |  |  |  |  |
| 37 | *T. annae_*2 |  |  |  |  |  |  |  |  |  |  |  |  |  |  |  |  |  |  |  |  |  |  |  |  |  |  |  |  |  |  |  |  |  |  |  |  |  |  |  |  |  |  |  |  |  |
| 38 | *T. annae_*3 |  |  |  |  |  |  |  |  |  |  |  |  |  |  |  |  |  |  |  |  |  |  |  |  |  |  |  |  |  |  |  |  |  |  |  |  |  |  |  |  |  |  |  |  |  |
| 39 | *T. annae_*4 |  |  |  |  |  |  |  |  |  |  |  |  |  |  |  |  |  |  |  |  |  |  |  |  |  |  |  |  |  |  |  |  |  |  |  |  |  |  |  |  |  |  |  |  |  |
| 40 | *T. annae_*5 |  |  |  |  |  |  |  |  |  |  |  |  |  |  |  |  |  |  |  |  |  |  |  |  |  |  |  |  |  |  |  |  |  |  |  |  |  |  |  |  |  |  |  |  |  |
| 41 | *T. asperum_*1 |  |  |  |  |  |  |  |  |  |  |  |  |  |  |  |  |  |  |  |  |  |  |  |  |  |  |  |  |  |  |  |  |  |  |  |  |  |  |  |  |  |  |  |  |  |
| 42 | *T. asperum_*2 |  |  |  |  |  |  |  |  |  |  |  |  |  |  |  |  |  |  |  |  |  |  |  |  |  |  |  |  |  |  |  |  |  |  |  |  |  |  |  |  |  |  |  |  |  |
| 43 | *T. auratum_*1 |  |  |  |  |  |  |  |  |  |  |  |  |  |  |  |  |  |  |  |  |  |  |  |  |  |  |  |  |  |  |  |  |  |  |  |  |  |  |  |  |  |  |  |  |  |
| 44 | *T. auratum_*2 |  |  |  |  |  |  |  |  |  |  |  |  |  |  |  |  |  |  |  |  |  |  |  |  |  |  |  |  |  |  |  |  |  |  |  |  |  |  |  |  |  |  |  |  |  |
| 45 | *T. auratum_*3 |  |  |  |  |  |  |  |  |  |  |  |  |  |  |  |  |  |  |  |  |  |  |  |  |  |  |  |  |  |  |  |  |  |  |  |  |  |  |  |  |  |  |  |  |  |
| 46 | *T. auratum_*4 |  |  |  |  |  |  |  |  |  |  |  |  |  |  |  |  |  |  |  |  |  |  |  |  |  |  |  |  |  |  |  |  |  |  |  |  |  |  |  |  |  |  |  |  |  |
| 47 | *T. auratum_*5 |  |  |  |  |  |  |  |  |  |  |  |  |  |  |  |  |  |  |  |  |  |  |  |  |  |  |  |  |  |  |  |  |  |  |  |  |  |  |  |  |  |  |  |  |  |
| 48 | *T. auratum_*6 |  |  |  |  |  |  |  |  |  |  |  |  |  |  |  |  |  |  |  |  |  |  |  |  |  |  |  |  |  |  |  |  |  |  |  |  |  |  |  |  |  |  |  |  |  |
| 49 | *T. baibungense_*2 |  |  |  |  |  |  |  |  |  |  |  |  |  |  |  |  |  |  |  |  |  |  |  |  |  |  |  |  |  |  |  |  |  |  |  |  |  |  |  |  |  |  |  |  |  |
| 50 | *T. baibungense_*3 |  |  |  |  |  |  |  |  |  |  |  |  |  |  |  |  |  |  |  |  |  |  |  |  |  |  |  |  |  |  |  |  |  |  |  |  |  |  |  |  |  |  |  |  |  |
| 51 | *T. baibungense_*4 |  |  |  |  |  |  |  |  |  |  |  |  |  |  |  |  |  |  |  |  |  |  |  |  |  |  |  |  |  |  |  |  |  |  |  |  |  |  |  |  |  |  |  |  |  |
| 52 | *T. bicolor_*1 |  |  |  |  |  |  |  |  |  |  |  |  |  |  |  |  |  |  |  |  |  |  |  |  |  |  |  |  |  |  |  |  |  |  |  |  |  |  |  |  |  |  |  |  |  |
| 53 | *T. bicolor_*2 |  |  |  |  |  |  |  |  |  |  |  |  |  |  |  |  |  |  |  |  |  |  |  |  |  |  |  |  |  |  |  |  |  |  |  |  |  |  |  |  |  |  |  |  |  |
| 54 | *T. bicolor_*3 |  |  |  |  |  |  |  |  |  |  |  |  |  |  |  |  |  |  |  |  |  |  |  |  |  |  |  |  |  |  |  |  |  |  |  |  |  |  |  |  |  |  |  |  |  |
| 55 | *T. bicolor_*4 |  |  |  |  |  |  |  |  |  |  |  |  |  |  |  |  |  |  |  |  |  |  |  |  |  |  |  |  |  |  |  |  |  |  |  |  |  |  |  |  |  |  |  |  |  |
| 56 | *T. bicolor_*5 |  |  |  |  |  |  |  |  |  |  |  |  |  |  |  |  |  |  |  |  |  |  |  |  |  |  |  |  |  |  |  |  |  |  |  |  |  |  |  |  |  |  |  |  |  |
| 57 | *T. bicolor_*6 |  |  |  |  |  |  |  |  |  |  |  |  |  |  |  |  |  |  |  |  |  |  |  |  |  |  |  |  |  |  |  |  |  |  |  |  |  |  |  |  |  |  |  |  |  |
| 58 | *T. bicolor_*7 |  |  |  |  |  |  |  |  |  |  |  |  |  |  |  |  |  |  |  |  |  |  |  |  |  |  |  |  |  |  |  |  |  |  |  |  |  |  |  |  |  |  |  |  |  |
| 59 | *T. bicolor_*8 |  |  |  |  |  |  |  |  |  |  |  |  |  |  |  |  |  |  |  |  |  |  |  |  |  |  |  |  |  |  |  |  |  |  |  |  |  |  |  |  |  |  |  |  |  |
| 60 | *T. bicolor_*9 |  |  |  |  |  |  |  |  |  |  |  |  |  |  |  |  |  |  |  |  |  |  |  |  |  |  |  |  |  |  |  |  |  |  |  |  |  |  |  |  |  |  |  |  |  |
| 61 | *T. bicolor_*10 |  |  |  |  |  |  |  |  |  |  |  |  |  |  |  |  |  |  |  |  |  |  |  |  |  |  |  |  |  |  |  |  |  |  |  |  |  |  |  |  |  |  |  |  |  |
| 62 | *T. corticale_*1 |  |  |  |  |  |  |  |  |  |  |  |  |  |  |  |  |  |  |  |  |  |  |  |  |  |  |  |  |  |  |  |  |  |  |  |  |  |  |  |  |  |  |  |  |  |
| 63 | *T. corticale_*2 |  |  |  |  |  |  |  |  |  |  |  |  |  |  |  |  |  |  |  |  |  |  |  |  |  |  |  |  |  |  |  |  |  |  |  |  |  |  |  |  |  |  |  |  |  |
| 64 | *T. corticale_*3 |  |  |  |  |  |  |  |  |  |  |  |  |  |  |  |  |  |  |  |  |  |  |  |  |  |  |  |  |  |  |  |  |  |  |  |  |  |  |  |  |  |  |  |  |  |
| 65 | *T. corticale_*4 |  |  |  |  |  |  |  |  |  |  |  |  |  |  |  |  |  |  |  |  |  |  |  |  |  |  |  |  |  |  |  |  |  |  |  |  |  |  |  |  |  |  |  |  |  |
| 66 | *T. corticale_*5 |  |  |  |  |  |  |  |  |  |  |  |  |  |  |  |  |  |  |  |  |  |  |  |  |  |  |  |  |  |  |  |  |  |  |  |  |  |  |  |  |  |  |  |  |  |
| 67 | *T. corticale_*6 |  |  |  |  |  |  |  |  |  |  |  |  |  |  |  |  |  |  |  |  |  |  |  |  |  |  |  |  |  |  |  |  |  |  |  |  |  |  |  |  |  |  |  |  |  |
| 68 | *T. corticale_*7 |  |  |  |  |  |  |  |  |  |  |  |  |  |  |  |  |  |  |  |  |  |  |  |  |  |  |  |  |  |  |  |  |  |  |  |  |  |  |  |  |  |  |  |  |  |
| 69 | *T. corticale_*8 |  |  |  |  |  |  |  |  |  |  |  |  |  |  |  |  |  |  |  |  |  |  |  |  |  |  |  |  |  |  |  |  |  |  |  |  |  |  |  |  |  |  |  |  |  |
| 70 | *T. corticale_*9 |  |  |  |  |  |  |  |  |  |  |  |  |  |  |  |  |  |  |  |  |  |  |  |  |  |  |  |  |  |  |  |  |  |  |  |  |  |  |  |  |  |  |  |  |  |
| 71 | *T. corticale_*10 |  |  |  |  |  |  |  |  |  |  |  |  |  |  |  |  |  |  |  |  |  |  |  |  |  |  |  |  |  |  |  |  |  |  |  |  |  |  |  |  |  |  |  |  |  |
| 72 | *T. corticale_*11 |  |  |  |  |  |  |  |  |  |  |  |  |  |  |  |  |  |  |  |  |  |  |  |  |  |  |  |  |  |  |  |  |  |  |  |  |  |  |  |  |  |  |  |  |  |
| 73 | *T. corticale_*12 |  |  |  |  |  |  |  |  |  |  |  |  |  |  |  |  |  |  |  |  |  |  |  |  |  |  |  |  |  |  |  |  |  |  |  |  |  |  |  |  |  |  |  |  |  |
| 74 | *T. corticale_*13 |  |  |  |  |  |  |  |  |  |  |  |  |  |  |  |  |  |  |  |  |  |  |  |  |  |  |  |  |  |  |  |  |  |  |  |  |  |  |  |  |  |  |  |  |  |
| 75 | *T. corticale_*14 |  |  |  |  |  |  |  |  |  |  |  |  |  |  |  |  |  |  |  |  |  |  |  |  |  |  |  |  |  |  |  |  |  |  |  |  |  |  |  |  |  |  |  |  |  |
| 76 | *T. corticale_*15 |  |  |  |  |  |  |  |  |  |  |  |  |  |  |  |  |  |  |  |  |  |  |  |  |  |  |  |  |  |  |  |  |  |  |  |  |  |  |  |  |  |  |  |  |  |
| 77 | *T. corticale_*16 |  |  |  |  |  |  |  |  |  |  |  |  |  |  |  |  |  |  |  |  |  |  |  |  |  |  |  |  |  |  |  |  |  |  |  |  |  |  |  |  |  |  |  |  |  |
| 78 | *T. corticale_*17 |  |  |  |  |  |  |  |  |  |  |  |  |  |  |  |  |  |  |  |  |  |  |  |  |  |  |  |  |  |  |  |  |  |  |  |  |  |  |  |  |  |  |  |  |  |
| 79 | *T. corticale_*18 |  |  |  |  |  |  |  |  |  |  |  |  |  |  |  |  |  |  |  |  |  |  |  |  |  |  |  |  |  |  |  |  |  |  |  |  |  |  |  |  |  |  |  |  |  |
| 80 | *T. corticale_*19 |  |  |  |  |  |  |  |  |  |  |  |  |  |  |  |  |  |  |  |  |  |  |  |  |  |  |  |  |  |  |  |  |  |  |  |  |  |  |  |  |  |  |  |  |  |
| 81 | *T. corticale_*20 |  |  |  |  |  |  |  |  |  |  |  |  |  |  |  |  |  |  |  |  |  |  |  |  |  |  |  |  |  |  |  |  |  |  |  |  |  |  |  |  |  |  |  |  |  |
| 82 | *T. corticale_*21 |  |  |  |  |  |  |  |  |  |  |  |  |  |  |  |  |  |  |  |  |  |  |  |  |  |  |  |  |  |  |  |  |  |  |  |  |  |  |  |  |  |  |  |  |  |
| 83 | *T. corticale_*22 |  |  |  |  |  |  |  |  |  |  |  |  |  |  |  |  |  |  |  |  |  |  |  |  |  |  |  |  |  |  |  |  |  |  |  |  |  |  |  |  |  |  |  |  |  |
| 84 | *T. corticale_*23 |  |  |  |  |  |  |  |  |  |  |  |  |  |  |  |  |  |  |  |  |  |  |  |  |  |  |  |  |  |  |  |  |  |  |  |  |  |  |  |  |  |  |  |  |  |
| 85 | *T. gordoni_*1 |  |  |  |  |  |  |  |  |  |  |  |  |  |  |  |  |  |  |  |  |  |  |  |  |  |  |  |  |  |  |  |  |  |  |  |  |  |  |  |  |  |  |  |  |  |
| 86 | *T. gordoni_*2 |  |  |  |  |  |  |  |  |  |  |  |  |  |  |  |  |  |  |  |  |  |  |  |  |  |  |  |  |  |  |  |  |  |  |  |  |  |  |  |  |  |  |  |  |  |
| 87 | *T. gordoni_*3 |  |  |  |  |  |  |  |  |  |  |  |  |  |  |  |  |  |  |  |  |  |  |  |  |  |  |  |  |  |  |  |  |  |  |  |  |  |  |  |  |  |  |  |  |  |
| 88 | *T. gordoni_*4 |  |  |  |  |  |  |  |  |  |  |  |  |  |  |  |  |  |  |  |  |  |  |  |  |  |  |  |  |  |  |  |  |  |  |  |  |  |  |  |  |  |  |  |  |  |
| 89 | *T. gordoni_*5 |  |  |  |  |  |  |  |  |  |  |  |  |  |  |  |  |  |  |  |  |  |  |  |  |  |  |  |  |  |  |  |  |  |  |  |  |  |  |  |  |  |  |  |  |  |
| 90 | *T. gordoni_*6 |  |  |  |  |  |  |  |  |  |  |  |  |  |  |  |  |  |  |  |  |  |  |  |  |  |  |  |  |  |  |  |  |  |  |  |  |  |  |  |  |  |  |  |  |  |
| 91 | *T. gordoni_*7 |  |  |  |  |  |  |  |  |  |  |  |  |  |  |  |  |  |  |  |  |  |  |  |  |  |  |  |  |  |  |  |  |  |  |  |  |  |  |  |  |  |  |  |  |  |
| 92 | *T. gordoni_*8 |  |  |  |  |  |  |  |  |  |  |  |  |  |  |  |  |  |  |  |  |  |  |  |  |  |  |  |  |  |  |  |  |  |  |  |  |  |  |  |  |  |  |  |  |  |
| 93 | *T. hekouense_*1 |  |  |  |  |  |  |  |  |  |  |  |  |  |  |  |  |  |  |  |  |  |  |  |  |  |  |  |  |  |  |  |  |  |  |  |  |  |  |  |  |  |  |  |  |  |
| 94 | *T. hekouense_*2 |  |  |  |  |  |  |  |  |  |  |  |  |  |  |  |  |  |  |  |  |  |  |  |  |  |  |  |  |  |  |  |  |  |  |  |  |  |  |  |  |  |  |  |  |  |
| 95 | *T. hekouense_*3 |  |  |  |  |  |  |  |  |  |  |  |  |  |  |  |  |  |  |  |  |  |  |  |  |  |  |  |  |  |  |  |  |  |  |  |  |  |  |  |  |  |  |  |  |  |
| 96 | *T. hekouense_*4 |  |  |  |  |  |  |  |  |  |  |  |  |  |  |  |  |  |  |  |  |  |  |  |  |  |  |  |  |  |  |  |  |  |  |  |  |  |  |  |  |  |  |  |  |  |
| 97 | *T. hekouense_*5 |  |  |  |  |  |  |  |  |  |  |  |  |  |  |  |  |  |  |  |  |  |  |  |  |  |  |  |  |  |  |  |  |  |  |  |  |  |  |  |  |  |  |  |  |  |
| 98 | *T. hekouense_*6 |  |  |  |  |  |  |  |  |  |  |  |  |  |  |  |  |  |  |  |  |  |  |  |  |  |  |  |  |  |  |  |  |  |  |  |  |  |  |  |  |  |  |  |  |  |
| 99 | *T. hekouense_*7 |  |  |  |  |  |  |  |  |  |  |  |  |  |  |  |  |  |  |  |  |  |  |  |  |  |  |  |  |  |  |  |  |  |  |  |  |  |  |  |  |  |  |  |  |  |
| 100 | *T. hekouense_*8 |  |  |  |  |  |  |  |  |  |  |  |  |  |  |  |  |  |  |  |  |  |  |  |  |  |  |  |  |  |  |  |  |  |  |  |  |  |  |  |  |  |  |  |  |  |
| 101 | *T. hekouense_*9 |  |  |  |  |  |  |  |  |  |  |  |  |  |  |  |  |  |  |  |  |  |  |  |  |  |  |  |  |  |  |  |  |  |  |  |  |  |  |  |  |  |  |  |  |  |
| 102 | *T. hekouense_*10 |  |  |  |  |  |  |  |  |  |  |  |  |  |  |  |  |  |  |  |  |  |  |  |  |  |  |  |  |  |  |  |  |  |  |  |  |  |  |  |  |  |  |  |  |  |
| 103 | *T. horridum_*1 |  |  |  |  |  |  |  |  |  |  |  |  |  |  |  |  |  |  |  |  |  |  |  |  |  |  |  |  |  |  |  |  |  |  |  |  |  |  |  |  |  |  |  |  |  |
| 104 | *T. horridum_*2 |  |  |  |  |  |  |  |  |  |  |  |  |  |  |  |  |  |  |  |  |  |  |  |  |  |  |  |  |  |  |  |  |  |  |  |  |  |  |  |  |  |  |  |  |  |
| 105 | *T. horridum_*3 |  |  |  |  |  |  |  |  |  |  |  |  |  |  |  |  |  |  |  |  |  |  |  |  |  |  |  |  |  |  |  |  |  |  |  |  |  |  |  |  |  |  |  |  |  |
| 106 | *T. horridum5* |  |  |  |  |  |  |  |  |  |  |  |  |  |  |  |  |  |  |  |  |  |  |  |  |  |  |  |  |  |  |  |  |  |  |  |  |  |  |  |  |  |  |  |  |  |
| 107 | *T. khoii_*1 |  |  |  |  |  |  |  |  |  |  |  |  |  |  |  |  |  |  |  |  |  |  |  |  |  |  |  |  |  |  |  |  |  |  |  |  |  |  |  |  |  |  |  |  |  |
| 108 | *T. khoii*2 |  |  |  |  |  |  |  |  |  |  |  |  |  |  |  |  |  |  |  |  |  |  |  |  |  |  |  |  |  |  |  |  |  |  |  |  |  |  |  |  |  |  |  |  |  |
| 109 | *T. lacustrinum_*1 |  |  |  |  |  |  |  |  |  |  |  |  |  |  |  |  |  |  |  |  |  |  |  |  |  |  |  |  |  |  |  |  |  |  |  |  |  |  |  |  |  |  |  |  |  |
| 110 | *T. lacustrinum_*2 |  |  |  |  |  |  |  |  |  |  |  |  |  |  |  |  |  |  |  |  |  |  |  |  |  |  |  |  |  |  |  |  |  |  |  |  |  |  |  |  |  |  |  |  |  |
| 111 | *T. laeve_*1 |  |  |  |  |  |  |  |  |  |  |  |  |  |  |  |  |  |  |  |  |  |  |  |  |  |  |  |  |  |  |  |  |  |  |  |  |  |  |  |  |  |  |  |  |  |
| 112 | *T. laeve_*2 |  |  |  |  |  |  |  |  |  |  |  |  |  |  |  |  |  |  |  |  |  |  |  |  |  |  |  |  |  |  |  |  |  |  |  |  |  |  |  |  |  |  |  |  |  |
| 113 | *T. laeve_*3 |  |  |  |  |  |  |  |  |  |  |  |  |  |  |  |  |  |  |  |  |  |  |  |  |  |  |  |  |  |  |  |  |  |  |  |  |  |  |  |  |  |  |  |  |  |
| 114 | *T. laeve_*4 |  |  |  |  |  |  |  |  |  |  |  |  |  |  |  |  |  |  |  |  |  |  |  |  |  |  |  |  |  |  |  |  |  |  |  |  |  |  |  |  |  |  |  |  |  |
| 115 | *T. laeve_*5 |  |  |  |  |  |  |  |  |  |  |  |  |  |  |  |  |  |  |  |  |  |  |  |  |  |  |  |  |  |  |  |  |  |  |  |  |  |  |  |  |  |  |  |  |  |
| 116 | *T. laeve_*6 |  |  |  |  |  |  |  |  |  |  |  |  |  |  |  |  |  |  |  |  |  |  |  |  |  |  |  |  |  |  |  |  |  |  |  |  |  |  |  |  |  |  |  |  |  |
| 117 | *T. laeve_*7 |  |  |  |  |  |  |  |  |  |  |  |  |  |  |  |  |  |  |  |  |  |  |  |  |  |  |  |  |  |  |  |  |  |  |  |  |  |  |  |  |  |  |  |  |  |
| 118 | *T. laeve_*8 |  |  |  |  |  |  |  |  |  |  |  |  |  |  |  |  |  |  |  |  |  |  |  |  |  |  |  |  |  |  |  |  |  |  |  |  |  |  |  |  |  |  |  |  |  |
| 119 | *T. laeve_*9 |  |  |  |  |  |  |  |  |  |  |  |  |  |  |  |  |  |  |  |  |  |  |  |  |  |  |  |  |  |  |  |  |  |  |  |  |  |  |  |  |  |  |  |  |  |
| 120 | *T. laeve_*10 |  |  |  |  |  |  |  |  |  |  |  |  |  |  |  |  |  |  |  |  |  |  |  |  |  |  |  |  |  |  |  |  |  |  |  |  |  |  |  |  |  |  |  |  |  |
| 121 | *T. lateriticum_*1 |  |  |  |  |  |  |  |  |  |  |  |  |  |  |  |  |  |  |  |  |  |  |  |  |  |  |  |  |  |  |  |  |  |  |  |  |  |  |  |  |  |  |  |  |  |
| 122 | *T. lateriticum_*2 |  |  |  |  |  |  |  |  |  |  |  |  |  |  |  |  |  |  |  |  |  |  |  |  |  |  |  |  |  |  |  |  |  |  |  |  |  |  |  |  |  |  |  |  |  |
| 123 | *T. lateriticum_*3 |  |  |  |  |  |  |  |  |  |  |  |  |  |  |  |  |  |  |  |  |  |  |  |  |  |  |  |  |  |  |  |  |  |  |  |  |  |  |  |  |  |  |  |  |  |
| 124 | *T. lateriticum_*4 |  |  |  |  |  |  |  |  |  |  |  |  |  |  |  |  |  |  |  |  |  |  |  |  |  |  |  |  |  |  |  |  |  |  |  |  |  |  |  |  |  |  |  |  |  |
| 125 | *T. lateriticum_*5 |  |  |  |  |  |  |  |  |  |  |  |  |  |  |  |  |  |  |  |  |  |  |  |  |  |  |  |  |  |  |  |  |  |  |  |  |  |  |  |  |  |  |  |  |  |
| 126 | *T. lateriticum_*6 |  |  |  |  |  |  |  |  |  |  |  |  |  |  |  |  |  |  |  |  |  |  |  |  |  |  |  |  |  |  |  |  |  |  |  |  |  |  |  |  |  |  |  |  |  |
| 127 | *T. lateriticum_*LT01 |  |  |  |  |  |  |  |  |  |  |  |  |  |  |  |  |  |  |  |  |  |  |  |  |  |  |  |  |  |  |  |  |  |  |  |  |  |  |  |  |  |  |  |  |  |
| 128 | *T. lateriticum_*LT011 |  |  |  |  |  |  |  |  |  |  |  |  |  |  |  |  |  |  |  |  |  |  |  |  |  |  |  |  |  |  |  |  |  |  |  |  |  |  |  |  |  |  |  |  |  |
| 129 | *T. leporosum_*1 |  |  |  |  |  |  |  |  |  |  |  |  |  |  |  |  |  |  |  |  |  |  |  |  |  |  |  |  |  |  |  |  |  |  |  |  |  |  |  |  |  |  |  |  |  |
| 130 | *T. leporosum_*2 |  |  |  |  |  |  |  |  |  |  |  |  |  |  |  |  |  |  |  |  |  |  |  |  |  |  |  |  |  |  |  |  |  |  |  |  |  |  |  |  |  |  |  |  |  |
| 131 | *T. leporosum_*3 |  |  |  |  |  |  |  |  |  |  |  |  |  |  |  |  |  |  |  |  |  |  |  |  |  |  |  |  |  |  |  |  |  |  |  |  |  |  |  |  |  |  |  |  |  |
| 132 | *T. licin_*1 |  |  |  |  |  |  |  |  |  |  |  |  |  |  |  |  |  |  |  |  |  |  |  |  |  |  |  |  |  |  |  |  |  |  |  |  |  |  |  |  |  |  |  |  |  |
| 133 | *T. licin_*2 |  |  |  |  |  |  |  |  |  |  |  |  |  |  |  |  |  |  |  |  |  |  |  |  |  |  |  |  |  |  |  |  |  |  |  |  |  |  |  |  |  |  |  |  |  |
| 134 | *T. licin_*3 |  |  |  |  |  |  |  |  |  |  |  |  |  |  |  |  |  |  |  |  |  |  |  |  |  |  |  |  |  |  |  |  |  |  |  |  |  |  |  |  |  |  |  |  |  |
| 135 | *T. moloch_*1 |  |  |  |  |  |  |  |  |  |  |  |  |  |  |  |  |  |  |  |  |  |  |  |  |  |  |  |  |  |  |  |  |  |  |  |  |  |  |  |  |  |  |  |  |  |
| 136 | *T. moloch_*2 |  |  |  |  |  |  |  |  |  |  |  |  |  |  |  |  |  |  |  |  |  |  |  |  |  |  |  |  |  |  |  |  |  |  |  |  |  |  |  |  |  |  |  |  |  |
| 137 | *T. moloch_*4 |  |  |  |  |  |  |  |  |  |  |  |  |  |  |  |  |  |  |  |  |  |  |  |  |  |  |  |  |  |  |  |  |  |  |  |  |  |  |  |  |  |  |  |  |  |
| 138 | *T. nebulosum_*1 |  |  |  |  |  |  |  |  |  |  |  |  |  |  |  |  |  |  |  |  |  |  |  |  |  |  |  |  |  |  |  |  |  |  |  |  |  |  |  |  |  |  |  |  |  |
| 139 | *T. nebulosum_*2 |  |  |  |  |  |  |  |  |  |  |  |  |  |  |  |  |  |  |  |  |  |  |  |  |  |  |  |  |  |  |  |  |  |  |  |  |  |  |  |  |  |  |  |  |  |
| 140 | *T. nebulosum_*3 |  |  |  |  |  |  |  |  |  |  |  |  |  |  |  |  |  |  |  |  |  |  |  |  |  |  |  |  |  |  |  |  |  |  |  |  |  |  |  |  |  |  |  |  |  |
| 141 | *T. nebulosum_*4 |  |  |  |  |  |  |  |  |  |  |  |  |  |  |  |  |  |  |  |  |  |  |  |  |  |  |  |  |  |  |  |  |  |  |  |  |  |  |  |  |  |  |  |  |  |
| 142 | *T. palliatum_*1 |  |  |  |  |  |  |  |  |  |  |  |  |  |  |  |  |  |  |  |  |  |  |  |  |  |  |  |  |  |  |  |  |  |  |  |  |  |  |  |  |  |  |  |  |  |
| 143 | *T. palliatum_*2 |  |  |  |  |  |  |  |  |  |  |  |  |  |  |  |  |  |  |  |  |  |  |  |  |  |  |  |  |  |  |  |  |  |  |  |  |  |  |  |  |  |  |  |  |  |
| 144 | *T. palliatum_*3 |  |  |  |  |  |  |  |  |  |  |  |  |  |  |  |  |  |  |  |  |  |  |  |  |  |  |  |  |  |  |  |  |  |  |  |  |  |  |  |  |  |  |  |  |  |
| 145 | *T. palliatum_*4 |  |  |  |  |  |  |  |  |  |  |  |  |  |  |  |  |  |  |  |  |  |  |  |  |  |  |  |  |  |  |  |  |  |  |  |  |  |  |  |  |  |  |  |  |  |
| 146 | *T. palliatum_*5 |  |  |  |  |  |  |  |  |  |  |  |  |  |  |  |  |  |  |  |  |  |  |  |  |  |  |  |  |  |  |  |  |  |  |  |  |  |  |  |  |  |  |  |  |  |
| 147 | *T. palliatum_*6 |  |  |  |  |  |  |  |  |  |  |  |  |  |  |  |  |  |  |  |  |  |  |  |  |  |  |  |  |  |  |  |  |  |  |  |  |  |  |  |  |  |  |  |  |  |
| 148 | *T. palliatum_*7 |  |  |  |  |  |  |  |  |  |  |  |  |  |  |  |  |  |  |  |  |  |  |  |  |  |  |  |  |  |  |  |  |  |  |  |  |  |  |  |  |  |  |  |  |  |
| 149 | *T. palliatum_*8 |  |  |  |  |  |  |  |  |  |  |  |  |  |  |  |  |  |  |  |  |  |  |  |  |  |  |  |  |  |  |  |  |  |  |  |  |  |  |  |  |  |  |  |  |  |
| 150 | *T. palliatum_*9 |  |  |  |  |  |  |  |  |  |  |  |  |  |  |  |  |  |  |  |  |  |  |  |  |  |  |  |  |  |  |  |  |  |  |  |  |  |  |  |  |  |  |  |  |  |
| 151 | *T. palliatum_*10 |  |  |  |  |  |  |  |  |  |  |  |  |  |  |  |  |  |  |  |  |  |  |  |  |  |  |  |  |  |  |  |  |  |  |  |  |  |  |  |  |  |  |  |  |  |
| 152 | *T. palliatum_*11 | 0.7 |  |  |  |  |  |  |  |  |  |  |  |  |  |  |  |  |  |  |  |  |  |  |  |  |  |  |  |  |  |  |  |  |  |  |  |  |  |  |  |  |  |  |  |  |
| 153 | *T. petilum_*11 | 14.6 | 14.6 |  |  |  |  |  |  |  |  |  |  |  |  |  |  |  |  |  |  |  |  |  |  |  |  |  |  |  |  |  |  |  |  |  |  |  |  |  |  |  |  |  |  |  |
| 154 | *T. phrynoderma_*1 | 15.0 | 15.0 | 13.8 |  |  |  |  |  |  |  |  |  |  |  |  |  |  |  |  |  |  |  |  |  |  |  |  |  |  |  |  |  |  |  |  |  |  |  |  |  |  |  |  |  |  |
| 155 | *T. phrynoderma_*2 | 13.4 | 13.4 | 13.0 | 1.6 |  |  |  |  |  |  |  |  |  |  |  |  |  |  |  |  |  |  |  |  |  |  |  |  |  |  |  |  |  |  |  |  |  |  |  |  |  |  |  |  |  |
| 156 | *T. pyaukkya_*1 | 12.2 | 12.2 | 9.0 | 10.2 | 10.2 |  |  |  |  |  |  |  |  |  |  |  |  |  |  |  |  |  |  |  |  |  |  |  |  |  |  |  |  |  |  |  |  |  |  |  |  |  |  |  |  |
| 157 | *T. pyaukkya_*2 | 12.2 | 12.2 | 9.4 | 10.6 | 10.6 | 0.7 |  |  |  |  |  |  |  |  |  |  |  |  |  |  |  |  |  |  |  |  |  |  |  |  |  |  |  |  |  |  |  |  |  |  |  |  |  |  |  |
| 158 | *T. pyaukkya_*3 | 11.5 | 11.5 | 8.7 | 11.0 | 11.0 | 1.4 | 0.7 |  |  |  |  |  |  |  |  |  |  |  |  |  |  |  |  |  |  |  |  |  |  |  |  |  |  |  |  |  |  |  |  |  |  |  |  |  |  |
| 159 | *T. pyaukkya_*4 | 13.5 | 13.5 | 8.3 | 11.4 | 11.4 | 5.9 | 5.2 | 4.5 |  |  |  |  |  |  |  |  |  |  |  |  |  |  |  |  |  |  |  |  |  |  |  |  |  |  |  |  |  |  |  |  |  |  |  |  |  |
| 160 | *T. pyaukkya_*5 | 13.5 | 13.5 | 8.3 | 11.4 | 11.4 | 5.9 | 5.2 | 4.5 | 0.0 |  |  |  |  |  |  |  |  |  |  |  |  |  |  |  |  |  |  |  |  |  |  |  |  |  |  |  |  |  |  |  |  |  |  |  |  |
| 161 | *T. rhododiscus_*1 | 10.4 | 10.4 | 12.8 | 16.9 | 15.4 | 12.5 | 12.2 | 11.5 | 11.1 | 11.1 |  |  |  |  |  |  |  |  |  |  |  |  |  |  |  |  |  |  |  |  |  |  |  |  |  |  |  |  |  |  |  |  |  |  |  |
| 162 | *T. rhododiscus*10 | 10.1 | 10.1 | 12.8 | 16.5 | 15.0 | 12.5 | 12.2 | 11.5 | 10.8 | 10.8 | 1.4 |  |  |  |  |  |  |  |  |  |  |  |  |  |  |  |  |  |  |  |  |  |  |  |  |  |  |  |  |  |  |  |  |  |  |
| 163 | *T. rhododiscus_*2 | 10.4 | 10.4 | 12.8 | 16.9 | 15.4 | 12.5 | 12.2 | 11.5 | 11.1 | 11.1 | 0.0 | 1.4 |  |  |  |  |  |  |  |  |  |  |  |  |  |  |  |  |  |  |  |  |  |  |  |  |  |  |  |  |  |  |  |  |  |
| 164 | *T. rhododiscu_s*3 | 10.4 | 10.4 | 12.8 | 16.9 | 15.4 | 12.5 | 12.2 | 11.5 | 11.1 | 11.1 | 0.0 | 1.4 | 0.0 |  |  |  |  |  |  |  |  |  |  |  |  |  |  |  |  |  |  |  |  |  |  |  |  |  |  |  |  |  |  |  |  |
| 165 | *T. rhododiscus_*4 | 10.4 | 10.4 | 12.8 | 16.9 | 15.4 | 12.5 | 12.2 | 11.5 | 11.1 | 11.1 | 0.0 | 1.4 | 0.0 | 0.0 |  |  |  |  |  |  |  |  |  |  |  |  |  |  |  |  |  |  |  |  |  |  |  |  |  |  |  |  |  |  |  |
| 166 | *T. rhododiscus_*5 | 10.4 | 10.4 | 12.8 | 16.9 | 15.4 | 12.5 | 12.2 | 11.5 | 11.1 | 11.1 | 0.0 | 1.4 | 0.0 | 0.0 | 0.0 |  |  |  |  |  |  |  |  |  |  |  |  |  |  |  |  |  |  |  |  |  |  |  |  |  |  |  |  |  |  |
| 167 | *T. rhododiscus_*6 | 10.4 | 10.4 | 12.8 | 16.9 | 15.4 | 12.5 | 12.2 | 11.5 | 11.1 | 11.1 | 0.0 | 1.4 | 0.0 | 0.0 | 0.0 | 0.0 |  |  |  |  |  |  |  |  |  |  |  |  |  |  |  |  |  |  |  |  |  |  |  |  |  |  |  |  |  |
| 168 | *T. rhododiscus_*7 | 10.4 | 10.4 | 12.8 | 16.9 | 15.4 | 12.5 | 12.2 | 11.5 | 11.1 | 11.1 | 0.0 | 1.4 | 0.0 | 0.0 | 0.0 | 0.0 | 0.0 |  |  |  |  |  |  |  |  |  |  |  |  |  |  |  |  |  |  |  |  |  |  |  |  |  |  |  |  |
| 169 | *T. rhododiscus_*8 | 10.4 | 10.4 | 12.8 | 16.9 | 15.4 | 12.5 | 12.2 | 11.5 | 11.1 | 11.1 | 0.3 | 1.7 | 0.3 | 0.3 | 0.3 | 0.3 | 0.3 | 0.3 |  |  |  |  |  |  |  |  |  |  |  |  |  |  |  |  |  |  |  |  |  |  |  |  |  |  |  |
| 170 | *T. rhododiscus_*9 | 9.7 | 9.7 | 12.5 | 16.5 | 15.0 | 11.5 | 11.1 | 11.1 | 11.1 | 11.1 | 1.4 | 1.0 | 1.4 | 1.4 | 1.4 | 1.4 | 1.4 | 1.4 | 1.7 |  |  |  |  |  |  |  |  |  |  |  |  |  |  |  |  |  |  |  |  |  |  |  |  |  |  |
| 171 | *T. ryabovi_*1 | 13.5 | 13.5 | 13.5 | 9.8 | 8.3 | 10.4 | 10.8 | 10.8 | 10.8 | 10.8 | 11.1 | 10.8 | 11.1 | 11.1 | 11.1 | 11.1 | 11.1 | 11.1 | 11.1 | 11.1 |  |  |  |  |  |  |  |  |  |  |  |  |  |  |  |  |  |  |  |  |  |  |  |  |  |
| 172 | *T. ryabovi_*2 | 16.7 | 16.7 | 16.0 | 12.2 | 10.6 | 12.8 | 13.2 | 13.2 | 13.2 | 13.2 | 14.2 | 13.9 | 14.2 | 14.2 | 14.2 | 14.2 | 14.2 | 14.2 | 14.2 | 14.2 | 3.1 |  |  |  |  |  |  |  |  |  |  |  |  |  |  |  |  |  |  |  |  |  |  |  |  |
| 173 | *T. ryabovi_*3 | 16.7 | 16.7 | 16.0 | 12.2 | 10.6 | 12.8 | 13.2 | 13.2 | 13.2 | 13.2 | 14.2 | 13.9 | 14.2 | 14.2 | 14.2 | 14.2 | 14.2 | 14.2 | 14.2 | 14.2 | 3.1 | 0.0 |  |  |  |  |  |  |  |  |  |  |  |  |  |  |  |  |  |  |  |  |  |  |  |
| 174 | *T. stellatum_*1 | 15.3 | 16.0 | 16.0 | 20.9 | 20.1 | 18.8 | 18.4 | 17.7 | 18.4 | 18.4 | 16.0 | 16.0 | 16.0 | 16.0 | 16.0 | 16.0 | 16.0 | 16.0 | 15.6 | 16.3 | 20.5 | 23.3 | 23.3 |  |  |  |  |  |  |  |  |  |  |  |  |  |  |  |  |  |  |  |  |  |  |
| 175 | *T. stellatum_*2 | 15.3 | 16.0 | 16.0 | 20.9 | 20.1 | 18.8 | 18.4 | 17.7 | 18.4 | 18.4 | 16.0 | 16.0 | 16.0 | 16.0 | 16.0 | 16.0 | 16.0 | 16.0 | 15.6 | 16.3 | 20.5 | 23.3 | 23.3 | 0.0 |  |  |  |  |  |  |  |  |  |  |  |  |  |  |  |  |  |  |  |  |  |
| 176 | *T. truongsonense_*1 | 13.5 | 13.9 | 13.5 | 12.2 | 11.8 | 13.9 | 13.5 | 12.8 | 12.5 | 12.5 | 13.5 | 14.2 | 13.5 | 13.5 | 13.5 | 13.5 | 13.5 | 13.5 | 13.5 | 13.9 | 14.2 | 17.4 | 17.4 | 17.4 | 17.4 |  |  |  |  |  |  |  |  |  |  |  |  |  |  |  |  |  |  |  |  |
| 177 | *T. truongsonense_*2 | 13.5 | 13.9 | 14.2 | 13.8 | 13.4 | 14.6 | 14.2 | 13.5 | 13.2 | 13.2 | 13.2 | 13.9 | 13.2 | 13.2 | 13.2 | 13.2 | 13.2 | 13.2 | 13.2 | 13.5 | 14.6 | 17.7 | 17.7 | 17.0 | 17.0 | 1.4 |  |  |  |  |  |  |  |  |  |  |  |  |  |  |  |  |  |  |  |
| 178 | *T. truongsonense_*3 | 13.5 | 13.9 | 14.2 | 13.8 | 13.4 | 14.6 | 14.2 | 13.5 | 13.2 | 13.2 | 13.2 | 13.9 | 13.2 | 13.2 | 13.2 | 13.2 | 13.2 | 13.2 | 13.2 | 13.5 | 14.6 | 17.7 | 17.7 | 17.0 | 17.0 | 1.4 | 0.0 |  |  |  |  |  |  |  |  |  |  |  |  |  |  |  |  |  |  |
| 179 | *T. truongsonense_*4 | 13.5 | 13.9 | 14.2 | 12.6 | 11.0 | 14.2 | 14.2 | 13.5 | 13.5 | 13.5 | 13.9 | 14.6 | 13.9 | 13.9 | 13.9 | 13.9 | 13.9 | 13.9 | 13.9 | 14.2 | 11.8 | 14.9 | 14.9 | 16.3 | 16.3 | 5.6 | 6.6 | 6.6 |  |  |  |  |  |  |  |  |  |  |  |  |  |  |  |  |  |
| 180 | *T. truongsonense_*5 | 17.0 | 17.0 | 15.5 | 15.0 | 14.2 | 17.0 | 16.6 | 15.9 | 15.1 | 15.1 | 15.5 | 16.2 | 15.5 | 15.5 | 15.5 | 15.5 | 15.5 | 15.5 | 15.1 | 15.9 | 15.9 | 18.5 | 18.5 | 17.0 | 17.0 | 5.2 | 5.5 | 5.5 | 4.1 |  |  |  |  |  |  |  |  |  |  |  |  |  |  |  |  |
| 181 | *T. truongsonense_*6 | 14.2 | 14.6 | 13.5 | 13.4 | 12.6 | 14.2 | 13.9 | 13.2 | 13.2 | 13.2 | 13.9 | 14.6 | 13.9 | 13.9 | 13.9 | 13.9 | 13.9 | 13.9 | 13.9 | 14.2 | 13.2 | 16.3 | 16.3 | 14.6 | 14.6 | 5.6 | 6.6 | 6.6 | 2.1 | 3.3 |  |  |  |  |  |  |  |  |  |  |  |  |  |  |  |
| 182 | *T. truongsonense_*7 | 14.2 | 14.6 | 13.5 | 13.4 | 12.6 | 14.2 | 13.9 | 13.2 | 13.2 | 13.2 | 13.9 | 14.6 | 13.9 | 13.9 | 13.9 | 13.9 | 13.9 | 13.9 | 13.9 | 14.2 | 13.2 | 16.3 | 16.3 | 14.6 | 14.6 | 5.6 | 6.6 | 6.6 | 2.1 | 3.3 | 0.0 |  |  |  |  |  |  |  |  |  |  |  |  |  |  |
| 183 | *T. vietnamense_*1 | 13.9 | 14.6 | 15.3 | 19.7 | 18.9 | 16.3 | 16.0 | 15.3 | 16.3 | 16.3 | 14.6 | 13.9 | 14.6 | 14.6 | 14.6 | 14.6 | 14.6 | 14.6 | 14.6 | 14.2 | 17.0 | 19.8 | 19.8 | 9.0 | 9.0 | 15.6 | 14.6 | 14.6 | 14.6 | 15.5 | 13.9 | 13.9 |  |  |  |  |  |  |  |  |  |  |  |  |  |
| 184 | *T. vietnamense_*2 | 14.6 | 15.3 | 14.9 | 20.1 | 19.3 | 16.7 | 16.3 | 15.6 | 16.7 | 16.7 | 14.2 | 14.2 | 14.2 | 14.2 | 14.2 | 14.2 | 14.2 | 14.2 | 14.2 | 14.6 | 17.4 | 20.1 | 20.1 | 9.0 | 9.0 | 16.0 | 14.9 | 14.9 | 14.9 | 15.9 | 14.2 | 14.2 | 1.0 |  |  |  |  |  |  |  |  |  |  |  |  |
| 185 | *T. vietnamense_*3 | 14.9 | 15.6 | 17.0 | 20.9 | 20.1 | 16.7 | 16.3 | 16.3 | 18.1 | 18.1 | 15.6 | 14.9 | 15.6 | 15.6 | 15.6 | 15.6 | 15.6 | 15.6 | 15.6 | 15.3 | 16.7 | 19.4 | 19.4 | 10.4 | 10.4 | 17.0 | 16.0 | 16.0 | 16.3 | 17.3 | 15.6 | 15.6 | 2.4 | 2.8 |  |  |  |  |  |  |  |  |  |  |  |
| 186 | *T. vietnamense_*4 | 15.0 | 15.7 | 17.1 | 20.6 | 19.8 | 16.7 | 16.4 | 16.4 | 18.1 | 18.1 | 15.7 | 15.0 | 15.7 | 15.7 | 15.7 | 15.7 | 15.7 | 15.7 | 15.7 | 15.3 | 16.4 | 19.2 | 19.2 | 10.5 | 10.5 | 16.7 | 15.7 | 15.7 | 16.0 | 17.0 | 15.7 | 15.7 | 2.4 | 2.8 | 0.0 |  |  |  |  |  |  |  |  |  |  |
| 187 | *T. vietnamense_*5 | 14.9 | 15.6 | 16.0 | 20.1 | 19.3 | 16.3 | 16.0 | 16.0 | 17.0 | 17.0 | 14.9 | 14.9 | 14.9 | 14.9 | 14.9 | 14.9 | 14.9 | 14.9 | 14.9 | 15.3 | 17.0 | 19.8 | 19.8 | 9.4 | 9.4 | 16.3 | 15.3 | 15.3 | 15.3 | 16.2 | 14.6 | 14.6 | 2.1 | 1.7 | 2.4 | 2.4 |  |  |  |  |  |  |  |  |  |
| 188 | *T. vietnamense_*6 | 14.9 | 15.6 | 16.0 | 20.1 | 19.3 | 16.3 | 16.0 | 16.0 | 17.0 | 17.0 | 14.9 | 14.9 | 14.9 | 14.9 | 14.9 | 14.9 | 14.9 | 14.9 | 14.9 | 15.3 | 17.0 | 19.8 | 19.8 | 9.4 | 9.4 | 16.3 | 15.3 | 15.3 | 15.3 | 16.2 | 14.6 | 14.6 | 2.1 | 1.7 | 2.4 | 2.4 | 0.0 |  |  |  |  |  |  |  |  |
| 189 | *T. vietnamense_*7 | 14.9 | 15.6 | 16.0 | 20.1 | 19.3 | 16.3 | 16.0 | 16.0 | 17.0 | 17.0 | 14.9 | 14.9 | 14.9 | 14.9 | 14.9 | 14.9 | 14.9 | 14.9 | 14.9 | 15.3 | 17.0 | 19.8 | 19.8 | 9.4 | 9.4 | 16.3 | 15.3 | 15.3 | 15.3 | 16.2 | 14.6 | 14.6 | 2.1 | 1.7 | 2.4 | 2.4 | 0.0 | 0.0 |  |  |  |  |  |  |  |
| 190 | *T. vietnamense_*8 | 14.6 | 15.3 | 16.3 | 20.1 | 19.3 | 16.0 | 16.0 | 16.0 | 17.0 | 17.0 | 15.3 | 15.3 | 15.3 | 15.3 | 15.3 | 15.3 | 15.3 | 15.3 | 15.3 | 15.6 | 17.4 | 20.1 | 20.1 | 9.7 | 9.7 | 16.7 | 15.6 | 15.6 | 14.9 | 16.6 | 14.9 | 14.9 | 2.4 | 2.1 | 2.8 | 2.8 | 0.3 | 0.3 | 0.3 |  |  |  |  |  |  |
| 191 | *T. vietnamense_*9 | 14.6 | 15.3 | 16.3 | 20.1 | 19.3 | 16.0 | 16.0 | 16.0 | 17.0 | 17.0 | 15.3 | 15.3 | 15.3 | 15.3 | 15.3 | 15.3 | 15.3 | 15.3 | 15.3 | 15.6 | 17.4 | 20.1 | 20.1 | 9.7 | 9.7 | 16.7 | 15.6 | 15.6 | 14.9 | 16.6 | 14.9 | 14.9 | 2.4 | 2.1 | 2.8 | 2.8 | 0.3 | 0.3 | 0.3 | 0.0 |  |  |  |  |  |
| 192 | *T. vietnamense_*10 | 15.6 | 16.3 | 16.7 | 20.1 | 19.3 | 16.7 | 16.3 | 16.3 | 17.4 | 17.4 | 15.6 | 15.6 | 15.6 | 15.6 | 15.6 | 15.6 | 15.6 | 15.6 | 15.6 | 16.0 | 17.4 | 20.1 | 20.1 | 10.1 | 10.1 | 17.0 | 16.0 | 16.0 | 16.0 | 16.6 | 15.3 | 15.3 | 2.8 | 2.4 | 3.1 | 3.1 | 0.7 | 0.7 | 0.7 | 1.0 | 1.0 |  |  |  |  |
| 193 | *T. vietnamense_*11 | 14.9 | 15.6 | 16.0 | 20.1 | 19.3 | 16.3 | 16.0 | 16.0 | 17.0 | 17.0 | 14.9 | 14.9 | 14.9 | 14.9 | 14.9 | 14.9 | 14.9 | 14.9 | 14.9 | 15.3 | 17.0 | 19.8 | 19.8 | 9.4 | 9.4 | 16.3 | 15.3 | 15.3 | 15.3 | 16.2 | 14.6 | 14.6 | 2.1 | 1.7 | 2.4 | 2.4 | 0.0 | 0.0 | 0.0 | 0.3 | 0.3 | 0.7 |  |  |  |
| 194 | *T. vietnamense_*12 | 14.9 | 15.6 | 16.0 | 20.1 | 19.3 | 16.3 | 16.0 | 16.0 | 17.0 | 17.0 | 14.9 | 14.9 | 14.9 | 14.9 | 14.9 | 14.9 | 14.9 | 14.9 | 14.9 | 15.3 | 17.0 | 19.8 | 19.8 | 9.4 | 9.4 | 16.3 | 15.3 | 15.3 | 15.3 | 16.2 | 14.6 | 14.6 | 2.1 | 1.7 | 2.4 | 2.4 | 0.0 | 0.0 | 0.0 | 0.3 | 0.3 | 0.7 | 0.0 |  |  |
| 195 | *T. vietnamense_*13 | 14.9 | 15.6 | 16.0 | 20.1 | 19.3 | 16.3 | 16.0 | 16.0 | 17.0 | 17.0 | 14.9 | 14.9 | 14.9 | 14.9 | 14.9 | 14.9 | 14.9 | 14.9 | 14.9 | 15.3 | 17.0 | 19.8 | 19.8 | 9.4 | 9.4 | 16.3 | 15.3 | 15.3 | 15.3 | 16.2 | 14.6 | 14.6 | 2.1 | 1.7 | 2.4 | 2.4 | 0.0 | 0.0 | 0.0 | 0.3 | 0.3 | 0.7 | 0.0 | 0.0 |  |

**Table S8** Revised taxonomy of the subgenera and species groups in the *Theloderma*.

| ID | Subgenus | Species group | Species |
| --- | --- | --- | --- |
| 1 | Subgenus *Stelladerma* | *T.* *horridum* species group | *Theloderma* *horridum* |
| 2 |  |  | *Theloderma pseudohorridum* |
| 3 |  |  | *Theloderma stellatum* |
| 4 |  |  | *Theloderma vietnamense* |
| 5 | Subgenus *Theloderma* | *T. moloch* species group | *Theloderma moloch* |
| 6 |  |  | *Theloderma phrynoderma* |
| 7 |  |  | *Theloderma ryabovi* |
| 8 |  | *T. asperum* species group | *Theloderma albopunctatum* |
| 9 |  |  | *Theloderma asperum* |
| 10 |  |  | *Theloderma baibungense* |
| 11 |  |  | *Theloderma licin* |
| 12 |  |  | *Theloderma petilum* |
| 13 |  |  | *Theloderma pyaukkya* |
| 14 |  | *T. leporosum* species group | *Theloderma gordoni* |
| 15 |  |  | *Theloderma leporosum* |
| 16 |  | *T. lateriticum* species group | *Theloderma lacustrinum* |
| 17 |  |  | *Theloderma lateriticum* |
| 18 |  | *T. laeve* species group | *Theloderma annae* |
| 19 |  |  | *Theloderma laeve* |
| 20 |  |  | *Theloderma nebulosum* |
| 21 |  |  | *Theloderma truongsonense* |
| 22 |  | *T. corticale* species group | *Theloderma auratum* |
| 23 |  |  | *Theloderma bicolor* |
| 24 |  |  | *Theloderma corticale* |
| 25 |  |  | *Theloderma hekouense* |
| 26 |  |  | *Theloderma khoii* |
| 27 |  |  | *Theloderma nagalandense* |
| 28 |  |  | *Theloderma palliatum* |
| 29 |  |  | *Theloderma rhododiscus* |
