# Supplementary material for: Integrated phylogenetic analyses reveal the evolutionary, biogeographic, and diversification history of Asian warty treefrog genus Theloderma (Anura, Rhacophoridae)
Source: Ecol Evol. 2023 Dec 21;13(12):e10829. doi: 10.1002/ece3.10829 (PMC10739124; doi:10.1002/ece3.10829)
Supplement: Supplementary file 1 — Data S1 [file ECE3-13-e10829-s001.zip › Table S10.docx]

**Table S10** Revised taxonomy of the subgenera and species groups in the *Theloderma*.

| ID | Subgenus | Species group | Species |
| --- | --- | --- | --- |
| 1 | Subgenus *Stelladerma* | *T.* *horridum* species group | *Theloderma* *horridum* |
| 2 |  |  | *Theloderma pseudohorridum* |
| 3 |  |  | *Theloderma stellatum* |
| 4 |  |  | *Theloderma vietnamense* |
| 5 | Subgenus *Theloderma* | *T. moloch* species group | *Theloderma moloch* |
| 6 |  |  | *Theloderma phrynoderma* |
| 7 |  |  | *Theloderma ryabovi* |
| 8 |  | *T. asperum* species group | *Theloderma albopunctatum* |
| 9 |  |  | *Theloderma asperum* |
| 10 |  |  | *Theloderma baibungense* |
| 11 |  |  | *Theloderma licin* |
| 12 |  |  | *Theloderma petilum* |
| 13 |  |  | *Theloderma pyaukkya* |
| 14 |  | *T. leporosum* species group | *Theloderma gordoni* |
| 15 |  |  | *Theloderma leporosum* |
| 16 |  | *T. lateriticum* species group | *Theloderma lacustrinum* |
| 17 |  |  | *Theloderma lateriticum* |
| 18 |  | *T. laeve* species group | *Theloderma annae* |
| 19 |  |  | *Theloderma laeve* |
| 20 |  |  | *Theloderma nebulosum* |
| 21 |  |  | *Theloderma truongsonense* |
| 22 |  | *T. corticale* species group | *Theloderma auratum* |
| 23 |  |  | *Theloderma bicolor* |
| 24 |  |  | *Theloderma corticale* |
| 25 |  |  | *Theloderma hekouense* |
| 26 |  |  | *Theloderma khoii* |
| 27 |  |  | *Theloderma nagalandense* |
| 28 |  |  | *Theloderma palliatum* |
| 29 |  |  | *Theloderma rhododiscus* |
