# Supplementary material for: Integrated phylogenetic analyses reveal the evolutionary, biogeographic, and diversification history of Asian warty treefrog genus Theloderma (Anura, Rhacophoridae)
Source: Ecol Evol. 2023 Dec 21;13(12):e10829. doi: 10.1002/ece3.10829 (PMC10739124; doi:10.1002/ece3.10829)
Supplement: Supplementary file 1 — Data S1 [file ECE3-13-e10829-s001.zip › Table S9.docx]

**Table S9** Lowest, highest, and mean elevations of the three major clades within the *Theloderma*.

| Clade | Range | Mean ± SD |
| --- | --- | --- |
| Clade A | 10-1048 | 261 ± 258 |
| Clade B | 201-2805 | 1009 ± 551 |
| Clade C | 210-2606 | 1010 ± 541 |
